# Identification and validation of a 7-genes prognostic signature for adult acute myeloid leukemia based on aging-related genes

**DOI:** 10.18632/aging.204843

**Published:** 2023-06-26

**Authors:** Peng Ke, Qian Zhu, Ting Xu, Xiaofei Yang, Ying Wang, Huiying Qiu, Depei Wu, Xiebing Bao, Suning Chen

**Affiliations:** 1 National Clinical Research Center for Hematologic Diseases, Jiangsu Institute of Hematology, The First Affiliated Hospital of Soochow University, Suzhou, China; 2 Institute of Blood and Marrow Transplantation, Collaborative Innovation Center of Hematology, Soochow University, Suzhou, China; 3 Soochow Hopes Hematonosis Hospital, Suzhou, China

**Keywords:** aging-related genes, acute myeloid leukemia, immune characteristics, Pevonedistat, NEDD8

## Abstract

To explore effects of aging-related genes (ARGs) on the prognosis of Acute Myeloid Leukemia (AML), a seven-ARGs signature was developed and validated in AML patients. The numbers of seven-ARG sequences were selected to construct the survival prognostic signature in TCGA-LAML cohort, and two GEO datasets were used independently to verify the prognostic values of signature. According to seven-ARGs signature, patients were categorized into two subgroups. Patients with high-risk prognostic score were defined as HRPS-group/high-risk group, while others were set as LRPS-group/low-risk group. HRPS-group presented adverse overall survival (OS) than LRPS-group in TCGA-AML cohort (HR=3.39, *P*<0.001). In validation, the results emphasized a satisfactory discrimination in different time points, and confirmed the poor OS of HRPS-group both in GSE37642 (HR=1.96, *P*=0.001) and GSE106291 (HR=1.88, *P*<0.001). Many signal pathways, including immune- and tumor-related processes, especially *NF-κB* signaling, were highly enriched in HRPS-group. Coupled with high immune-inflamed infiltration, the HRPS-group was highly associated with the driver gene and oncogenic signaling pathway of *TP53*. Prediction of blockade therapy targeting immune checkpoint indicated varied benefits base on the different ARGs signature score, and the results of predicted drug response suggested that Pevonedistat, an inhibitor of *NEDD8*-activating enzyme, targeting *NF-κB* signaling, may have potential therapeutic value for HRPS-group. Compared with clinical factors alone, the signature had an independent value and more predictive power of AML prognosis. The 7-ARGs signature may help to guide clinical-decision making to predict drug response, and survival in AML patients.

## INTRODUCTION

Acute myeloid leukemia (AML) is a type of malignancy that causes malignant clonal proliferation due to genetic mutations and seriously endangers human health [1], and it is far more common in elderly patients [2]. In the era of traditional chemotherapy, elderly AML patients face a significantly poorer prognosis, when compared with younger AML patients [3]. With the recent advent of molecularly targeted therapies, survival has been improved for elderly AML patients, but the prognosis is still poor [4, 5].

In elderly AML population, prognosis is associated with multiple factors. Badness of health status could lead to poor tolerance of the effective treatment measures [6]. Lower therapeutic response and shorter duration of remission time in the elderly patients may also result in irregular treatment [6, 7]. Moreover, elderly AML patients often accompany with multiple abnormalities both in cytogenetics and molecular biology [8].

Aging is a complicated, continual, progressive, and time-dependent biological process that results in decreased physiologic function across all organ systems, eventually culminating in death; it is an irreversible and progressive process [9], including genomic instability, metabolic changes, loss of ability for stem cell self-renewal, and so on [10]. It seems to be that aging is an adverse prognostic element for cancers, but the exact mechanism is still obscure [10, 11]. More frequency of mutations happened in epigenetic modifiers as AML patients got older [12]. On the other hand, aging could accelerate drastic decrease of B cell diversity, which may promote oncogenesis in the elderly patients [13]. Genetics and intrinsic factors play important roles in influencing aging. With the development of genomics and transcriptome sequencing, as well as the application of high-throughput genetic screens, over 300 genes have been found to be involved in aging in humans alone [14].

Not only targeted therapy but survival prediction could be guided by signatures of aging-related genes (ARGs) in multiple different tumors [15–18]. Moreover, the primary markers of aging such as ARGs have been investigated widely, and the most main phenotypes contained mitochondrial dysfunction and immunological senescence [19]. To evaluate the effects of ARGs on prognosis of adult AML, we obtained ARGs from website of HAGR (human aging genome resource) [20] and assessed the association between ARGs and AML prognosis.

## RESULTS

### Construction of ARGs-related prognostic signature

The flow chart of this study was presented in Supplementary Figure 1. In training set (TCGA-LAML, n = 151), 83 (55.0%) were male and 68 (45.0%) were female, with a median age of 56 years at diagnosis. In patients with complete survival data (n = 140), the median time of OS was 577 days.

**Figure 1:**
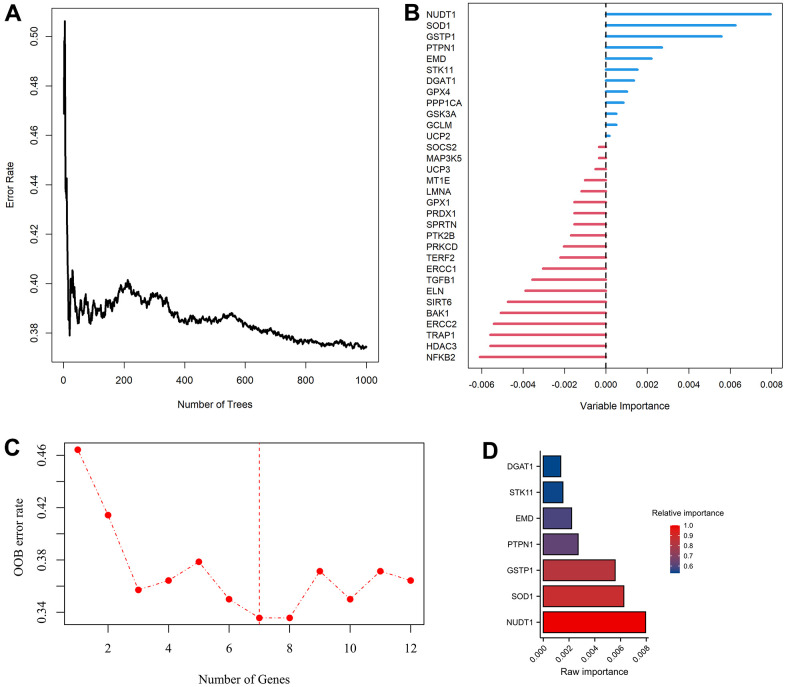
**Modelling of aging-related genes (ARGs) prognostic signature.** (**A**) Random survival forest algorithm for selecting hub ARGs. (**B**) The raw importance of the 32 candidate ARGs. (**C**) Weighted random forest (Ranger) was preformed to choose the optimization model with a lowest out of bag (OOB) error rate. (**D**) Seven ARGs with a relative importance greater than 0.52 were transferred to construct prognostic signature using COX regression model.

Univariate COX analysis demonstrated that 84 of 307 ARGs were associated with AML prognosis (Supplementary Figure 2). Via random survival forest algorithm (Figure 1A, 1B), we identified a 7-ARGs signature with a lowest OOB error rate for predicting AML survival (Figure 1C), Subsequently, seven ARGs with a relative importance greater than 0.52 were transferred to establish a prognostic signature for AML (Figure 1D). Table 1 described the coefficients and other parameters of these seven ARGs in detail. The Prognostic Risk Score (PRS) of signature was estimated as follows:


$$\begin{array}{l} \text{PRS}=0.466051\times \mathrm{PTPN1}+0.430832\times \mathrm{DGAT1}\\ \;\;\;+0.377577\times \mathrm{SOD1}+0.250209\times \mathrm{GSTP1}\\ \;\;\;+0.050049\times \mathrm{NUDT1}-0.001312\times \mathrm{EMD}\\ \;\;\;-0.443539\times \mathrm{STK1} \end{array}$$

**Table 1:** Multivariate COX regression analysis of 7 prognosis-related ARGs in AML.

**Ensemble gene ID**	**Symbol**	**Coefficient**	**Hazard ratio**	**Importance**	**Relative importance**
ENSG00000196396	PTPN1	0.466051	1.593688	0.008	1.000
ENSG00000185000	DGAT1	0.430832	1.538536	0.006	0.880
ENSG00000142168	SOD1	0.377577	1.458746	0.006	0.831
ENSG00000084207	GSTP1	0.250209	1.284294	0.003	0.627
ENSG00000106268	NUDT1	0.050049	1.051322	0.002	0.590
ENSG00000102119	EMD	-0.001312	0.998689	0.002	0.542
ENSG00000118046	STK11	-0.443539	0.641761	0.001	0.530

Abbreviations: ARGs, aging-related genes; AML, Acute Myelocytic Leukemia.

### Validation of 7-ARGs prognostic signature

**Figure 2:**
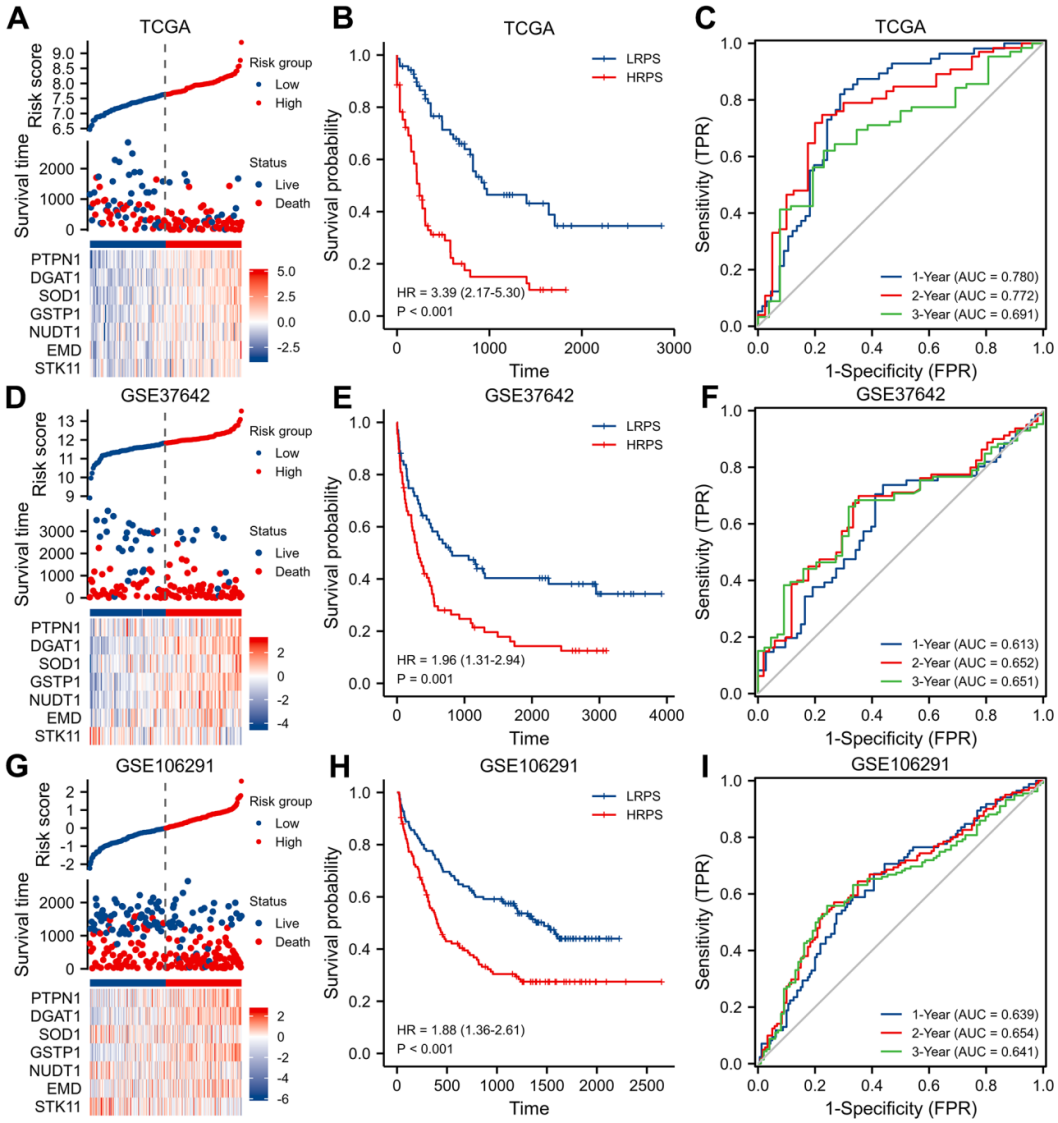
**Identification and validation of a seven-ARGs prognostic model.** (**A**) Plot of risk classification, survival status and heatmap of seven-ARGs; (**B**) curve of OS (Overall Survival) stratified by high-risk prognostic score (HRPS)-group/high-risk group and low-risk prognostic score (LRPS)-group/low-risk group; (**C**) curves of time-dependent ROC (receiver operator characteristic) in training set. (**D**–**F**) Plot of risk classification, survival status and heatmap of seven-ARGs; curve of OS; curves of time-dependent ROC in validation set of GSE37642. (**G**–**I**) Plot of risk classification, survival status and heatmap of seven-ARGs; curve of OS; curves of time-dependent ROC in validation set of GSE106291.

In training set, patients were divided into HRPS- and LRPS-group according to median value of PRS (Figure 2A). LRPS-group had a notably better OS than HRPS-group (Figure 2B, *P*<0.001). The median survival time of LRPS-group was 915 days (Q1: 303.75, Q3: 1193.5), whereas that of HRPS-group was 242 days (Q1: 52, Q3: 372.75). The area under curves (AUCs) meant that 7-ARGs signature has a good predictive performance (Figure 2C), with values of 0.780, 0.772 and 0.691 at 1-, 2-, and 3-year.

Then, two independent datasets were applied to verify the performance of this 7-ARGs signature. Patients in GSE37642 were stratified into two different risk groups in terms of the median PRS value too (Figure 2D). Similarly, LRPS-group significantly prolonged OS (Figure 2E, *P*=0.001), and 7-ARGs signature also had an ability to predict OS (Figure 2F). In GSE106291 cohort (Figure 2G), HRPS-group exhibited a worse OS than LRPS-group (Figure 2H, *P*<0.001), and values of AUCs at 1-, 2- and 3-year were 0.639, 0.654 and 0.641 (Figure 2I).

### GSEA analysis

**Figure 3:**
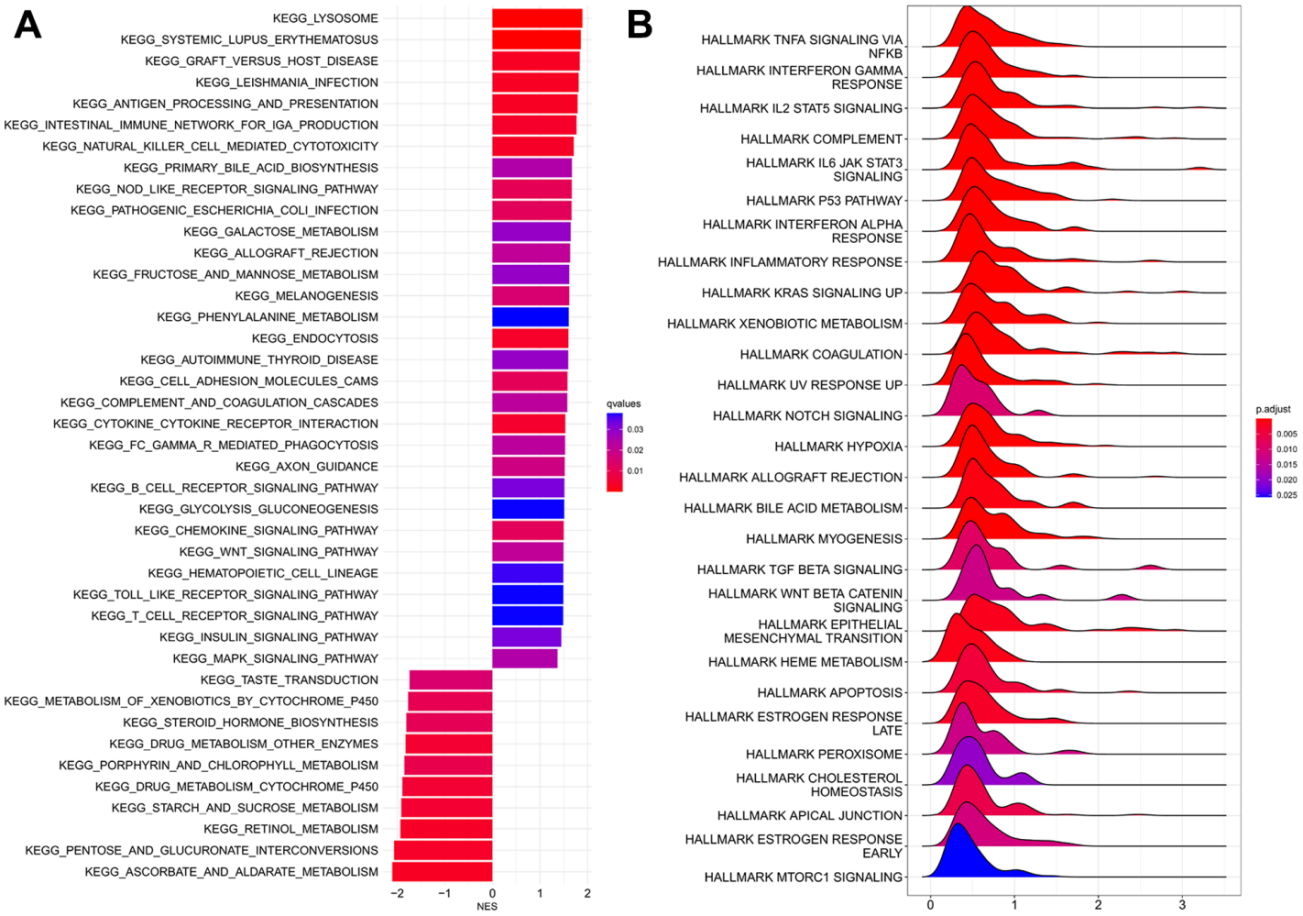
**GSEA analyses between HRPS-group/high-risk group and LRPS-group/low-risk group.** (**A**) KEGG analyses. (**B**) Analyses of hallmark pathways.

GSEA analysis of KEGG connoted that HRPS-group was enriched in many pathways, such as signaling of *WNT*, Toll-like receptor and *MAPK*. Similarly, many items of immune pathways were also enriched in HRPS-group (processing and presentation of antigen, interaction of cytokine and cytokine receptor, signaling pathway of Chemokine and T cell receptor) (Figure 3A).

The results of hallmark analysis suggested that many tumor-related signaling pathways were identified for HRPS-group (Figure 3B), such as *NF-κB* pathway in response to *TNF*, *IL-25* signaling pathway through *STAT5*, signaling pathway of *IL6/JAK/STAT3*, *p53*, *KRAS*, *notch*, and *TGF-β*.

### Characteristics of immune-related cells

**Figure 4:**
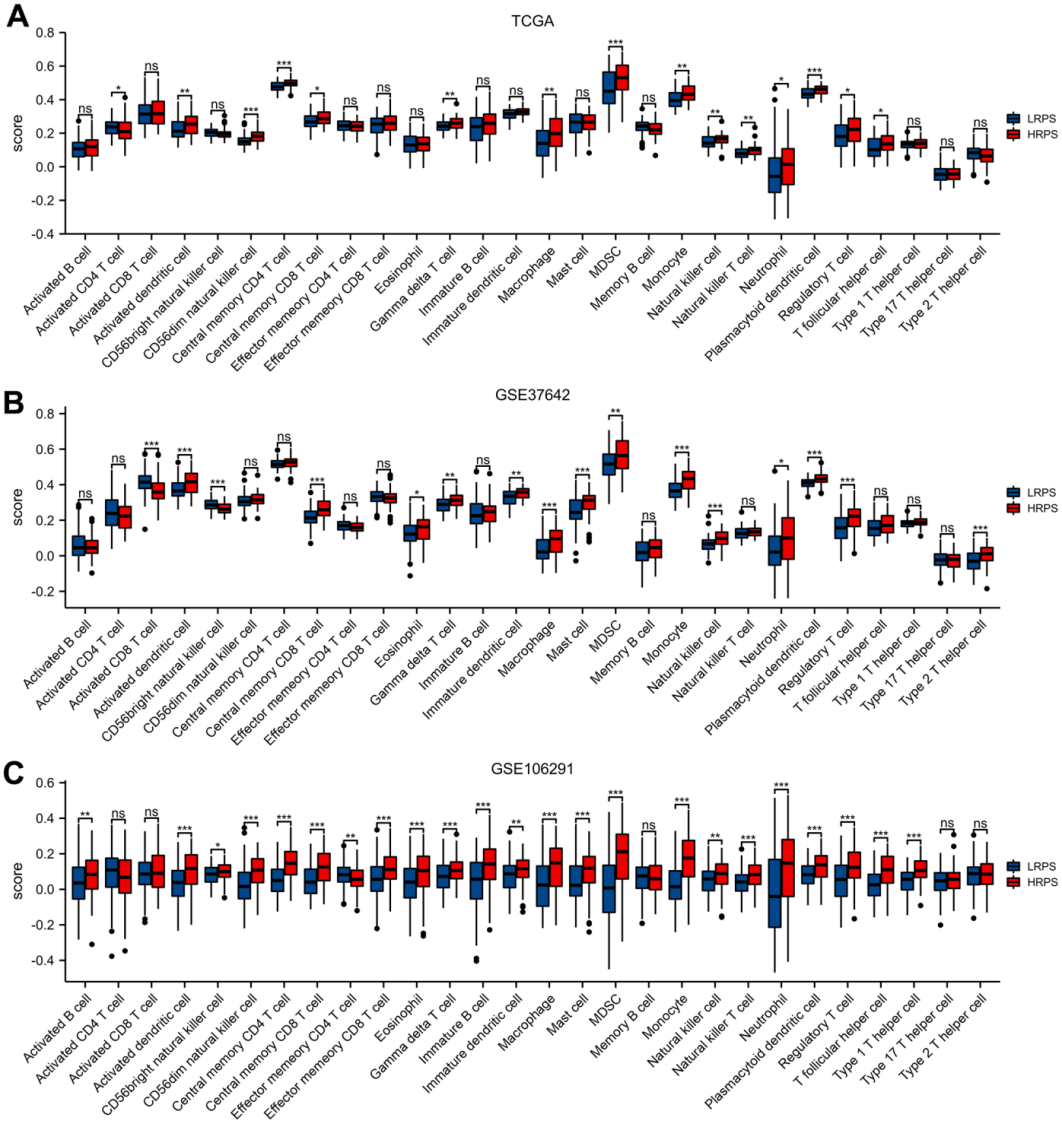
Different cell abundance of twenty-eight immune categories between HRPS-group/high-risk group and LRPS-group/low-risk group in training set (**A**) and validation set (**B**, **C**). (*P < 0.05, **P <0.01, ***P < 0.001).

The assessments above confirmed the prognostic value and biological function of the 7 ARGs-related signature, including many tumors and immune/inflammatory signaling pathways, prompting us to further explore the different proportion of immune-related cells between HRPS- and LRPS-group. HRPS-group had a higher ESTIMATE and immune score than LRPS-group (Supplementary Figure 3). The differences of immune-related features were displayed in Figure 4, and the results were almost consistent with each other in three AML cohorts (TCGA-AML, GSE37642 and GSE106291). HRPS-group were accompanied with higher scores of immune cells, such as MDSCs (myeloid-derived suppressor cells), many subtypes of T cell (central memory CD8 T, regulatory T, and Gamma delta T), Monocyte, macrophage, and etc. In addition, HRPS-group was characterized by increased enrichment of MHC I, Parainflammation, Type-I *IFN* Response and Check-point (Supplementary Figure 4).

Immune checkpoints play an important role in regulating immune infiltration [21], and up-regulated score of check-point molecule in HRPS-group might be a potential therapeutic target. So, we next explored the relevance of PRS and expression levels of 8 immune checkpoints, which indicated that *PDCD1* (R= 0.314), *LAG3* (R= 0.247), *CTLA4* (R= 0.199), and *CD274* (R= 0.198) had a positive relationship with PRS (Supplementary Figure 5).

### Scores of twelve subtypes in ARGs signal

To explore the distribution of ARGs subtypes and possible pathogenic mechanisms between HRPS- and LRPS-group, ssGSEA was performed to assess each score of 12 aging-related gene subsets. Results manifested that score of altered intercellular communication, deregulated nutrient sensing, *NF-κB* related gene and others were remarkably increased in the HRPS-group; whereas the score of stem cell exhaustion and genomic instability depressed inversely (Supplementary Figure 6).

### Mutation profile between HRPS- and LRPS-group

**Figure 5:**
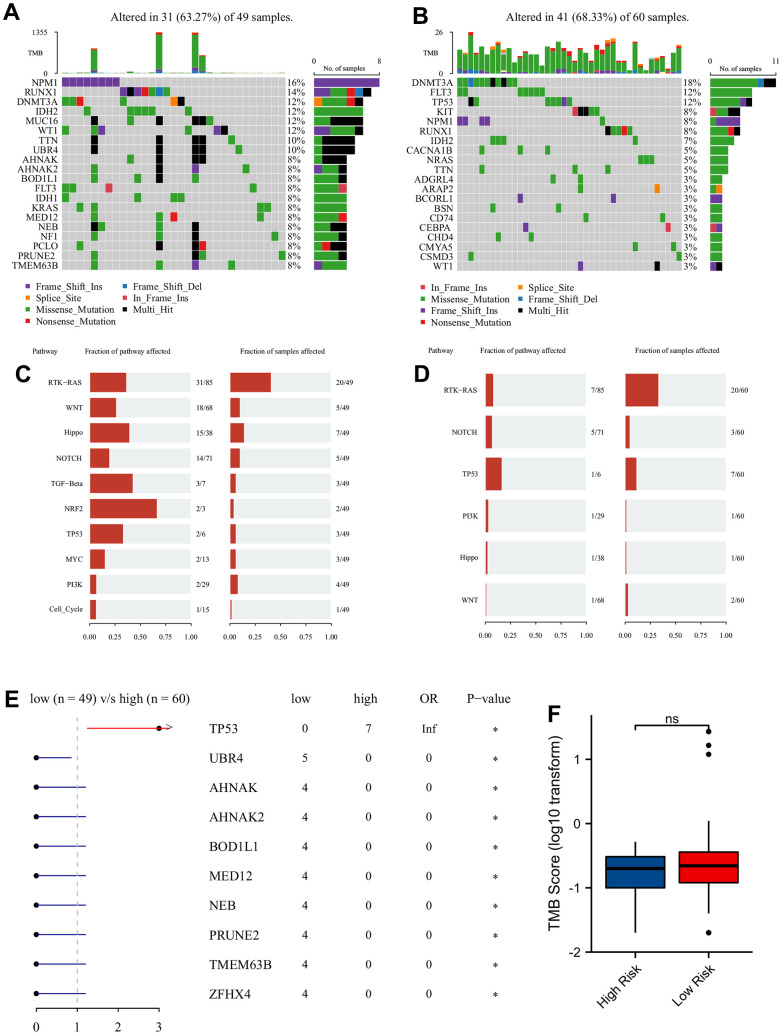
**Mutation analysis of two different prognostic risk score groups.** Oncoplot of top 20 high frequency of mutated genes in LRPS-group/low-risk group (**A**) and HRPS-group/high-risk group (**B**). The major mutation-enriched pathways in LRPS-group/low-risk group (**C**) and HRPS-group/high-risk group (**D**). Difference of mutated genes (**E**) and TMB score (**F**) in two different PRS groups.

The top 20 frequent genes of mutation were visualized in Figure 5. *NPM1* was the primary mutated gene in LRPS-group (Figure 5A), whereas *TP53*, *KIT* and *FLT3* mutated frequently in HRPS-group (Figure 5B). The results of Oncogenic Signaling Pathways revealed that LRPS-group displayed a higher frequency of mutations, which were enriched in many different pathways, including *NRF2*, *TGF-β*, *Hippo*, *MYC*, *PI3K* and Cell Cycle (Figure 5C). In HRPS group, *TP53* followed by *RAS* and *NOTCH* pathways were the most mutation-enriched pathways (Figure 5D). Forest plot confirmed that HRPS-group occurred more frequent *TP53* mutation (Figure 5E). However, no difference of TMB was found between these two groups (Figure 5F). PRS distributions showed that more patients with higher PRS were assigned to *FLT3-TKD* mutation (Supplementary Figure 7A), but those with lower PRS were more likely to appear in mutations of double *CEBPA* and *IDH2* (Supplementary Figure 7B, 7C). However, no differences of PRS distribution were found between patients with wild type and mutations of *FLT3-ITD, NPM1*, *IDH1*, *KRAS*, and *NRAS* (Supplementary Figure 7D–7H). These results implied that HRPS-group was highly associated with the driver gene and signaling pathway of *TP53*.

### High PRS patients may response to Pevonedistat

**Figure 6:**
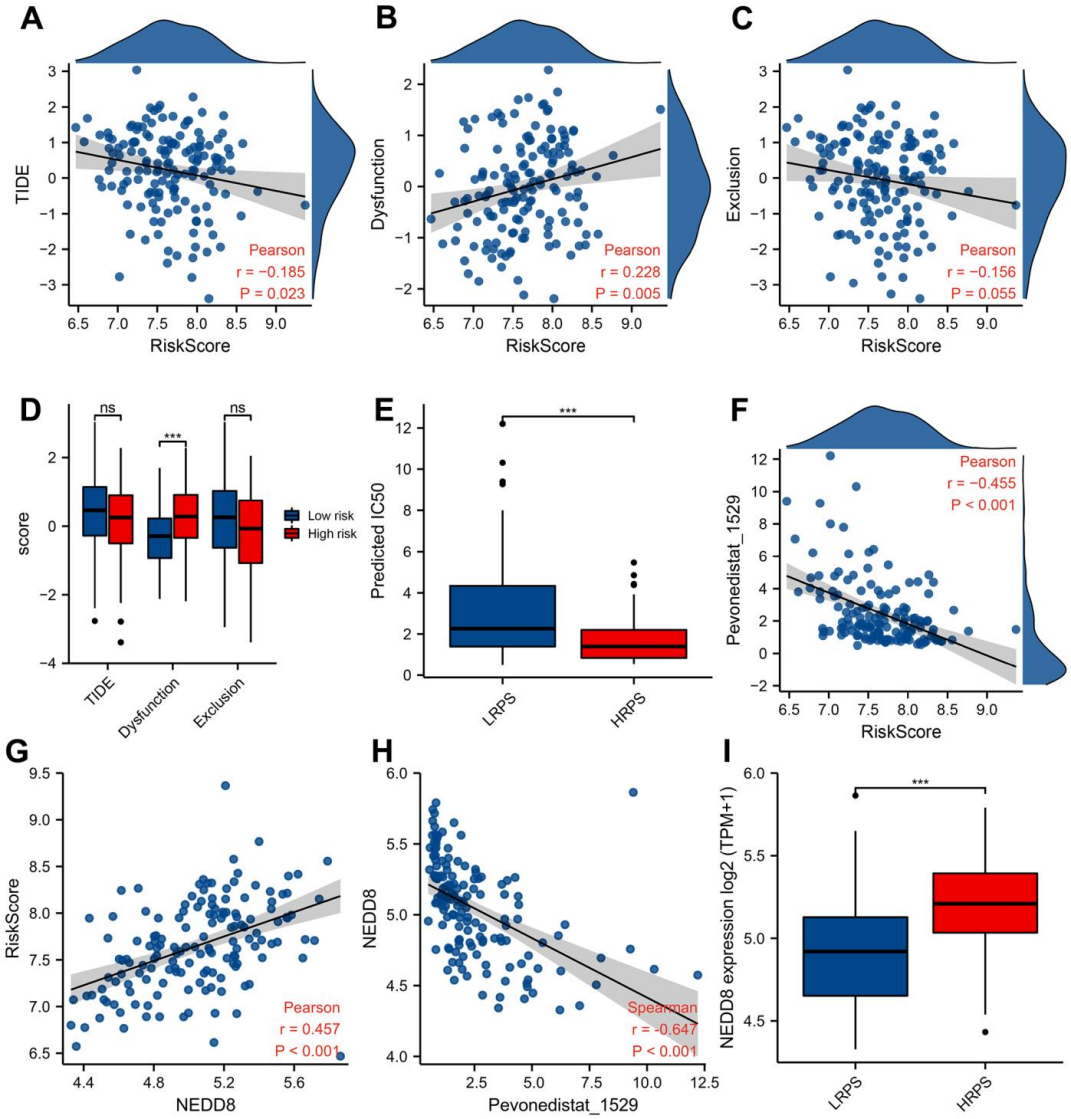
**Predictive response of checkpoint blocking-up and drug in training cohort.** Correlation between PRS and TIDE score (**A**), Dysfunction score (**B**), and Exclusion score (**C**). Boxplot of TIDE/Dysfunction/Exclusion score (**D**) and IC50 value of Pevonedistat (**E**) in HRPS-group/high-risk group and LRPS-group/low-risk group. (**F**) Relationship between Pevonedistat IC50 and PRS according to 7-ARGs signature, and an outlier was removed from curve of Pearson correlation analysis. Correlation analysis of PRS (**G**) and Pevonedistat’s IC50 (**H**) with the *NEDD8* expression level. Boxplot of *NEDD8* expression level in HRPS-group/high-risk group and LRPS-group/low-risk group (**I**).

The predicted response hinted that increased PRS was significantly correlated with decreased score of TIDE (Figure 6A, *P*=0.023) and increased dysfunction score (Figure 6B, *P*=0.005). No difference of TIDE score was seen between these two groups, while HRPS-group had a higher dysfunction score (Figure 6D, *P*<0.001). In addition, a markedly lower IC50 value of Pevonedistat was found in HRPS-group (Figure 6E, *P*<0.001), and other information of predicted drugs with potential therapeutic values could be downloaded in Supplementary Tables 7, 8. A negative and moderate correlation was also seen between IC50 level of Pevonedistat and PRS (Figure 6F, *P*<0.001).

Pevonedistat is an inhibitor of *NEDD8*-activating enzyme, the response of which is significantly correlated with expression level of *NEDD8*. To reveal the relationship between 7-ARGs signature and *NEDD8* expression level, Figure 6G demonstrated that *NEDD8* expression is positively related with PRS (R=0.457), and negatively (Figure 6H, R= -0.647) related with the predicted IC50 of Pevonedistat (*P*<0.001). Moreover, HRPS-group also had a higher *NEDD8* expression than LRPS-group (Figure 6I, *P*<0.001). These results suggested that Pevonedistat may be a potential drug for the HRPS-group.

### Development of a nomogram containing 7-ARGs signature

The clinical features of TCGA-LAML were described in Table 2. No significant differences were found between HRPS- and LRPS-group, including gender, race, gene mutation of *FLT3*, *RAS*, *NPM1* and *IDH1*. However, adverse-risk of cytogenetics and older patients were occurred frequently in HRPS-group (P<0.05).

**Table 2:** The characteristics of 151 acute myeloid leukemia patients in TCGA database.

**Characteristic**	**Low-risk (n=76)**	**High-risk (n=75)**	**P value**
Gender			0.284
Female	38 (25.2%)	30 (19.9%)	
Male	38 (25.2%)	45 (29.8%)	
Race			1.000
Asian or unavailable	2 (1.3%)	1 (0.7%)	
Black or African American	7 (4.6%)	6 (4.0%)	
White	67 (44.4%)	68 (45.0%)	
Age (Median [IQR]) years	51 [35.8, 62.0]	62 [47.5, 69.5]	0.001
Cytogenetics risk			0.004
Favorable	22 (14.8%)	9 (6.0%)	
Intermediate	43 (28.9%)	39 (26.2%)	
Adverse	11 (7.4%)	25 (16.8%)	
*FLT3* mutation			0.956
Negative	52 (35.4%)	50 (34.0%)	
Positive	22 (15.0%)	23 (15.6%)	
*RAS* mutation			0.719
Negative	71 (47.3%)	71 (47.3%)	
Positive	5 (3.3%)	3 (2.0%)	
*NPM1* mutation			1.000
Negative	59 (39.3%)	58 (38.7%)	
Positive	17 (11.3%)	16 (10.7%)	
*IDH1* mutation			0.593
Negative	61 (40.9%)	62 (41.6%)	
Positive	15 (10.1%)	11 (7.4%)	
PB-blast (%)	70 [51.0, 85.0]	73 [48.0, 85.0]	0.994
BM-blast (%)	45.5 [12.5, 70.3]	23 [6.0, 56.0]	0.065
WBC (×10^9^/L)	14.5 [4.0, 42.3]	26.5 [6.0, 61.3]	0.214
Hemoglobin (g/L)	90 [60, 130]	90 [60, 130]	0.940
PLT (×10^9^/L)	45.5 [27.3, 87.3]	44 [30.5, 83.5]	0.669

Abbreviations: TCGA, The Cancer Genome Atlas; IQR, Interquartile range; FLT3, FMS-like tyrosine kinase 3; NPM1, Nucleophosmin; IDH1, Isocitrate dehydrogenase 1; PB, Peripheral blood; BM, Bone marrow; WBC, White blood cell; PLT, Platelet.

**Figure 7:**
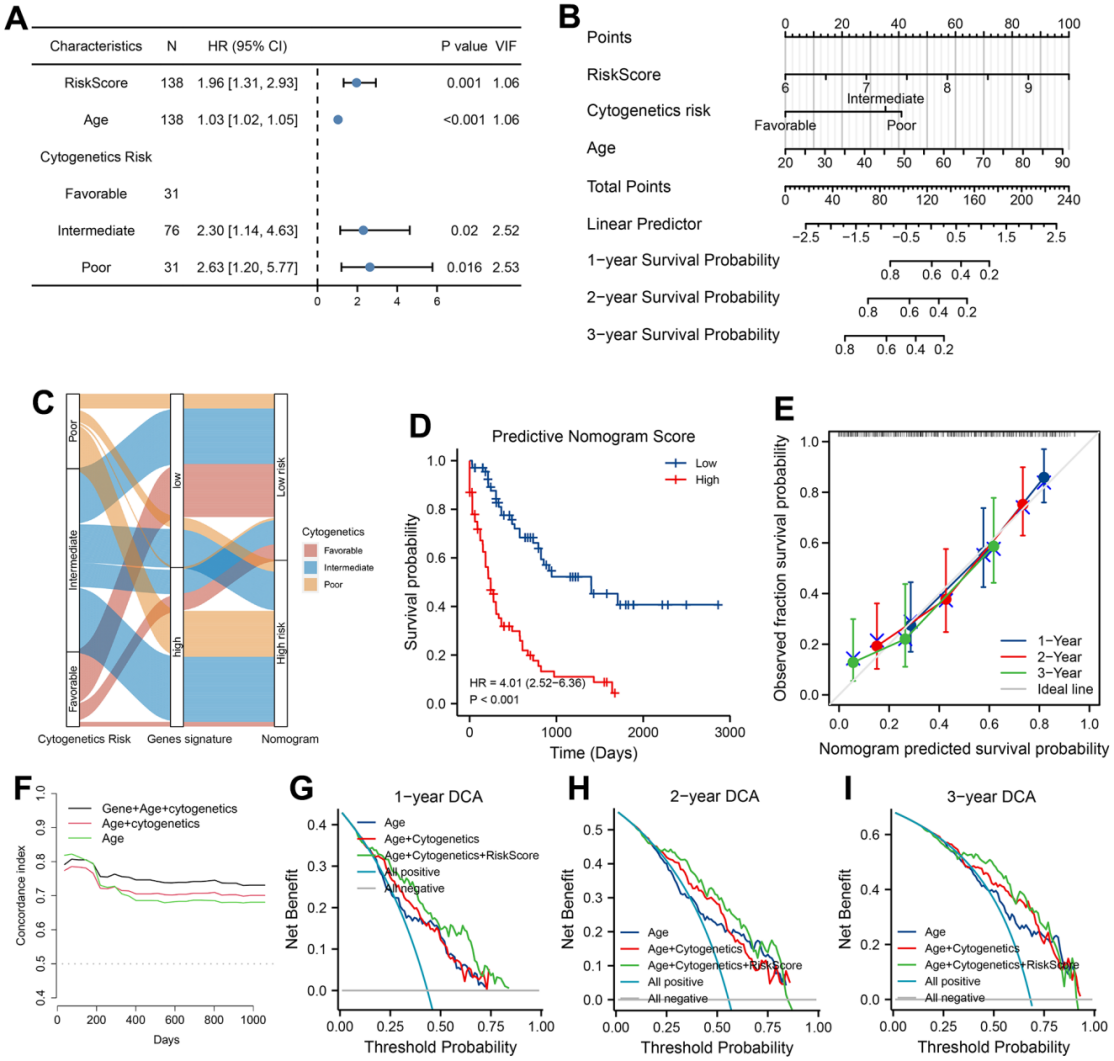
**Development, validation and clinical use of predicted nomogram.** (**A**) Prognostic analysis of Nomogram. VIF indicated Variance inflation factor. (**B**) Construction of Nomogram according to prognostic factors of AML patients in training cohort. Values of prognostic risk Score (PRS) were calculated by 7-ARGs signature. (**C**) Sankey diagram of transitions from cytogenetics risk category to reclassification of 7-ARGs signature and Nomogram. (**D**) Analysis of survival relying on stratification of Nomogram. (**E**) Calibration curve of Nomogram at 1-, 2-, and 3-year. Discrimination with C-statistic at different time (**F**) and DCA plot at 1-, 2- and 3-year (**G**–**I**) for Nomogram and clinical features (model with age alone or with age plus cytogenetics risk).

To seek clinical applicability, we constructed a predicted Nomogram composed of 7-ARGs signature in training set. Results of univariate and multivariable survival analysis could be found in Supplementary Table 9 and Figure 7A. All predictors accompanied with a VIF value less than 5, which meant that no collinearity happened in the predicted nomogram. Combining with age, cytogenetics risk and PRS of ARGs signature, a nomogram was constructed for predicting AML prognosis (Figure 7B). According to the nomogram, the cytogenetics risk stratification can be further reclassified (Figure 7C), and prognostic difference of OS was well accounted too (Figure 7D, *P*<0.001). Good performance was verified by calibration plots (Figure 7E).

Moreover, plots of C-statistic demonstrated a good discrimination (Figure 7F), with a value of 0.754, 0.741 and 0.731 at 1-, 2- and 3-year, respectively. Compared with the model of age or combined model with age plus cytogenetics risk, higher C-statistic values were seen in predicted nomogram (Figure 7F). With regard to DCA analysis at 1-, 2- and 3-year, various degrees of benefits were presented for nomogram prediction than two other models. Moreover, using the nomogram to predict AML prognosis could add more benefits than either the strategy of intervene-none or intervene-all-patients if the probability is higher than the threshold (Figure 7G–7I).

## DISCUSSION

AML is a common malignant tumor in the elderly [2], and these group of patients were associated with poor prognosis [22–24]. However, the mechanism how aging affects AML prognosis is not quite clear. A prognostic signature base on expression level of 7-ARGs was established and verified in this study, which can well classify AML patients into two risk groups with different prognosis.

In the 7-ARGs signature, *PTPN1*, *DGAT1*, *SOD1*, *GSTP1*, and *NUDT1* acted as risk factors, whereas *EMD* and *STK11* were protective factors. Some of them were confirmed with the formation and progression of AML. Overexpression of *GSTP1* is linked with drug resistance in AML [22]. *STK11* is a tumor suppressor gene, which can encode kinase B1 in liver (*LKB1*) [23], and pathway regulated by which was highly associated with tumor suppression in AML [24]. Although there are no definite investigations of the roles other five ARGs played in AML, these genes will be worthy of further study. The 7-ARGs signature exhibited a good performance in predicting overall survival both in the training and validation cohorts. Adjusted by clinical features, 7-ARGs signature acted as an independent risk factor of AML prognosis. Furthermore, the nomogram based on 7-ARGs signature both took on a good calibration and discrimination, which can also help to make clinical decisions.

Functional analyses of 7-ARGs signature emphasized that HRPS-group was enriched in pathways of processing and presentation of antigen, interaction of cytokine receptor, and Toll-like receptor, which could help to promote AML progression [25]. Activation of inflammatory pathways can also lead to myeloproliferative disease by increasing levels of cytokine and ROS (reactive oxygen species) [26].

GSEA analysis revealed that signaling pathways of *NF-κB* and *KRAS* were significantly activated in HRPS-group. AS a member of transcription factors, *NF-κB* is widely involved in many biological processes, such as responses of immune and inflammation, proliferation, differentiation, cell survival, and all of which were strongly linked to survival of AML stem cells [27–31]. Results *in vivo* and *vitro* also verified that inhibiting pathway of *NF-κB* could provoke cell death in AML, while this phenomenon was not obvious in normal bone marrow stem cells [31, 32]. Moreover, abnormal activation of *NF-κB* signal has been proven to involve in the process of AML resistance to chemotherapeutic agents [33]. In addition, excessive activation of *RAS* signaling is also considered to be one of the primary pathogenic items of AML [34]. All these may help to account for poor prognosis of AML patients in HRPS-group.

Aging changed the status of immunity and microenvironment as always, which might also facilitate tumor progression [35]. Our results certified that HRPS-group had significantly a higher score of ESTIMATE and immunity. Consistent with previous studies [36], our results suggested that higher immune score could also lead to adverse prognosis. HRPS-group had more increased components of immune suppression by myeloid cells, monocyte and macrophage, which could also lead to a poor survival rate. TAMs (tumor-associated macrophages) could promote leukemic stem cells proliferation and immortality via diverse mechanisms, including angiogenesis, extracellular matrix remodeling, and lymphangiogenesis [37, 38]. Immune suppression by myeloid cells, one of MDSCs, could also block the anti-tumor effects by multiple mechanisms, which may also help to explain to some extent why HRPS-group was accompanied with poor prognosis [39].

The aging process affects not only immune system by enhance or suppress of immune cell function, but also immune supervision of checkpoints [40, 41]. Nowadays, therapy of blocking checkpoint has led to breakthroughs in cancer therapy, such as lymphomas, lung cancer and melanoma [42]. Although studies of immune checkpoint therapy in AML were limited, some studies still had encouraging results [42–44]. It is of great significance to find reliable predictive biomarkers and therapeutic targets for AML with different risks. Positive correlations were found between PRS from ARGs signature and four expression genes of checkpoints (*PDCD1*, *CTLA4*, *LAG3* and *CD274*). TIDE algorithm also revealed that higher 7-ARGs signature score was highly in relation to lower TIDE score, and lower TIDE score denoted more benefits from immune checkpoint therapy. No difference of TIDE score between two risk groups may due to higher dysfunction of T cells in HRPS-group, which may limit benefits of immune checkpoint therapy. Furthermore, Pevonedistat was predicted as a potential therapeutic drug for patients in HRPS-group. In 7-ARGs signature, *NF-κB* was the primary signaling with abnormal activation in HRPS-group, while Pevonedistat can inhibit *NF-κB* signaling [45], those might help to explain our results of drug prediction. Pevonedistat may be more suitable for AML with higher prognostic score of 7-ARGs signature.

Interestingly, subtype analysis of ARGs illustrated a consistent result with enrichment analysis. HRPS-group had a higher score of ssGSEA in *NF-κB* related gene set, which was the primary enrichment pathway in GSEA analysis. Furthermore, deregulated nutrient sensing were significant associated with humans’ longevity [46]. Inflammation is one of the prominent alterations in aging-associated communication of intercellular space. This immunosenescence may affect the normal function of the body, dysfunction of clearing abnormal and/or infected cells, especially for tumor cells [10]. This suggests that altered intercellular communication may participate in modulating tumor development. Higher scores both in purity of tumor and genomic instability in LRPS-group may explain why this group of patients had a higher ssGSEA score of stem cell exhaustion. In addition, patients in HRPS-group had more frequent mutation of *TP53,* which was confirmed with poor prognosis in AML patients [47]. Accordingly, LRPS-group had a higher frequency of mutation in *UBR4*, *AHNAK*, *BOD1L1*, and etc. In Bladder Cancer, mutation of *AHNAK* is one sign of favorable prognosis [48]. Some of them, including *MED12*, *NEB*, and *ZFHX4*, were considered to be poor prognostic factors in some solid tumors [49–51], but the role of which in AML is unclear.

However, our current study also had some limitations. Firstly, although the effectiveness of 7-ARGs prognostic signature has been proved both in training and validation datasets, the applied AUC of validation set is unsatisfactory, which might be on account of different sequencing platforms. The training cohort is obtained from RNA-sequencing, while the validation set (GSE37642) is generated from microarray platform. Although the AUC values of validation cohorts are lower than that of the training set, predictive power is still exhibited in validation sets according to the curves of ROC and Kaplan-Meier survival analysis. Secondary, more independent cohorts and experimental studies (*in vitro* or *in vivo*) are required to validate the effectiveness and disclose underlying mechanisms. In the future, the exact prognostic values of these ARGs deserve further study in AML.

## CONCLUSIONS

In conclusion, we identified and validated an aging-related risk signature based on seven-ARGs for predicting AML prognosis, and also tried to reveal the possible mechanisms from many aspects. These findings could be helpful to improve diagnosis, risk stratification or treatment of AML.

## MATERIALS AND METHODS

### Source of data

TCGA-LAML cohort was downloaded as the training set for constructing prognostic signature base on ARGs, including publicly available gene expression and clinical data. The RNA-seq data of which was standardized by Transcripts Per Kilobase of exon model per Million mapped reads (TPM) and normalized as log2 (TPM + 1). Two independent AML datasets of GEO, including GSE37642 (GPL570 platform, n = 136) and GSE106291 (n = 250), were also adopted to validate the reliability of prognostic signature. Human ARGs were obtained from the website of HAGR (Supplementary Table 1, n = 307) (https://www.genomics.senescence.info/). Moreover, GSE14468 (n = 461) was also acquired to further compare the differences of PRS between several gene mutations, including *NPM1*, *IDH1*, *IDH2*, *KRAS*, *NRAS*, *CEBPA*, *FLT3-ITD*, and *FLT3-TKD*. All data used in this study were conducted in accordance with the publication guidelines, as a result, informed consent and ethical approval were not required.

### Development and validation of a 7-ARGs signature for AML prognosis

According to the median value of gene expression, univariate COX analysis was carried out to select ARGs with prognostic values in the training set (Supplementary Table 2). A total of 48 ARGs were associated with AML prognosis (Supplementary Table 3, *P*<0.05), with 35 of adverse prognostic ARGs and 13 of favorable prognostic ARGs. Thirty-two of 35 adverse prognostic ARGs were chosen as candidate genes, which were co-expressed in GSE37642 cohort. The importance of 32 ARGs was ranked using a model of random survival forest (ntree=1000, “randomForestSRC” R package) [20]. To choose the optimal model for constructing prognostic signature, 12 of 32 ARGs with a positive raw importance were chosen and screened using method of weighted random forest (“Ranger”, “randomForest” and “survival” R packages). Finally, seven-ARGs signature was identified with a lowest out of bag (OOB) error rate in the training set, and seven ARGs with a relative importance greater than 0.52 were transferred to further assessment using by COX regression model. The expression level and COX regression coefficient of ARGs were used to calculate the prognostic risk score (PRS) as below:


$$\text{Prognostic}\;\text{risk}\;\text{score}\;(\text{PRS})=\sum_{i=1}^{n}\text{Coef}(i)\;X(i)$$

where n indicates the number of ARGs included in the prognostic signature, Coef(i) means the coefficient of each ARGs in COX regression, and X(i) represents the expression level of each ARGs. In the light of median PRS value, AML patients were categorized into two subgroups. Patients with high value of PRS were defined as HRPS-group/high-risk group, while others were deemed as LRPS-group/low-risk group. Curves of survival analysis and time-dependent ROC analysis were performed to evaluate the survival differences and discrimination of ARGs signature, using R packages of “survival”, “ggrisk”, “survminer” and “timeROC”.

### Enrichment analyses

To assess biological functions and signaling pathways associated with HRPS-group, gene set enrichment analysis (GSEA) was carried out using programs of Hallmark and c2 KEGG from MSigDB. The significant difference was set as |normalized enrichment score| >1, False Discovery Rate < 0.25, and the adjusted *P* value lower than 0.05.

### Immune characteristics of microenvironment

Differences of immune-related characteristics were analyzed between the HRPS- and LRPS-group, adopting R package of ESTIMATE for calculating stromal-score, immune-related score, purity of tumor, and ESTIMATE-related score [52]. Meanwhile, using R packages of GSVA and GSEABase, ssGSEA was performed to quantify different types of immune-related cells. The immune-related gene sets were collected from previous studies (Supplementary Tables 4, 5) [53, 54]. To further identify the potential therapeutic targets of checkpoints for ARGs signature, we also evaluated the correlations of PRS with the expressed levels of 8 immune-related checkpoints (*HAVCR2*, *SIGLEC15*, *PDCD1*, *PDCD1LG2*, *CD274*, *LAG3*, *CTLA4* and *TIGIT*).

### ssGSEA analysis of twelve aging-related gene signals

According to previous studies [55], all ARGs can be divided into 12 sub-categories, which may help to conceptualize the essence of cancers and their underlying mechanisms. To depict the distributions of different subtypes and possible pathogenic mechanisms between these two different risk groups, ssGSEA was also performed in 12 aging-related gene sets from National Genomics Data Center (Supplementary Table 6, https://ngdc.cncb.ac.cn), including *NF-κB* related gene, senescence-associated secretory phenotype, stem cell exhaustion, telomere attrition and others. We compared the difference of 12 ssGSEA scores between the HRPS- and LRPS-group.

### Mutational features

To better understand the difference of driver genes between HRPS- and LRPS-group, the somatic mutations of TCGA-LAML were acquired and analyzed using R package of “maftools” [56]. The mutational landscape of two different PRS groups were investigated by Fisher exact test, and visualized graphically using by waterfall curve and forest plot. Enrichment analysis of Oncogenic Signaling Pathways was also performed to exhibit the difference between these two groups. Moreover, recent studies have demonstrated that a higher tumor mutated burden (TMB) was strongly associated with a better anti-tumor immune response [57], so we also compared the difference of TMB between HRPS- and LRPS-group. In addition, the distributions of PRS between different gene mutations were also compared and visualized through box plots.

### Prediction of response to checkpoint blocking-up and drugs

To further probe whether HRPS-group can benefit from checkpoint blocking-up and potential drugs, possibility of response to immunotherapy was predicted using an algorithm reported previously, including dysfunction and exclusion of anti-tumor immune (TIDE) [58]. The predicted responses to drugs were done using the R package “oncoPredict” based on version 2 database of Genomics of Drug Sensitivity in Cancer [59]. Using PRS from ARGs signature, Pearson correlation analysis was used to scan the candidate drugs, which had a *P* value less than 0.001 and correlation coefficient less than -0.3. Correlation coefficients reflected the relations between PRS and IC50 of candidate therapeutic agents. And then the differences of candidate agents were also compared between HRPS- and LRPS-group.

### Construction of a nomogram using 7-ARGs signature

To appraise clinical applicability, we established a nomogram using variables with independent prognostic values in multivariable analysis of COX proportional hazards regression model in the training set. Variance inflation factor (VIF) was calculated to test the collinearity among predictors, and variables with a VIF value less than 5 were excluded from collinearity. Curves of C-statistic and calibration were draw to demonstrate the efficacy and accuracy of nomogram, while decision curve analysis (DCA) was executed for evaluation of clinical utility.

### Statistical settings

Statistical processes were conducted by related packages of R 4.1 software. Tests of Fisher exact/Chi-square were run to analyse categorical variables, whereas tests of Kruskal-Wallis/Wilcoxon rank-sum were done for numerical variables. Curves of Kaplan-Meier with test of Log-rank/COX analysis were utilized to estimate the difference of overall survival (OS). When the values of *P* less than a threshold (< 0.05), the results were deemed to be statistically different.

## Supplementary Material

Supplementary Figures

Supplementary Table 1

Supplementary Table 2

Supplementary Table 3

Supplementary Table 4

Supplementary Table 5

Supplementary Table 6

Supplementary Table 7

Supplementary Table 8

Supplementary Table 9
