# Supplementary material for: Identification and validation of a 7-genes prognostic signature for adult acute myeloid leukemia based on aging-related genes
Source: Aging (Albany NY). 2023 Jun 26;15(12):5826–53. doi: 10.18632/aging.204843 (PMC10333094; doi:10.18632/aging.204843)
Supplement: Supplementary Figures [file aging-15-204843-s001.pdf]

## SUPPLEMENTARY FIGURES

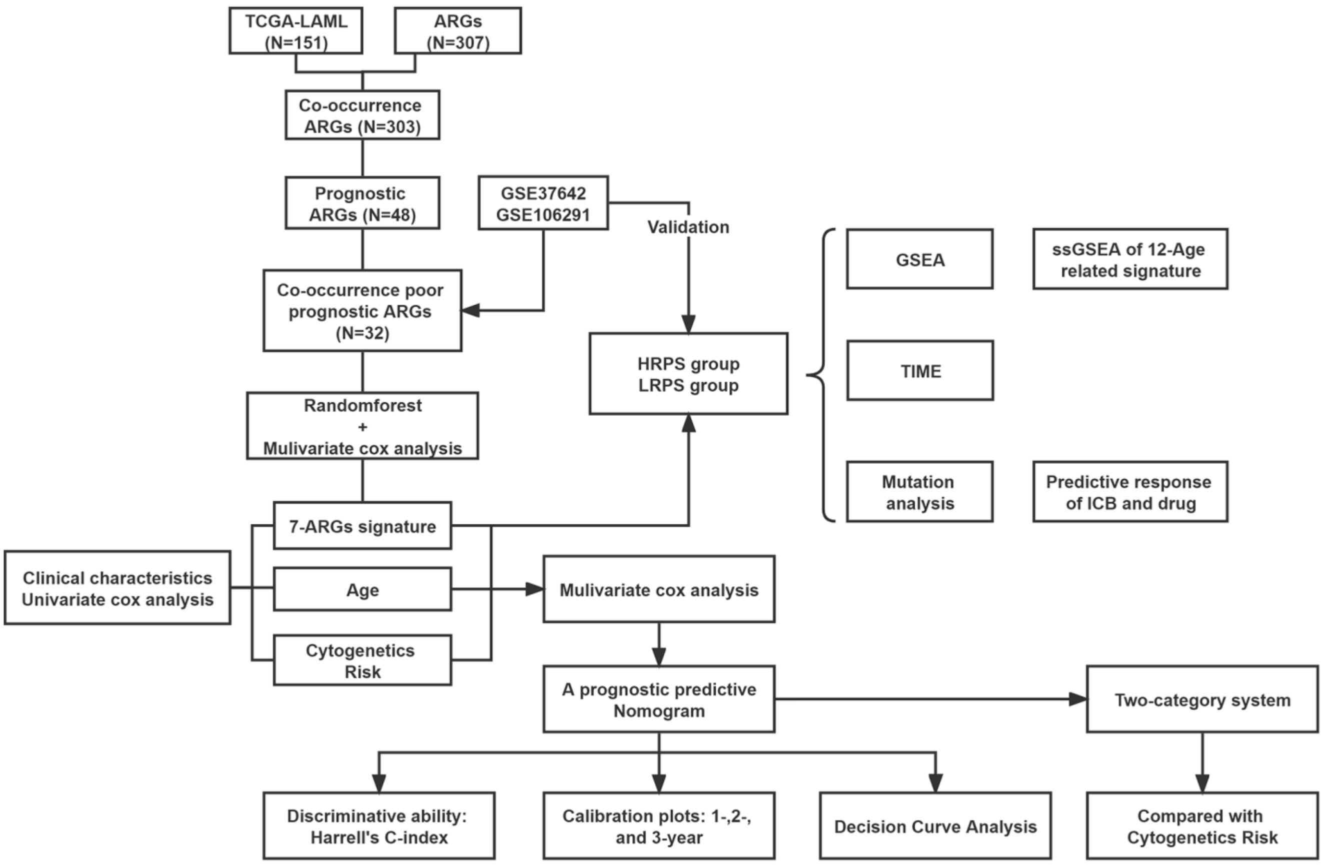


**Supplementary Figure 1. Work flow of the current study.** ARGs indicated aging-related genes. TIME indicated immune microenvironment of tumor.

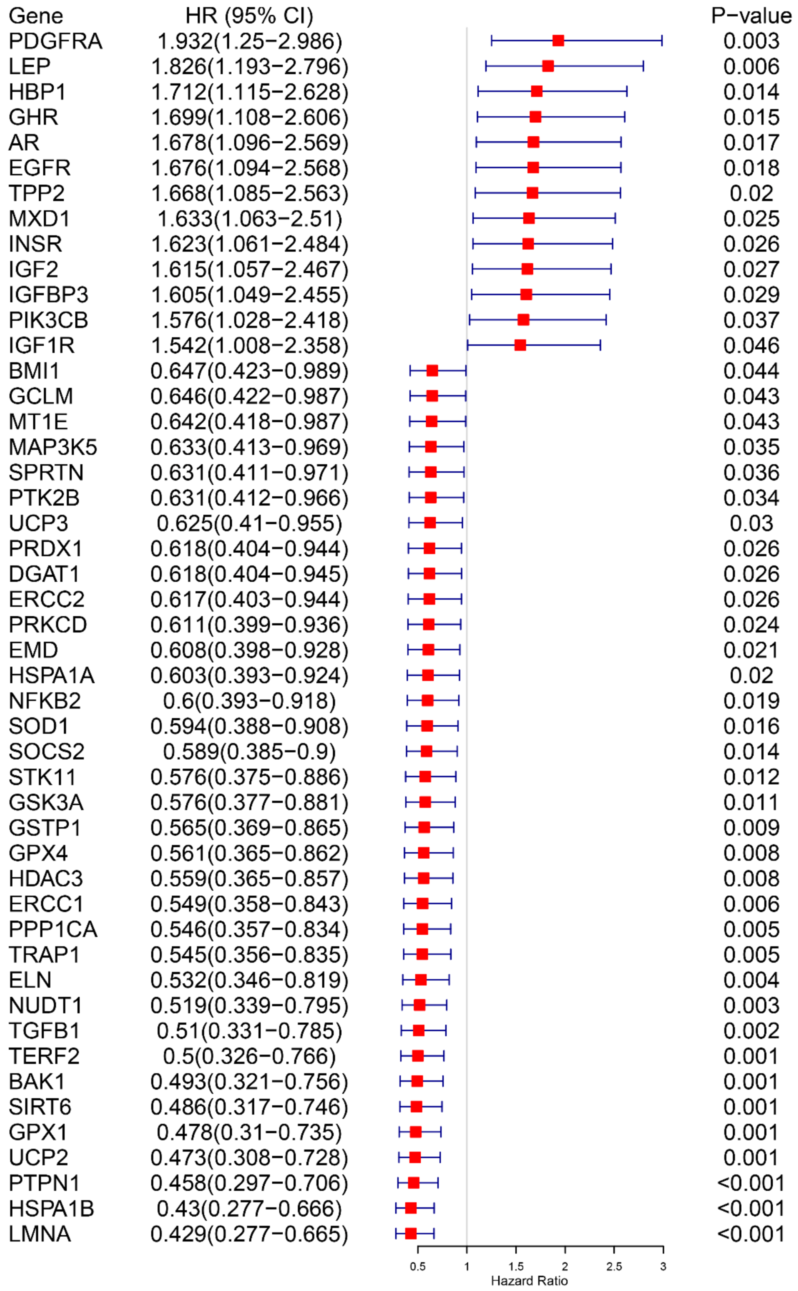


**Supplementary Figure 2. Prognostic analyses of 84 ARGs in training cohort.** Median expression values of genes were used as the threshold values, and patients with high value than which were set as a reference group.

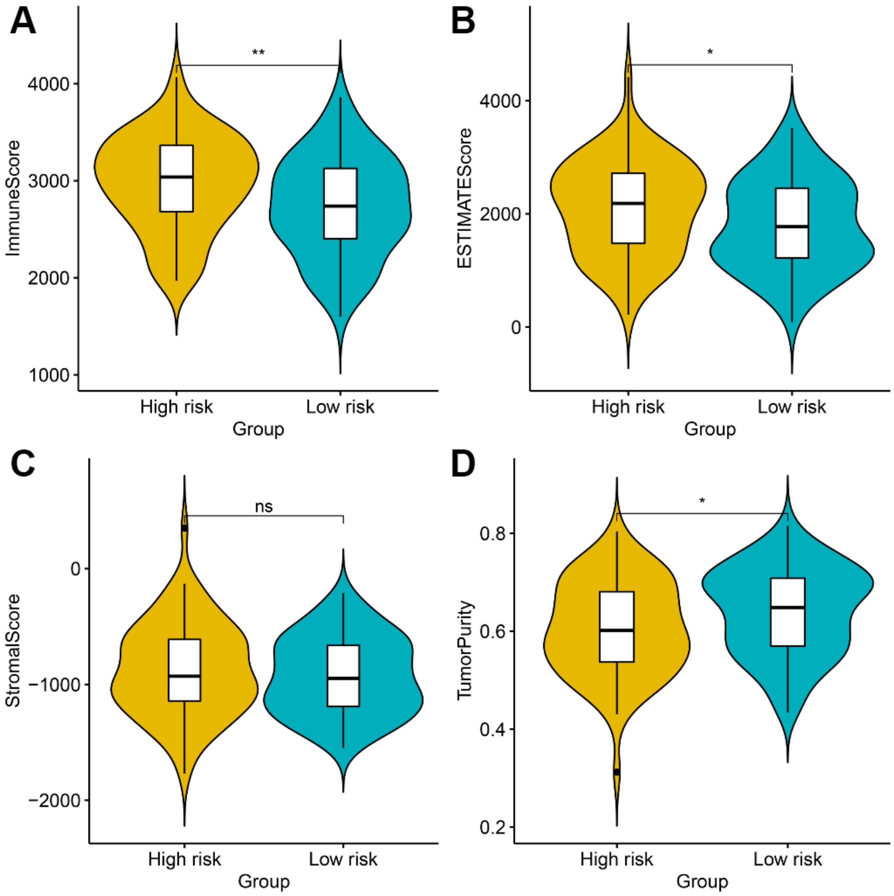


**Supplementary Figure 3. Characteristics of immune-related microenvironment in HRPS-group/high-risk group and LRPS-group/low-risk group.** (**A**) Immune Scores. (**B**) ESTIMATE Scores. (**C**) Stromal scores. (**D**) Tumor Purity. (*P < 0.05, **P <0.01, ***P < 0.001).

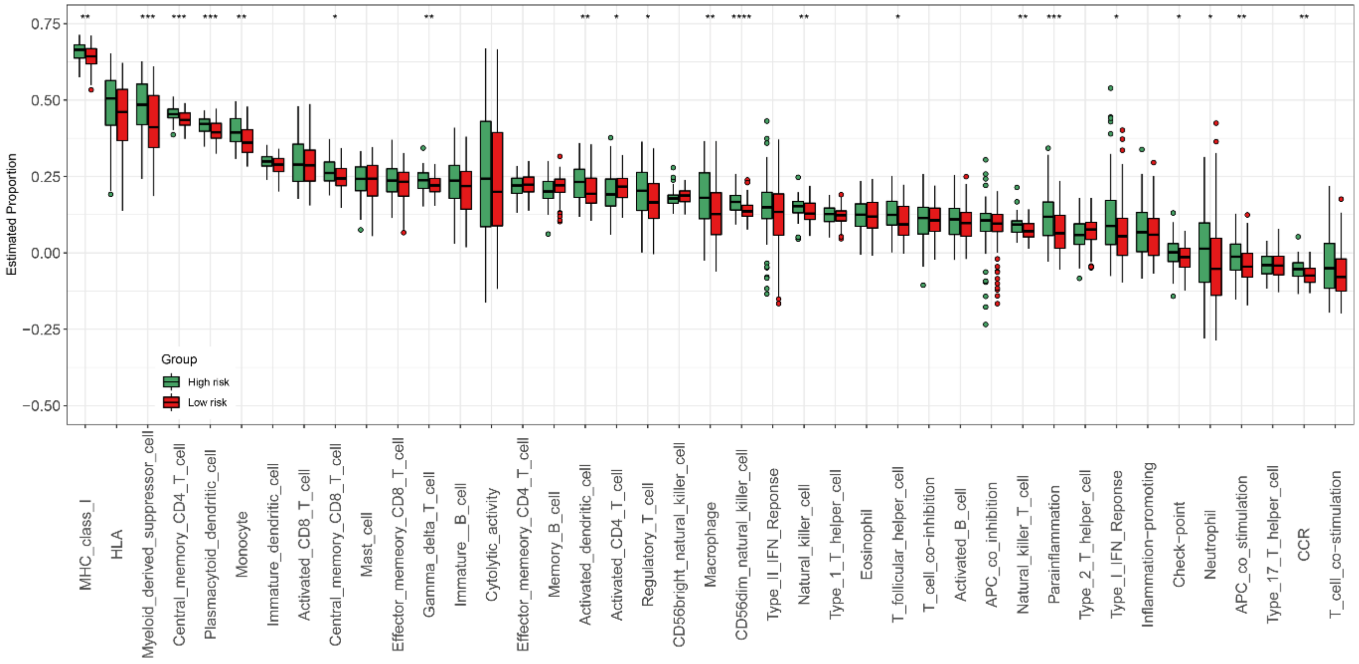


**Supplementary Figure 4. Different cell abundance of forty-one immune categories between HRPS-group/high-risk group and LRPS-group/low-risk group.** (*P < 0.05, **P <0.01, ***P < 0.001).

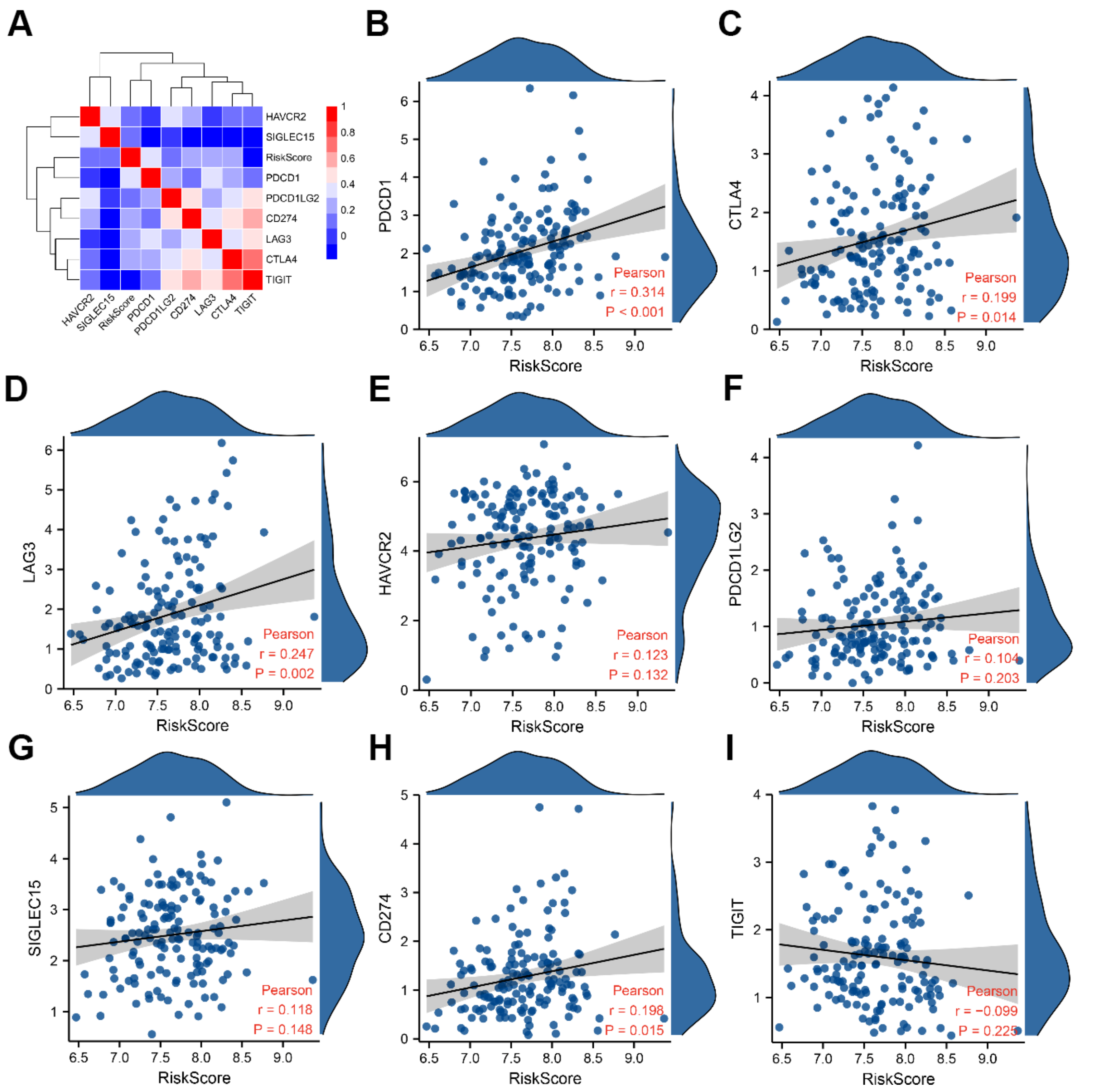


**Supplementary Figure 5. Relationship between PRS and 8 checkpoints' expression.** (**A**) The heatmap of correlation between PRS score and 8 immune checkpoint expression levels. (**B**–**I**) Scatter plot of correlation between PRS score and expression level of each immune checkpoint.

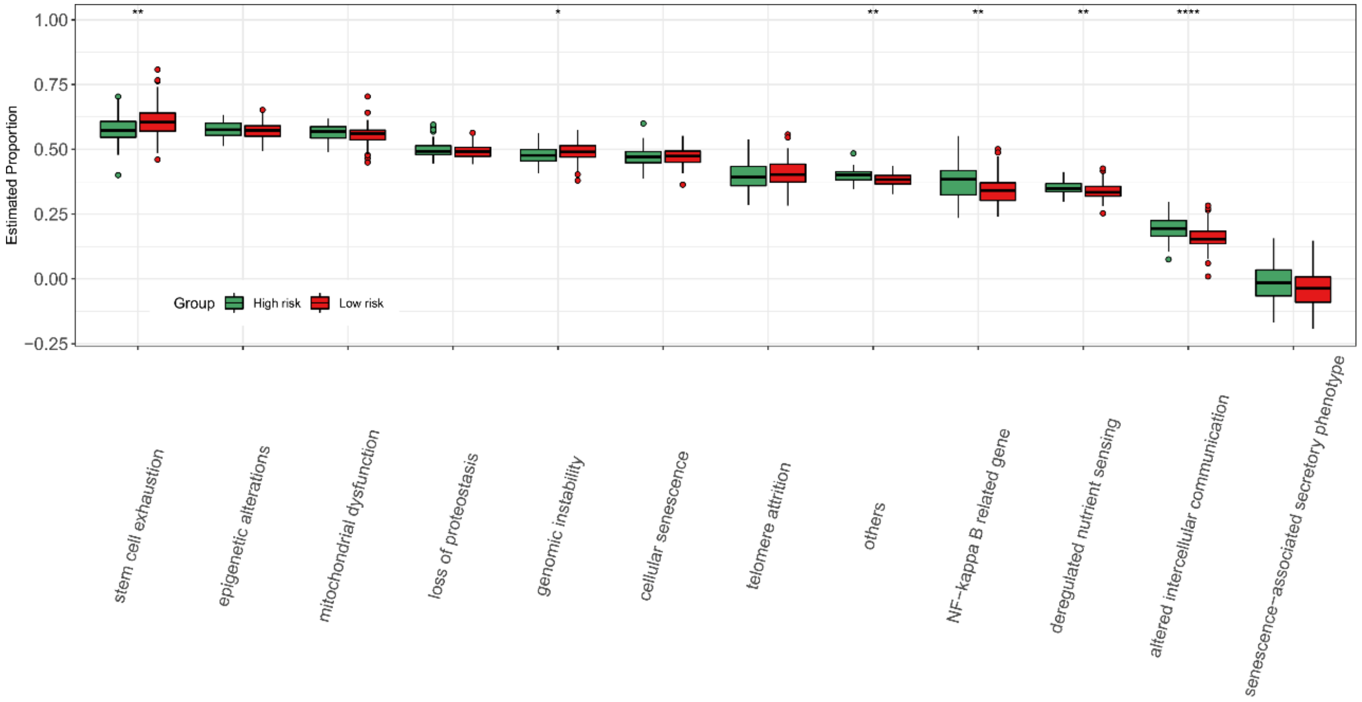


**Supplementary Figure 6. Difference of ssGSEA scores about 12 aging-related gene signals between two different PRS groups according to ARGs prognostic signature.** (*P < 0.05, **P <0.01, ***P < 0.001).

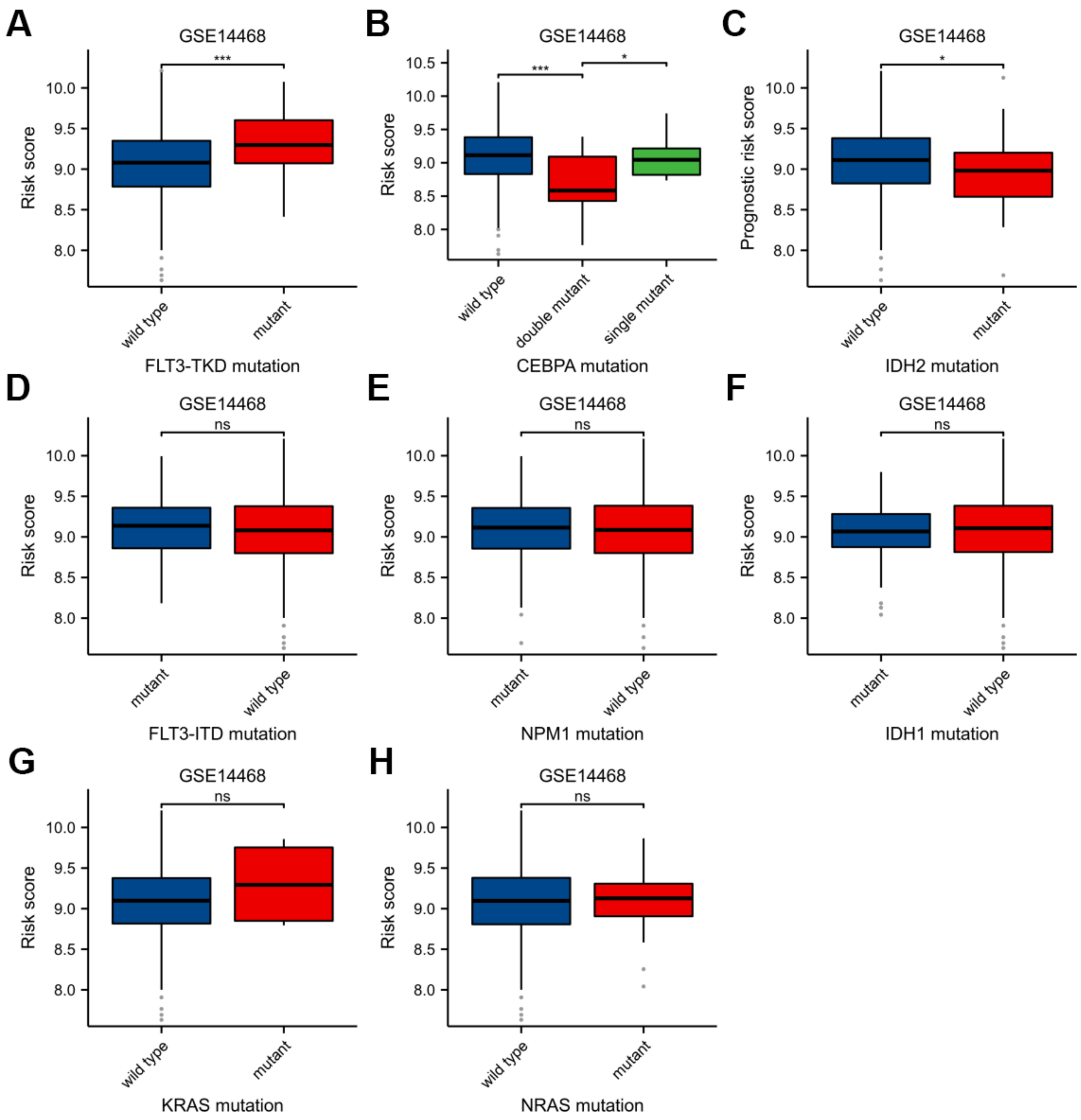


**Supplementary Figure 7. Differences of PRS between wild-type and mutation of several common genes.** (**A**) *FLT3-TKD,* (**B**) *CEBPA*, (**C**) *IDH2*, (**D**) *FLT3-ITD*, (**E**) *NPM*1, (**F**) *IDH1*, (**G**) *KRAS*, (**H**) *NRAS*. (*P < 0.05, **P <0.01, ***P < 0.001).
