# Supplementary material for: Identification and validation of a 7-genes prognostic signature for adult acute myeloid leukemia based on aging-related genes
Source: Aging (Albany NY). 2023 Jun 26;15(12):5826–53. doi: 10.18632/aging.204843 (PMC10333094; doi:10.18632/aging.204843)
Supplement: Supplementary Table 1 [file aging-15-204843-s002.docx]

**Supplementary Table 1. Three hundred seven human genes related to aging.**

| **Gene ID** | **symbol** | **name** | **entrez gene id** |
| --- | --- | --- | --- |
| 1 | GHR | growth hormone receptor | 2690 |
| 2 | GHRH | growth hormone releasing hormone | 2691 |
| 3 | SHC1 | SHC (Src homology 2 domain containing) transforming protein 1 | 6464 |
| 4 | POU1F1 | POU class 1 homeobox 1 | 5449 |
| 5 | PROP1 | PROP paired-like homeobox 1 | 5626 |
| 6 | TP53 | tumor protein p53 | 7157 |
| 7 | TERC | telomerase RNA component | 7012 |
| 8 | TERT | telomerase reverse transcriptase | 7015 |
| 9 | ATM | ATM serine/threonine kinase | 472 |
| 10 | PLAU | plasminogen activator, urokinase | 5328 |
| 11 | ERCC2 | excision repair cross-complementation group 2 | 2068 |
| 12 | ERCC8 | excision repair cross-complementation group 8 | 1161 |
| 13 | WRN | Werner syndrome, RecQ helicase-like | 7486 |
| 14 | LMNA | lamin A/C | 4000 |
| 15 | IGF1R | insulin-like growth factor 1 receptor | 3480 |
| 16 | TXN | thioredoxin | 7295 |
| 17 | KL | klotho | 9365 |
| 18 | E2F1 | E2F transcription factor 1 | 1869 |
| 19 | PTPN11 | protein tyrosine phosphatase, non-receptor type 11 | 5781 |
| 20 | NFKB2 | nuclear factor of kappa light polypeptide gene enhancer in B-cells 2 (p49/p100) | 4791 |
| 21 | STAT5B | signal transducer and activator of transcription 5B | 6777 |
| 22 | STAT3 | signal transducer and activator of transcription 3 (acute-phase response factor) | 6774 |
| 23 | STAT5A | signal transducer and activator of transcription 5A | 6776 |
| 24 | NRG1 | neuregulin 1 | 3084 |
| 25 | HDAC3 | histone deacetylase 3 | 8841 |
| 26 | GH1 | growth hormone 1 | 2688 |
| 27 | IL7R | interleukin 7 receptor | 3575 |
| 28 | IGF1 | insulin-like growth factor 1 (somatomedin C) | 3479 |
| 29 | IGF2 | insulin-like growth factor 2 | 3481 |
| 30 | INS | insulin | 3630 |
| 31 | NGF | nerve growth factor (beta polypeptide) | 4803 |
| 32 | IRS1 | insulin receptor substrate 1 | 3667 |
| 33 | PTPN1 | protein tyrosine phosphatase, non-receptor type 1 | 5770 |
| 34 | IRS2 | insulin receptor substrate 2 | 8660 |
| 35 | AKT1 | v-akt murine thymoma viral oncogene homolog 1 | 207 |
| 36 | PIK3CB | phosphatidylinositol-4,5-bisphosphate 3-kinase, catalytic subunit beta | 5291 |
| 37 | NGFR | nerve growth factor receptor | 4804 |
| 38 | HRAS | Harvey rat sarcoma viral oncogene homolog | 3265 |
| 39 | MYC | v-myc avian myelocytomatosis viral oncogene homolog | 4609 |
| 40 | EGFR | epidermal growth factor receptor | 1956 |
| 41 | ERBB2 | erb-b2 receptor tyrosine kinase 2 | 2064 |
| 42 | INSR | insulin receptor | 3643 |
| 43 | NCOR1 | nuclear receptor corepressor 1 | 9611 |
| 44 | NBN | nibrin | 4683 |
| 45 | JUND | jun D proto-oncogene | 3727 |
| 46 | IL2 | interleukin 2 | 3558 |
| 47 | PDGFB | platelet-derived growth factor beta polypeptide | 5155 |
| 48 | EGF | epidermal growth factor | 1950 |
| 49 | IL2RG | interleukin 2 receptor, gamma | 3561 |
| 50 | FOS | FBJ murine osteosarcoma viral oncogene homolog | 2353 |
| 51 | PDGFRB | platelet-derived growth factor receptor, beta polypeptide | 5159 |
| 52 | EPOR | Erythropoietin receptor | 2057 |
| 53 | SST | somatostatin | 6750 |
| 54 | PRKCD | protein kinase C, delta | 5580 |
| 55 | PPARA | peroxisome proliferator-activated receptor alpha | 5465 |
| 56 | RET | ret proto-oncogene | 5979 |
| 57 | PLCG2 | phospholipase C, gamma 2 (phosphatidylinositol-specific) | 5336 |
| 58 | PEX5 | peroxisomal biogenesis factor 5 | 5830 |
| 59 | TCF3 | transcription factor 3 | 6929 |
| 60 | PARP1 | poly (ADP-ribose) polymerase 1 | 142 |
| 61 | BRCA1 | breast cancer 1, early onset | 672 |
| 62 | PIN1 | peptidylprolyl cis/trans isomerase, NIMA-interacting 1 | 5300 |
| 63 | PTEN | phosphatase and tensin homolog | 5728 |
| 64 | CREBBP | CREB binding protein | 1387 |
| 65 | HIF1A | hypoxia inducible factor 1, alpha subunit (basic helix-loop-helix transcription factor) | 3091 |
| 66 | UBB | ubiquitin B | 7314 |
| 67 | RPA1 | replication protein A1, 70kDa | 6117 |
| 68 | BLM | Bloom syndrome, RecQ helicase-like | 641 |
| 69 | BCL2 | B-cell CLL/lymphoma 2 | 596 |
| 70 | S100B | S100 calcium binding protein B | 6285 |
| 71 | VCP | valosin containing protein | 7415 |
| 72 | POLG | polymerase (DNA directed), gamma | 5428 |
| 73 | IGFBP3 | insulin-like growth factor binding protein 3 | 3486 |
| 74 | HSP90AA1 | heat shock protein 90kDa alpha (cytosolic), class A member 1 | 3320 |
| 75 | NR3C1 | nuclear receptor subfamily 3, group C, member 1 (glucocorticoid receptor) | 2908 |
| 76 | EGR1 | early growth response 1 | 1958 |
| 77 | VEGFA | vascular endothelial growth factor A | 7422 |
| 78 | ABL1 | ABL proto-oncogene 1, non-receptor tyrosine kinase | 25 |
| 79 | BRCA2 | breast cancer 2, early onset | 675 |
| 80 | TOP2A | topoisomerase (DNA) II alpha | 7153 |
| 81 | TOP2B | topoisomerase (DNA) II beta | 7155 |
| 82 | NFKB1 | nuclear factor of kappa light polypeptide gene enhancer in B-cells 1 | 4790 |
| 83 | TOP1 | topoisomerase (DNA) I | 7150 |
| 84 | RAD51 | RAD51 recombinase | 5888 |
| 85 | UBE2I | ubiquitin-conjugating enzyme E2I | 7329 |
| 86 | TNF | tumor necrosis factor | 7124 |
| 87 | PDPK1 | 3-phosphoinositide dependent protein kinase 1 | 5170 |
| 88 | CEBPA | CCAAT/enhancer binding protein (C/EBP), alpha | 1050 |
| 89 | CEBPB | CCAAT/enhancer binding protein (C/EBP), beta | 1051 |
| 90 | MXI1 | MAX interactor 1, dimerization protein | 4601 |
| 91 | TGFB1 | transforming growth factor, beta 1 | 7040 |
| 92 | ERCC6 | excision repair cross-complementation group 6 | 2074 |
| 93 | STK11 | serine/threonine kinase 11 | 6794 |
| 94 | EP300 | E1A binding protein p300 | 2033 |
| 95 | APTX | aprataxin | 54840 |
| 96 | PML | promyelocytic leukemia | 5371 |
| 97 | GSK3B | glycogen synthase kinase 3 beta | 2932 |
| 98 | HTT | huntingtin | 3064 |
| 99 | PRKCA | protein kinase C, alpha | 5578 |
| 100 | SSTR3 | somatostatin receptor 3 | 6753 |
| 101 | HELLS | helicase, lymphoid-specific | 3070 |
| 102 | APOC3 | apolipoprotein C-III | 345 |
| 103 | EEF2 | eukaryotic translation elongation factor 2 | 1938 |
| 104 | ERCC3 | excision repair cross-complementation group 3 | 2071 |
| 105 | TERF1 | telomeric repeat binding factor (NIMA-interacting) 1 | 7013 |
| 106 | PRKDC | protein kinase, DNA-activated, catalytic polypeptide | 5591 |
| 107 | CAT | catalase | 847 |
| 108 | ERCC5 | excision repair cross-complementation group 5 | 2073 |
| 109 | AR | androgen receptor | 367 |
| 110 | GTF2H2 | general transcription factor IIH, polypeptide 2, 44kDa | 2966 |
| 111 | XRCC5 | X-ray repair complementing defective repair in Chinese hamster cells 5 (double-strand-break rejoining) | 7520 |
| 112 | PCNA | proliferating cell nuclear antigen | 5111 |
| 113 | FEN1 | flap structure-specific endonuclease 1 | 2237 |
| 114 | FAS | Fas cell surface death receptor | 355 |
| 115 | TERF2 | telomeric repeat binding factor 2 | 7014 |
| 116 | XRCC6 | X-ray repair complementing defective repair in Chinese hamster cells 6 | 2547 |
| 117 | POLD1 | polymerase (DNA directed), delta 1, catalytic subunit | 5424 |
| 118 | BAX | BCL2-associated X protein | 581 |
| 119 | RB1 | retinoblastoma 1 | 5925 |
| 120 | EMD | emerin | 2010 |
| 121 | GRB2 | growth factor receptor-bound protein 2 | 2885 |
| 122 | FOXO3 | forkhead box O3 | 2309 |
| 123 | FOXO1 | forkhead box O1 | 2308 |
| 124 | HSF1 | heat shock transcription factor 1 | 3297 |
| 125 | XPA | xeroderma pigmentosum, complementation group A | 7507 |
| 126 | MSRA | methionine sulfoxide reductase A | 4482 |
| 127 | RECQL4 | RecQ helicase-like 4 | 9401 |
| 128 | SOD2 | superoxide dismutase 2, mitochondrial | 6648 |
| 129 | SOD1 | superoxide dismutase 1, soluble | 6647 |
| 130 | FOXM1 | forkhead box M1 | 2305 |
| 131 | COQ7 | coenzyme Q7 homolog, ubiquinone (yeast) | 10229 |
| 132 | CACNA1A | calcium channel, voltage-dependent, P/Q type, alpha 1A subunit | 773 |
| 133 | LRP2 | low density lipoprotein receptor-related protein 2 | 4036 |
| 134 | AIFM1 | apoptosis-inducing factor, mitochondrion-associated, 1 | 9131 |
| 135 | UCHL1 | ubiquitin carboxyl-terminal esterase L1 (ubiquitin thiolesterase) | 7345 |
| 136 | APP | amyloid beta (A4) precursor protein | 351 |
| 137 | APOE | apolipoprotein E | 348 |
| 138 | A2M | alpha-2-macroglobulin | 2 |
| 139 | SNCG | synuclein, gamma (breast cancer-specific protein 1) | 6623 |
| 140 | PRDX1 | peroxiredoxin 1 | 5052 |
| 141 | PON1 | paraoxonase 1 | 5444 |
| 142 | RELA | v-rel avian reticuloendotheliosis viral oncogene homolog A | 5970 |
| 143 | IL6 | interleukin 6 | 3569 |
| 144 | RGN | regucalcin | 9104 |
| 145 | ATP5O | ATP synthase, H+ transporting, mitochondrial F1 complex, O subunit | 539 |
| 146 | RAD52 | RAD52 homolog, DNA repair protein | 5893 |
| 147 | TOP3B | topoisomerase (DNA) III beta | 8940 |
| 148 | ERCC1 | excision repair cross-complementation group 1 | 2067 |
| 149 | SIRT1 | sirtuin 1 | 23411 |
| 150 | HDAC1 | histone deacetylase 1 | 3065 |
| 151 | HSPA9 | heat shock 70kDa protein 9 (mortalin) | 3313 |
| 152 | GPX1 | glutathione peroxidase 1 | 2876 |
| 153 | GSR | glutathione reductase | 2936 |
| 154 | GSS | glutathione synthetase | 2937 |
| 155 | GSTA4 | glutathione S-transferase alpha 4 | 2941 |
| 156 | GSTP1 | glutathione S-transferase pi 1 | 2950 |
| 157 | MT-CO1 | mitochondrially encoded cytochrome c oxidase I | 4512 |
| 158 | HSPD1 | heat shock 60kDa protein 1 (chaperonin) | 3329 |
| 159 | HSPA1A | heat shock 70kDa protein 1A | 3303 |
| 160 | HSPA1B | heat shock 70kDa protein 1B | 3304 |
| 161 | PCMT1 | protein-L-isoaspartate (D-aspartate) O-methyltransferase | 5110 |
| 162 | MAPK8 | mitogen-activated protein kinase 8 | 5599 |
| 163 | YWHAZ | tyrosine 3-monooxygenase/tryptophan 5-monooxygenase activation protein, zeta | 7534 |
| 164 | PTK2B | protein tyrosine kinase 2 beta | 2185 |
| 165 | PTK2 | protein tyrosine kinase 2 | 5747 |
| 166 | IL7 | interleukin 7 | 3574 |
| 167 | MAPK14 | mitogen-activated protein kinase 14 | 1432 |
| 168 | FGFR1 | fibroblast growth factor receptor 1 | 2260 |
| 169 | SP1 | Sp1 transcription factor | 6667 |
| 170 | FLT1 | fms-related tyrosine kinase 1 | 2321 |
| 171 | JUN | jun proto-oncogene | 3725 |
| 172 | MED1 | mediator complex subunit 1 | 5469 |
| 173 | MAPK9 | mitogen-activated protein kinase 9 | 5601 |
| 174 | MAPK3 | mitogen-activated protein kinase 3 | 5595 |
| 175 | HMGB1 | high mobility group box 1 | 3146 |
| 176 | CCNA2 | cyclin A2 | 890 |
| 177 | HMGB2 | high mobility group box 2 | 3148 |
| 178 | MAP3K5 | mitogen-activated protein kinase kinase kinase 5 | 4217 |
| 179 | TAF1 | TAF1 RNA polymerase II, TATA box binding protein (TBP)-associated factor, 250kDa | 6872 |
| 180 | LMNB1 | lamin B1 | 4001 |
| 181 | SDHC | succinate dehydrogenase complex, subunit C, integral membrane protein, 15kDa | 6391 |
| 182 | FOXO4 | forkhead box O4 | 4303 |
| 183 | HESX1 | HESX homeobox 1 | 8820 |
| 184 | PIK3R1 | phosphoinositide-3-kinase, regulatory subunit 1 (alpha) | 5295 |
| 185 | BSCL2 | Berardinelli-Seip congenital lipodystrophy 2 (seipin) | 26580 |
| 186 | AGPAT2 | 1-acylglycerol-3-phosphate O-acyltransferase 2 | 10555 |
| 187 | BMI1 | BMI1 proto-oncogene, polycomb ring finger | 648 |
| 188 | EEF1A1 | eukaryotic translation elongation factor 1 alpha 1 | 1915 |
| 189 | TFAP2A | transcription factor AP-2 alpha (activating enhancer binding protein 2 alpha) | 7020 |
| 190 | BDNF | brain-derived neurotrophic factor | 627 |
| 191 | CREB1 | cAMP responsive element binding protein 1 | 1385 |
| 192 | ATF2 | activating transcription factor 2 | 1386 |
| 193 | TBP | TATA box binding protein | 6908 |
| 194 | APEX1 | APEX nuclease (multifunctional DNA repair enzyme) 1 | 328 |
| 195 | HBP1 | HMG-box transcription factor 1 | 26959 |
| 196 | BUB1B | BUB1 mitotic checkpoint serine/threonine kinase B | 701 |
| 197 | PTGS2 | prostaglandin-endoperoxide synthase 2 (prostaglandin G/H synthase and cyclooxygenase) | 5743 |
| 198 | HSPA8 | heat shock 70kDa protein 8 | 3312 |
| 199 | SIN3A | SIN3 transcription regulator family member A | 25942 |
| 200 | CDK1 | cyclin-dependent kinase 1 | 983 |
| 201 | TFDP1 | transcription factor Dp-1 | 7027 |
| 202 | DDIT3 | DNA-damage-inducible transcript 3 | 1649 |
| 203 | POLA1 | polymerase (DNA directed), alpha 1, catalytic subunit | 5422 |
| 204 | MAPT | microtubule-associated protein tau | 4137 |
| 205 | CTGF | connective tissue growth factor | 1490 |
| 206 | HDAC2 | histone deacetylase 2 | 3066 |
| 207 | MAX | MYC associated factor X | 4149 |
| 208 | MXD1 | MAX dimerization protein 1 | 4084 |
| 209 | MDM2 | MDM2 proto-oncogene, E3 ubiquitin protein ligase | 4193 |
| 210 | SUMO1 | small ubiquitin-like modifier 1 | 7341 |
| 211 | H2AFX | H2A histone family, member X | 3014 |
| 212 | HOXB7 | homeobox B7 | 3217 |
| 213 | HOXC4 | homeobox C4 | 3221 |
| 214 | JAK2 | Janus kinase 2 | 3717 |
| 215 | ESR1 | estrogen receptor 1 | 2099 |
| 216 | LEP | leptin | 3952 |
| 217 | LEPR | leptin receptor | 3953 |
| 218 | NFKBIA | nuclear factor of kappa light polypeptide gene enhancer in B-cells inhibitor, alpha | 4792 |
| 219 | CLU | clusterin | 1191 |
| 220 | MTOR | mechanistic target of rapamycin (serine/threonine kinase) | 2475 |
| 221 | GHRHR | growth hormone releasing hormone receptor | 2692 |
| 222 | CTNNB1 | catenin (cadherin-associated protein), beta 1, 88kDa | 1499 |
| 223 | PSEN1 | presenilin 1 | 5663 |
| 224 | DLL3 | delta-like 3 (Drosophila) | 10683 |
| 225 | CDKN2A | cyclin-dependent kinase inhibitor 2A | 1029 |
| 226 | PPP1CA | protein phosphatase 1, catalytic subunit, alpha isozyme | 5499 |
| 227 | DBN1 | drebrin 1 | 1627 |
| 228 | NOG | noggin | 9241 |
| 229 | ELN | elastin | 2006 |
| 230 | ATR | ATR serine/threonine kinase | 545 |
| 231 | UCP3 | uncoupling protein 3 (mitochondrial, proton carrier) | 7352 |
| 232 | ZMPSTE24 | zinc metallopeptidase STE24 | 10269 |
| 233 | TP63 | tumor protein p63 | 8626 |
| 234 | UCP2 | uncoupling protein 2 (mitochondrial, proton carrier) | 7351 |
| 235 | POLB | polymerase (DNA directed), beta | 5423 |
| 236 | GCLC | glutamate-cysteine ligase, catalytic subunit | 2729 |
| 237 | GCLM | glutamate-cysteine ligase, modifier subunit | 2730 |
| 238 | SIRT6 | sirtuin 6 | 51548 |
| 239 | BUB3 | BUB3 mitotic checkpoint protein | 9184 |
| 240 | RAE1 | ribonucleic acid export 1 | 8480 |
| 241 | PMCH | pro-melanin-concentrating hormone | 5367 |
| 242 | MLH1 | mutL homolog 1 | 4292 |
| 243 | CSNK1E | casein kinase 1, epsilon | 1454 |
| 244 | STUB1 | STIP1 homology and U-box containing protein 1, E3 ubiquitin protein ligase | 10273 |
| 245 | PPM1D | protein phosphatase, Mg2+/Mn2+ dependent, 1D | 8493 |
| 246 | CHEK2 | checkpoint kinase 2 | 11200 |
| 247 | PCK1 | phosphoenolpyruvate carboxykinase 1 (soluble) | 5105 |
| 248 | ARHGAP1 | Rho GTPase activating protein 1 | 392 |
| 249 | CDC42 | cell division cycle 42 | 998 |
| 250 | ARNTL | aryl hydrocarbon receptor nuclear translocator-like | 406 |
| 251 | CLOCK | clock circadian regulator | 9575 |
| 252 | HIC1 | hypermethylated in cancer 1 | 3090 |
| 253 | PAPPA | pregnancy-associated plasma protein A, pappalysin 1 | 5069 |
| 254 | ADCY5 | adenylate cyclase 5 | 111 |
| 255 | PPARGC1A | peroxisome proliferator-activated receptor gamma, coactivator 1 alpha | 10891 |
| 256 | GPX4 | glutathione peroxidase 4 | 2879 |
| 257 | UCP1 | uncoupling protein 1 (mitochondrial, proton carrier) | 7350 |
| 258 | FGF23 | fibroblast growth factor 23 | 8074 |
| 259 | EFEMP1 | EGF containing fibulin-like extracellular matrix protein 1 | 2202 |
| 260 | ERCC4 | excision repair cross-complementation group 4 | 2072 |
| 261 | CETP | cholesteryl ester transfer protein, plasma | 1071 |
| 262 | PPARG | peroxisome proliferator-activated receptor gamma | 5468 |
| 263 | AGTR1 | angiotensin II receptor, type 1 | 185 |
| 264 | CISD2 | CDGSH iron sulfur domain 2 | 493856 |
| 265 | EEF1E1 | eukaryotic translation elongation factor 1 epsilon 1 | 9521 |
| 266 | EPS8 | epidermal growth factor receptor pathway substrate 8 | 2059 |
| 267 | KCNA3 | potassium channel, voltage gated shaker related subfamily A, member 3 | 3738 |
| 268 | SIRT7 | sirtuin 7 | 51547 |
| 269 | SLC13A1 | solute carrier family 13 (sodium/sulfate symporter), member 1 | 6561 |
| 270 | SOCS2 | suppressor of cytokine signaling 2 | 8835 |
| 271 | TPP2 | tripeptidyl peptidase II | 7174 |
| 272 | TP53BP1 | tumor protein p53 binding protein 1 | 7158 |
| 273 | SIRT3 | sirtuin 3 | 23410 |
| 274 | NCOR2 | nuclear receptor corepressor 2 | 9612 |
| 275 | SUN1 | Sad1 and UNC84 domain containing 1 | 23353 |
| 276 | BAK1 | BCL2-antagonist/killer 1 | 578 |
| 277 | IGFBP2 | insulin-like growth factor binding protein 2, 36kDa | 3485 |
| 278 | PYCR1 | pyrroline-5-carboxylate reductase 1 | 5831 |
| 279 | TP73 | tumor protein p73 | 7161 |
| 280 | CNR1 | cannabinoid receptor 1 (brain) | 1268 |
| 281 | NFE2L2 | nuclear factor, erythroid 2-like 2 | 4780 |
| 282 | CDKN1A | cyclin-dependent kinase inhibitor 1A (p21, Cip1) | 1026 |
| 283 | PDGFRA | platelet-derived growth factor receptor, alpha polypeptide | 5156 |
| 284 | PIK3CA | phosphatidylinositol-4,5-bisphosphate 3-kinase, catalytic subunit alpha | 5290 |
| 285 | C1QA | complement component 1, q subcomponent, A chain | 712 |
| 286 | CDKN2B | cyclin-dependent kinase inhibitor 2B (p15, inhibits CDK4) | 1030 |
| 287 | EIF5A2 | eukaryotic translation initiation factor 5A2 | 56648 |
| 288 | MIF | macrophage migration inhibitory factor (glycosylation-inhibiting factor) | 4282 |
| 289 | DGAT1 | diacylglycerol O-acyltransferase 1 | 8694 |
| 290 | MT1E | metallothionein 1E | 4493 |
| 291 | FGF21 | fibroblast growth factor 21 | 26291 |
| 292 | HTRA2 | HtrA serine peptidase 2 | 27429 |
| 293 | GSK3A | glycogen synthase kinase 3 alpha | 2931 |
| 294 | NUDT1 | nudix (nucleoside diphosphate linked moiety X)-type motif 1 | 4521 |
| 295 | IKBKB | inhibitor of kappa light polypeptide gene enhancer in B-cells, kinase beta | 3551 |
| 296 | SQSTM1 | sequestosome 1 | 8878 |
| 297 | CDK7 | cyclin-dependent kinase 7 | 1022 |
| 298 | GRN | granulin | 2896 |
| 299 | SERPINE1 | serpin peptidase inhibitor, clade E (nexin, plasminogen activator inhibitor type 1), member 1 | 5054 |
| 300 | SPRTN | SprT-like N-terminal domain | 83932 |
| 301 | RICTOR | RPTOR independent companion of MTOR, complex 2 | 253260 |
| 302 | CTF1 | cardiotrophin 1 | 1489 |
| 303 | TRAP1 | TNF receptor-associated protein 1 | 10131 |
| 304 | TRPV1 | transient receptor potential cation channel subfamily V member 1 | 7442 |
| 305 | NFE2L1 | nuclear factor, erythroid 2-like 1 | 4779 |
| 306 | IFNB1 | Interferon beta | 3456 |
| 307 | GDF11 | growth differentiation factor 11 | 10220 |
