# Supplementary material for: Identification and validation of a 7-genes prognostic signature for adult acute myeloid leukemia based on aging-related genes
Source: Aging (Albany NY). 2023 Jun 26;15(12):5826–53. doi: 10.18632/aging.204843 (PMC10333094; doi:10.18632/aging.204843)
Supplement: Supplementary Table 2 [file aging-15-204843-s003.docx]

| **Supplementary Table 2. Univariate analyses of 303 ARGs for overall survival.** | | | | | | |
| --- | --- | --- | --- | --- | --- | --- |
| **Gene name** | **Coef** | **Se** | **P** | **HR** | **95%CI low** | **95%CI High** |
| LMNA | -0.845 | 0.223 | <0.001 | 0.429 | 0.277 | 0.665 |
| HSPA1B | -0.844 | 0.224 | <0.001 | 0.430 | 0.277 | 0.666 |
| PTPN1 | -0.780 | 0.221 | <0.001 | 0.458 | 0.297 | 0.706 |
| UCP2 | -0.748 | 0.220 | 0.001 | 0.473 | 0.308 | 0.728 |
| GPX1 | -0.739 | 0.220 | 0.001 | 0.478 | 0.310 | 0.735 |
| SIRT6 | -0.721 | 0.218 | 0.001 | 0.486 | 0.317 | 0.746 |
| BAK1 | -0.708 | 0.218 | 0.001 | 0.493 | 0.321 | 0.756 |
| TERF2 | -0.694 | 0.218 | 0.001 | 0.500 | 0.326 | 0.766 |
| TGFB1 | -0.673 | 0.220 | 0.002 | 0.510 | 0.331 | 0.785 |
| NUDT1 | -0.656 | 0.218 | 0.003 | 0.519 | 0.339 | 0.795 |
| ELN | -0.631 | 0.220 | 0.004 | 0.532 | 0.346 | 0.819 |
| TRAP1 | -0.606 | 0.217 | 0.005 | 0.545 | 0.356 | 0.835 |
| PPP1CA | -0.606 | 0.217 | 0.005 | 0.546 | 0.357 | 0.834 |
| ERCC1 | -0.599 | 0.218 | 0.006 | 0.549 | 0.358 | 0.843 |
| HDAC3 | -0.581 | 0.218 | 0.008 | 0.559 | 0.365 | 0.857 |
| GPX4 | -0.578 | 0.219 | 0.008 | 0.561 | 0.365 | 0.862 |
| GSTP1 | -0.571 | 0.218 | 0.009 | 0.565 | 0.369 | 0.865 |
| STK11 | -0.551 | 0.219 | 0.012 | 0.576 | 0.375 | 0.886 |
| GSK3A | -0.551 | 0.217 | 0.011 | 0.576 | 0.377 | 0.881 |
| SOCS2 | -0.530 | 0.216 | 0.014 | 0.589 | 0.385 | 0.900 |
| SOD1 | -0.521 | 0.217 | 0.016 | 0.594 | 0.388 | 0.908 |
| NFKB2 | -0.510 | 0.217 | 0.019 | 0.600 | 0.393 | 0.918 |
| HSPA1A | -0.506 | 0.218 | 0.020 | 0.603 | 0.393 | 0.924 |
| EMD | -0.498 | 0.216 | 0.021 | 0.608 | 0.398 | 0.928 |
| PRKCD | -0.492 | 0.218 | 0.024 | 0.611 | 0.399 | 0.936 |
| ERCC2 | -0.483 | 0.217 | 0.026 | 0.617 | 0.403 | 0.944 |
| PRDX1 | -0.482 | 0.216 | 0.026 | 0.618 | 0.404 | 0.944 |
| DGAT1 | -0.481 | 0.217 | 0.026 | 0.618 | 0.404 | 0.945 |
| UCP3 | -0.470 | 0.216 | 0.030 | 0.625 | 0.410 | 0.955 |
| SPRTN | -0.460 | 0.219 | 0.036 | 0.631 | 0.411 | 0.971 |
| PTK2B | -0.461 | 0.217 | 0.034 | 0.631 | 0.412 | 0.966 |
| MAP3K5 | -0.458 | 0.218 | 0.035 | 0.633 | 0.413 | 0.969 |
| MT1E | -0.443 | 0.219 | 0.043 | 0.642 | 0.418 | 0.987 |
| GCLM | -0.437 | 0.217 | 0.043 | 0.646 | 0.422 | 0.987 |
| BMI1 | -0.435 | 0.217 | 0.044 | 0.647 | 0.423 | 0.989 |
| IL7 | -0.417 | 0.216 | 0.053 | 0.659 | 0.432 | 1.006 |
| HSF1 | -0.411 | 0.215 | 0.057 | 0.663 | 0.435 | 1.012 |
| ARNTL | -0.410 | 0.217 | 0.059 | 0.664 | 0.434 | 1.016 |
| HDAC1 | -0.407 | 0.216 | 0.059 | 0.665 | 0.436 | 1.016 |
| CEBPB | -0.406 | 0.217 | 0.061 | 0.666 | 0.435 | 1.019 |
| PTK2 | -0.403 | 0.217 | 0.063 | 0.668 | 0.437 | 1.023 |
| PCNA | -0.398 | 0.215 | 0.064 | 0.671 | 0.440 | 1.024 |
| XRCC6 | -0.398 | 0.216 | 0.065 | 0.671 | 0.440 | 1.025 |
| ZMPSTE24 | -0.396 | 0.217 | 0.068 | 0.673 | 0.440 | 1.030 |
| KCNA3 | -0.393 | 0.216 | 0.069 | 0.675 | 0.442 | 1.031 |
| IGF1 | -0.390 | 0.218 | 0.073 | 0.677 | 0.442 | 1.037 |
| FEN1 | -0.384 | 0.216 | 0.075 | 0.681 | 0.446 | 1.039 |
| GH1 | -0.380 | 0.218 | 0.081 | 0.684 | 0.446 | 1.048 |
| PARP1 | -0.376 | 0.216 | 0.082 | 0.687 | 0.450 | 1.048 |
| FGF21 | -0.372 | 0.216 | 0.085 | 0.689 | 0.451 | 1.053 |
| GSR | -0.366 | 0.217 | 0.091 | 0.693 | 0.453 | 1.061 |
| STUB1 | -0.365 | 0.217 | 0.093 | 0.695 | 0.454 | 1.062 |
| HOXC4 | -0.362 | 0.218 | 0.097 | 0.696 | 0.454 | 1.068 |
| GSS | -0.363 | 0.216 | 0.093 | 0.696 | 0.456 | 1.063 |
| PON1 | -0.353 | 0.219 | 0.107 | 0.703 | 0.458 | 1.079 |
| MTOR | -0.350 | 0.215 | 0.104 | 0.705 | 0.462 | 1.074 |
| YWHAZ | -0.349 | 0.217 | 0.107 | 0.706 | 0.462 | 1.079 |
| MED1 | -0.341 | 0.216 | 0.113 | 0.711 | 0.466 | 1.084 |
| HOXB7 | -0.340 | 0.217 | 0.117 | 0.712 | 0.465 | 1.089 |
| AIFM1 | -0.339 | 0.216 | 0.117 | 0.713 | 0.467 | 1.088 |
| CDC42 | -0.331 | 0.216 | 0.124 | 0.718 | 0.471 | 1.095 |
| EPOR | -0.327 | 0.216 | 0.130 | 0.721 | 0.472 | 1.101 |
| SHC1 | -0.324 | 0.218 | 0.136 | 0.723 | 0.472 | 1.108 |
| HSPA8 | -0.321 | 0.216 | 0.136 | 0.725 | 0.475 | 1.107 |
| RB1 | -0.314 | 0.216 | 0.146 | 0.730 | 0.478 | 1.116 |
| PIN1 | -0.309 | 0.216 | 0.152 | 0.734 | 0.481 | 1.121 |
| NFE2L1 | -0.307 | 0.216 | 0.154 | 0.736 | 0.482 | 1.122 |
| SOD2 | -0.304 | 0.216 | 0.160 | 0.738 | 0.483 | 1.128 |
| GRB2 | -0.301 | 0.216 | 0.164 | 0.740 | 0.485 | 1.131 |
| CAT | -0.297 | 0.216 | 0.170 | 0.743 | 0.487 | 1.136 |
| HRAS | -0.296 | 0.215 | 0.170 | 0.744 | 0.488 | 1.135 |
| TXN | -0.290 | 0.215 | 0.177 | 0.748 | 0.491 | 1.140 |
| VCP | -0.288 | 0.216 | 0.181 | 0.750 | 0.491 | 1.144 |
| MIF | -0.284 | 0.215 | 0.186 | 0.752 | 0.494 | 1.147 |
| CTNNB1 | -0.284 | 0.216 | 0.188 | 0.753 | 0.493 | 1.149 |
| NFKBIA | -0.282 | 0.215 | 0.189 | 0.754 | 0.495 | 1.149 |
| EEF2 | -0.278 | 0.215 | 0.196 | 0.757 | 0.496 | 1.155 |
| PPARGC1A | -0.276 | 0.216 | 0.203 | 0.759 | 0.497 | 1.160 |
| BAX | -0.275 | 0.217 | 0.205 | 0.759 | 0.496 | 1.163 |
| DBN1 | -0.270 | 0.215 | 0.209 | 0.763 | 0.501 | 1.164 |
| CDKN2B | -0.270 | 0.216 | 0.210 | 0.763 | 0.500 | 1.164 |
| GCLC | -0.267 | 0.215 | 0.214 | 0.765 | 0.502 | 1.167 |
| NCOR2 | -0.266 | 0.216 | 0.217 | 0.766 | 0.502 | 1.169 |
| APTX | -0.264 | 0.215 | 0.220 | 0.768 | 0.504 | 1.171 |
| ATM | -0.262 | 0.216 | 0.227 | 0.770 | 0.504 | 1.176 |
| SP1 | -0.261 | 0.216 | 0.227 | 0.770 | 0.504 | 1.176 |
| GSK3B | -0.258 | 0.216 | 0.231 | 0.772 | 0.506 | 1.178 |
| MAPK3 | -0.256 | 0.216 | 0.235 | 0.774 | 0.507 | 1.181 |
| FOXO1 | -0.254 | 0.218 | 0.244 | 0.776 | 0.506 | 1.189 |
| ESR1 | -0.251 | 0.216 | 0.244 | 0.778 | 0.509 | 1.187 |
| CACNA1A | -0.249 | 0.217 | 0.251 | 0.780 | 0.510 | 1.193 |
| MXI1 | -0.246 | 0.215 | 0.253 | 0.782 | 0.513 | 1.193 |
| HSP90AA1 | -0.243 | 0.215 | 0.258 | 0.784 | 0.515 | 1.195 |
| BUB3 | -0.237 | 0.215 | 0.270 | 0.789 | 0.518 | 1.203 |
| EPS8 | -0.234 | 0.216 | 0.279 | 0.791 | 0.518 | 1.208 |
| COQ7 | -0.235 | 0.215 | 0.275 | 0.791 | 0.519 | 1.206 |
| AGPAT2 | -0.224 | 0.215 | 0.297 | 0.799 | 0.524 | 1.218 |
| ARHGAP1 | -0.224 | 0.215 | 0.298 | 0.799 | 0.524 | 1.218 |
| HTRA2 | -0.223 | 0.215 | 0.300 | 0.800 | 0.525 | 1.220 |
| SIRT7 | -0.224 | 0.216 | 0.300 | 0.800 | 0.524 | 1.220 |
| NGFR | -0.222 | 0.216 | 0.305 | 0.801 | 0.524 | 1.223 |
| JUND | -0.220 | 0.215 | 0.306 | 0.802 | 0.526 | 1.223 |
| RAD51 | -0.219 | 0.215 | 0.310 | 0.804 | 0.527 | 1.226 |
| SUMO1 | -0.215 | 0.216 | 0.318 | 0.806 | 0.529 | 1.230 |
| GTF2H2 | -0.207 | 0.215 | 0.335 | 0.813 | 0.533 | 1.239 |
| POU1F1 | -0.204 | 0.215 | 0.344 | 0.816 | 0.535 | 1.244 |
| RECQL4 | -0.204 | 0.216 | 0.345 | 0.816 | 0.534 | 1.245 |
| EGF | -0.198 | 0.216 | 0.358 | 0.820 | 0.537 | 1.252 |
| RPA1 | -0.198 | 0.215 | 0.358 | 0.820 | 0.538 | 1.252 |
| IKBKB | -0.197 | 0.216 | 0.361 | 0.821 | 0.538 | 1.253 |
| HELLS | -0.195 | 0.216 | 0.365 | 0.822 | 0.539 | 1.255 |
| NR3C1 | -0.193 | 0.216 | 0.370 | 0.824 | 0.540 | 1.258 |
| TP63 | -0.188 | 0.215 | 0.382 | 0.829 | 0.543 | 1.263 |
| CISD2 | -0.186 | 0.215 | 0.388 | 0.830 | 0.544 | 1.267 |
| CEBPA | -0.186 | 0.215 | 0.386 | 0.830 | 0.545 | 1.265 |
| SNCG | -0.184 | 0.216 | 0.393 | 0.832 | 0.545 | 1.270 |
| UBE2I | -0.183 | 0.215 | 0.394 | 0.832 | 0.546 | 1.269 |
| UBB | -0.167 | 0.215 | 0.439 | 0.846 | 0.555 | 1.291 |
| POLG | -0.166 | 0.216 | 0.441 | 0.847 | 0.555 | 1.293 |
| FOXO4 | -0.163 | 0.215 | 0.450 | 0.850 | 0.557 | 1.296 |
| FOXM1 | -0.160 | 0.215 | 0.456 | 0.852 | 0.559 | 1.299 |
| ERCC4 | -0.156 | 0.215 | 0.469 | 0.856 | 0.561 | 1.304 |
| PRKDC | -0.154 | 0.215 | 0.474 | 0.857 | 0.562 | 1.307 |
| C1QA | -0.153 | 0.217 | 0.482 | 0.858 | 0.561 | 1.314 |
| LRP2 | -0.146 | 0.215 | 0.496 | 0.864 | 0.567 | 1.317 |
| RELA | -0.145 | 0.215 | 0.500 | 0.865 | 0.567 | 1.319 |
| PSEN1 | -0.133 | 0.215 | 0.535 | 0.875 | 0.574 | 1.334 |
| TP53 | -0.133 | 0.215 | 0.535 | 0.875 | 0.575 | 1.333 |
| PDPK1 | -0.126 | 0.215 | 0.558 | 0.882 | 0.578 | 1.344 |
| HSPD1 | -0.123 | 0.215 | 0.566 | 0.884 | 0.580 | 1.347 |
| CTF1 | -0.124 | 0.215 | 0.566 | 0.884 | 0.580 | 1.347 |
| ABL1 | -0.120 | 0.215 | 0.576 | 0.887 | 0.582 | 1.351 |
| RGN | -0.119 | 0.215 | 0.580 | 0.888 | 0.583 | 1.353 |
| APOC3 | -0.118 | 0.216 | 0.585 | 0.889 | 0.583 | 1.356 |
| MDM2 | -0.116 | 0.215 | 0.589 | 0.890 | 0.584 | 1.357 |
| PTPN11 | -0.115 | 0.215 | 0.591 | 0.891 | 0.585 | 1.358 |
| AKT1 | -0.115 | 0.216 | 0.594 | 0.891 | 0.584 | 1.361 |
| BLM | -0.113 | 0.215 | 0.598 | 0.893 | 0.586 | 1.360 |
| CDKN1A | -0.109 | 0.215 | 0.611 | 0.896 | 0.589 | 1.365 |
| TFDP1 | -0.109 | 0.215 | 0.611 | 0.896 | 0.588 | 1.366 |
| NFE2L2 | -0.109 | 0.216 | 0.614 | 0.897 | 0.588 | 1.369 |
| HMGB1 | -0.109 | 0.215 | 0.611 | 0.897 | 0.589 | 1.366 |
| TOP1 | -0.104 | 0.215 | 0.627 | 0.901 | 0.591 | 1.372 |
| CDK7 | -0.102 | 0.215 | 0.635 | 0.903 | 0.592 | 1.377 |
| UCHL1 | -0.101 | 0.216 | 0.640 | 0.904 | 0.592 | 1.380 |
| CLU | -0.101 | 0.215 | 0.638 | 0.904 | 0.593 | 1.378 |
| RAD52 | -0.101 | 0.215 | 0.638 | 0.904 | 0.593 | 1.377 |
| SIRT3 | -0.090 | 0.217 | 0.677 | 0.914 | 0.598 | 1.397 |
| S100B | -0.089 | 0.215 | 0.680 | 0.915 | 0.600 | 1.395 |
| PTGS2 | -0.087 | 0.215 | 0.687 | 0.917 | 0.602 | 1.398 |
| MYC | -0.082 | 0.215 | 0.703 | 0.921 | 0.605 | 1.403 |
| STAT5A | -0.081 | 0.216 | 0.707 | 0.922 | 0.604 | 1.408 |
| APEX1 | -0.080 | 0.216 | 0.709 | 0.923 | 0.605 | 1.408 |
| PML | -0.078 | 0.215 | 0.717 | 0.925 | 0.607 | 1.410 |
| NFKB1 | -0.072 | 0.215 | 0.736 | 0.930 | 0.610 | 1.417 |
| PPARA | -0.071 | 0.215 | 0.740 | 0.931 | 0.611 | 1.419 |
| ERBB2 | -0.071 | 0.215 | 0.743 | 0.932 | 0.612 | 1.420 |
| PCMT1 | -0.068 | 0.215 | 0.752 | 0.934 | 0.613 | 1.425 |
| PYCR1 | -0.063 | 0.216 | 0.769 | 0.939 | 0.615 | 1.432 |
| HDAC2 | -0.062 | 0.215 | 0.774 | 0.940 | 0.617 | 1.433 |
| ADCY5 | -0.055 | 0.215 | 0.797 | 0.946 | 0.620 | 1.443 |
| GSTA4 | -0.055 | 0.215 | 0.799 | 0.947 | 0.622 | 1.442 |
| ERCC6 | -0.052 | 0.215 | 0.808 | 0.949 | 0.623 | 1.447 |
| RAE1 | -0.053 | 0.216 | 0.807 | 0.949 | 0.621 | 1.448 |
| PAPPA | -0.050 | 0.216 | 0.817 | 0.951 | 0.623 | 1.452 |
| TRPV1 | -0.050 | 0.215 | 0.816 | 0.951 | 0.624 | 1.449 |
| HESX1 | -0.048 | 0.215 | 0.822 | 0.953 | 0.626 | 1.451 |
| SDHC | -0.045 | 0.215 | 0.835 | 0.956 | 0.628 | 1.457 |
| UCP1 | -0.045 | 0.218 | 0.838 | 0.956 | 0.624 | 1.467 |
| PLAU | -0.044 | 0.216 | 0.838 | 0.957 | 0.627 | 1.461 |
| XPA | -0.043 | 0.215 | 0.843 | 0.958 | 0.628 | 1.462 |
| ERCC5 | -0.043 | 0.216 | 0.843 | 0.958 | 0.627 | 1.463 |
| TERT | -0.035 | 0.215 | 0.872 | 0.966 | 0.634 | 1.472 |
| MT-CO1 | -0.035 | 0.215 | 0.872 | 0.966 | 0.634 | 1.472 |
| PRKCA | -0.031 | 0.216 | 0.884 | 0.969 | 0.635 | 1.479 |
| BDNF | -0.024 | 0.215 | 0.910 | 0.976 | 0.640 | 1.489 |
| HTT | -0.023 | 0.215 | 0.914 | 0.977 | 0.641 | 1.489 |
| HMGB2 | -0.018 | 0.216 | 0.935 | 0.983 | 0.643 | 1.501 |
| POLD1 | -0.014 | 0.216 | 0.947 | 0.986 | 0.646 | 1.505 |
| HIC1 | -0.012 | 0.216 | 0.957 | 0.988 | 0.648 | 1.508 |
| ATF2 | -0.008 | 0.215 | 0.970 | 0.992 | 0.651 | 1.511 |
| TCF3 | -0.004 | 0.215 | 0.986 | 0.996 | 0.654 | 1.519 |
| PDGFB | 0.000 | 0.215 | 0.999 | 1.000 | 0.656 | 1.525 |
| FAS | 0.002 | 0.215 | 0.994 | 1.002 | 0.657 | 1.527 |
| PMCH | 0.005 | 0.215 | 0.983 | 1.005 | 0.659 | 1.531 |
| CCNA2 | 0.006 | 0.215 | 0.976 | 1.006 | 0.661 | 1.533 |
| MAPK9 | 0.006 | 0.215 | 0.979 | 1.006 | 0.660 | 1.532 |
| NGF | 0.007 | 0.260 | 0.980 | 1.007 | 0.605 | 1.676 |
| JAK2 | 0.008 | 0.215 | 0.971 | 1.008 | 0.661 | 1.537 |
| SLC13A1 | 0.013 | 0.235 | 0.954 | 1.014 | 0.639 | 1.607 |
| LMNB1 | 0.015 | 0.215 | 0.943 | 1.015 | 0.667 | 1.547 |
| FOXO3 | 0.020 | 0.215 | 0.926 | 1.020 | 0.669 | 1.555 |
| GRN | 0.025 | 0.215 | 0.906 | 1.026 | 0.674 | 1.562 |
| HSPA9 | 0.028 | 0.215 | 0.895 | 1.029 | 0.675 | 1.569 |
| A2M | 0.030 | 0.215 | 0.891 | 1.030 | 0.676 | 1.570 |
| POLB | 0.030 | 0.215 | 0.888 | 1.031 | 0.676 | 1.571 |
| XRCC5 | 0.033 | 0.216 | 0.879 | 1.033 | 0.677 | 1.578 |
| NBN | 0.034 | 0.215 | 0.876 | 1.034 | 0.679 | 1.575 |
| CDKN2A | 0.033 | 0.215 | 0.877 | 1.034 | 0.679 | 1.575 |
| SIN3A | 0.034 | 0.215 | 0.874 | 1.035 | 0.679 | 1.575 |
| POLA1 | 0.036 | 0.215 | 0.866 | 1.037 | 0.681 | 1.579 |
| SSTR3 | 0.038 | 0.215 | 0.858 | 1.039 | 0.682 | 1.585 |
| BRCA2 | 0.039 | 0.215 | 0.857 | 1.040 | 0.682 | 1.584 |
| PROP1 | 0.042 | 0.215 | 0.845 | 1.043 | 0.684 | 1.589 |
| INS | 0.042 | 0.353 | 0.906 | 1.043 | 0.522 | 2.084 |
| PPARG | 0.044 | 0.216 | 0.840 | 1.045 | 0.684 | 1.596 |
| DDIT3 | 0.048 | 0.215 | 0.823 | 1.049 | 0.688 | 1.600 |
| SUN1 | 0.049 | 0.215 | 0.820 | 1.050 | 0.689 | 1.601 |
| TFAP2A | 0.052 | 0.215 | 0.808 | 1.054 | 0.691 | 1.605 |
| PDGFRB | 0.054 | 0.217 | 0.803 | 1.055 | 0.690 | 1.614 |
| SQSTM1 | 0.054 | 0.216 | 0.802 | 1.056 | 0.691 | 1.612 |
| EEF1A1 | 0.056 | 0.217 | 0.797 | 1.057 | 0.692 | 1.617 |
| MAPT | 0.056 | 0.215 | 0.793 | 1.058 | 0.694 | 1.612 |
| CDK1 | 0.057 | 0.215 | 0.789 | 1.059 | 0.695 | 1.614 |
| NCOR1 | 0.058 | 0.215 | 0.788 | 1.059 | 0.696 | 1.614 |
| GHRHR | 0.060 | 0.221 | 0.786 | 1.062 | 0.688 | 1.638 |
| PEX5 | 0.063 | 0.215 | 0.771 | 1.065 | 0.698 | 1.623 |
| PLCG2 | 0.063 | 0.215 | 0.771 | 1.065 | 0.698 | 1.624 |
| IFNB1 | 0.065 | 0.215 | 0.762 | 1.067 | 0.700 | 1.627 |
| IRS2 | 0.076 | 0.215 | 0.725 | 1.079 | 0.707 | 1.645 |
| BSCL2 | 0.087 | 0.215 | 0.686 | 1.091 | 0.716 | 1.662 |
| LEPR | 0.094 | 0.215 | 0.661 | 1.099 | 0.721 | 1.675 |
| MAPK8 | 0.094 | 0.215 | 0.661 | 1.099 | 0.721 | 1.676 |
| TOP2A | 0.096 | 0.215 | 0.656 | 1.101 | 0.722 | 1.679 |
| GHRH | 0.102 | 0.216 | 0.637 | 1.107 | 0.726 | 1.690 |
| CHEK2 | 0.106 | 0.215 | 0.623 | 1.112 | 0.729 | 1.695 |
| BRCA1 | 0.115 | 0.215 | 0.591 | 1.122 | 0.737 | 1.710 |
| AGTR1 | 0.117 | 0.215 | 0.585 | 1.124 | 0.738 | 1.712 |
| EP300 | 0.117 | 0.216 | 0.587 | 1.125 | 0.736 | 1.718 |
| MLH1 | 0.123 | 0.217 | 0.570 | 1.131 | 0.740 | 1.729 |
| TBP | 0.124 | 0.216 | 0.568 | 1.132 | 0.740 | 1.729 |
| TP73 | 0.126 | 0.215 | 0.558 | 1.135 | 0.744 | 1.731 |
| RET | 0.128 | 0.216 | 0.554 | 1.136 | 0.744 | 1.736 |
| EEF1E1 | 0.129 | 0.215 | 0.549 | 1.137 | 0.747 | 1.733 |
| APOE | 0.130 | 0.215 | 0.545 | 1.139 | 0.747 | 1.737 |
| IL7R | 0.134 | 0.215 | 0.532 | 1.144 | 0.751 | 1.743 |
| IL2RG | 0.135 | 0.217 | 0.533 | 1.145 | 0.749 | 1.750 |
| MAPK14 | 0.138 | 0.215 | 0.521 | 1.148 | 0.753 | 1.752 |
| PTEN | 0.140 | 0.215 | 0.516 | 1.150 | 0.754 | 1.754 |
| TERF1 | 0.142 | 0.215 | 0.509 | 1.152 | 0.757 | 1.755 |
| E2F1 | 0.142 | 0.216 | 0.510 | 1.153 | 0.755 | 1.760 |
| PIK3CA | 0.147 | 0.215 | 0.496 | 1.158 | 0.759 | 1.765 |
| FGFR1 | 0.148 | 0.216 | 0.493 | 1.159 | 0.759 | 1.770 |
| IGFBP2 | 0.159 | 0.215 | 0.461 | 1.172 | 0.769 | 1.787 |
| KL | 0.160 | 0.216 | 0.457 | 1.174 | 0.769 | 1.792 |
| SIRT1 | 0.163 | 0.215 | 0.450 | 1.177 | 0.772 | 1.796 |
| TAF1 | 0.164 | 0.215 | 0.445 | 1.178 | 0.773 | 1.796 |
| APP | 0.169 | 0.215 | 0.432 | 1.185 | 0.777 | 1.807 |
| EIF5A2 | 0.174 | 0.216 | 0.422 | 1.189 | 0.779 | 1.817 |
| RICTOR | 0.173 | 0.215 | 0.422 | 1.189 | 0.779 | 1.813 |
| TNF | 0.177 | 0.216 | 0.411 | 1.194 | 0.782 | 1.821 |
| VEGFA | 0.181 | 0.216 | 0.402 | 1.198 | 0.785 | 1.828 |
| CREBBP | 0.183 | 0.216 | 0.397 | 1.200 | 0.786 | 1.833 |
| CREB1 | 0.185 | 0.215 | 0.390 | 1.203 | 0.789 | 1.834 |
| FLT1 | 0.192 | 0.218 | 0.379 | 1.212 | 0.790 | 1.858 |
| BUB1B | 0.196 | 0.215 | 0.363 | 1.217 | 0.798 | 1.856 |
| EGR1 | 0.208 | 0.215 | 0.334 | 1.231 | 0.808 | 1.877 |
| CLOCK | 0.209 | 0.215 | 0.331 | 1.232 | 0.809 | 1.877 |
| TP53BP1 | 0.216 | 0.216 | 0.319 | 1.241 | 0.812 | 1.896 |
| ERCC3 | 0.217 | 0.221 | 0.328 | 1.242 | 0.805 | 1.916 |
| HIF1A | 0.220 | 0.216 | 0.307 | 1.247 | 0.817 | 1.903 |
| NRG1 | 0.226 | 0.216 | 0.294 | 1.254 | 0.822 | 1.912 |
| SST | 0.228 | 0.353 | 0.518 | 1.256 | 0.629 | 2.511 |
| ERCC8 | 0.234 | 0.216 | 0.277 | 1.264 | 0.828 | 1.930 |
| CSNK1E | 0.236 | 0.218 | 0.278 | 1.266 | 0.827 | 1.940 |
| TOP2B | 0.238 | 0.216 | 0.270 | 1.268 | 0.831 | 1.935 |
| CNR1 | 0.265 | 0.216 | 0.220 | 1.304 | 0.854 | 1.991 |
| WRN | 0.268 | 0.216 | 0.215 | 1.308 | 0.856 | 1.997 |
| CETP | 0.281 | 0.215 | 0.191 | 1.325 | 0.869 | 2.019 |
| ATR | 0.283 | 0.217 | 0.191 | 1.327 | 0.868 | 2.028 |
| SERPINE1 | 0.287 | 0.217 | 0.186 | 1.332 | 0.871 | 2.037 |
| PPM1D | 0.289 | 0.215 | 0.179 | 1.335 | 0.876 | 2.037 |
| STAT3 | 0.294 | 0.219 | 0.179 | 1.342 | 0.874 | 2.062 |
| MSRA | 0.296 | 0.217 | 0.172 | 1.344 | 0.879 | 2.055 |
| IL6 | 0.300 | 0.215 | 0.164 | 1.349 | 0.885 | 2.058 |
| NOG | 0.302 | 0.216 | 0.162 | 1.352 | 0.886 | 2.063 |
| JUN | 0.305 | 0.216 | 0.157 | 1.357 | 0.889 | 2.072 |
| PCK1 | 0.311 | 0.215 | 0.149 | 1.365 | 0.895 | 2.081 |
| PIK3R1 | 0.321 | 0.216 | 0.138 | 1.378 | 0.902 | 2.104 |
| STAT5B | 0.347 | 0.217 | 0.110 | 1.415 | 0.925 | 2.164 |
| GDF11 | 0.352 | 0.217 | 0.105 | 1.423 | 0.929 | 2.179 |
| DLL3 | 0.358 | 0.216 | 0.097 | 1.431 | 0.937 | 2.186 |
| FGF23 | 0.367 | 0.215 | 0.088 | 1.443 | 0.947 | 2.200 |
| MAX | 0.374 | 0.217 | 0.085 | 1.454 | 0.949 | 2.226 |
| TOP3B | 0.382 | 0.217 | 0.079 | 1.465 | 0.957 | 2.244 |
| IL2 | 0.386 | 0.242 | 0.111 | 1.471 | 0.916 | 2.364 |
| IRS1 | 0.387 | 0.217 | 0.075 | 1.472 | 0.962 | 2.251 |
| EFEMP1 | 0.397 | 0.216 | 0.067 | 1.487 | 0.973 | 2.273 |
| FOS | 0.410 | 0.215 | 0.057 | 1.506 | 0.988 | 2.298 |
| BCL2 | 0.418 | 0.217 | 0.055 | 1.519 | 0.992 | 2.325 |
| IGF1R | 0.433 | 0.217 | 0.046 | 1.542 | 1.008 | 2.358 |
| PIK3CB | 0.455 | 0.218 | 0.037 | 1.576 | 1.028 | 2.418 |
| IGFBP3 | 0.473 | 0.217 | 0.029 | 1.605 | 1.049 | 2.455 |
| IGF2 | 0.479 | 0.216 | 0.027 | 1.615 | 1.057 | 2.467 |
| INSR | 0.484 | 0.217 | 0.026 | 1.623 | 1.061 | 2.484 |
| MXD1 | 0.491 | 0.219 | 0.025 | 1.633 | 1.063 | 2.510 |
| TPP2 | 0.511 | 0.219 | 0.020 | 1.668 | 1.085 | 2.563 |
| EGFR | 0.517 | 0.218 | 0.018 | 1.676 | 1.094 | 2.568 |
| AR | 0.518 | 0.217 | 0.017 | 1.678 | 1.096 | 2.569 |
| GHR | 0.530 | 0.218 | 0.015 | 1.699 | 1.108 | 2.606 |
| HBP1 | 0.537 | 0.219 | 0.014 | 1.712 | 1.115 | 2.628 |
| LEP | 0.602 | 0.217 | 0.006 | 1.826 | 1.193 | 2.796 |
| PDGFRA | 0.659 | 0.222 | 0.003 | 1.932 | 1.250 | 2.986 |
