# Supplementary material for: Identification and validation of a 7-genes prognostic signature for adult acute myeloid leukemia based on aging-related genes
Source: Aging (Albany NY). 2023 Jun 26;15(12):5826–53. doi: 10.18632/aging.204843 (PMC10333094; doi:10.18632/aging.204843)
Supplement: Supplementary Table 3 [file aging-15-204843-s004.pdf]

Supplementary Table 3. 48 ARGs were associated with prognosis by univariate analyses.

Gene name	Coef	Se	P	HR	95%CI low	95%CI High
LMNA	-0.845	0.223	<0.001	0.429	0.277	0.665
HSPA1B	-0.844	0.224	<0.001	0.430	0.277	0.666
PTPN1	-0.780	0.221	<0.001	0.458	0.297	0.706
UCP2	-0.748	0.220	0.001	0.473	0.308	0.728
GPX1	-0.739	0.220	0.001	0.478	0.310	0.735
SIRT6	-0.721	0.218	0.001	0.486	0.317	0.746
BAK1	-0.708	0.218	0.001	0.493	0.321	0.756
TERF2	-0.694	0.218	0.001	0.500	0.326	0.766
TGFB1	-0.673	0.220	0.002	0.510	0.331	0.785
NUDT1	-0.656	0.218	0.003	0.519	0.339	0.795
ELN	-0.631	0.220	0.004	0.532	0.346	0.819
TRAP1	-0.606	0.217	0.005	0.545	0.356	0.835
PPP1CA	-0.606	0.217	0.005	0.546	0.357	0.834
ERCC1	-0.599	0.218	0.006	0.549	0.358	0.843
HDAC3	-0.581	0.218	0.008	0.559	0.365	0.857
GPX4	-0.578	0.219	0.008	0.561	0.365	0.862
GSTP1	-0.571	0.218	0.009	0.565	0.369	0.865
STK11	-0.551	0.219	0.012	0.576	0.375	0.886
GSK3A	-0.551	0.217	0.011	0.576	0.377	0.881
SOCS2	-0.530	0.216	0.014	0.589	0.385	0.900
SOD1	-0.521	0.217	0.016	0.594	0.388	0.908
NFKB2	-0.510	0.217	0.019	0.600	0.393	0.918
HSPA1A	-0.506	0.218	0.020	0.603	0.393	0.924
EMD	-0.498	0.216	0.021	0.608	0.398	0.928
PRKCD	-0.492	0.218	0.024	0.611	0.399	0.936
ERCC2	-0.483	0.217	0.026	0.617	0.403	0.944
PRDX1	-0.482	0.216	0.026	0.618	0.404	0.944
DGAT1	-0.481	0.217	0.026	0.618	0.404	0.945
UCP3	-0.470	0.216	0.030	0.625	0.410	0.955
SPRTN	-0.460	0.219	0.036	0.631	0.411	0.971
PTK2B	-0.461	0.217	0.034	0.631	0.412	0.966
MAP3K5	-0.458	0.218	0.035	0.633	0.413	0.969
MT1E	-0.443	0.219	0.043	0.642	0.418	0.987
GCLM	-0.437	0.217	0.043	0.646	0.422	0.987
BMI1	-0.435	0.217	0.044	0.647	0.423	0.989
IGF1R	0.433	0.217	0.046	1.542	1.008	2.358
PIK3CB	0.455	0.218	0.037	1.576	1.028	2.418
IGFBP3	0.473	0.217	0.029	1.605	1.049	2.455
IGF2	0.479	0.216	0.027	1.615	1.057	2.467
INSR	0.484	0.217	0.026	1.623	1.061	2.484
MXD1	0.491	0.219	0.025	1.633	1.063	2.510
TPP2	0.511	0.219	0.020	1.668	1.085	2.563
EGFR	0.517	0.218	0.018	1.676	1.094	2.568
AR	0.518	0.217	0.017	1.678	1.096	2.569
GHR	0.530	0.218	0.015	1.699	1.108	2.606
HBP1	0.537	0.219	0.014	1.712	1.115	2.628
LEP	0.602	0.217	0.006	1.826	1.193	2.796
PDGFRA	0.659	0.222	0.003	1.932	1.250	2.986
