# Supplementary material for: Identification and validation of a 7-genes prognostic signature for adult acute myeloid leukemia based on aging-related genes
Source: Aging (Albany NY). 2023 Jun 26;15(12):5826–53. doi: 10.18632/aging.204843 (PMC10333094; doi:10.18632/aging.204843)
Supplement: Supplementary Table 4 [file aging-15-204843-s005.docx]

| **Supplementary Table 4. List of Pan-cancer immune metagenes.** | | |
| --- | --- | --- |
| **Metagene** | **Cell type** | **Immunity** |
| ADAM28 | Activated B cell | Adaptive |
| CD180 | Activated B cell | Adaptive |
| CD79B | Activated B cell | Adaptive |
| BLK | Activated B cell | Adaptive |
| CD19 | Activated B cell | Adaptive |
| MS4A1 | Activated B cell | Adaptive |
| TNFRSF17 | Activated B cell | Adaptive |
| IGHM | Activated B cell | Adaptive |
| GNG7 | Activated B cell | Adaptive |
| MICAL3 | Activated B cell | Adaptive |
| SPIB | Activated B cell | Adaptive |
| HLA-DOB | Activated B cell | Adaptive |
| IGKC | Activated B cell | Adaptive |
| PNOC | Activated B cell | Adaptive |
| FCRL2 | Activated B cell | Adaptive |
| BACH2 | Activated B cell | Adaptive |
| CR2 | Activated B cell | Adaptive |
| TCL1A | Activated B cell | Adaptive |
| AKNA | Activated B cell | Adaptive |
| ARHGAP25 | Activated B cell | Adaptive |
| CCL21 | Activated B cell | Adaptive |
| CD27 | Activated B cell | Adaptive |
| CD38 | Activated B cell | Adaptive |
| CLEC17A | Activated B cell | Adaptive |
| CLEC9A | Activated B cell | Adaptive |
| CLECL1 | Activated B cell | Adaptive |
| AIM2 | Activated CD4 T cell | Adaptive |
| BIRC3 | Activated CD4 T cell | Adaptive |
| BRIP1 | Activated CD4 T cell | Adaptive |
| CCL20 | Activated CD4 T cell | Adaptive |
| CCL4 | Activated CD4 T cell | Adaptive |
| CCL5 | Activated CD4 T cell | Adaptive |
| CCNB1 | Activated CD4 T cell | Adaptive |
| CCR7 | Activated CD4 T cell | Adaptive |
| DUSP2 | Activated CD4 T cell | Adaptive |
| ESCO2 | Activated CD4 T cell | Adaptive |
| ETS1 | Activated CD4 T cell | Adaptive |
| EXO1 | Activated CD4 T cell | Adaptive |
| EXOC6 | Activated CD4 T cell | Adaptive |
| IARS | Activated CD4 T cell | Adaptive |
| ITK | Activated CD4 T cell | Adaptive |
| KIF11 | Activated CD4 T cell | Adaptive |
| KNTC1 | Activated CD4 T cell | Adaptive |
| NUF2 | Activated CD4 T cell | Adaptive |
| PRC1 | Activated CD4 T cell | Adaptive |
| PSAT1 | Activated CD4 T cell | Adaptive |
| RGS1 | Activated CD4 T cell | Adaptive |
| RTKN2 | Activated CD4 T cell | Adaptive |
| SAMSN1 | Activated CD4 T cell | Adaptive |
| SELL | Activated CD4 T cell | Adaptive |
| TRAT1 | Activated CD4 T cell | Adaptive |
| ADRM1 | Activated CD8 T cell | Adaptive |
| AHSA1 | Activated CD8 T cell | Adaptive |
| C1GALT1C1 | Activated CD8 T cell | Adaptive |
| CCT6B | Activated CD8 T cell | Adaptive |
| CD37 | Activated CD8 T cell | Adaptive |
| CD3D | Activated CD8 T cell | Adaptive |
| CD3E | Activated CD8 T cell | Adaptive |
| CD3G | Activated CD8 T cell | Adaptive |
| CD69 | Activated CD8 T cell | Adaptive |
| CD8A | Activated CD8 T cell | Adaptive |
| CETN3 | Activated CD8 T cell | Adaptive |
| CSE1L | Activated CD8 T cell | Adaptive |
| GEMIN6 | Activated CD8 T cell | Adaptive |
| GNLY | Activated CD8 T cell | Adaptive |
| GPT2 | Activated CD8 T cell | Adaptive |
| GZMA | Activated CD8 T cell | Adaptive |
| GZMH | Activated CD8 T cell | Adaptive |
| GZMK | Activated CD8 T cell | Adaptive |
| IL2RB | Activated CD8 T cell | Adaptive |
| LCK | Activated CD8 T cell | Adaptive |
| MPZL1 | Activated CD8 T cell | Adaptive |
| NKG7 | Activated CD8 T cell | Adaptive |
| PIK3IP1 | Activated CD8 T cell | Adaptive |
| PTRH2 | Activated CD8 T cell | Adaptive |
| TIMM13 | Activated CD8 T cell | Adaptive |
| ZAP70 | Activated CD8 T cell | Adaptive |
| ABHD3 | Central memory CD4 T cell | Adaptive |
| AHNAK | Central memory CD4 T cell | Adaptive |
| ANXA2P2 | Central memory CD4 T cell | Adaptive |
| AQP3 | Central memory CD4 T cell | Adaptive |
| ATHL1 | Central memory CD4 T cell | Adaptive |
| BMI1 | Central memory CD4 T cell | Adaptive |
| BZW2 | Central memory CD4 T cell | Adaptive |
| CD63 | Central memory CD4 T cell | Adaptive |
| COL4A1 | Central memory CD4 T cell | Adaptive |
| CYLD | Central memory CD4 T cell | Adaptive |
| ELMO2 | Central memory CD4 T cell | Adaptive |
| FYN | Central memory CD4 T cell | Adaptive |
| GLIPR1 | Central memory CD4 T cell | Adaptive |
| GSS | Central memory CD4 T cell | Adaptive |
| IFITM2 | Central memory CD4 T cell | Adaptive |
| ITGB1 | Central memory CD4 T cell | Adaptive |
| ITGB2 | Central memory CD4 T cell | Adaptive |
| KLF5 | Central memory CD4 T cell | Adaptive |
| LSP1 | Central memory CD4 T cell | Adaptive |
| NDUFB9 | Central memory CD4 T cell | Adaptive |
| PKM2 | Central memory CD4 T cell | Adaptive |
| SFXN3 | Central memory CD4 T cell | Adaptive |
| SIRPG | Central memory CD4 T cell | Adaptive |
| SMAD4 | Central memory CD4 T cell | Adaptive |
| STX4 | Central memory CD4 T cell | Adaptive |
| TRADD | Central memory CD4 T cell | Adaptive |
| VIM | Central memory CD4 T cell | Adaptive |
| XRCC6 | Central memory CD4 T cell | Adaptive |
| ACTN4 | Central memory CD8 T cell | Adaptive |
| ADAM12 | Central memory CD8 T cell | Adaptive |
| ADCY9 | Central memory CD8 T cell | Adaptive |
| F13A1 | Central memory CD8 T cell | Adaptive |
| FCER1G | Central memory CD8 T cell | Adaptive |
| FCGR3B | Central memory CD8 T cell | Adaptive |
| FGF7 | Central memory CD8 T cell | Adaptive |
| FKBP4 | Central memory CD8 T cell | Adaptive |
| GLUD1 | Central memory CD8 T cell | Adaptive |
| GM2A | Central memory CD8 T cell | Adaptive |
| GUSB | Central memory CD8 T cell | Adaptive |
| IL1RN | Central memory CD8 T cell | Adaptive |
| NOL11 | Central memory CD8 T cell | Adaptive |
| NTRK1 | Central memory CD8 T cell | Adaptive |
| RARA | Central memory CD8 T cell | Adaptive |
| RNF128 | Central memory CD8 T cell | Adaptive |
| SIGLEC1 | Central memory CD8 T cell | Adaptive |
| TNFRSF11A | Central memory CD8 T cell | Adaptive |
| TOX4 | Central memory CD8 T cell | Adaptive |
| UBA52 | Central memory CD8 T cell | Adaptive |
| ULBP1 | Central memory CD8 T cell | Adaptive |
| ATM | Effector memeory CD4 T cell | Adaptive |
| CASP3 | Effector memeory CD4 T cell | Adaptive |
| CASQ1 | Effector memeory CD4 T cell | Adaptive |
| CD300E | Effector memeory CD4 T cell | Adaptive |
| DARS | Effector memeory CD4 T cell | Adaptive |
| DOCK9 | Effector memeory CD4 T cell | Adaptive |
| EXOSC9 | Effector memeory CD4 T cell | Adaptive |
| EZH2 | Effector memeory CD4 T cell | Adaptive |
| GDE1 | Effector memeory CD4 T cell | Adaptive |
| IL34 | Effector memeory CD4 T cell | Adaptive |
| NCOA4 | Effector memeory CD4 T cell | Adaptive |
| NEFL | Effector memeory CD4 T cell | Adaptive |
| PDGFRL | Effector memeory CD4 T cell | Adaptive |
| PTGS1 | Effector memeory CD4 T cell | Adaptive |
| REPS1 | Effector memeory CD4 T cell | Adaptive |
| SCG2 | Effector memeory CD4 T cell | Adaptive |
| SDPR | Effector memeory CD4 T cell | Adaptive |
| SIGLEC14 | Effector memeory CD4 T cell | Adaptive |
| SIGLEC6 | Effector memeory CD4 T cell | Adaptive |
| TAL1 | Effector memeory CD4 T cell | Adaptive |
| TFEC | Effector memeory CD4 T cell | Adaptive |
| TIPIN | Effector memeory CD4 T cell | Adaptive |
| TPK1 | Effector memeory CD4 T cell | Adaptive |
| UQCRB | Effector memeory CD4 T cell | Adaptive |
| USP9Y | Effector memeory CD4 T cell | Adaptive |
| WIPF1 | Effector memeory CD4 T cell | Adaptive |
| ZCRB1 | Effector memeory CD4 T cell | Adaptive |
| ACAP1 | Effector memeory CD8 T cell | Adaptive |
| APOL3 | Effector memeory CD8 T cell | Adaptive |
| ARHGAP10 | Effector memeory CD8 T cell | Adaptive |
| ATP10D | Effector memeory CD8 T cell | Adaptive |
| C3AR1 | Effector memeory CD8 T cell | Adaptive |
| CCR5 | Effector memeory CD8 T cell | Adaptive |
| CD160 | Effector memeory CD8 T cell | Adaptive |
| CD55 | Effector memeory CD8 T cell | Adaptive |
| CFLAR | Effector memeory CD8 T cell | Adaptive |
| CMKLR1 | Effector memeory CD8 T cell | Adaptive |
| DAPP1 | Effector memeory CD8 T cell | Adaptive |
| FCRL6 | Effector memeory CD8 T cell | Adaptive |
| FLT3LG | Effector memeory CD8 T cell | Adaptive |
| GZMM | Effector memeory CD8 T cell | Adaptive |
| HAPLN3 | Effector memeory CD8 T cell | Adaptive |
| HLA-DMB | Effector memeory CD8 T cell | Adaptive |
| HLA-DPA1 | Effector memeory CD8 T cell | Adaptive |
| HLA-DPB1 | Effector memeory CD8 T cell | Adaptive |
| IFI16 | Effector memeory CD8 T cell | Adaptive |
| LIME1 | Effector memeory CD8 T cell | Adaptive |
| LTK | Effector memeory CD8 T cell | Adaptive |
| NFKBIA | Effector memeory CD8 T cell | Adaptive |
| SETD7 | Effector memeory CD8 T cell | Adaptive |
| SIK1 | Effector memeory CD8 T cell | Adaptive |
| TRIB2 | Effector memeory CD8 T cell | Adaptive |
| ACP5 | Gamma delta T cell | Adaptive |
| AQP9 | Gamma delta T cell | Adaptive |
| BTN3A2 | Gamma delta T cell | Adaptive |
| C1orf54 | Gamma delta T cell | Adaptive |
| CARD8 | Gamma delta T cell | Adaptive |
| CCL18 | Gamma delta T cell | Adaptive |
| CD209 | Gamma delta T cell | Adaptive |
| CD33 | Gamma delta T cell | Adaptive |
| CD36 | Gamma delta T cell | Adaptive |
| CDK5 | Gamma delta T cell | Adaptive |
| IL10RB | Gamma delta T cell | Adaptive |
| KLRF1 | Gamma delta T cell | Adaptive |
| LGALS1 | Gamma delta T cell | Adaptive |
| MAPK7 | Gamma delta T cell | Adaptive |
| KLHL7 | Gamma delta T cell | Adaptive |
| KRT80 | Gamma delta T cell | Adaptive |
| LAMC1 | Gamma delta T cell | Adaptive |
| LCORL | Gamma delta T cell | Adaptive |
| LMNB1 | Gamma delta T cell | Adaptive |
| MEIS3P1 | Gamma delta T cell | Adaptive |
| MPL | Gamma delta T cell | Adaptive |
| FABP1 | Gamma delta T cell | Adaptive |
| FABP5 | Gamma delta T cell | Adaptive |
| FADD | Gamma delta T cell | Adaptive |
| MFAP3L | Gamma delta T cell | Adaptive |
| MINPP1 | Gamma delta T cell | Adaptive |
| RPS24 | Gamma delta T cell | Adaptive |
| RPS7 | Gamma delta T cell | Adaptive |
| RPS9 | Gamma delta T cell | Adaptive |
| DBNL | Gamma delta T cell | Adaptive |
| CCL13 | Gamma delta T cell | Adaptive |
| CD22 | Immature B cell | Adaptive |
| CYBB | Immature B cell | Adaptive |
| FAM129C | Immature B cell | Adaptive |
| FCRL1 | Immature B cell | Adaptive |
| FCRL3 | Immature B cell | Adaptive |
| FCRL5 | Immature B cell | Adaptive |
| FCRLA | Immature B cell | Adaptive |
| HDAC9 | Immature B cell | Adaptive |
| HLA-DQA1 | Immature B cell | Adaptive |
| HVCN1 | Immature B cell | Adaptive |
| KIAA0226 | Immature B cell | Adaptive |
| NCF1 | Immature B cell | Adaptive |
| NCF1B | Immature B cell | Adaptive |
| P2RY10 | Immature B cell | Adaptive |
| SP100 | Immature B cell | Adaptive |
| TXNIP | Immature B cell | Adaptive |
| STAP1 | Immature B cell | Adaptive |
| TAGAP | Immature B cell | Adaptive |
| ZCCHC2 | Immature B cell | Adaptive |
| AICDA | Memory B cell | Adaptive |
| CCNA2 | Memory B cell | Adaptive |
| CDKN3 | Memory B cell | Adaptive |
| CLCN5 | Memory B cell | Adaptive |
| ENPP1 | Memory B cell | Adaptive |
| FCER1A | Memory B cell | Adaptive |
| FCRL4 | Memory B cell | Adaptive |
| MYC | Memory B cell | Adaptive |
| RUNX2 | Memory B cell | Adaptive |
| SORL1 | Memory B cell | Adaptive |
| SOX5 | Memory B cell | Adaptive |
| STAT5A | Memory B cell | Adaptive |
| STAT5B | Memory B cell | Adaptive |
| TLR9 | Memory B cell | Adaptive |
| CCL3L1 | Regulatory T cell | Adaptive |
| CD72 | Regulatory T cell | Adaptive |
| CLEC5A | Regulatory T cell | Adaptive |
| FOXP3 | Regulatory T cell | Adaptive |
| ITGA4 | Regulatory T cell | Adaptive |
| L1CAM | Regulatory T cell | Adaptive |
| LIPA | Regulatory T cell | Adaptive |
| LRP1 | Regulatory T cell | Adaptive |
| LRRC42 | Regulatory T cell | Adaptive |
| MARCO | Regulatory T cell | Adaptive |
| MMP12 | Regulatory T cell | Adaptive |
| MNDA | Regulatory T cell | Adaptive |
| MRC1 | Regulatory T cell | Adaptive |
| MS4A6A | Regulatory T cell | Adaptive |
| PELO | Regulatory T cell | Adaptive |
| PLEK | Regulatory T cell | Adaptive |
| PRSS23 | Regulatory T cell | Adaptive |
| PTGIR | Regulatory T cell | Adaptive |
| ST8SIA4 | Regulatory T cell | Adaptive |
| STAB1 | Regulatory T cell | Adaptive |
| B3GAT1 | T follicular helper cell | Adaptive |
| CDK5R1 | T follicular helper cell | Adaptive |
| PDCD1 | T follicular helper cell | Adaptive |
| BCL6 | T follicular helper cell | Adaptive |
| CD200 | T follicular helper cell | Adaptive |
| CD83 | T follicular helper cell | Adaptive |
| CD84 | T follicular helper cell | Adaptive |
| FGF2 | T follicular helper cell | Adaptive |
| GPR18 | T follicular helper cell | Adaptive |
| CEBPA | T follicular helper cell | Adaptive |
| CECR1 | T follicular helper cell | Adaptive |
| CLEC10A | T follicular helper cell | Adaptive |
| CLEC4A | T follicular helper cell | Adaptive |
| CSF1R | T follicular helper cell | Adaptive |
| CTSS | T follicular helper cell | Adaptive |
| DMN | T follicular helper cell | Adaptive |
| DPP4 | T follicular helper cell | Adaptive |
| LRRC32 | T follicular helper cell | Adaptive |
| MC5R | T follicular helper cell | Adaptive |
| MICA | T follicular helper cell | Adaptive |
| NCAM1 | T follicular helper cell | Adaptive |
| NCR2 | T follicular helper cell | Adaptive |
| NRP1 | T follicular helper cell | Adaptive |
| PDCD1LG2 | T follicular helper cell | Adaptive |
| PDCD6 | T follicular helper cell | Adaptive |
| PRDX1 | T follicular helper cell | Adaptive |
| RAE1 | T follicular helper cell | Adaptive |
| RAET1E | T follicular helper cell | Adaptive |
| SIGLEC7 | T follicular helper cell | Adaptive |
| SIGLEC9 | T follicular helper cell | Adaptive |
| TYRO3 | T follicular helper cell | Adaptive |
| CHST12 | T follicular helper cell | Adaptive |
| CLIC3 | T follicular helper cell | Adaptive |
| IVNS1ABP | T follicular helper cell | Adaptive |
| KIR2DL2 | T follicular helper cell | Adaptive |
| LGMN | T follicular helper cell | Adaptive |
| CD70 | Type 1 T helper cell | Adaptive |
| TBX21 | Type 1 T helper cell | Adaptive |
| ADAM8 | Type 1 T helper cell | Adaptive |
| AHCYL2 | Type 1 T helper cell | Adaptive |
| ALCAM | Type 1 T helper cell | Adaptive |
| B3GALNT1 | Type 1 T helper cell | Adaptive |
| BBS12 | Type 1 T helper cell | Adaptive |
| BST1 | Type 1 T helper cell | Adaptive |
| CD151 | Type 1 T helper cell | Adaptive |
| CD47 | Type 1 T helper cell | Adaptive |
| CD48 | Type 1 T helper cell | Adaptive |
| CD52 | Type 1 T helper cell | Adaptive |
| CD53 | Type 1 T helper cell | Adaptive |
| CD59 | Type 1 T helper cell | Adaptive |
| CD6 | Type 1 T helper cell | Adaptive |
| CD68 | Type 1 T helper cell | Adaptive |
| CD7 | Type 1 T helper cell | Adaptive |
| CD96 | Type 1 T helper cell | Adaptive |
| CFHR3 | Type 1 T helper cell | Adaptive |
| CHRM3 | Type 1 T helper cell | Adaptive |
| CLEC7A | Type 1 T helper cell | Adaptive |
| COL23A1 | Type 1 T helper cell | Adaptive |
| COL4A4 | Type 1 T helper cell | Adaptive |
| COL5A3 | Type 1 T helper cell | Adaptive |
| DAB1 | Type 1 T helper cell | Adaptive |
| DLEU7 | Type 1 T helper cell | Adaptive |
| DOC2B | Type 1 T helper cell | Adaptive |
| EMP1 | Type 1 T helper cell | Adaptive |
| F12 | Type 1 T helper cell | Adaptive |
| FURIN | Type 1 T helper cell | Adaptive |
| GAB3 | Type 1 T helper cell | Adaptive |
| GATM | Type 1 T helper cell | Adaptive |
| GFPT2 | Type 1 T helper cell | Adaptive |
| GPR25 | Type 1 T helper cell | Adaptive |
| GREM2 | Type 1 T helper cell | Adaptive |
| HAVCR1 | Type 1 T helper cell | Adaptive |
| HSD11B1 | Type 1 T helper cell | Adaptive |
| HUNK | Type 1 T helper cell | Adaptive |
| IGF2 | Type 1 T helper cell | Adaptive |
| RCSD1 | Type 1 T helper cell | Adaptive |
| RYR1 | Type 1 T helper cell | Adaptive |
| SAV1 | Type 1 T helper cell | Adaptive |
| SELE | Type 1 T helper cell | Adaptive |
| SELP | Type 1 T helper cell | Adaptive |
| SH3KBP1 | Type 1 T helper cell | Adaptive |
| SIT1 | Type 1 T helper cell | Adaptive |
| SLC35B3 | Type 1 T helper cell | Adaptive |
| SIGLEC10 | Type 1 T helper cell | Adaptive |
| SKAP1 | Type 1 T helper cell | Adaptive |
| THUMPD2 | Type 1 T helper cell | Adaptive |
| TIGIT | Type 1 T helper cell | Adaptive |
| ZEB2 | Type 1 T helper cell | Adaptive |
| ENC1 | Type 1 T helper cell | Adaptive |
| FAM134B | Type 1 T helper cell | Adaptive |
| FBXO30 | Type 1 T helper cell | Adaptive |
| FCGR2C | Type 1 T helper cell | Adaptive |
| STAC | Type 1 T helper cell | Adaptive |
| LTC4S | Type 1 T helper cell | Adaptive |
| MAN1B1 | Type 1 T helper cell | Adaptive |
| MDH1 | Type 1 T helper cell | Adaptive |
| MMD | Type 1 T helper cell | Adaptive |
| RGS16 | Type 1 T helper cell | Adaptive |
| IL12A | Type 1 T helper cell | Adaptive |
| P2RX5 | Type 1 T helper cell | Adaptive |
| CD97 | Type 1 T helper cell | Adaptive |
| ITGB4 | Type 1 T helper cell | Adaptive |
| ICAM3 | Type 1 T helper cell | Adaptive |
| METRNL | Type 1 T helper cell | Adaptive |
| TNFRSF1A | Type 1 T helper cell | Adaptive |
| IRF1 | Type 1 T helper cell | Adaptive |
| HTR2B | Type 1 T helper cell | Adaptive |
| CALD1 | Type 1 T helper cell | Adaptive |
| MOCOS | Type 1 T helper cell | Adaptive |
| TRAF3IP2 | Type 1 T helper cell | Adaptive |
| TLR8 | Type 1 T helper cell | Adaptive |
| TRAF1 | Type 1 T helper cell | Adaptive |
| DUSP14 | Type 1 T helper cell | Adaptive |
| IL17A | Type 17 T helper cell | Adaptive |
| IL17RA | Type 17 T helper cell | Adaptive |
| C2CD4A | Type 17 T helper cell | Adaptive |
| C2CD4B | Type 17 T helper cell | Adaptive |
| CA2 | Type 17 T helper cell | Adaptive |
| CCDC65 | Type 17 T helper cell | Adaptive |
| CEACAM3 | Type 17 T helper cell | Adaptive |
| IL17C | Type 17 T helper cell | Adaptive |
| IL17F | Type 17 T helper cell | Adaptive |
| IL17RC | Type 17 T helper cell | Adaptive |
| IL17RE | Type 17 T helper cell | Adaptive |
| IL23A | Type 17 T helper cell | Adaptive |
| ILDR1 | Type 17 T helper cell | Adaptive |
| LONRF3 | Type 17 T helper cell | Adaptive |
| SH2D6 | Type 17 T helper cell | Adaptive |
| TNIP2 | Type 17 T helper cell | Adaptive |
| ABCA1 | Type 17 T helper cell | Adaptive |
| ABCB1 | Type 17 T helper cell | Adaptive |
| ADAMTS12 | Type 17 T helper cell | Adaptive |
| ANK1 | Type 17 T helper cell | Adaptive |
| ANKRD22 | Type 17 T helper cell | Adaptive |
| B3GALT2 | Type 17 T helper cell | Adaptive |
| CAMTA1 | Type 17 T helper cell | Adaptive |
| CCR9 | Type 17 T helper cell | Adaptive |
| CD40 | Type 17 T helper cell | Adaptive |
| GPR44 | Type 17 T helper cell | Adaptive |
| IFT80 | Type 17 T helper cell | Adaptive |
| ASB2 | Type 2 T helper cell | Adaptive |
| CSRP2 | Type 2 T helper cell | Adaptive |
| DAPK1 | Type 2 T helper cell | Adaptive |
| DLC1 | Type 2 T helper cell | Adaptive |
| DNAJC12 | Type 2 T helper cell | Adaptive |
| DUSP6 | Type 2 T helper cell | Adaptive |
| GNAI1 | Type 2 T helper cell | Adaptive |
| LAMP3 | Type 2 T helper cell | Adaptive |
| NRP2 | Type 2 T helper cell | Adaptive |
| OSBPL1A | Type 2 T helper cell | Adaptive |
| PDE4B | Type 2 T helper cell | Adaptive |
| PHLDA1 | Type 2 T helper cell | Adaptive |
| PLA2G4A | Type 2 T helper cell | Adaptive |
| RAB27B | Type 2 T helper cell | Adaptive |
| RBMS3 | Type 2 T helper cell | Adaptive |
| RNF125 | Type 2 T helper cell | Adaptive |
| TMPRSS3 | Type 2 T helper cell | Adaptive |
| GATA3 | Type 2 T helper cell | Adaptive |
| BIRC5 | Type 2 T helper cell | Adaptive |
| CDC25C | Type 2 T helper cell | Adaptive |
| CDC7 | Type 2 T helper cell | Adaptive |
| CENPF | Type 2 T helper cell | Adaptive |
| CXCR6 | Type 2 T helper cell | Adaptive |
| DHFR | Type 2 T helper cell | Adaptive |
| EVI5 | Type 2 T helper cell | Adaptive |
| GSTA4 | Type 2 T helper cell | Adaptive |
| HELLS | Type 2 T helper cell | Adaptive |
| IL26 | Type 2 T helper cell | Adaptive |
| LAIR2 | Type 2 T helper cell | Adaptive |
| ABCD1 | Activated dendritic cell | Innate |
| C1QC | Activated dendritic cell | Innate |
| CAPG | Activated dendritic cell | Innate |
| CCL3L3 | Activated dendritic cell | Innate |
| CD207 | Activated dendritic cell | Innate |
| CD302 | Activated dendritic cell | Innate |
| ATP5B | Activated dendritic cell | Innate |
| ATP5L | Activated dendritic cell | Innate |
| ATP6V1A | Activated dendritic cell | Innate |
| BCL2L1 | Activated dendritic cell | Innate |
| C1QB | Activated dendritic cell | Innate |
| SNURF | Activated dendritic cell | Innate |
| SPCS3 | Activated dendritic cell | Innate |
| CCNA1 | Activated dendritic cell | Innate |
| CEACAM8 | Activated dendritic cell | Innate |
| NOS2 | Activated dendritic cell | Innate |
| SRA1 | Activated dendritic cell | Innate |
| TNFRSF6B | Activated dendritic cell | Innate |
| TREM1 | Activated dendritic cell | Innate |
| TREML1 | Activated dendritic cell | Innate |
| RHOA | Activated dendritic cell | Innate |
| SLC25A37 | Activated dendritic cell | Innate |
| TNFSF14 | Activated dendritic cell | Innate |
| TREML4 | Activated dendritic cell | Innate |
| VNN2 | Activated dendritic cell | Innate |
| XPO6 | Activated dendritic cell | Innate |
| CLEC4C | Activated dendritic cell | Innate |
| TNFAIP2 | Activated dendritic cell | Innate |
| UBD | Activated dendritic cell | Innate |
| ACTR3 | Activated dendritic cell | Innate |
| RAB1A | Activated dendritic cell | Innate |
| SLA | Activated dendritic cell | Innate |
| HLA-DQA2 | Activated dendritic cell | Innate |
| SIGLEC5 | Activated dendritic cell | Innate |
| SLAMF9 | Activated dendritic cell | Innate |
| ABAT | CD56bright natural killer cell | Innate |
| C11orf75 | CD56bright natural killer cell | Innate |
| C5orf15 | CD56bright natural killer cell | Innate |
| CDHR1 | CD56bright natural killer cell | Innate |
| DCAF12 | CD56bright natural killer cell | Innate |
| DYNLL1 | CD56bright natural killer cell | Innate |
| GPR137B | CD56bright natural killer cell | Innate |
| HCP5 | CD56bright natural killer cell | Innate |
| HDGFRP2 | CD56bright natural killer cell | Innate |
| KRT86 | CD56bright natural killer cell | Innate |
| MLST8 | CD56bright natural killer cell | Innate |
| ELMOD3 | CD56bright natural killer cell | Innate |
| ENTPD5 | CD56bright natural killer cell | Innate |
| FAM119A | CD56bright natural killer cell | Innate |
| FAM179A | CD56bright natural killer cell | Innate |
| CLIC2 | CD56bright natural killer cell | Innate |
| COX7A2L | CD56bright natural killer cell | Innate |
| CREB3L4 | CD56bright natural killer cell | Innate |
| CSF1 | CD56bright natural killer cell | Innate |
| CSNK2A2 | CD56bright natural killer cell | Innate |
| CSTA | CD56bright natural killer cell | Innate |
| CSTB | CD56bright natural killer cell | Innate |
| CTPS | CD56bright natural killer cell | Innate |
| CTSD | CD56bright natural killer cell | Innate |
| FST | CD56bright natural killer cell | Innate |
| GATA2 | CD56bright natural killer cell | Innate |
| GMPR | CD56bright natural killer cell | Innate |
| HDC | CD56bright natural killer cell | Innate |
| HEY1 | CD56bright natural killer cell | Innate |
| HOXA1 | CD56bright natural killer cell | Innate |
| HS2ST1 | CD56bright natural killer cell | Innate |
| HS3ST1 | CD56bright natural killer cell | Innate |
| BCL11B | CD56bright natural killer cell | Innate |
| CDH3 | CD56bright natural killer cell | Innate |
| MYL6B | CD56bright natural killer cell | Innate |
| NAA16 | CD56bright natural killer cell | Innate |
| ClQA | CD56bright natural killer cell | Innate |
| ClQB | CD56bright natural killer cell | Innate |
| CYP27B1 | CD56bright natural killer cell | Innate |
| EIF3M | CD56bright natural killer cell | Innate |
| CYP27A1 | CD56dim natural killer cell | Innate |
| DDX55 | CD56dim natural killer cell | Innate |
| DYRK2 | CD56dim natural killer cell | Innate |
| RPL37A | CD56dim natural killer cell | Innate |
| NOTCH3 | CD56dim natural killer cell | Innate |
| AKR7A3 | CD56dim natural killer cell | Innate |
| GPRC5C | CD56dim natural killer cell | Innate |
| GRIN1 | CD56dim natural killer cell | Innate |
| HLA-E | CD56dim natural killer cell | Innate |
| PORCN | CD56dim natural killer cell | Innate |
| PSMC4 | CD56dim natural killer cell | Innate |
| UPP1 | CD56dim natural killer cell | Innate |
| IL21R | CD56dim natural killer cell | Innate |
| KIR2DS1 | CD56dim natural killer cell | Innate |
| KIR2DS2 | CD56dim natural killer cell | Innate |
| KIR2DS5 | CD56dim natural killer cell | Innate |
| GIPR | Eosinophil | Innate |
| KRT18P50 | Eosinophil | Innate |
| LRMP | Eosinophil | Innate |
| FOSB | Eosinophil | Innate |
| RRP12 | Eosinophil | Innate |
| GPR183 | Eosinophil | Innate |
| NR4A3 | Eosinophil | Innate |
| ST3GAL6 | Eosinophil | Innate |
| DEPDC5 | Eosinophil | Innate |
| PDE6C | Eosinophil | Innate |
| PKD2L2 | Eosinophil | Innate |
| GPR65 | Eosinophil | Innate |
| IL5RA | Eosinophil | Innate |
| P2RY14 | Eosinophil | Innate |
| DACH1 | Eosinophil | Innate |
| DAPK2 | Eosinophil | Innate |
| EMR3 | Eosinophil | Innate |
| ACADM | Immature dendritic cell | Innate |
| AHCYL1 | Immature dendritic cell | Innate |
| ALDH1A2 | Immature dendritic cell | Innate |
| ALDH3A2 | Immature dendritic cell | Innate |
| ALDH9A1 | Immature dendritic cell | Innate |
| ALOX15 | Immature dendritic cell | Innate |
| AMT | Immature dendritic cell | Innate |
| ARL1 | Immature dendritic cell | Innate |
| ATIC | Immature dendritic cell | Innate |
| ATP5A1 | Immature dendritic cell | Innate |
| CAPZA1 | Immature dendritic cell | Innate |
| LILRA5 | Immature dendritic cell | Innate |
| RDX | Immature dendritic cell | Innate |
| RRAGD | Immature dendritic cell | Innate |
| TACSTD2 | Immature dendritic cell | Innate |
| INPP5F | Immature dendritic cell | Innate |
| RAB38 | Immature dendritic cell | Innate |
| PLAU | Immature dendritic cell | Innate |
| CSF3R | Immature dendritic cell | Innate |
| SLC18A2 | Immature dendritic cell | Innate |
| AMPD2 | Immature dendritic cell | Innate |
| CLTB | Immature dendritic cell | Innate |
| C1orf162 | Immature dendritic cell | Innate |
| AIF1 | Macrophage | Innate |
| CCL1 | Macrophage | Innate |
| CCL14 | Macrophage | Innate |
| CCL23 | Macrophage | Innate |
| CCL26 | Macrophage | Innate |
| CD300LB | Macrophage | Innate |
| CNR1 | Macrophage | Innate |
| CNR2 | Macrophage | Innate |
| EIF1 | Macrophage | Innate |
| EIF4A1 | Macrophage | Innate |
| FPR1 | Macrophage | Innate |
| FPR2 | Macrophage | Innate |
| FRAT2 | Macrophage | Innate |
| GPR27 | Macrophage | Innate |
| GPR77 | Macrophage | Innate |
| RNASE2 | Macrophage | Innate |
| MS4A2 | Macrophage | Innate |
| BASP1 | Macrophage | Innate |
| IGSF6 | Macrophage | Innate |
| HK3 | Macrophage | Innate |
| VNN1 | Macrophage | Innate |
| FES | Macrophage | Innate |
| NPL | Macrophage | Innate |
| FZD2 | Macrophage | Innate |
| FAM198B | Macrophage | Innate |
| HNMT | Macrophage | Innate |
| SLC15A3 | Macrophage | Innate |
| CD4 | Macrophage | Innate |
| TXNDC3 | Macrophage | Innate |
| FRMD4A | Macrophage | Innate |
| CRYBB1 | Macrophage | Innate |
| HRH1 | Macrophage | Innate |
| WNT5B | Macrophage | Innate |
| ADAMTS3 | Mast cell | Innate |
| CPA3 | Mast cell | Innate |
| CMA1 | Mast cell | Innate |
| CTSG | Mast cell | Innate |
| ARHGAP15 | Mast cell | Innate |
| CPM | Mast cell | Innate |
| FCN1 | Mast cell | Innate |
| FTL | Mast cell | Innate |
| HSPA6 | Mast cell | Innate |
| ITGA9 | Mast cell | Innate |
| RNASE3 | Mast cell | Innate |
| S100A4 | Mast cell | Innate |
| SIGLEC8 | Mast cell | Innate |
| SLC6A4 | Mast cell | Innate |
| PTGS2 | Mast cell | Innate |
| EGR3 | Mast cell | Innate |
| PILRA | Mast cell | Innate |
| CCR2 | MDSC | Innate |
| CD14 | MDSC | Innate |
| CD2 | MDSC | Innate |
| CD86 | MDSC | Innate |
| CXCR4 | MDSC | Innate |
| FCGR2A | MDSC | Innate |
| FCGR2B | MDSC | Innate |
| FCGR3A | MDSC | Innate |
| FERMT3 | MDSC | Innate |
| GPSM3 | MDSC | Innate |
| IL18BP | MDSC | Innate |
| IL4R | MDSC | Innate |
| ITGAL | MDSC | Innate |
| ITGAM | MDSC | Innate |
| PARVG | MDSC | Innate |
| PSAP | MDSC | Innate |
| PTGER2 | MDSC | Innate |
| PTGES2 | MDSC | Innate |
| S100A8 | MDSC | Innate |
| S100A9 | MDSC | Innate |
| ASGR2 | Monocyte | Innate |
| CFP | Monocyte | Innate |
| ASGR1 | Monocyte | Innate |
| CD1D | Monocyte | Innate |
| UPK3A | Monocyte | Innate |
| ACTG1 | Monocyte | Innate |
| ANXA5 | Monocyte | Innate |
| ATP6V1B2 | Monocyte | Innate |
| CFL1 | Monocyte | Innate |
| DAZAP2 | Monocyte | Innate |
| CTBS | Monocyte | Innate |
| EMR4P | Monocyte | Innate |
| HIVEP2 | Monocyte | Innate |
| MARCKSL1 | Monocyte | Innate |
| MBP | Monocyte | Innate |
| MMP15 | Monocyte | Innate |
| PNPLA6 | Monocyte | Innate |
| TMBIM6 | Monocyte | Innate |
| PQBP1 | Monocyte | Innate |
| TEX264 | Monocyte | Innate |
| IKZF1 | Monocyte | Innate |
| AKT3 | Natural killer cell | Innate |
| AXL | Natural killer cell | Innate |
| BST2 | Natural killer cell | Innate |
| CDH2 | Natural killer cell | Innate |
| CRTAM | Natural killer cell | Innate |
| CSF2RA | Natural killer cell | Innate |
| CTSZ | Natural killer cell | Innate |
| CXCL1 | Natural killer cell | Innate |
| CYTH1 | Natural killer cell | Innate |
| DAXX | Natural killer cell | Innate |
| DGKH | Natural killer cell | Innate |
| DLL4 | Natural killer cell | Innate |
| DPYD | Natural killer cell | Innate |
| ERBB3 | Natural killer cell | Innate |
| F11R | Natural killer cell | Innate |
| FAM27A | Natural killer cell | Innate |
| FAM49A | Natural killer cell | Innate |
| FASLG | Natural killer cell | Innate |
| FCGR1A | Natural killer cell | Innate |
| FN1 | Natural killer cell | Innate |
| FSTL1 | Natural killer cell | Innate |
| FUCA1 | Natural killer cell | Innate |
| GBP3 | Natural killer cell | Innate |
| GLS2 | Natural killer cell | Innate |
| GRB2 | Natural killer cell | Innate |
| LST1 | Natural killer cell | Innate |
| BCL2 | Natural killer cell | Innate |
| CDC5L | Natural killer cell | Innate |
| FGF18 | Natural killer cell | Innate |
| FUT5 | Natural killer cell | Innate |
| FZR1 | Natural killer cell | Innate |
| GAGE2 | Natural killer cell | Innate |
| IGFBP5 | Natural killer cell | Innate |
| KANK2 | Natural killer cell | Innate |
| LDB3 | Natural killer cell | Innate |
| BTN2A2 | Natural killer T cell | Innate |
| CD101 | Natural killer T cell | Innate |
| CD109 | Natural killer T cell | Innate |
| CNPY3 | Natural killer T cell | Innate |
| CNPY4 | Natural killer T cell | Innate |
| CREB1 | Natural killer T cell | Innate |
| CRTC2 | Natural killer T cell | Innate |
| CRTC3 | Natural killer T cell | Innate |
| CSF2 | Natural killer T cell | Innate |
| KLRC1 | Natural killer T cell | Innate |
| FUT4 | Natural killer T cell | Innate |
| ICAM2 | Natural killer T cell | Innate |
| IL32 | Natural killer T cell | Innate |
| LAMP2 | Natural killer T cell | Innate |
| LILRB5 | Natural killer T cell | Innate |
| KLRG1 | Natural killer T cell | Innate |
| HSPA4 | Natural killer T cell | Innate |
| HSPB6 | Natural killer T cell | Innate |
| ISM2 | Natural killer T cell | Innate |
| ITIH2 | Natural killer T cell | Innate |
| KDM4C | Natural killer T cell | Innate |
| KIR2DS4 | Natural killer T cell | Innate |
| KIRREL3 | Natural killer T cell | Innate |
| SDCBP | Natural killer T cell | Innate |
| NFATC2IP | Natural killer T cell | Innate |
| MICB | Natural killer T cell | Innate |
| KIR2DL1 | Natural killer T cell | Innate |
| KIR2DL3 | Natural killer T cell | Innate |
| KIR3DL1 | Natural killer T cell | Innate |
| KIR3DL2 | Natural killer T cell | Innate |
| NCR1 | Natural killer T cell | Innate |
| FOSL1 | Natural killer T cell | Innate |
| TSLP | Natural killer T cell | Innate |
| SLC7A7 | Natural killer T cell | Innate |
| SPP1 | Natural killer T cell | Innate |
| TREM2 | Natural killer T cell | Innate |
| UBASH3A | Natural killer T cell | Innate |
| YBX2 | Natural killer T cell | Innate |
| CCDC88A | Natural killer T cell | Innate |
| CLEC1A | Natural killer T cell | Innate |
| THBD | Natural killer T cell | Innate |
| PDPN | Natural killer T cell | Innate |
| VCAM1 | Natural killer T cell | Innate |
| EMR1 | Natural killer T cell | Innate |
| CREB5 | Neutrophil | Innate |
| CDA | Neutrophil | Innate |
| CHST15 | Neutrophil | Innate |
| S100A12 | Neutrophil | Innate |
| APOBEC3A | Neutrophil | Innate |
| CASP5 | Neutrophil | Innate |
| MMP25 | Neutrophil | Innate |
| HAL | Neutrophil | Innate |
| C1orf183 | Neutrophil | Innate |
| FFAR2 | Neutrophil | Innate |
| MAK | Neutrophil | Innate |
| CXCR1 | Neutrophil | Innate |
| STEAP4 | Neutrophil | Innate |
| MGAM | Neutrophil | Innate |
| BTNL8 | Neutrophil | Innate |
| CXCR2 | Neutrophil | Innate |
| TNFRSF10C | Neutrophil | Innate |
| VNN3 | Neutrophil | Innate |
| CBX6 | Plasmacytoid dendritic cell | Innate |
| DAB2 | Plasmacytoid dendritic cell | Innate |
| DDX17 | Plasmacytoid dendritic cell | Innate |
| HIGD1A | Plasmacytoid dendritic cell | Innate |
| IDH3A | Plasmacytoid dendritic cell | Innate |
| IL3RA | Plasmacytoid dendritic cell | Innate |
| MAGED1 | Plasmacytoid dendritic cell | Innate |
| NUCB2 | Plasmacytoid dendritic cell | Innate |
| OFD1 | Plasmacytoid dendritic cell | Innate |
| OGT | Plasmacytoid dendritic cell | Innate |
| PDIA4 | Plasmacytoid dendritic cell | Innate |
| SERTAD2 | Plasmacytoid dendritic cell | Innate |
| SIRPA | Plasmacytoid dendritic cell | Innate |
| TMED2 | Plasmacytoid dendritic cell | Innate |
| ENG | Plasmacytoid dendritic cell | Innate |
| FCAR | Plasmacytoid dendritic cell | Innate |
| IGF1 | Plasmacytoid dendritic cell | Innate |
| ITGA2B | Plasmacytoid dendritic cell | Innate |
| GABARAP | Plasmacytoid dendritic cell | Innate |
| GPX1 | Plasmacytoid dendritic cell | Innate |
| KRT23 | Plasmacytoid dendritic cell | Innate |
| PROK2 | Plasmacytoid dendritic cell | Innate |
| RALB | Plasmacytoid dendritic cell | Innate |
| RETNLB | Plasmacytoid dendritic cell | Innate |
| RNF141 | Plasmacytoid dendritic cell | Innate |
| SEC14L1 | Plasmacytoid dendritic cell | Innate |
| SEPX1 | Plasmacytoid dendritic cell | Innate |
| EMP3 | Plasmacytoid dendritic cell | Innate |
| CD300LF | Plasmacytoid dendritic cell | Innate |
| ABTB1 | Plasmacytoid dendritic cell | Innate |
| KLHL21 | Plasmacytoid dendritic cell | Innate |
| PHRF1 | Plasmacytoid dendritic cell | Innate |
