# Supplementary material for: Identification and validation of a 7-genes prognostic signature for adult acute myeloid leukemia based on aging-related genes
Source: Aging (Albany NY). 2023 Jun 26;15(12):5826–53. doi: 10.18632/aging.204843 (PMC10333094; doi:10.18632/aging.204843)
Supplement: Supplementary Table 5 [file aging-15-204843-s006.docx]

| **Supplementary Table 5. Single-sample gene-set for tumor immune microenvironment analysis.** | | |
| --- | --- | --- |
| Activated_CD8_T_cell | NA | ADRM1/AHSA1/C1GALT1C1/CCT6B/CD37/CD3D/CD3E/CD3G/CD69/CD8A/CETN3/CSE1L/GEMIN6/GNLY/GPT2/GZMA/GZMH/GZMK/IL2RB/LCK/MPZL1/NKG7/PIK3IP1/PTRH2/TIMM13/ZAP70 |
| Central_memory_CD8_T_cell | NA | ACTN4/ADAM12/ADCY9/F13A1/FCER1G/FCGR3B/FGF7/FKBP4/GLUD1/GM2A/GUSB/IL1RN/NOL11/NTRK1/RARA/RNF128/SIGLEC1/TNFRSF11A/TOX4/UBA52/ULBP1 |
| Effector_memeory_CD8_T_cell | NA | ACAP1/APOL3/ARHGAP10/ATP10D/C3AR1/CCR5/CD160/CD55/CFLAR/CMKLR1/DAPP1/FCRL6/FLT3LG/GZMM/HAPLN3/HLA-DMB/HLA-DPA1/HLA-DPB1/IFI16/LIME1/LTK/NFKBIA/SETD7/SIK1/TRIB2 |
| Activated_CD4_T_cell | NA | AIM2/BIRC3/BRIP1/CCL20/CCL4/CCL5/CCNB1/CCR7/DUSP2/ESCO2/ETS1/EXO1/EXOC6/IARS/ITK/KIF11/KNTC1/NUF2/PRC1/PSAT1/RGS1/RTKN2/SAMSN1/SELL/TRAT1 |
| Central_memory_CD4_T_cell | NA | ABHD3/AHNAK/ANXA2P2/AQP3/ATHL1/BMI1/BZW2/CD63/COL4A1/CYLD/ELMO2/FYN/GLIPR1/GSS/IFITM2/ITGB1/ITGB2/KLF5/LSP1/NDUFB9/PKM2/SFXN3/SIRPG/SMAD4/STX4/TRADD/VIM/XRCC6 |
| Effector_memeory_CD4_T_cell | NA | ATM/CASP3/CASQ1/CD300E/DARS/DOCK9/EXOSC9/EZH2/GDE1/IL34/NCOA4/NEFL/PDGFRL/PTGS1/REPS1/SCG2/SDPR/SIGLEC14/SIGLEC6/TAL1/TFEC/TIPIN/TPK1/UQCRB/USP9Y/WIPF1/ZCRB1 |
| T_follicular_helper_cell | NA | B3GAT1/CDK5R1/PDCD1/BCL6/CD200/CD83/CD84/FGF2/GPR18/CEBPA/CECR1/CLEC10A/CLEC4A/CSF1R/CTSS/DMN/DPP4/LRRC32/MC5R/MICA/NCAM1/NCR2/NRP1/PDCD1LG2/PDCD6/PRDX1/RAE1/RAET1E/SIGLEC7/SIGLEC9/TYRO3/CHST12/CLIC3/IVNS1ABP/KIR2DL2/LGMN |
| Gamma_delta_T_cell | NA | ACP5/AQP9/BTN3A2/C1orf54/CARD8/CCL18/CD209/CD33/CD36/CDK5/IL10RB/KLRF1/LGALS1/MAPK7/KLHL7/KRT80/LAMC1/LCORL/LMNB1/MEIS3P1/MPL/FABP1/FABP5/FADD/MFAP3L/MINPP1/RPS24/RPS7/RPS9/DBNL/CCL13 |
| Type_1_T_helper_cell | NA | CD70/TBX21/ADAM8/AHCYL2/ALCAM/B3GALNT1/BBS12/BST1/CD151/CD47/CD48/CD52/CD53/CD59/CD6/CD68/CD7/CD96/CFHR3/CHRM3/CLEC7A/COL23A1/COL4A4/COL5A3/DAB1/DLEU7/DOC2B/EMP1/F12/FURIN/GAB3/GATM/GFPT2/GPR25/GREM2/HAVCR1/HSD11B1/HUNK/IGF2/RCSD1/RYR1/SAV1/SELE/SELP/SH3KBP1/SIT1/SLC35B3/SIGLEC10/SKAP1/THUMPD2/TIGIT/ZEB2/ENC1/FAM134B/FBXO30/FCGR2C/STAC/LTC4S/MAN1B1/MDH1/MMD/RGS16/IL12A/P2RX5/CD97/ITGB4/ICAM3/METRNL/TNFRSF1A/IRF1/HTR2B/CALD1/MOCOS/TRAF3IP2/TLR8/TRAF1/DUSP14 |
| Type_17_T_helper_cell | NA | IL17A/IL17RA/C2CD4A/C2CD4B/CA2/CCDC65/CEACAM3/IL17C/IL17F/IL17RC/IL17RE/IL23A/ILDR1/LONRF3/SH2D6/TNIP2/ABCA1/ABCB1/ADAMTS12/ANK1/ANKRD22/B3GALT2/CAMTA1/CCR9/CD40/GPR44/IFT80 |
| Type_2_T_helper_cell | NA | ASB2/CSRP2/DAPK1/DLC1/DNAJC12/DUSP6/GNAI1/LAMP3/NRP2/OSBPL1A/PDE4B/PHLDA1/PLA2G4A/RAB27B/RBMS3/RNF125/TMPRSS3/GATA3/BIRC5/CDC25C/CDC7/CENPF/CXCR6/DHFR/EVI5/GSTA4/HELLS/IL26/LAIR2 |
| Regulatory_T_cell | NA | CCL3L1/CD72/CLEC5A/FOXP3/ITGA4/L1CAM/LIPA/LRP1/LRRC42/MARCO/MMP12/MNDA/MRC1/MS4A6A/PELO/PLEK/PRSS23/PTGIR/ST8SIA4/STAB1 |
| Activated_B_cell | NA | ADAM28/CD180/CD79B/BLK/CD19/MS4A1/TNFRSF17/IGHM/GNG7/MICAL3/SPIB/HLA-DOB/IGKC/PNOC/FCRL2/BACH2/CR2/TCL1A/AKNA/ARHGAP25/CCL21/CD27/CD38/CLEC17A/CLEC9A/CLECL1 |
| Immature__B_cell | NA | CD22/CYBB/FAM129C/FCRL1/FCRL3/FCRL5/FCRLA/HDAC9/HLA-DQA1/HVCN1/KIAA0226/NCF1/NCF1B/P2RY10/SP100/TXNIP/STAP1/TAGAP/ZCCHC2 |
| Memory_B_cell | NA | AICDA/CCNA2/CDKN3/CLCN5/ENPP1/FCER1A/FCRL4/MYC/RUNX2/SORL1/SOX5/STAT5A/STAT5B/TLR9 |
| Natural_killer_cell | NA | AKT3/AXL/BST2/CDH2/CRTAM/CSF2RA/CTSZ/CXCL1/CYTH1/DAXX/DGKH/DLL4/DPYD/ERBB3/F11R/FAM27A/FAM49A/FASLG/FCGR1A/FN1/FSTL1/FUCA1/GBP3/GLS2/GRB2/LST1/BCL2/CDC5L/FGF18/FUT5/FZR1/GAGE2/IGFBP5/KANK2/LDB3 |
| CD56bright_natural_killer_cell | NA | ABAT/C11orf75/C5orf15/CDHR1/DCAF12/DYNLL1/GPR137B/HCP5/HDGFRP2/KRT86/MLST8/ELMOD3/ENTPD5/FAM119A/FAM179A/CLIC2/COX7A2L/CREB3L4/CSF1/CSNK2A2/CSTA/CSTB/CTPS/CTSD/FST/GATA2/GMPR/HDC/HEY1/HOXA1/HS2ST1/HS3ST1/BCL11B/CDH3/MYL6B/NAA16/ClQA/ClQB/CYP27B1/EIF3M |
| CD56dim_natural_killer_cell | NA | CYP27A1/DDX55/DYRK2/RPL37A/NOTCH3/AKR7A3/GPRC5C/GRIN1/HLA-E/PORCN/PSMC4/UPP1/IL21R/KIR2DS1/KIR2DS2/KIR2DS5 |
| Myeloid_derived_suppressor_cell | NA | CCR2/CD14/CD2/CD86/CXCR4/FCGR2A/FCGR2B/FCGR3A/FERMT3/GPSM3/IL18BP/IL4R/ITGAL/ITGAM/PARVG/PSAP/PTGER2/PTGES2/S100A8/S100A9 |
| Natural_killer_T_cell | NA | BTN2A2/CD101/CD109/CNPY3/CNPY4/CREB1/CRTC2/CRTC3/CSF2/KLRC1/FUT4/ICAM2/IL32/LAMP2/LILRB5/KLRG1/HSPA4/HSPB6/ISM2/ITIH2/KDM4C/KIR2DS4/KI-REL3/SDCBP/NFATC2IP/MICB/KIR2DL1/KIR2DL3/KIR3DL1/KIR3DL2/NCR1/FOSL1/TSLP/SLC7A7/SPP1/TREM2/UBASH3A/YBX2/CCDC88A/CLEC1A/THBD/PDPN/VCAM1/EMR1 |
| Activated_dendritic_cell | NA | ABCD1/C1QC/CAPG/CCL3L3/CD207/CD302/ATP5B/ATP5L/ATP6V1A/BCL2L1/C1QB/SNURF/SPCS3/CCNA1/CEACAM8/NOS2/SRA1/TNFRSF6B/TREM1/TREML1/RHOA/SLC25A37/TNFSF14/TREML4/VNN2/XPO6/CLEC4C/TNFAIP2/UBD/ACTR3/RAB1A/SLA/HLA-DQA2/SIGLEC5/SLAMF9 |
| Plasmacytoid_dendritic_cell | NA | CBX6/DAB2/DDX17/HIGD1A/IDH3A/IL3RA/MAGED1/NUCB2/OFD1/OGT/PDIA4/SERTAD2/SIRPA/TMED2/ENG/FCAR/IGF1/ITGA2B/GABARAP/GPX1/KRT23/PROK2/RALB/RETNLB/RNF141/SEC14L1/SEPX1/EMP3/CD300LF/ABTB1/KLHL21/PHRF1 |
| Immature_dendritic_cell | NA | ACADM/AHCYL1/ALDH1A2/ALDH3A2/ALDH9A1/ALOX15/AMT/ARL1/ATIC/ATP5A1/CAPZA1/LILRA5/RDX/RRAGD/TACSTD2/INPP5F/RAB38/PLAU/CSF3R/SLC18A2/AMPD2/CLTB/C1orf162/ |
| Macrophage | NA | AIF1/CCL1/CCL14/CCL23/CCL26/CD300LB/CNR1/CNR2/EIF1/EIF4A1/FPR1/FPR2/FRAT2/GPR27/GPR77/RNASE2/MS4A2/BASP1/IGSF6/HK3/VNN1/FES/NPL/FZD2/FAM198B/HNMT/SLC15A3/CD4/TXNDC3/FRMD4A/CRYBB1/HRH1/WNT5B |
| Eosinophil | NA | GIPR/KRT18P50/LRMP/FOSB/RRP12/GPR183/NR4A3/ST3GAL6/DEPDC5/PDE6C/PKD2L2/GPR65/IL5RA/P2RY14/DACH1/DAPK2/EMR3 |
| Mast_cell | NA | ADAMTS3/CPA3/CMA1/CTSG/ARHGAP15/CPM/FCN1/FTL/HSPA6/ITGA9/RNASE3/S100A4/SIGLEC8/SLC6A4/PTGS2/EGR3/PILRA |
| Monocyte | NA | ASGR2/CFP/ASGR1/CD1D/UPK3A/ACTG1/ANXA5/ATP6V1B2/CFL1/DAZAP2/CTBS/EMR4P/HIVEP2/MARCKSL1/MBP/MMP15/PNPLA6/TMBIM6/PQBP1/TEX264/IKZF1 |
| Neutrophil | NA | CREB5/CDA/CHST15/S100A12/APOBEC3A/CASP5/MMP25/HAL/C1orf183/FFAR2/MAK/CXCR1/STEAP4/MGAM/BTNL8/CXCR2/TNFRSF10C/VNN3 |
| APC_co_inhibition | NA | C10orf54/CD274/LGALS9/PDCD1LG2/PVRL3 |
| APC_co_stimulation | NA | CD40/CD58/CD70/ICOSLG/SLAMF1/TNFSF14/TNFSF15/TNFSF18/TNFSF4/TNFSF8/TNFSF9 |
| CCR | NA | CCL16/TPO/TGFBR2/CXCL2/CCL14/TGFBR3/IL11RA/CCL11/IL4I1/IL33/CXCL12/CXCL10/BMPER/BMP8A/CXCL11/IL21R/IL17B/TNFRSF9/ILF2/CX3CR1/CCR8/TNFSF12/CSF3/TNFSF4/BMP3/CX3CL1/BMP5/CXCR2/TNFRSF10D/BMP2/CXCL14/CCL28/CXCL3/BMP6/CCL21/CXCL9/CCL23/IL6/TNFRSF18/IL17RD/IL17D/IL27/CCL7/IL1R1/CXCR4/CXCR2P1/TGFB1I1/IFNGR1/IL9R/IL1RAPL1/IL11/CSF1/IL20RA/IL25/TNFRSF4/IL18/ILF3/CCL20/TNFRSF12A/IL6ST/CXCL13/IL12B/TNFRSF8/IL6R/BMPR2/IFNE/IL1RAPL2/IL3RA/BMP4/CCL24/TNFSF13B/CCR4/IL2RA/IL32/TNFRSF10C/IL22RA1/BMPR1A/CXCR5/CXCR3/IFNA8/IL17REL/IFNB1/IFNAR1/TNFRSF1B/CCL17/IFNL1/IL16/IL1RL1/ILK/CCL25/ILDR2/CXCR1/IL36RN/IL34/TGFB1/IFNG/IL19/ILKAP/BMP2K/CCR10/ILDR1/EPO/CCR7/IL17C/IL23A/CCR5/IL7/EPOR/CCL13/IL2RG/IL31RA/TNFAIP6/IFNL2/BMP1/IL12RB1/TNFAIP8/IL4R/TNFRSF6B/TNFAIP8L1/TNFRSF10B/IFNL3/CCL5/CXCL6/CXCL1/CCR3/TNFSF11/CSF1R/IL21/IL1RAP/IL12RB2/CCL1/IL17RA/CCR1/IL1RN/TNFRSF11B/TNFRSF14/IL13/IL2RB/BMP8B/CCL2/IL24/IL18RAP/TGFBI/TNFSF10/TNFRSF11A/CXCL5/IL5RA/TNFSF9/IL1RL2/TNFRSF13C/IL36G/IL15RA/TNFRSF21/CXCL8/IL22RA2/TNFAIP8L2/IL18R1/IFNLR1/CXCR6/CCL3L3/TNFRSF1A/IL17RE/IFNGR2/IL17RC/TNFAIP8L3/ILVBL/TGFBRAP1/CCL4L1/CSF2RA/CCRN4L/CCL26/TNFAIP1/CCRL2/IFNA10/TNFRSF17/IFNA13/IL20/IL18BP/CCL3L1/TNFSF12-TNFSF13/IL5/IL23R/IL26/TNF/TGFA/CSF2/IL1F10/CXCL17/TNFSF13/IFNA4/IL37/IL12A/IL7R/IFNA1/IL1A/IL4/IL2/CCL22/CSF3R/IL10/IFNK/TGFB2/IL1R2/IL1B/IL17F/IL27RA/IL15/TNFSF8/IL36B/XCL1/CXCL16/TNFRSF19/IL3/CCL3/IFNA2/BMPR1B/IFNA21/TNFSF18/CCL8/IL17RB/TNFRSF25/IL22/IL10RB/IFNAR2/CCL18/IFNA16/CSF2RB/IL36A/TNFAIP3/IL13RA2/IL13RA1/CCR9/TNFRSF10A/IFNA7/IFNW1/XCL2/TNFSF14/CCR2/BMP15/BMP10/CCL15-CCL14/TGFBR1/IFNA5/BMP7/IFNA14/IL20RB/IL10RA/IFNA17/CCR6/TGFB3/CCL15/CCL4/CCL27/TNFRSF13B/TNFAIP2/IL31/IL17A/TNFSF15/CCL19/IFNA6/IL9 |
| Check-point | NA | IDO1/LAG3/CTLA4/TNFRSF9/ICOS/CD80/PDCD1LG2/TIGIT/CD70/TNFSF9/ICOSLG/KIR3DL1/CD86/PDCD1/LAIR1/TNFRSF8/TNFSF15/TNFRSF14/IDO2/CD276/CD40/TNFRSF4/TNFSF14/HHLA2/CD244/CD274/HAVCR2/CD27/BTLA/LGALS9/TMIGD2/CD28/CD48/TNFRSF25/CD40LG/ADORA2A/VTCN1/CD160/CD44/TNFSF18/TNFRSF18/BTNL2/C10orf54/CD200R1/TNFSF4/CD200/NRP1 |
| Cytolytic_activity | NA | PRF1/GZMA |
| HLA | NA | HLA-E/HLA-DPB2/HLA-C/HLA-J/HLA-DQB1/HLA-DQB2/HLA-DQA2/HLA-DQA1/HLA-A/HLA-DMA/HLA-DOB/HLA-DRB1/HLA-H/HLA-B/HLA-DRB5/HLA-DOA/HLA-DPB1/HLA-DRA/HLA-DRB6/HLA-L/HLA-F/HLA-G/HLA-DMB/HLA-DPA1 |
| Inflammation-promoting | NA | CCL5/CD19/CD8B/CXCL10/CXCL13/CXCL9/GNLY/GZMB/IFNG/IL12A/IL12B/IRF1/PRF1/STAT1/TBX21 |
| MHC_class_I | NA | B2M/HLA-A/TAP1 |
| Parainflammation | NA | CXCL10/PLAT/CCND1/LGMN/PLAUR/AIM2/MMP7/ICAM1/MX2/CXCL9/ANXA1/TLR2/PLA2G2D/ITGA2/MX1/HMOX1/CD276/TIRAP/IL33/PTGES/TNFRSF12A/SCARB1/CD14/BLNK/IFIT3/RETNLB/IFIT2/ISG15/OAS2/REL/OAS3/CD44/PPARG/BST2/OAS1/NOX1/PLA2G2A/IFIT1/IFITM3/IL1RN |
| T_cell_co-inhibition | NA | BTLA/C10orf54/CD160/CD244/CD274/CTLA4/HAVCR2/LAG3/LAIR1/TIGIT |
| T_cell_co-stimulation | NA | CD2/CD226/CD27/CD28/CD40LG/ICOS/SLAMF1/TNFRSF18/TNFRSF25/TNFRSF4/TNFRSF8/TNFRSF9/TNFSF14/ |
| Type_I_IFN_Reponse | NA | DDX4/IFIT1/IFIT2/IFIT3/IRF7/ISG20/MX1/MX2/RSAD2/TNFSF10 |
| Type_II_IFN_Reponse | NA | GPR146/SELP/AHR |
