# Supplementary material for: Identification and validation of a 7-genes prognostic signature for adult acute myeloid leukemia based on aging-related genes
Source: Aging (Albany NY). 2023 Jun 26;15(12):5826–53. doi: 10.18632/aging.204843 (PMC10333094; doi:10.18632/aging.204843)
Supplement: Supplementary Table 6 [file aging-15-204843-s007.docx]

**Supplementary Table 6. Twelve aging-related gene sets.**

| **Symbol** | **Description** | **Function** | **Gene Set** | **KEGG Name** |
| --- | --- | --- | --- | --- |
|  |  |  |  |  |
| IL2RB | Interleukin 2 Receptor Subunit Beta | Receptor for interleukin-2. This beta subunit is involved in receptor mediated endocytosis and transduces the mitogenic signals of IL2. Probably in association with IL15RA, involved in the stimulation of neutrophil phagocytosis by IL15. | others | Cytokine-cytokine receptor interaction |
| PSAT1 | Phosphoserine Aminotransferase 1 | Catalyzes the reversible conversion of 3-phosphohydroxypyruvate to phosphoserine and of 3-hydroxy-2-oxo-4-phosphonooxybutanoate to phosphohydroxythreonine. | others | Glycine, serine and threonine metabolism |
| A2M | alpha-2-macroglobulin | Alpha-2-macroglobulin is a serum pan-protease inhibitor that has been implicated in Alzheimer disease (AD) based on its ability to mediate the clearance and degradation of A beta, the major component of beta-amyloid deposits. | others | Unknown |
| AARS1 | Alanyl-TRNA Synthetase 1 | Catalyzes the attachment of alanine to tRNA (Ala) in a two-step reaction: alanine is first activated by ATP to form Ala-AMP and then transferred to the acceptor end of tRNA (Ala) (PubMed:27622773, PubMed:27911835, PubMed:28493438). Also edits incorrectly charged tRNA (Ala) via its editing domain. | loss of proteostasis | Aminoacyl-tRNA biosynthesis |
| ABL1 | ABL proto-oncogene 1, non-receptor tyrosine kinase | c-Abl stimulates p73-mediated transactivation and apoptosis. This regulation of p73 by c-Abl in response to DNA damage is also demonstrated by a failure of ionizing-radiation-induced apoptosis after disruption of the c-Abl-p73 interaction. | genomic instability | Cell cycle |
| ADCY1 | Adenylate cyclase type 1 | Catalyzes the formation of the signaling molecule cAMP in response to G-protein signaling. Mediates responses to increased cellular Ca2+/calmodulin levels. May be involved in regulatory processes in the central nervous system. May play a role in memory and learning. Plays a role in the regulation of the circadian rhythm of daytime contrast sensitivity probably by modulating the rhythmic synthesis of cyclic AMP in the retina. | altered intercellular communication | Longevity regulating pathway |
| ADCY2 | Adenylate cyclase type 2 | Catalyzes the formation of the signaling molecule cAMP in response to G-protein signaling. Down-stream signaling cascades mediate changes in gene expression patterns and lead to increased IL6 production. Functions in signaling cascades downstream of the muscarinic acetylcholine receptors. | altered intercellular communication | Longevity regulating pathway |
| ADCY3 | Adenylate cyclase type 3 | Catalyzes the formation of the signaling molecule cAMP in response to G-protein signaling. Participates in signaling cascades triggered by odorant receptors via its function in cAMP biosynthesis. Required for the perception of odorants. Required for normal sperm motility and normal male fertility. Plays a role in regulating insulin levels and body fat accumulation in response to a high fat diet. | altered intercellular communication | Longevity regulating pathway |
| ADCY4 | Adenylate cyclase type 4 | Catalyzes the formation of the signaling molecule cAMP in response to G-protein signaling. | altered intercellular communication | Longevity regulating pathway |
| ADCY5 | adenylate cyclase 5 | Catalyzes the formation of the signaling molecule cAMP in response to G-protein signaling. Mediates signaling downstream of ADRB1. Regulates the increase of free cytosolic Ca2+ in response to increased blood glucose levels and contributes to the regulation of Ca2+-dependent insulin secretion. | altered intercellular communication | Longevity regulating pathway |
| ADCY6 | Adenylate cyclase type 6 | Catalyzes the formation of the signaling molecule cAMP downstream of G protein-coupled receptors. Functions in signaling cascades downstream of beta-adrenergic receptors in the heart and in vascular smooth muscle cells. Functions in signaling cascades downstream of the vasopressin receptor in the kidney and has a role in renal water reabsorption. Functions in signaling cascades downstream of PTH1R and plays a role in regulating renal phosphate excretion. Functions in signaling cascades downstream of the VIP and SCT receptors in pancreas and contributes to the regulation of pancreatic amylase and fluid secretion. Signaling mediates cAMP-dependent activation of protein kinase PKA. This promotes increased phosphorylation of various proteins, including AKT. Plays a role in regulating cardiac sarcoplasmic reticulum Ca2+ uptake and storage, and is required for normal heart ventricular contractibility. | altered intercellular communication | Longevity regulating pathway |
| ADCY7 | Adenylate cyclase type 7 | Catalyzes the formation of cAMP in response to activation of G protein-coupled receptors. Functions in signaling cascades activated namely by thrombin and sphingosine 1-phosphate and mediates regulation of cAMP synthesis through synergistic action of the stimulatory G alpha protein with GNA13. Also, during inflammation, mediates zymosan-induced increase intracellular cAMP, leading to protein kinase A pathway activation in order to modulate innate immune responses through heterotrimeric G proteins G (12/13). Functions in signaling cascades activated namely by dopamine and C5 alpha chain and mediates regulation of cAMP synthesis through synergistic action of the stimulatory G protein with G beta: gamma complex. | altered intercellular communication | Longevity regulating pathway |
| ADCY8 | Adenylate cyclase type 8 | Catalyzes the formation of cAMP in response to calcium entry leadings to cAMP signaling activation that affect processes suche as synaptic plasticity and insulin secretion. Plays a role in many brain functions, such as learning, memory, drug addiction, and anxiety modulation through regulation of synaptic plasticity by modulating long-term memory and long-term potentiation (LTP) through CREB transcription factor activity modulation. Plays a central role in insulin secretion by controlling glucose homeostasis through glucagon-like peptide 1 and glucose signaling pathway and maintains insulin secretion through calcium-dependent PKA activation leading to vesicle pool replenishment. Also, allows PTGER3 to induce potentiation of PTGER4-mediated PLA2 secretion by switching from a negative to a positive regulation, during the IL1B induced-dedifferentiation of smooth muscle cells. | altered intercellular communication | Longevity regulating pathway |
| ADCY9 | Adenylate cyclase type 9 | Adenylyl cyclase that catalyzes the formation of the signaling molecule cAMP in response to activation of G protein-coupled receptors. Contributes to signaling cascades activated by CRH (corticotropin-releasing factor), corticosteroids and beta-adrenergic receptors. | altered intercellular communication | Longevity regulating pathway |
| ADH1B | alcohol dehydrogenase 1B (class I), beta polypeptide | Catalyzes the NAD-dependent oxidation of all-trans-retinol and its derivatives such as all-trans-4-hydroxyretinol and may participate in retinoid metabolism. In vitro can also catalyzes the NADH-dependent reduction of all-trans-retinal and its derivatives such as all-trans-4-oxoretinal. | genomic instability | Unknown |
| ADH5 | alcohol dehydrogenase 5 (class III), chi polypeptide | Catalyzes the oxidation of long-chain primary alcohols and the oxidation of S-(hydroxymethyl) glutathione. Also oxidizes long chain omega-hydroxy fatty acids, such as 20-HETE, producing both the intermediate aldehyde, 20-oxoarachidonate and the end product, a dicarboxylic acid, (5Z,8Z,11Z,14Z)-eicosatetraenedioate. | others | Unknown |
| ADIPOQ | Adiponectin receptor protein 1 | Receptor for ADIPOQ, an essential hormone secreted by adipocytes that regulates glucose and lipid metabolism. Required for normal glucose and fat homeostasis and for maintaining a normal body weight. ADIPOQ-binding activates a signaling cascade that leads to increased AMPK activity, and ultimately to increased fatty acid oxidation, increased glucose uptake and decreased gluconeogenesis. | deregulated nutrient sensing | Longevity regulating pathway |
| ADIPOR1 | Adiponectin receptor protein 1 | Receptor for ADIPOQ, an essential hormone secreted by adipocytes that regulates glucose and lipid metabolism. Required for normal glucose and fat homeostasis and for maintaining a normal body weight. ADIPOQ-binding activates a signaling cascade that leads to increased AMPK activity, and ultimately to increased fatty acid oxidation, increased glucose uptake and decreased gluconeogenesis. | deregulated nutrient sensing | Longevity regulating pathway |
| ADIPOR2 | Adiponectin receptor protein 2 | Receptor for ADIPOQ, an essential hormone secreted by adipocytes that regulates glucose and lipid metabolism. Required for normal body fat and glucose homeostasis. ADIPOQ-binding activates a signaling cascade that leads to increased PPARA activity, and ultimately to increased fatty acid oxidation and glucose uptake. Has intermediate affinity for globular and full-length adiponectin. Required for normal revascularization after chronic ischemia caused by severing of blood vessels. | deregulated nutrient sensing | Longevity regulating pathway |
| AGPAT2 | 1-acylglycerol-3-phosphate O-acyltransferase 2 | This gene encodes a member of the 1-acylglycerol-3-phosphate O-acyltransferase family. The protein is located within the endoplasmic reticulum membrane and converts lysophosphatidic acid to phosphatidic acid, the second step in de novo phospholipid biosynthesis. Mutations in this gene have been associated with congenital generalized lipodystrophy (CGL), or Berardinelli-Seip syndrome, a disease characterized by a near absence of adipose tissue and severe insulin resistance. Alternate transcriptional splice variants, encoding different isoforms, have been characterized. | deregulated nutrient sensing | Unknown |
| AGTR1 | angiotensin II receptor, type 1 | Receptor for angiotensin II. Mediates its action by association with G proteins that activate a phosphatidylinositol-calcium second messenger system. | altered intercellular communication | Unknown |
| AIFM1 | apoptosis-inducing factor, mitochondrion-associated, 1 | AIFM1 is an important player in apoptosis. Like cytochrome c, AIF is localized to mitochondria and released in response to death stimuli. | mitochondrial dysfunction | Apoptosis |
| AKT1 | v-akt murine thymoma viral oncogene homolog 1 | AKT1 is one of 3 closely related serine/threonine-protein kinases (AKT1, AKT2 and AKT3) called the AKT kinase, and which regulate many processes including metabolism, proliferation, cell survival, growth and angiogenesis. | cellular senescence | Longevity regulating pathway |
| AKT1S1 | AKT1 substrate 1 | Subunit of mTORC1, which regulates cell growth and survival in response to nutrient and hormonal signals. Activated mTORC1 up-regulates protein synthesis by phosphorylating key regulators of mRNA translation and ribosome synthesis. mTORC1 phosphorylates EIF4EBP1 and releases it from inhibiting the elongation initiation factor 4E (eiF4E). mTORC1 phosphorylates and activates S6K1 at 'Thr-389', which then promotes protein synthesis by phosphorylating PDCD4 and targeting it for degradation. May also play a role in nerve growth factor-mediated neuroprotection. | deregulated nutrient sensing | Longevity regulating pathway |
| AKT2 | RAC-beta serine/threonine-protein kinase | AKT2 is one of 3 closely related serine/threonine-protein kinases (AKT1, AKT2 and AKT3) called the AKT kinase, and which regulate many processes including metabolism, proliferation, cell survival, growth and angiogenesis. AKT mediates insulin-stimulated protein synthesis by phosphorylating TSC2 at 'Ser-939' and 'Thr-1462', thereby activating mTORC1 signaling and leading to both phosphorylation of 4E-BP1 and in activation of RPS6KB1. AKT is involved in the phosphorylation of members of the FOXO factors (Forkhead family of transcription factors), leading to binding of 14-3-3 proteins and cytoplasmic localization. AKT phosphorylates 'Ser-454' on ATP citrate lyase (ACLY), thereby potentially regulating ACLY activity and fatty acid synthesis. AKT plays a role as key modulator of the AKT-mTOR signaling pathway controlling the tempo of the process of newborn neurons integration during adult neurogenesis, including correct neuron positioning, dendritic development and synapse formation. | deregulated nutrient sensing | Longevity regulating pathway |
| AKT3 | RAC-gamma serine/threonine-protein kinase | AKT3 is one of 3 closely related serine/threonine-protein kinases (AKT1, AKT2 and AKT3) called the AKT kinase, and which regulate many processes including metabolism, proliferation, cell survival, growth and angiogenesis. This is mediated through serine and/or threonine phosphorylation of a range of downstream substrates. AKT3 is the least studied AKT isoform. It plays an important role in brain development and is crucial for the viability of malignant glioma cells. AKT3 isoform may also be the key molecule in up-regulation and down-regulation of MMP13 via IL13. Required for the coordination of mitochondrial biogenesis with growth factor-induced increases in cellular energy demands. Down-regulation by RNA interference reduces the expression of the phosphorylated form of BAD, resulting in the induction of caspase-dependent apoptosis. | mitochondrial dysfunction | Longevity regulating pathway |
| ALDH2 | aldehyde dehydrogenase 2 family member | Reaction=an aldehyde + H2O + NAD (+) = a carboxylate + 2 H (+) + NADH. | others | Unknown |
| ALDH9A1 | aldehyde dehydrogenase 9 family member A1 | Converts gamma-trimethylaminobutyraldehyde into gamma-butyrobetaine with high efficiency (in vitro). Can catalyze the irreversible oxidation of a broad range of aldehydes to the corresponding acids in an NAD-dependent reaction, but with low efficiency. | others | Unknown |
| APEX1 | APEX nuclease (multifunctional DNA repair enzyme) 1 | APEX1 repairs oxidative DNA damage. | genomic instability | Unknown |
| APOC3 | apolipoprotein C-III | Polymorphisms in the human APOC3 gene and promoter have been associated with lipoprotein profile, cardiovascular health, insulin (INS) sensitivity, and longevity. | deregulated nutrient sensing | Unknown |
| APOE | apolipoprotein E | APOE activates a non-canonical MAP kinase cascade that enhances APP transcription and amyloid-β synthesis. Several polymorphisms in the APOE gene have been associated with Alzheimer’s disease. | others | Alzheimer disease |
| APP | amyloid beta (A4) precursor protein | APP is an important player in Alzheimer's disease. | others | Alzheimer disease |
| APPL1 | DCC-interacting protein 13-alpha | Multifunctional adapter protein that binds to various membrane receptors, nuclear factors and signaling proteins to regulate many processes, such as cell proliferation, immune response, endosomal trafficking and cell metabolism. Regulates signaling pathway leading to cell proliferation through interaction with RAB5A and subunits of the NuRD/MeCP1 complex. Functions as a positive regulator of innate immune response via activation of AKT1 signaling pathway by forming a complex with APPL1 and PIK3R1. Involved in trafficking of the TGFBR1 from the endosomes to the nucleus via microtubules in a TRAF6-dependent manner. Plays a role in cell metabolism by regulating adiponecting and insulin signaling pathways. Required for fibroblast migration through HGF cell signaling. | altered intercellular communication | Longevity regulating pathway |
| APTX | aprataxin | The APTX protein is involved in DNA repair, and it interacts with DNA repair pathways as well as with ageing-related genes. | genomic instability | Unknown |
| AR | androgen receptor | Androgen-AR plays key roles in the development of insulin and leptin resistance, which suggests that AR may contribute to certain age-related diseases such as type 2 diabetes and cardiovascular disease. | deregulated nutrient sensing | MAPK signaling pathway |
| AREG | amphiregulin | Ligand of the EGF receptor/EGFR. Autocrine growth factor as well as a mitogen for a broad range of target cells including astrocytes, Schwann cells and fibroblasts. | senescence-associated secretory phenotype | MAPK signaling pathway |
| ARHGAP1 | Rho GTPase activating protein 1 | GTPase activator for the Rho, Rac and Cdc42 proteins, converting them to the putatively inactive GDP-bound state. Cdc42 seems to be the preferred substrate. | others | Unknown |
| ARNTL | aryl hydrocarbon receptor nuclear translocator-like | Transcriptional activator which forms a core component of the circadian clock. The circadian clock, an internal time-keeping system, regulates various physiological processes through the generation of approximately 24 hours circadian rhythms in gene expression, which are translated into rhythms in metabolism and behavior. | others | Unknown |
| ATF2 | activating transcription factor 2 | TF2 is an important transcription factor involved in a variety of functions that may also be involved in oxidative stress response and cellular growth arrest and senescence. | cellular senescence | Longevity regulating pathway |
| ATF4 | Cyclic AMP-dependent transcription factor ATF-4 | Transcription factor that binds the cAMP response element (CRE) (consensus: 5'-GTGACGT[AC][AG]-3') and acts both as a regulator of normal metabolic and redox processes, and as a master transcription factor during the integrated stress response (ISR). Binds to asymmetric CRE's as a heterodimer and to palindromic CRE's as a homodimer. Core effector of the ISR, which is required for adaptation to various stress, such as endoplasmic reticulum (ER) stress, amino acid starvation, mitochondrial stress or oxidative stress. Protects cells against metabolic consequences of ER oxidation by promoting expression of genes linked to amino acid sufficiency and resistance to oxidative stress. Regulates the induction of DDIT3/CHOP and asparagine synthetase (ASNS) in response to amino acid deprivation or endoplasmic reticulum (ER) stress. Together with DDIT3/CHOP, mediates ER-mediated cell death by promoting expression of genes involved in cellular amino acid metabolic processes, mRNA translation and the unfolded protein response (UPR) in response to ER stress. | others | Longevity regulating pathway |
| ATF6B | Cyclic AMP-dependent transcription factor ATF-6 beta | Transcriptional factor that acts in the unfolded protein response (UPR) pathway by activating UPR target genes induced during ER stress. Binds DNA on the 5'-CCAC[GA]-3' half of the ER stress response element (ERSE) (5'-CCAATN9CCAC[GA]-3') when NF-Y is bound to ERSE. | loss of proteostasis | Longevity regulating pathway |
| ATG101 | Autophagy-related protein 101 | Autophagy factor required for autophagosome formation. Stabilizes ATG13, protecting it from proteasomal degradation. | others | Longevity regulating pathway |
| ATG13 | Autophagy-related protein 13 | Autophagy factor required for autophagosome formation and mitophagy. Target of the TOR kinase signaling pathway that regulates autophagy through the control of the phosphorylation status of ATG13 and ULK1, and the regulation of the ATG13-ULK1-RB1CC1 complex. Through its regulation of ULK1 activity, plays a role in the regulation of the kinase activity of mTORC1 and cell proliferation. | others | Longevity regulating pathway |
| ATG5 | Autophagy protein 5 | Involved in autophagic vesicle formation. Conjugation with ATG12, through a ubiquitin-like conjugating system involving ATG7 as an E1-like activating enzyme and ATG10 as an E2-like conjugating enzyme, is essential for its function. The ATG12-ATG5 conjugate acts as an E3-like enzyme which is required for lipidation of ATG8 family proteins and their association to the vesicle membranes. Involved in mitochondrial quality control after oxidative damage, and in subsequent cellular longevity. Plays a critical role in multiple aspects of lymphocyte development and is essential for both B and T lymphocyte survival and proliferation. Required for optimal processing and presentation of antigens for MHC II. Involved in the maintenance of axon morphology and membrane structures, as well as in normal adipocyte differentiation. Promotes primary ciliogenesis through removal of OFD1 from centriolar satellites and degradation of IFT20 via the autophagic pathway. | others | Longevity regulating pathway |
| ATM | ATM serine/threonine kinase | Serine/threonine protein kinase which activates checkpoint signaling upon double strand breaks (DSBs), apoptosis and genotoxic stresses such as ionizing ultraviolet A light (UVA), thereby acting as a DNA damage sensor. | genomic instability | p53 signaling pathway |
| ATP5O | ATP synthase, H+ transporting, mitochondrial F1 complex, O subunit | ATP5O is a mitochondrial protein. | mitochondrial dysfunction | Unknown |
| ATR | ATR serine/threonine kinase | ATR is involved in DNA repair by activating checkpoint signaling during genotoxic stresses including the phosphorylation of ageing-related proteins such as BRCA1 and TP53. | stem cell exhaustion | p53 signaling pathway |
| AXL | AXL receptor tyrosine kinase | Receptor tyrosine kinase that transduces signals from the extracellular matrix into the cytoplasm by binding growth factor GAS6 and which is thus regulating many physiological processes including cell survival, cell proliferation, migration and differentiation. Ligand binding at the cell surface induces dimerization and autophosphorylation of AXL. | senescence-associated secretory phenotype | Unknown |
| BAK1 | BCL2-antagonist/killer 1 | Plays a role in the mitochondrial apoptosic process. Upon arrival of cell death signals, promotes mitochondrial outer membrane (MOM) permeabilization by oligomerizing to form pores within the MOM. This releases apoptogenic factors into the cytosol, including cytochrome c, promoting the activation of caspase 9 which in turn processes and activates the effector caspases. | mitochondrial dysfunction | Apoptosis |
| BAX | BCL2-associated X protein | BAX is an important player in apoptosis. | others | Longevity regulating pathway |
| BCL10 | BCL10 immune signaling adaptor | Involved in adaptive immune response (PubMed:25365219). Promotes apoptosis, pro-caspase-9 maturation and activation of NF-kappa-B via NIK and IKK. May be an adapter protein between upstream TNFR1-TRADD-RIP complex and the downstream NIK-IKK-IKAP complex. | NF-κB related gene | NF-kappa B signaling pathway |
| BCL2 | B-cell CLL/lymphoma 2 | Suppresses apoptosis in a variety of cell systems including factor-dependent lymphohematopoietic and neural cells. Regulates cell death by controlling the mitochondrial membrane permeability. | others | p53 signaling pathway |
| BCL2A1 | BCL2 related protein A1 | Retards apoptosis induced by IL-3 deprivation. May function in the response of hemopoietic cells to external signals and in maintaining endothelial survival during infection. | NF-κB related gene | NF-kappa B signaling pathway |
| BCL2L1 | BCL2 like 1 | Potent inhibitor of cell death. Inhibits activation of caspases. Appears to regulate cell death by blocking the voltage-dependent anion channel (VDAC) by binding to it and preventing the release of the caspase activator, CYC1, from the mitochondrial membrane. | NF-κB related gene | NF-kappa B signaling pathway |
| BDNF | brain-derived neurotrophic factor | BDNF is a growth factor that promotes neuronal survival and is involved in numerous neuronal responses. | cellular senescence | MAPK signaling pathway |
| BIRC2 | baculoviral IAP repeat containing 2 | Multi-functional protein which regulates not only caspases and apoptosis, but also modulates inflammatory signaling and immunity, mitogenic kinase signaling, and cell proliferation, as well as cell invasion and metastasis. | NF-κB related gene | NF-kappa B signaling pathway |
| BIRC3 | Baculoviral IAP Repeat Containing 3 | Multi-functional protein which regulates not only caspases and apoptosis, but also modulates inflammatory signaling and immunity, mitogenic kinase signaling and cell proliferation, as well as cell invasion and metastasis. | NF-κB related gene | NF-kappa B signaling pathway |
| BLM | Bloom syndrome, RecQ helicase-like | ATP-dependent DNA helicase that unwinds single- and double-stranded DNA in a 3'-5' direction. | genomic instability | Unknown |
| BMI1 | BMI1 proto-oncogene, polycomb ring finger | BMI1 is an oncogene involved in transcriptional regulation by remodelling chromatin. It may also be involved in development. | stem cell exhaustion | Signaling pathways regulating pluripotency of stem cells |
| BMP2 | Bone Morphogenetic Protein 2 | Induces cartilage and bone formation. Stimulates the differentiation of myoblasts into osteoblasts via the EIF2AK3-EIF2A- ATF4 pathway. BMP2 activation of EIF2AK3 stimulates phosphorylation of EIF2A which leads to increased expression of ATF4 which plays a central role in osteoblast differentiation. | senescence-associated secretory phenotype | TGF-beta signaling pathway |
| BMP6 | Bone Morphogenetic Protein 6 | Induces cartilage and bone formation. | senescence-associated secretory phenotype | TGF-beta signaling pathway |
| BRCA1 | breast cancer 1, early onset | E3 ubiquitin-protein ligase that specifically mediates the formation of 'Lys-6'-linked polyubiquitin chains and plays a central role in DNA repair by facilitating cellular responses to DNA damage. | genomic instability | PI3K-Akt signaling pathway |
| BRCA2 | breast cancer 2, early onset | XRCC1 is a molecular scaffold protein that assembles multi-protein complexes involved in DNA single-strand break repair. Cells from Homo sapiens patients with mutations in BRCA2 exhibited reduced rates of DNA repair and elevated levels of protein ADP-ribosylation. | genomic instability | Unknown |
| BSCL2 | Berardinelli-Seip congenital lipodystrophy 2 (seipin) | It is a regulator of lipid catabolism essential for adipocyte differentiation. May also be involved in the central regulation of energy homeostasis. Necessary for correct lipid storage and lipid droplets maintenance; may play a tissue-autonomous role in controlling lipid storage in adipocytes and in preventing ectopic lipid droplet formation in non-adipose tissues. | deregulated nutrient sensing | Unknown |
| BTK | Bruton Tyrosine Kinase | Non-receptor tyrosine kinase indispensable for B lymphocyte development, differentiation and signaling. Binding of antigen to the B-cell antigen receptor (BCR) triggers signaling that ultimately leads to B-cell activation. After BCR engagement and activation at the plasma membrane, phosphorylates PLCG2 at several sites, igniting the downstream signaling pathway through calcium mobilization, followed by activation of the protein kinase C (PKC) family members. | NF-κB related gene | NF-kappa B signaling pathway |
| BUB1B | BUB1 mitotic checkpoint serine/threonine kinase B | BUB1B is a mitotic checkpoint that controls chromosome segregation and maintains genetic stability. Inhibit aging. | genomic instability | Cell cycle |
| BUB3 | BUB3 mitotic checkpoint protein | It has a dual function in spindle-assembly checkpoint signaling and in promoting the establishment of correct kinetochore-microtubule (K-MT) attachments. Promotes the formation of stable end-on bipolar attachments. Necessary for kinetochore localization of BUB1. Regulates chromosome segregation during oocyte meiosis. The BUB1/BUB3 complex plays a role in the inhibition of anaphase-promoting complex or cyclosome (APC/C) when spindle-assembly checkpoint is activated and inhibits the ubiquitin ligase activity of APC/C by phosphorylating its activator CDC20. This complex can also phosphorylate MAD1L1. | others | Cell cycle |
| C1QA | complement component 1, q subcomponent, A chain | C1q associates with the proenzymes C1r and C1s to yield C1, the first component of the serum complement system. The collagen-like regions of C1q interact with the Ca (2+)-dependent C1r (2)C1s (2) proenzyme complex, and efficient activation of C1 takes place on interaction of the globular heads of C1q with the Fc regions of IgG or IgM antibody present in immune complexes. | others | Unknown |
| CACNA1A | calcium channel, voltage-dependent, P/Q type, alpha 1A subunit | CACNA1A mediates calcium ions in a variety of processes such as cell division and gene expression. | others | MAPK signaling pathway |
| CALR | Calreticulin | Calcium-binding chaperone that promotes folding, oligomeric assembly and quality control in the endoplasmic reticulum (ER) via the calreticulin/calnexin cycle. This lectin interacts transiently with almost all of the monoglucosylated glycoproteins that are synthesized in the ER. Interacts with the DNA-binding domain of NR3C1 and mediates its nuclear export. Involved in maternal gene expression regulation. May participate in oocyte maturation via the regulation of calcium homeostasis (By similarity). | loss of proteostasis | Protein processing in endoplasmic reticulum |
| CAMK4 | calcium/calmodulin-dependent protein kinase type IV | Calcium/calmodulin-dependent protein kinase that operates in the calcium-triggered CaMKK-CaMK4 signaling cascade and regulates, mainly by phosphorylation, the activity of several transcription activators, such as CREB1, MEF2D, JUN and RORA, which play pivotal roles in immune response, inflammation, and memory consolidation. In the thymus, regulates the CD4+/CD8+ double positive thymocytes selection threshold during T-cell ontogeny. In CD4 memory T-cells, is required to link T-cell antigen receptor (TCR) signaling to the production of IL2, IFNG and IL4 (through the regulation of CREB and MEF2). Regulates the differentiation and survival phases of osteoclasts and dendritic cells (DCs). Mediates DCs survival by linking TLR4 and the regulation of temporal expression of BCL2. | others | Longevity regulating pathway |
| CAMKK2 | Calcium/calmodulin-dependent protein kinase kinase 2 | Calcium/calmodulin-dependent protein kinase belonging to a proposed calcium-triggered signaling cascade involved in a number of cellular processes. Isoform 1, isoform 2 and isoform 3 phosphorylate CAMK1 and CAMK4. Isoform 3 phosphorylates CAMK1D. Isoform 4, isoform 5 and isoform 6 lacking part of the calmodulin-binding domain are inactive. Efficiently phosphorylates 5'-AMP-activated protein kinase (AMPK) trimer, including that consisting of PRKAA1, PRKAB1 and PRKAG1. This phosphorylation is stimulated in response to Ca2+ signals. Seems to be involved in hippocampal activation of CREB1. May play a role in neurite growth. Isoform 3 may promote neurite elongation, while isoform 1 may promoter neurite branching. | others | Longevity regulating pathway |
| CARD10 | Caspase Recruitment Domain Family Member 10 | Activates NF-kappa-B via BCL10 and IKK. | NF-κB related gene | NF-kappa B signaling pathway |
| CAT | catalase | Cardiac-specific overexpression prolongs lifespan in mice. Catalase protects cardiomyocytes from ageing-induced contractile defects and protein damage. | others | Longevity regulating pathway |
| CBX7 | chromobox protein homolog 7 | Component of a Polycomb group (PcG) multiprotein PRC1-like complex, a complex class required to maintain the transcriptionally repressive state of many genes, including Hox genes, throughout development. PcG PRC1 complex acts via chromatin remodeling and modification of histones; it mediates monoubiquitination of histone H2A 'Lys-119', rendering chromatin heritably changed in its expressibility. Promotes histone H3 trimethylation at 'Lys-9' (H3K9me3). Binds to trimethylated lysine residues in histones, and possibly also other proteins. Regulator of cellular lifespan by maintaining the repression of CDKN2A, but not by inducing telomerase activity. | genomic instability | Unknown |
| CCL1 | C-C Motif Chemokine Ligand 1 | Cytokine that is chemotactic for monocytes but not for neutrophils. Binds to CCR8. | senescence-associated secretory phenotype | Unknown |
| CCL13 | C-C Motif Chemokine Ligand 13 | Chemotactic factor that attracts monocytes, lymphocytes, basophils and eosinophils, but not neutrophils. Signals through CCR2B and CCR3 receptors. Plays a role in the accumulation of leukocytes at both sides of allergic and non-allergic inflammation. | senescence-associated secretory phenotype | NF-kappa B signaling pathway |
| CCL19 | C-C Motif Chemokine Ligand 19 | May play a role not only in inflammatory and immunological responses but also in normal lymphocyte recirculation and homing. May play an important role in trafficking of T-cells in thymus, and T-cell and B-cell migration to secondary lymphoid organs. Binds to chemokine receptor CCR7. Recombinant CCL19 shows potent chemotactic activity for T-cells and B-cells but not for granulocytes and monocytes. Binds to atypical chemokine receptor ACKR4 and mediates the recruitment of beta-arrestin (ARRB1/2) to ACKR4. | NF-κB related gene | NF-kappa B signaling pathway |
| CCL20 | C-C Motif Chemokine Ligand 20 | Acts as a ligand for C-C chemokine receptor CCR6. Signals through binding and activation of CCR6 and induces a strong chemotactic response and mobilization of intracellular calcium ions. | senescence-associated secretory phenotype | Unknown |
| CCL21 | C-C Motif Chemokine Ligand 21 | Inhibits hemopoiesis and stimulates chemotaxis. Chemotactic in vitro for thymocytes and activated T-cells, but not for B-cells, macrophages, or neutrophils. Shows preferential activity towards naive T-cells. | NF-κB related gene | NF-kappa B signaling pathway |
| CCL26 | C-C Motif Chemokine Ligand 26 | Chemoattractant for eosinophils and basophils. | senescence-associated secretory phenotype | Unknown |
| CCL3 | C-C Motif Chemokine Ligand 3 | Monokine with inflammatory and chemokinetic properties. Binds to CCR1, CCR4 and CCR5. One of the major HIV-suppressive factors produced by CD8+ T-cells. | senescence-associated secretory phenotype | Unknown |
| CCL4 | C-C Motif Chemokine Ligand 4 | Monokine with inflammatory and chemokinetic properties. Binds to CCR5. One of the major HIV-suppressive factors produced by CD8+ T-cells. Recombinant MIP-1-beta induces a dose-dependent inhibition of different strains of HIV-1, HIV-2, and simian immunodeficiency virus (SIV). The processed form MIP-1-beta (3-69) retains the abilities to induce down-modulation of surface expression of the chemokine receptor CCR5 and to inhibit the CCR5-mediated entry of HIV-1 in T-cells. | NF-κB related gene | NF-kappa B signaling pathway |
| CCL7 | C-C Motif Chemokine Ligand 7 | Chemotactic factor that attracts monocytes and eosinophils, but not neutrophils. Augments monocyte anti-tumor activity. | senescence-associated secretory phenotype | Unknown |
| CCNA2 | cyclin A2 | Involved in cell cycle control， binds and activates CDK2 and promotes transition through G1/S and G2/M. | stem cell exhaustion | Cellular senescence |
| CD14 | CD14 Molecule | Coreceptor for bacterial lipopolysaccharide (PubMed:1698311, PubMed:23264655). In concert with LBP, binds to monomeric lipopolysaccharide and delivers it to the LY96/TLR4 complex, thereby mediating the innate immune response to bacterial lipopolysaccharide (LPS). | NF-κB related gene | NF-kappa B signaling pathway |
| CD40LG | CD40 Ligand | Cytokine that acts as a ligand to CD40/TNFRSF5. Costimulates T-cell proliferation and cytokine production. | NF-κB related gene | NF-kappa B signaling pathway |
| CD55 | CD55 Molecule (Cromer Blood Group) | This protein recognizes C4b and C3b fragments that condense with cell-surface hydroxyl or amino groups when nascent C4b and C3b are locally generated during C4 and c3 activation. | senescence-associated secretory phenotype | Unknown |
| CD9 | CD9 Molecule | Integral membrane protein associated with integrins, which regulates different processes, such as sperm-egg fusion, platelet activation and aggregation, and cell adhesion. | senescence-associated secretory phenotype | Unknown |
| CDC42 | cell division cycle 42 | Probably involved in the organization of the actin cytoskeleton by acting downstream of CDC42, inducing actin filament assembly. Alters CDC42-induced cell shape changes. In activated T-cells, may play a role in CDC42-mediated F-actin accumulation at the immunological synapse. May play a role in early contractile events in phagocytosis in macrophages. | others | MAPK signaling pathway |
| CDK1 | cyclin-dependent kinase 1 | An important regulator of the cell cycle, CDK1 also appears to be involved in apoptosis. | cellular senescence | p53 signaling pathway |
| CDK7 | cyclin-dependent kinase 7 | Serine/threonine kinase involved in cell cycle control and in RNA polymerase II-mediated RNA transcription. Cyclin-dependent kinases (CDKs) are activated by the binding to a cyclin and mediate the progression through the cell cycle. Each different complex controls a specific transition between 2 subsequent phases in the cell cycle. Required for both activation and complex formation of CDK1/cyclin-B during G2-M transition, and for activation of CDK2/cyclins during G1-S transition (but not complex formation). Its expression and activity are constant throughout the cell cycle. Upon DNA damage, triggers p53/TP53 activation by phosphorylation, but is inactivated in turn by p53/TP53; this feedback loop may lead to an arrest of the cell cycle and of the transcription, helping in cell recovery, or to apoptosis. Required for DNA-bound peptides-mediated transcription and cellular growth inhibition. | others | Cell cycle |
| CDKN1A | cyclin-dependent kinase inhibitor 1A (p21, Cip1) | May be involved in p53/TP53 mediated inhibition of cellular proliferation in response to DNA damage. Binds to and inhibits cyclin-dependent kinase activity, preventing phosphorylation of critical cyclin-dependent kinase substrates and blocking cell cycle progression. Functions in the nuclear localization and assembly of cyclin D-CDK4 complex and promotes its kinase activity towards RB1. At higher stoichiometric ratios, inhibits the kinase activity of the cyclin D-CDK4 complex. Inhibits DNA synthesis by DNA polymerase delta by competing with POLD3 for PCNA binding. Plays an important role in controlling cell cycle progression and DNA damage-induced G2 arrest. | genomic instability | p53 signaling pathway |
| CDKN2A | cyclin-dependent kinase inhibitor 2A | The CDKN2A gene encodes different transcripts involved mostly in cell cycle regulation and cellular senescence, including the tumour suppressor proteins p16 and p19. | cellular senescence | p53 signaling pathway |
| CDKN2B | cyclin-dependent kinase inhibitor 2B (p15, inhibits CDK4) | Interacts strongly with CDK4 and CDK6. Potent inhibitor. Potential effector of TGF-beta induced cell cycle arrest. | others | Cellular senescence |
| CEBPA | CCAAT/enhancer binding protein (C/EBP), alpha | CEBPA is a transcription factor expressed in adipose tissues that modulates the expression of leptin (LEP). CEBPA-null die shortly after birth. | others | Unknown |
| CEBPB | CCAAT/enhancer binding protein (C/EBP), beta | Like CEBPA, CEBPB is a transcription factor involved in fat metabolism. CEBPB also appears to play a role in liver regeneration. | others | IL-17 signaling pathway |
| CETP | cholesteryl ester transfer protein, plasma | Involved in the transfer of neutral lipids, including cholesteryl ester and triglyceride, among lipoprotein particles. Allows the net movement of cholesteryl ester from high density lipoproteins/HDL to triglyceride-rich very low-density lipoproteins/VLDL, and the equimolar transport of triglyceride from VLDL to HDL. Regulates the reverse cholesterol transport, by which excess cholesterol is removed from peripheral tissues and returned to the liver for elimination. | deregulated nutrient sensing | Unknown |
| CFLAR | CASP8 And FADD Like Apoptosis Regulator | Apoptosis regulator protein which may function as a crucial link between cell survival and cell death pathways in mammalian cells. Acts as an inhibitor of TNFRSF6 mediated apoptosis. | NF-κB related gene | NF-kappa B signaling pathway |
| CHCHD2 | coiled-coil-helix-coiled-coil-helix domain containing 2 | Mutations in the CHCHD2 gene have been linked to autosomal dominant forms of Parkinson's disease (PD). | others | Unknown |
| ARNTL | aryl hydrocarbon receptor nuclear translocator-like | Transcriptional activator which forms a core component of the circadian clock. The circadian clock, an internal time-keeping system, regulates various physiological processes through the generation of approximately 24 hours circadian rhythms in gene expression, which are translated into rhythms in metabolism and behavior. | others | Unknown |
| ATP5O | ATP synthase, H+ transporting, mitochondrial F1 complex, O subunit | ATP5O is a mitochondrial protein. | mitochondrial dysfunction | Unknown |
| AXL | AXL receptor tyrosine kinase | Receptor tyrosine kinase that transduces signals from the extracellular matrix into the cytoplasm by binding growth factor GAS6 and which is thus regulating many physiological processes including cell survival, cell proliferation, migration and differentiation. Ligand binding at the cell surface induces dimerization and autophosphorylation of AXL. | senescence-associated secretory phenotype | Unknown |
| BLM | Bloom syndrome, RecQ helicase-like | ATP-dependent DNA helicase that unwinds single- and double-stranded DNA in a 3'-5' direction. | genomic instability | Unknown |
| FOXL2 | Forkhead Box Protein L2 | Transcriptional regulator. Critical factor essential for ovary differentiation and maintenance, and repression of the genetic program for somatic testis determination. Prevents trans-differentiation of ovary to testis through transcriptional repression of the Sertoli cell-promoting gene SOX9 (By similarity). Has apoptotic activity in ovarian cells. Suppresses ESR1-mediated transcription of PTGS2/COX2 stimulated by tamoxifen (By similarity). Is a regulator of CYP19 expression (By similarity). Participates in SMAD3-dependent transcription of FST via the intronic SMAD-binding element (By similarity). Is a transcriptional repressor of STAR. Activates SIRT1 transcription under cellular stress conditions. Activates transcription of OSR2. | others | Unknown |
| BRCA2 | breast cancer 2, early onset | XRCC1 is a molecular scaffold protein that assembles multi-protein complexes involved in DNA single-strand break repair. Cells from Homo sapiens patients with mutations in BRCA2 exhibited reduced rates of DNA repair and elevated levels of protein ADP-ribosylation. | genomic instability | Unknown |
| C1QA | complement component 1, q subcomponent, A chain | C1q associates with the proenzymes C1r and C1s to yield C1, the first component of the serum complement system. The collagen-like regions of C1q interact with the Ca (2+)-dependent C1r(2)C1s(2) proenzyme complex, and efficient activation of C1 takes place on interaction of the globular heads of C1q with the Fc regions of IgG or IgM antibody present in immune complexes. | others | Unknown |
| CD9 | CD9 Molecule | Integral membrane protein associated with integrins, which regulates different processes, such as sperm-egg fusion, platelet activation and aggregation, and cell adhesion. | senescence-associated secretory phenotype | Unknown |
| CEBPA | CCAAT/enhancer binding protein (C/EBP), alpha | CEBPA is a transcription factor expressed in adipose tissues that modulates the expression of leptin (LEP). CEBPA-null die shortly after birth. | others | Unknown |
| CETP | cholesteryl ester transfer protein, plasma | Involved in the transfer of neutral lipids, including cholesteryl ester and triglyceride, among lipoprotein particles. Allows the net movement of cholesteryl ester from high density lipoproteins/HDL to triglyceride-rich very low-density lipoproteins/VLDL, and the equimolar transport of triglyceride from VLDL to HDL. Regulates the reverse cholesterol transport, by which excess cholesterol is removed from peripheral tissues and returned to the liver for elimination. | deregulated nutrient sensing | Unknown |
| ERCC8 | excision repair cross-complementation group 8 | Substrate-recognition component of the CSA complex, a DCX (DDB1-CUL4-X-box) E3 ubiquitin-protein ligase complex, involved in transcription-coupled nucleotide excision repair. | genomic instability | Unknown |
| CLU | clusterin | It may be involved in apoptosis. | cellular senescence | Unknown |
| CNR1 | cannabinoid receptor 1 (brain) | G-protein coupled receptor for endogenous cannabinoids (eCBs), including N-arachidonoylethanolamide (also called anandamide or AEA) and 2-arachidonoylglycerol (2-AG), as well as phytocannabinoids, such as delta9-tetrahydrocannabinol (THC). Mediates many cannabinoid-induced effects, acting, among others, on food intake, memory loss, gastrointestinal motility, catalepsy, ambulatory activity, anxiety, chronic pain. Signaling typically involves reduction in cyclic AMP. In the hypothalamus, may have a dual effect on mitochondrial respiration depending upon the agonist dose and possibly upon the cell type. May also affect de novo cholesterol synthesis and HDL-cholesteryl ether uptake. Peripherally modulates energy metabolism. | deregulated nutrient sensing | Unknown |
| CSF2RB | Colony Stimulating Factor 2 Receptor Subunit Beta | High affinity receptor for interleukin-3, interleukin-5 and granulocyte-macrophage colony-stimulating factor. | senescence-associated secretory phenotype | Unknown |
| CTGF | connective tissue growth factor | The encoded protein plays a role in chondrocyte proliferation and differentiation, cell adhesion in many cell types, and is related to platelet-derived growth factor. | stem cell exhaustion | Unknown |
| CD55 | CD55 Molecule (Cromer Blood Group) | This protein recognizes C4b and C3b fragments that condense with cell-surface hydroxyl or amino groups when nascent C4b and C3b are locally generated during C4 and c3 activation. | senescence-associated secretory phenotype | Unknown |
| DBN1 | drebrin 1 | DBN1 is thought to be involved in neuronal growth. | loss of proteostasis | Unknown |
| DLAT | dihydrolipoamide S-acetyltransferase | The pyruvate dehydrogenase complex catalyzes the overall conversion of pyruvate to acetyl-CoA and CO (2), and thereby links the glycolytic pathway to the tricarboxylic cycle. | mitochondrial dysfunction | Unknown |
| EEF1A1 | eukaryotic translation elongation factor 1 alpha 1 | EEF1A1 is involved in the binding of tRNA to ribosomes during protein synthesis. This protein promotes the GTP-dependent binding of aminoacyl-tRNA to the A-site of ribosomes during protein biosynthesis. With PARP1 and TXK, forms a complex that acts as a T helper 1 (Th1) cell-specific transcription factor and binds the promoter of IFN-gamma to directly regulate its transcription, and is thus involved importantly in Th1 cytokine production. | genomic instability | Unknown |
| EEF2 | eukaryotic translation elongation factor 2 | Age-dependent changes to EEF2 in rats, which could account for the decline in protein synthesis in old animals. | loss of proteostasis | Unknown |
| EMD | emerin | The functions of EMD in the nuclear lamina may indicate a role for EMD in ageing and have roles in chromatin organization, gene regulation and signal transduction. | others | Unknown |
| EPS8 | epidermal growth factor receptor pathway substrate 8 | Stimulates guanine exchange activity of SOS1. May play a role in membrane ruffling and remodeling of the actin cytoskeleton. In the cochlea, is required for stereocilia maintenance in adult hair cells. | others | Unknown |
| ERCC1 | excision repair cross-complementation group 1 | ERCC1 has a role in DNA repair in association with the other ERCC proteins. | genomic instability | Unknown |
| ERCC2 | excision repair cross-complementation group 2 | ATP-dependent 5'-3' DNA helicase, component of the general transcription and DNA repair factor IIH (TFIIH) core complex, which is involved in general and transcription-coupled nucleotide excision repair (NER) of damaged DNA and, when complexed to CAK, in RNA transcription by RNA polymerase II. | genomic instability | Unknown |
| ERCC3 | excision repair cross-complementation group 3 | ERCC3 is an ATP-dependent 3'-5' directed DNA helicase involved in basal RNA transcription and the nucleotide excision repair (NER) pathway. | genomic instability | Unknown |
| ERCC4 | excision repair cross-complementation group 4 | Catalytic component of a structure-specific DNA repair endonuclease responsible for the 5-prime incision during DNA repair. Involved in homologous recombination that assists in removing interstrand cross-link. | genomic instability | Unknown |
| ERCC5 | excision repair cross-complementation group 5 | ERCC5 may reduce endogenous DNA damage and attend therapy for human progeroid genome instability syndromes. | genomic instability | Unknown |
| ERCC6 | excision repair cross-complementation group 6 | Mutant for ERCC6 and XPA die before weaning and display some signs of premature ageing included stunted growth, neurological dysfunction, retinal degeneration, cachexia, and kyphosis. | others | Unknown |
| ESR1 | estrogen receptor 1 | ESR1 is a transcription factor that mediates the actions of estrogen. Mice without ESR1 were infertile and showed a variety of changes. | genomic instability | Unknown |
| EFEMP1 | EGF containing fibulin-like extracellular matrix protein 1 | Binds EGFR, the EGF receptor, inducing EGFR autophosphorylation and the activation of downstream signaling pathways. May play a role in cell adhesion and migration. May function as a negative regulator of chondrocyte differentiation. In the olfactory epithelium, it may regulate glial cell migration, differentiation and the ability of glial cells to support neuronal neurite outgrowth. | altered intercellular communication | Unknown |
| FEN1 | flap structure-specific endonuclease 1 | Involved in DNA processing, FEN-1 removes 5' overhanging flaps in DNA repair and processes the 5' ends of Okazaki fragments in lagging strand DNA synthesis. | genomic instability | Unknown |
| FLT1 | fms-related tyrosine kinase 1 | It is involved in signaling cascade. | epigenetic alterations | Unknown |
| XRCC6 | X-ray repair complementing defective repair in Chinese hamster cells 6 | Ku70 forms a heterodimer with Ku80, called Ku, that is critical for repairing DNA double-stand breaks by nonhomologous end joining and for maintaining telomeres. The Ku heterodimer is important for longevity assurance in mice. | others | Unknown |
| GCLC | glutamate-cysteine ligase, catalytic subunit | Glutamate-cysteine ligase, also known as gamma-glutamylcysteine synthetase is the first rate-limiting enzyme of glutathione synthesis. The enzyme consists of two subunits, a heavy catalytic subunit and a light regulatory subunit. This locus encodes the catalytic subunit, while the regulatory subunit is derived from a different gene located on chromosome 1p22-p21. Mutations at this locus have been associated with hemolytic anemia due to deficiency of gamma-glutamylcysteine synthetase and susceptibility to myocardial infarction. | loss of proteostasis | Unknown |
| GCLM | glutamate-cysteine ligase, modifier subunit | Glutamate-cysteine ligase, also known as gamma-glutamylcysteine synthetase, is the first rate limiting enzyme of glutathione synthesis. The enzyme consists of two subunits, a heavy catalytic subunit and a light regulatory subunit. Gamma glutamylcysteine synthetase deficiency has been implicated in some forms of hemolytic anemia. Alternative splicing results in multiple transcript variants encoding different isoforms. | loss of proteostasis | Unknown |
| GPX1 | glutathione peroxidase 1 | Overexpression of GPX1 in mice results in insulin (INS) resistance and obesity. | deregulated nutrient sensing | Unknown |
| GPX4 | glutathione peroxidase 4 | Essential antioxidant peroxidase that directly reduces phospholipid hydroperoxide even if they are incorporated in membranes and lipoproteins. Can also reduce fatty acid hydroperoxide, cholesterol hydroperoxide and thymine hydroperoxide. Plays a key role in protecting cells from oxidative damage by preventing membrane lipid peroxidation. Required to prevent cells from ferroptosis, a non-apoptotic cell death resulting from an iron-dependent accumulation of lipid reactive oxygen species. Plays a role in a primary T-cell response to viral and parasitic infection by protecting T-cells from ferroptosis and by supporting T-cell expansion. Plays a role of glutathione peroxidase in platelets in the arachidonic acid metabolism. | others | Unknown |
| GRN | granulin | Secreted protein that acts as a key regulator of lysosomal function and as a growth factor involved in inflammation, wound healing and cell proliferation. Regulates protein trafficking to lysosomes and, also the activity of lysosomal enzymes. Facilitates also the acidification of lysosomes, causing degradation of mature CTSD by CTSB. In addition, functions as wound-related growth factor that acts directly on dermal fibroblasts and endothelial cells to promote division, migration and the formation of capillary-like tubule structures. Also promotes epithelial cell proliferation by blocking TNF-mediated neutrophil activation preventing release of oxidants and proteases. Moreover, modulates inflammation in neurons by preserving neurons survival, axonal outgrowth and neuronal integrity. | others | Unknown |
| NR3C1 | nuclear receptor subfamily 3, group C, member 1 (glucocorticoid receptor) | Receptor for glucocorticoids. Has a dual mode of action: as a transcription factor that binds to glucocorticoid response elements (GRE), both for nuclear and mitochondrial DNA, and as a modulator of other transcription factors. | others | Unknown |
| GSK3A | glycogen synthase kinase 3 alpha | Constitutively active protein kinase that acts as a negative regulator in the hormonal control of glucose homeostasis, Wnt signaling and regulation of transcription factors and microtubules, by phosphorylating and inactivating glycogen synthase (GYS1 or GYS2), CTNNB1/beta-catenin, APC and AXIN1. Requires primed phosphorylation of the majority of its substrates. Contributes to insulin regulation of glycogen synthesis by phosphorylating and inhibiting GYS1 activity and hence glycogen synthesis. Regulates glycogen metabolism in liver, but not in muscle. May also mediate the development of insulin resistance by regulating activation of transcription factors. Acts as a regulator of autophagy by mediating phosphorylation of KAT5/TIP60 under starvation conditions, leading to activate KAT5/TIP60 acetyltransferase activity and promote acetylation of key autophagy regulators, such as ULK1 and RUBCNL/Pacer. | deregulated nutrient sensing | Unknown |
| GSR | glutathione reductase | GSR is involved in redox regulation and oxidative defence. | others | Unknown |
| GSS | glutathione synthetase | Mutations in the human GSS gene cause 5-oxoprolinuria. GSS is involved in redox regulation. | others | Unknown |
| GSTA4 | glutathione S-transferase alpha 4 | It is involved in oxidative protection as well as the removal of toxins and harmful metabolic by-products. | altered intercellular communication | Unknown |
| GSTP1 | glutathione S-transferase pi 1 | It is involved in oxidative protection, extend lifespan. | epigenetic alterations | Unknown |
| GTF2H2 | general transcription factor IIH, polypeptide 2, 44kDa | Transcription factor II H interacts with a variety of factors during transcription, suggesting that, in addition to its essential role in transcription initiation, it also participates as a regulatory factor. | epigenetic alterations | Unknown |
| H2AFX | H2A histone family, member X | H2AFX has been involved in DNA repair. | genomic instability | Unknown |
| HTT | huntingtin | DeltaQ-htt expression in vitro increases autophagosome synthesis and stimulates the Atg5-dependent clearance of truncated N-terminal htt aggregates. | loss of proteostasis | Unknown |
| HELLS | helicase, lymphoid-specific | The disruption of PASG (lsh), a SNF2-like factor that facilitates DNA methylation, causes global hypomethylation, developmental growth retardation and a premature aging phenotype. | epigenetic alterations | Unknown |
| NRG1 | neuregulin 1 | It is a direct ligand for ERBB3 and ERBB4 tyrosine kinase receptors. It concomitantly recruits ERBB1 and ERBB2 coreceptors, resulting in ligand-stimulated tyrosine phosphorylation and activation of the ERBB receptors. | cellular senescence | Unknown |
| HIC1 | hypermethylated in cancer 1 | Transcriptional repressor. Recognizes and binds to the consensus sequence '5-[CG]NG[CG]GGGCA[CA]CC-3'. May act as a tumor suppressor. May be involved in development of head, face, limbs and ventral body wall. Involved in down-regulation of SIRT1 and thereby is involved in regulation of p53/TP53-dependent apoptotic DNA-damage responses. The specific target gene promoter association seems to be depended on corepressors, such as CTBP1 or CTBP2 and MTA1. The regulation of SIRT1 transcription in response to nutrient deprivation seems to involve CTBP1. Involved in regulation of the Wnt signaling pathway probably by association with TCF7L2 and preventing TCF7L2 and CTNNB1 association with promoters of TCF-responsive genes. | genomic instability | Unknown |
| HMGB2 | high mobility group box 2 | HMGB2 has been related to DNA unwinding and stress response. | genomic instability | Unknown |
| HOXB7 | homeobox B7 | It is crucial in morphogenesis and development. The expression of many HOX genes, including HOXB7, declines with age, even prior to adulthood. | genomic instability | Unknown |
| HOXC4 | homeobox C4 | Sequence-specific transcription factor which is part of a developmental regulatory system that provides cells with specific positional identities on the anterior-posterior axis. | genomic instability | Unknown |
| HSF1 | heat shock transcription factor 1 | It plays a critical role in regulating the transcription of hsp genes. HSF1 in aged rats exhibits a decreased ability to bind DNA. | epigenetic alterations | Unknown |
| HSPA9 | heat shock 70kDa protein 9 (mortalin) | HSPA9 influenced pre-messenger RNA splicing by modulating the inclusion of the exons. | epigenetic alterations | Unknown |
| HSPD1 | heat shock 60kDa protein 1 (chaperonin) | It is involve in the aging-related apoptotic process, promote aging. | mitochondrial dysfunction | Unknown |
| IGFBP1 | insulin like growth factor binding protein 1 | As a downstream protein of Jagged1, IGFBP1 was correlated with the severity of coronary atherosclerosis in aging patients, and the increase of circulating IGFBP1 levels with aging may be an adaptive response to counter HCAEC senescence through Akt signaling. | senescence-associated secretory phenotype | Unknown |
| IGFBP2 | insulin-like growth factor binding protein 2, 36kDa | Inhibits IGF-mediated growth and developmental rates. IGF-binding proteins prolong the half-life of the IGFs and have been shown to either inhibit or stimulate the growth promoting effects of the IGFs on cell culture. They alter the interaction of IGFs with their cell surface receptors. | others | Unknown |
| KCNA3 | potassium channel, voltage gated shaker related subfamily A, member 3 | KCNA3 mediates the voltage-dependent potassium ion permeability of excitable membranes. Assuming opened or closed conformations in response to the voltage difference across the membrane, the protein forms a potassium-selective channel through which potassium ions may pass in accordance with their electrochemical gradient. | altered intercellular communication | Unknown |
| FADS1 | fatty acid desaturase 1 | Long-chain polyunsaturated fatty acids (LCPUFAs) biosynthetic capacity is regulated by FADS1 polymorphisms and decreased by aging in FADS1 C allele carriers. | others | Unknown |
| LRP2 | low density lipoprotein receptor-related protein 2 | LRP2 may also help regulate the release of parathyroid hormone. | deregulated nutrient sensing | Unknown |
| MXD1 | MAX dimerization protein 1 | MXD1 is involved in apoptosis. It forms with MAX a transcriptional repressor and might antagonize MYC function. | cellular senescence | Unknown |
| MIF | macrophage migration inhibitory factor (glycosylation-inhibiting factor) | Pro-inflammatory cytokine. It is involved in the innate immune response to bacterial pathogens. The expression of MIF at sites of inflammation suggests a role as mediator in regulating the function of macrophages in host defense. It counteracts the anti-inflammatory activity of glucocorticoids. It has phenylpyruvate tautomerase and dopachrome tautomerase activity (in vitro), but the physiological substrate is not known. | others | Unknown |
| MLH1 | mutL homolog 1 | Heterodimerizes with PMS2 to form MutL alpha, a component of the post-replicative DNA mismatch repair system (MMR). DNA repair is initiated by MutS alpha (MSH2-MSH6) or MutS beta (MSH2-MSH3) binding to a dsDNA mismatch, then MutL alpha is recruited to the heteroduplex. Assembly of the MutL-MutS-heteroduplex ternary complex in presence of RFC and PCNA is sufficient to activate endonuclease activity of PMS2. It introduces single-strand breaks near the mismatch and thus generates new entry points for the exonuclease EXO1 to degrade the strand containing the mismatch. | genomic instability | Unknown |
| MMP10 | Matrix Metallopeptidase 10 | Can degrade fibronectin, gelatins of type I, III, IV, and V; weakly collagens III, IV, and V. Activates procollagenase. | senescence-associated secretory phenotype | Unknown |
| MMP12 | Matrix Metallopeptidase 12 | May be involved in tissue injury and remodeling. Has significant elastolytic activity. Can accept large and small amino acids at the P1' site, but has a preference for leucine. Aromatic or hydrophobic residues are preferred at the P1 site, with small hydrophobic residues (preferably alanine) occupying P3. | senescence-associated secretory phenotype | Unknown |
| MSRA | methionine sulfoxide reductase A | A decline in MSRA activity could reduce the antioxidant defenses and increase the oxidation of critical proteins in neurons in the brain in AD. | others | Unknown |
| MT1E | metallothionein 1E | Metallothioneins have a high content of cysteine residues that bind various heavy metals; these proteins are transcriptionally regulated by both heavy metals and glucocorticoids. | others | Unknown |
| MT-CO1 | mitochondrially encoded cytochrome c oxidase I | MT-CO1 catalyses the reduction of oxygen to water, inactivation of it contributes to “immortality”. | mitochondrial dysfunction | Unknown |
| NUDT1 | nudix (nucleoside diphosphate linked moiety X)-type motif 1 | NUDT1 plays a redundant role in sanitizing oxidized nucleotide pools, such as 8-oxo-dGTP pools. It acts as a sanitizing enzyme for oxidized nucleotide pools, thus suppressing cell dysfunction and death induced by oxidative stress. It is able to hydrolyze also the corresponding ribonucleotides, 2-OH-ATP, 8-oxo-GTP and 8-oxo-ATP. | genomic instability | Unknown |
| MXI1 | MAX interactor 1, dimerization protein | Mxi1 is involved in the homeostasis of differentiated organ systems, acts as a tumour suppressor in vivo, and engages the Myc network in a functionally relevant manner. | others | Unknown |
| NFE2L1 | nuclear factor, erythroid 2-like 1 | NFE2L1 constitutes a precursor of the transcription factor NRF1. It is able to detect various cellular stresses, such as cholesterol excess, oxidative stress or proteasome inhibition. In response to stress, it is released from the endoplasmic reticulum membrane following cleavage by the protease DDI2 and translocates into the nucleus to form the transcription factor NRF1. | deregulated nutrient sensing | Unknown |
| NFE2L2 | nuclear factor, erythroid 2-like 2 | NFE2L2 is a ranscription factor that plays a key role in the response to oxidative stress: binds to antioxidant response (ARE) elements present in the promoter region of many cytoprotective genes, such as phase 2 detoxifying enzymes, and promotes their expression, thereby neutralizing reactive electrophiles. In normal conditions, ubiquitinated and degraded in the cytoplasm by the BCR(KEAP1) complex.The NFE2L2/NRF2 pathway is also activated in response to selective autophagy: autophagy promotes interaction between KEAP1 and SQSTM1/p62 and subsequent inactivation of the BCR(KEAP1) complex, leading to NFE2L2/NRF2 nuclear accumulation and expression of cytoprotective genes. | genomic instability | Unknown |
| PRDX1 | peroxiredoxin 1 | Prdx1 is an important defence against oxidants in ageing mice. Prdx1-deficient fibroblasts show decreased proliferation and increased sensitivity to oxidative DNA damage. | others | Unknown |
| PAPPA | pregnancy-associated plasma protein A, pappalysin 1 | PAPPA is a metalloproteinase which specifically cleaves insulin-like growth factor binding protein (IGFBP)-5 at the '163-Ser-\|-Lys-164' bond. It shows limited proteolysis toward IGFBP-3. | loss of proteostasis | Unknown |
| PCMT1 | protein-L-isoaspartate (D-aspartate) O-methyltransferase | It plays a role in the repair and/or degradation of damaged proteins, promote aging. | loss of proteostasis | Unknown |
| RAD27 | multifunctional nuclease RAD27 | Its deletion led to significant changes of chronological lifespan in yeast, featuring both lifespan shortening and lifespan extension. | genomic instability | Unknown |
| SPRTN | SprT-like N-terminal domain | SPRTN is recruited to sites of UV damage and interacts with ubiquitinated PCNA and RAD18, the E3 ubiquitin ligase that monoubiquitinates PCNA. SPRTN facilitates chromatin association of RAD18 and is required for efficient PCNA monoubiquitination, promoting a feed-forward loop to enhance PCNA ubiquitination and translesion DNA synthesis. SPRTN acts as a regulator of TLS by recruiting VCP/p97 to sites of DNA damage, possibly leading to extraction of DNA polymerase eta (POLH) by VCP/p97 to prevent excessive translesion DNA synthesis and limit the incidence of mutations induced by DNA damage. | genomic instability | Unknown |
| CISD2 | CDGSH iron sulfur domain 2 | Regulator of autophagy that contributes to antagonize BECN1-mediated cellular autophagy at the endoplasmic reticulum. Participates in the interaction of BCL2 with BECN1 and is required for BCL2-mediated depression of endoplasmic reticulum Ca2+ stores during autophagy. Contributes to BIK-initiated autophagy, while it is not involved in BIK-dependent activation of caspases. Involved in life span control, probably via its function as regulator of autophagy. | others | Unknown |
| TMEM67 | Transmembrane Protein 67 | Required for ciliary structure and function. Part of the tectonic-like complex which is required for tissue-specific ciliogenesis and may regulate ciliary membrane composition (By similarity). Involved in centrosome migration to the apical cell surface during early ciliogenesis. Involved in the regulation of cilia length and appropriate number through the control of centrosome duplication. Required for cell branching morphology. Essential for endoplasmic reticulum-associated degradation (ERAD) of surfactant protein C (SFTPC). | others | Unknown |
| NLRP3 | NLR family pyrin domain containing 3 | Several AMPK-dependent pathways regulate NLRP3 inflammasome activation during aging, suggesting NLRP3 as a potential pharmacological target in age-related diseases. | deregulated nutrient sensing | Unknown |
| MMP14 | Matrix Metallopeptidase 14 | Endopeptidase that degrades various components of the extracellular matrix such as collagen. Activates progelatinase A. Essential for pericellular collagenolysis and modeling of skeletal and extraskeletal connective tissues during development (By similarity). May be involved in actin cytoskeleton reorganization by cleaving PTK7 (PubMed:20837484). Acts as a positive regulator of cell growth and migration via activation of MMP15. Involved in the formation of the fibrovascular tissues in association with pro-MMP2 (PubMed:12714657). Cleaves ADGRB1 to release vasculostatin-40 which inhibits angiogenesis. | senescence-associated secretory phenotype | TNF signaling pathway |
| BMP2 | Bone Morphogenetic Protein 2 | Induces cartilage and bone formation. Stimulates the differentiation of myoblasts into osteoblasts via the EIF2AK3-EIF2A- ATF4 pathway. BMP2 activation of EIF2AK3 stimulates phosphorylation of EIF2A which leads to increased expression of ATF4 which plays a central role in osteoblast differentiation. | senescence-associated secretory phenotype | TGF-beta signaling pathway |
| BMP6 | Bone Morphogenetic Protein 6 | Induces cartilage and bone formation. | senescence-associated secretory phenotype | TGF-beta signaling pathway |
| SP1 | Sp1 transcription factor | SP1 regulates ageing-related genes such as WRN or cellular senescence-related genes like CDKN2A. | genomic instability | TGF-beta signaling pathway |
| NOG | noggin | NOG may be a factor in bone ageing. Essential for cartilage morphogenesis and joint formation. | stem cell exhaustion | TGF-beta signaling pathway |
| MVK | Mevalonate Kinase | Catalyzes the phosphorylation of mevalonate to mevalonate 5-phosphate, a key step in isoprenoid and cholesterol biosynthesis. | others | Terpenoid backbone biosynthesis |
| BMI1 | BMI1 proto-oncogene, polycomb ring finger | BMI1 is an oncogene involved in transcriptional regulation by remodelling chromatin. It may also be involved in development. | stem cell exhaustion | Signaling pathways regulating pluripotency of stem cells |
| TCF3 | transcription factor 3 | Transcriptional regulator. TCF3 is involved in the initiation of neuronal differentiation. Heterodimers between TCF3 and tissue-specific basic helix-loop-helix (bHLH) proteins play major roles in determining tissue-specific cell fate during embryogenesis, like muscle or early B-cell differentiation. | cellular senescence | Signaling pathways regulating pluripotency of stem cells |
| HESX1 | HESX homeobox 1 | HESX1 is an important regulator of development and, particularly, of pituitary development. | others | Signaling pathways regulating pluripotency of stem cells |
| CALR | Calreticulin | Calcium-binding chaperone that promotes folding, oligomeric assembly and quality control in the endoplasmic reticulum (ER) via the calreticulin/calnexin cycle. This lectin interacts transiently with almost all of the monoglucosylated glycoproteins that are synthesized in the ER. Interacts with the DNA-binding domain of NR3C1 and mediates its nuclear export. Involved in maternal gene expression regulation. May participate in oocyte maturation via the regulation of calcium homeostasis (By similarity). | loss of proteostasis | Protein processing in endoplasmic reticulum |
| BRCA1 | breast cancer 1, early onset | E3 ubiquitin-protein ligase that specifically mediates the formation of 'Lys-6'-linked polyubiquitin chains and plays a central role in DNA repair by facilitating cellular responses to DNA damage. | genomic instability | PI3K-Akt signaling pathway |
| ELN | elastin | ELN is structural protein that may be related to arterial morphogenesis. | altered intercellular communication | PI3K-Akt signaling pathway |
| HGF | hepatocyte growth factor | HGF and bFGF derived from hADSCs improved ovarian function during natural aging via reduction of oxidative stress by activating the SIRT1/FOXO1 signaling pathway. | senescence-associated secretory phenotype | PI3K-Akt signaling pathway |
| HSP90AA1 | heat shock protein 90kDa alpha (cytosolic), class A member 1 | Molecular chaperone that promotes the maturation, structural maintenance and proper regulation of specific target proteins involved for instance in cell cycle control and signal transduction. | others | PI3K-Akt signaling pathway |
| ITGA2 | integrin subunit alpha 2 | Overexpressed ITGA2 promoted the proliferation of ovarian cancer cells. ITGA2 regulated the phosphorylation of forkhead box O1 (FoxO1) by mediating AKT phosphorylation. | senescence-associated secretory phenotype | PI3K-Akt signaling pathway |
| RET | Ret Proto-Oncogene | Receptor tyrosine-protein kinase involved in numerous cellular mechanisms including cell proliferation, neuronal navigation, cell migration, and cell differentiation upon binding with glial cell derived neurotrophic factor family ligands. Phosphorylates PTK2/FAK1. Regulates both cell death/survival balance and positional information. Required for the molecular mechanisms orchestration during intestine organogenesis; involved in the development of enteric nervous system and renal organogenesis during embryonic life, and promotes the formation of Peyer's patch-like structures, a major component of the gut-associated lymphoid tissue. Modulates cell adhesion via its cleavage by caspase in sympathetic neurons and mediates cell migration in an integrin (e.g. ITGB1 and ITGB3)-dependent manner. Involved in the development of the neural crest. Active in the absence of ligand, triggering apoptosis through a mechanism that requires receptor intracellular caspase cleavage. Acts as a dependence receptor; in the presence of the ligand GDNF in somatotrophs (within pituitary), promotes survival and down regulates growth hormone (GH) production, but triggers apoptosis in absence of GDNF. Regulates nociceptor survival and size. Triggers the differentiation of rapidly adapting (RA) mechanoreceptors. Mediator of several diseases such as neuroendocrine cancers; these diseases are characterized by aberrant integrins-regulated cell migration. Mediates, through interaction with GDF15-receptor GFRAL, GDF15-induced cell-signaling in the brainstem which induces inhibition of food-intake. Activates MAPK- and AKT-signaling pathways. Isoform 1 in complex with GFRAL induces higher activation of MAPK-signaling pathway than isoform 2 in complex with GFRAL. | others | Pathways in cancer |
| PTEN | phosphatase and tensin homolog | Tumor suppressor. Acts as a dual-specificity protein phosphatase, dephosphorylating tyrosine-, serine- and threonine-phosphorylated proteins. | cellular senescence | p53 signaling pathway |
| TP73 | tumor protein p73 | TP73 participates in the apoptotic response to DNA damage. TP73 isoforms containing the transactivation domain are pro-apoptotic, isoforms lacking the domain are anti-apoptotic and block the function of p53 and transactivating p73 isoforms. TP73 may be a tumor suppressor protein. | genomic instability | p53 signaling pathway |
| PPM1D | protein phosphatase, Mg2+/Mn2+ dependent, 1D | It is required for the relief of p53-dependent checkpoint mediated cell cycle arrest. It binds to and dephosphorylates 'Ser-15' of TP53 and 'Ser-345' of CHEK1 which contributes to the functional inactivation of these proteins. It is also an important regulator of global heterochromatin silencing and critical in maintaining genome integrity. | genomic instability | p53 signaling pathway |
| CHEK2 | checkpoint kinase 2 | Serine/threonine-protein kinase which is required for checkpoint-mediated cell cycle arrest, activation of DNA repair and apoptosis in response to the presence of DNA double-strand breaks. May also negatively regulate cell cycle progression during unperturbed cell cycles. May also phosphorylate NEK6 which is involved in G2/M cell cycle arrest. Regulates DNA repair through phosphorylation of BRCA2, enhancing the association of RAD51 with chromatin which promotes DNA repair by homologous recombination. Also stimulates the transcription of genes involved in DNA repair (including BRCA2) through the phosphorylation and activation of the transcription factor FOXM1. Regulates apoptosis through the phosphorylation of p53/TP53, MDM4 and PML. Also controls the transcription of pro-apoptotic genes through phosphorylation of the transcription factor E2F1. | genomic instability | p53 signaling pathway |
| CYCS | Cytochrome C | Plays a role in apoptosis. Suppression of the anti-apoptotic members or activation of the pro-apoptotic members of the Bcl-2 family leads to altered mitochondrial membrane permeability resulting in release of cytochrome c into the cytosol. Binding of cytochrome c to Apaf-1 triggers the activation of caspase-9, which then accelerates apoptosis by activating other caspases. | cellular senescence | p53 signaling pathway |
| FAS | fas cell surface death receptor | Fas-mediated apoptosis has a role in aging of human epidermis. Epidermal Fas expression and apoptosis are increased in aged human skin. | others | p53 signaling pathway |
| ATM | ATM serine/threonine kinase | Serine/threonine protein kinase which activates checkpoint signaling upon double strand breaks (DSBs), apoptosis and genotoxic stresses such as ionizing ultraviolet A light (UVA), thereby acting as a DNA damage sensor. | genomic instability | p53 signaling pathway |
| ATR | ATR serine/threonine kinase | ATR is involved in DNA repair by activating checkpoint signaling during genotoxic stresses including the phosphorylation of ageing-related proteins such as BRCA1 and TP53. | stem cell exhaustion | p53 signaling pathway |
| BCL2 | B-cell CLL/lymphoma 2 | Suppresses apoptosis in a variety of cell systems including factor-dependent lymphohematopoietic and neural cells. Regulates cell death by controlling the mitochondrial membrane permeability. | others | p53 signaling pathway |
| CDK1 | cyclin-dependent kinase 1 | An important regulator of the cell cycle, CDK1 also appears to be involved in apoptosis. | cellular senescence | p53 signaling pathway |
| CDKN1A | cyclin-dependent kinase inhibitor 1A (p21, Cip1) | May be involved in p53/TP53 mediated inhibition of cellular proliferation in response to DNA damage. Binds to and inhibits cyclin-dependent kinase activity, preventing phosphorylation of critical cyclin-dependent kinase substrates and blocking cell cycle progression. Functions in the nuclear localization and assembly of cyclin D-CDK4 complex and promotes its kinase activity towards RB1. At higher stoichiometric ratios, inhibits the kinase activity of the cyclin D-CDK4 complex. Inhibits DNA synthesis by DNA polymerase delta by competing with POLD3 for PCNA binding. Plays an important role in controlling cell cycle progression and DNA damage-induced G2 arrest. | genomic instability | p53 signaling pathway |
| CDKN2A | cyclin-dependent kinase inhibitor 2A | The CDKN2A gene encodes different transcripts involved mostly in cell cycle regulation and cellular senescence, including the tumour suppressor proteins p16 and p19. | cellular senescence | p53 signaling pathway |
| IGFBP3 | insulin-like growth factor binding protein 3 | IGF-binding proteins prolong the half-life of the IGFs and have been shown to either inhibit or stimulate the growth promoting effects of the IGFs on cell culture. | cellular senescence | p53 signaling pathway |
| MDM2 | MDM2 proto-oncogene, E3 ubiquitin protein ligase | MDM2 is an oncogene that inhibits TP53. The MDM2 gene encodes multiple transcripts, many of which are tissue-specific. Disruption of MDM2 in mice resulted in increased TP53-dependent apoptosis and defects in multiple haematopoietic lineages. | cellular senescence | p53 signaling pathway |
| SERPINE1 | serpin peptidase inhibitor, clade E (nexin, plasminogen activator inhibitor type 1), member 1 | Serine protease inhibitor. SERPINE1 inhibits TMPRSS7. It is a primary inhibitor of tissue-type plasminogen activator (PLAT) and urokinase-type plasminogen activator (PLAU). As PLAT inhibitor, it is required for fibrinolysis down-regulation and is responsible for the controlled degradation of blood clots. As PLAU inhibitor, it is involved in the regulation of cell adhesion and spreading. | cellular senescence | p53 signaling pathway |
| NCOR2 | nuclear receptor corepressor 2 | It mediates the transcriptional repression activity of some nuclear receptors by promoting chromatin condensation, thus preventing access of the basal transcription. Isoform 1 and isoform 4 have different affinities for different nuclear receptors. It is involved in the regulation BCL6-dependent of the germinal center (GC) reactions, mainly through the control of the GC B-cells proliferation and survival. | others | Notch signaling pathway |
| DLL3 | delta-like 3 (Drosophila) | DLL3 has been linked to development and neurogenesis. | stem cell exhaustion | Notch signaling pathway |
| PLAU | plasminogen activator, urokinase | PLAU specifically cleaves the zymogen plasminogen to form the active enzyme plasmin. | others | NF-kappa B signaling pathway |
| PLCG1 | Phospholipase C Gamma 1 | Mediates the production of the second messenger molecules diacylglycerol (DAG) and inositol 1,4,5-trisphosphate (IP3). Plays an important role in the regulation of intracellular signaling cascades. Becomes activated in response to ligand-mediated activation of receptor-type tyrosine kinases, such as PDGFRA, PDGFRB, FGFR1, FGFR2, FGFR3 and FGFR4. Plays a role in actin reorganization and cell migration. | NF-κB related gene | NF-kappa B signaling pathway |
| PRKCB | Protein Kinase C Beta | Calcium-activated, phospholipid- and diacylglycerol (DAG)-dependent serine/threonine-protein kinase involved in various cellular processes such as regulation of the B-cell receptor (BCR) signalosome, oxidative stress-induced apoptosis, androgen receptor-dependent transcription regulation, insulin signaling and endothelial cells proliferation. Plays a key role in B-cell activation by regulating BCR-induced NF-kappa-B activation. Mediates the activation of the canonical NF-kappa-B pathway (NFKB1) by direct phosphorylation of CARD11/CARMA1 at 'Ser-559', 'Ser-644' and 'Ser-652'. Phosphorylation induces CARD11/CARMA1 association with lipid rafts and recruitment of the BCL10-MALT1 complex as well as MAP3K7/TAK1, which then activates IKK complex, resulting in nuclear translocation and activation of NFKB1. | NF-κB related gene | NF-kappa B signaling pathway |
| PRKCQ | Protein Kinase C Theta | Calcium-independent, phospholipid- and diacylglycerol (DAG)-dependent serine/threonine-protein kinase that mediates non-redundant functions in T-cell receptor (TCR) signaling, including T-cells activation, proliferation, differentiation and survival, by mediating activation of multiple transcription factors such as NF-kappa-B, JUN, NFATC1 and NFATC2. In TCR-CD3/CD28-co-stimulated T-cells, is required for the activation of NF-kappa-B and JUN, which in turn are essential for IL2 production, and participates in the calcium-dependent NFATC1 and NFATC2 transactivation. Mediates the activation of the canonical NF-kappa-B pathway (NFKB1) by direct phosphorylation of CARD11 on several serine residues, inducing CARD11 association with lipid rafts and recruitment of the BCL10-MALT1 complex, which then activates IKK complex, resulting in nuclear translocation and activation of NFKB1. | NF-κB related gene | NF-kappa B signaling pathway |
| RELB | RELB Proto-Oncogene, NF-KB Subunit | NF-kappa-B is a pleiotropic transcription factor which is present in almost all cell types and is involved in many biological processes such as inflammation, immunity, differentiation, cell growth, tumorigenesis and apoptosis. NF-kappa-B is a homo- or heterodimeric complex formed by the Rel-like domain-containing proteins RELA/p65, RELB, NFKB1/p105, NFKB1/p50, REL and NFKB2/p52. The dimers bind at kappa-B sites in the DNA of their target genes and the individual dimers have distinct preferences for different kappa-B sites that they can bind with distinguishable affinity and specificity. Different dimer combinations act as transcriptional activators or repressors, respectively. | NF-κB related gene | NF-kappa B signaling pathway |
| CCL4 | C-C Motif Chemokine Ligand 4 | Monokine with inflammatory and chemokinetic properties. Binds to CCR5. One of the major HIV-suppressive factors produced by CD8+ T-cells. Recombinant MIP-1-beta induces a dose-dependent inhibition of different strains of HIV-1, HIV-2, and simian immunodeficiency virus (SIV). The processed form MIP-1-beta (3-69) retains the abilities to induce down-modulation of surface expression of the chemokine receptor CCR5 and to inhibit the CCR5-mediated entry of HIV-1 in T-cells. | NF-κB related gene | NF-kappa B signaling pathway |
| CCL13 | C-C Motif Chemokine Ligand 13 | Chemotactic factor that attracts monocytes, lymphocytes, basophils and eosinophils, but not neutrophils. Signals through CCR2B and CCR3 receptors. Plays a role in the accumulation of leukocytes at both sides of allergic and non-allergic inflammation. | senescence-associated secretory phenotype | NF-kappa B signaling pathway |
| CCL19 | C-C Motif Chemokine Ligand 19 | May play a role not only in inflammatory and immunological responses but also in normal lymphocyte recirculation and homing. May play an important role in trafficking of T-cells in thymus, and T-cell and B-cell migration to secondary lymphoid organs. Binds to chemokine receptor CCR7. Recombinant CCL19 shows potent chemotactic activity for T-cells and B-cells but not for granulocytes and monocytes. Binds to atypical chemokine receptor ACKR4 and mediates the recruitment of beta-arrestin (ARRB1/2) to ACKR4. | NF-κB related gene | NF-kappa B signaling pathway |
| CCL21 | C-C Motif Chemokine Ligand 21 | Inhibits hemopoiesis and stimulates chemotaxis. Chemotactic in vitro for thymocytes and activated T-cells, but not for B-cells, macrophages, or neutrophils. Shows preferential activity towards naive T-cells. | NF-κB related gene | NF-kappa B signaling pathway |
| CXCL12 | C-X-C Motif Chemokine Ligand 12 | Chemoattractant active on T-lymphocytes and monocytes but not neutrophils. Activates the C-X-C chemokine receptor CXCR4 to induce a rapid and transient rise in the level of intracellular calcium ions and chemotaxis. | senescence-associated secretory phenotype | NF-kappa B signaling pathway |
| SYK | Spleen Associated Tyrosine Kinase | Non-receptor tyrosine kinase which mediates signal transduction downstream of a variety of transmembrane receptors including classical immunoreceptors like the B-cell receptor (BCR). Regulates several biological processes including innate and adaptive immunity, cell adhesion, osteoclast maturation, platelet activation and vascular development. Assembles into signaling complexes with activated receptors at the plasma membrane via interaction between its SH2 domains and the receptor tyrosine-phosphorylated ITAM domains. The association with the receptor can also be indirect and mediated by adapter proteins containing ITAM or partial hemITAM domains. The phosphorylation of the ITAM domains is generally mediated by SRC subfamily kinases upon engagement of the receptor. More rarely signal transduction via SYK could be ITAM-independent. Direct downstream effectors phosphorylated by SYK include VAV1, PLCG1, PI-3-kinase, LCP2 and BLNK. | NF-κB related gene | NF-kappa B signaling pathway |
| MAP3K7 | mitogen-activated protein kinase kinase kinase 7 | MAP3K7 is implicated in the cellular response to internal and external environmental changes, playing a crucial role in the inflammation processes that accompany aging. | NF-κB related gene | NF-kappa B signaling pathway |
| TLR4 | Toll Like Receptor 4 | Cooperates with LY96 and CD14 to mediate the innate immune response to bacterial lipopolysaccharide (LPS). Acts via MYD88, TIRAP and TRAF6, leading to NF-kappa-B activation, cytokine secretion and the inflammatory response. Also involved in LPS-independent inflammatory responses triggered by free fatty acids, such as palmitate, and Ni (2+). Responses triggered by Ni (2+) require non-conserved histidines and are, therefore, species-specific. Both M.tuberculosis HSP70 (dnaK) and HSP65 (groEL-2) act via this protein to stimulate NF-kappa-B expression. | NF-κB related gene | NF-kappa B signaling pathway |
| TNFAIP3 | TNF Alpha Induced Protein 3 | Ubiquitin-editing enzyme that contains both ubiquitin ligase and deubiquitinase activities. Involved in immune and inflammatory responses signaled by cytokines, such as TNF-alpha and IL-1 beta, or pathogens via Toll-like receptors (TLRs) through terminating NF-kappa-B activity. Essential component of a ubiquitin-editing protein complex, comprising also RNF11, ITCH and TAX1BP1, that ensures the transient nature of inflammatory signaling pathways. In cooperation with TAX1BP1 promotes disassembly of E2-E3 ubiquitin protein ligase complexes in IL-1R and TNFR-1 pathways; affected are at least E3 ligases TRAF6, TRAF2 and BIRC2, and E2 ubiquitin-conjugating enzymes UBE2N and UBE2D3. In cooperation with TAX1BP1 promotes ubiquitination of UBE2N and proteasomal degradation of UBE2N and UBE2D3. | NF-κB related gene | NF-kappa B signaling pathway |
| TRAF1 | TNF Receptor Associated Factor 1 | Adapter molecule that regulates the activation of NF-kappa-B and JNK. Plays a role in the regulation of cell survival and apoptosis. The heterotrimer formed by TRAF1 and TRAF2 is part of a E3 ubiquitin-protein ligase complex that promotes ubiquitination of target proteins, such as MAP3K14. The TRAF1/TRAF2 complex recruits the antiapoptotic E3 protein-ubiquitin ligases BIRC2 and BIRC3 to TNFRSF1B/TNFR2. | NF-κB related gene | NF-kappa B signaling pathway |
| TRAF2 | TNF Receptor Associated Factor 2 | Regulates activation of NF-kappa-B and JNK and plays a central role in the regulation of cell survival and apoptosis. Required for normal antibody isotype switching from IgM to IgG. Has E3 ubiquitin-protein ligase activity and promotes 'Lys-63'-linked ubiquitination of target proteins, such as BIRC3, RIPK1 and TICAM1. Is an essential constituent of several E3 ubiquitin-protein ligase complexes, where it promotes the ubiquitination of target proteins by bringing them into contact with other E3 ubiquitin ligases. Regulates BIRC2 and BIRC3 protein levels by inhibiting their autoubiquitination and subsequent degradation; this does not depend on the TRAF2 RING-type zinc finger domain. Plays a role in mediating activation of NF-kappa-B by EIF2AK2/PKR. In complex with BIRC2 or BIRC3, promotes ubiquitination of IKBKE. | NF-κB related gene | NF-kappa B signaling pathway |
| TRAF3 | TNF Receptor Associated Factor 3 | Regulates pathways leading to the activation of NF-kappa-B and MAP kinases, and plays a central role in the regulation of B-cell survival. Part of signaling pathways leading to the production of cytokines and interferon. Required for normal antibody isotype switching from IgM to IgG. Plays a role T-cell dependent immune responses. Plays a role in the regulation of antiviral responses. Is an essential constituent of several E3 ubiquitin-protein ligase complexes. May have E3 ubiquitin-protein ligase activity and promote 'Lys-63'-linked ubiquitination of target proteins. Inhibits activation of NF-kappa-B in response to LTBR stimulation. Inhibits TRAF2-mediated activation of NF-kappa-B. Down-regulates proteolytic processing of NFKB2, and thereby inhibits non-canonical activation of NF-kappa-B. Promotes ubiquitination and proteasomal degradation of MAP3K14. | NF-κB related gene | NF-kappa B signaling pathway |
| TRAF6 | TNF Receptor Associated Factor 6 | E3 ubiquitin ligase that, together with UBE2N and UBE2V1, mediates the synthesis of 'Lys-63'-linked-polyubiquitin chains conjugated to proteins, such as IKBKG, IRAK1, AKT1 and AKT2. Also mediates ubiquitination of free/unanchored polyubiquitin chain that leads to MAP3K7 activation. Leads to the activation of NF-kappa-B and JUN. May be essential for the formation of functional osteoclasts. Seems to also play a role in dendritic cells (DCs) maturation and/or activation. Represses c-Myb-mediated transactivation, in B-lymphocytes. Adapter protein that seems to play a role in signal transduction initiated via TNF receptor, IL-1 receptor and IL-17 receptor. Regulates osteoclast differentiation by mediating the activation of adapter protein complex 1 (AP-1) and NF-kappa-B, in response to RANK-L stimulation. Together with MAP3K8, mediates CD40 signals that activate ERK in B-cells and macrophages, and thus may play a role in the regulation of immunoglobulin production. | NF-κB related gene | NF-kappa B signaling pathway |
| VCAM1 | Vascular Cell Adhesion Molecule 1 | mportant in cell-cell recognition. Appears to function in leukocyte-endothelial cell adhesion. Interacts with integrin alpha-4/beta-1 (ITGA4/ITGB1) on leukocytes, and mediates both adhesion and signal transduction. The VCAM1/ITGA4/ITGB1 interaction may play a pathophysiologic role both in immune responses and in leukocyte emigration to sites of inflammation. | NF-κB related gene | NF-kappa B signaling pathway |
| ZAP70 | Zeta Chain Of T Cell Receptor Associated Protein Kinase 70 | Tyrosine kinase that plays an essential role in regulation of the adaptive immune response. Regulates motility, adhesion and cytokine expression of mature T-cells, as well as thymocyte development. Contributes also to the development and activation of primary B-lymphocytes. When antigen presenting cells (APC) activate T-cell receptor (TCR), a serie of phosphorylations lead to the recruitment of ZAP70 to the doubly phosphorylated TCR component CD247/CD3Z through ITAM motif at the plasma membrane. This recruitment serves to localization to the stimulated TCR and to relieve its autoinhibited conformation. Release of ZAP70 active conformation is further stabilized by phosphorylation mediated by LCK. | NF-κB related gene | NF-kappa B signaling pathway |
| TRIM25 | Tripartite Motif Containing 25 | unctions as a ubiquitin E3 ligase and as an ISG15 E3 ligase. Involved in innate immune defense against viruses by mediating ubiquitination of DDX58 and IFIH1. Mediates 'Lys-63'-linked polyubiquitination of the DDX58 N-terminal CARD-like region and may play a role in signal transduction that leads to the production of interferons in response to viral infection. Mediates 'Lys-63'-linked polyubiquitination of IFIH1. Promotes ISGylation of 14-3-3 sigma (SFN), an adapter protein implicated in the regulation of a large spectrum signaling pathway. Mediates estrogen action in various target organs. Mediates the ubiquitination and subsequent proteasomal degradation of ZFHX3. | NF-κB related gene | NF-kappa B signaling pathway |
| IKBKG | Inhibitor Of Nuclear Factor Kappa B Kinase Regulatory Subunit | Regulatory subunit of the IKK core complex which phosphorylates inhibitors of NF-kappa-B thus leading to the dissociation of the inhibitor/NF-kappa-B complex and ultimately the degradation of the inhibitor. Its binding to scaffolding polyubiquitin seems to play a role in IKK activation by multiple signaling receptor pathways. However, the specific type of polyubiquitin recognized upon cell stimulation (either 'Lys-63'-linked or linear polyubiquitin) and its functional importance is reported conflictingly. Also considered to be a mediator for TAX activation of NF-kappa-B. Could be implicated in NF-kappa-B-mediated protection from cytokine toxicity. Essential for viral activation of IRF3. Involved in TLR3- and IFIH1-mediated antiviral innate response; this function requires 'Lys-27'-linked polyubiquitination. | NF-κB related gene | NF-kappa B signaling pathway |
| TNFSF11 | TNF Superfamily Member 11 | Cytokine that binds to TNFRSF11B/OPG and to TNFRSF11A/RANK. Osteoclast differentiation and activation factor. Augments the ability of dendritic cells to stimulate naive T-cell proliferation. May be an important regulator of interactions between T-cells and dendritic cells and may play a role in the regulation of the T-cell-dependent immune response. May also play an important role in enhanced bone-resorption in humoral hypercalcemia of malignancy. Induces osteoclastogenesis by activating multiple signaling pathways in osteoclast precursor cells, chief among which is induction of long lasting oscillations in the intracellular concentration of Ca (2+) resulting in the activation of NFATC1, which translocates to the nucleus and induces osteoclast-specific gene transcription to allow differentiation of osteoclasts. During osteoclast differentiation, in a TMEM64 and ATP2A2-dependent manner induces activation of CREB1 and mitochondrial ROS generation necessary for proper osteoclast generation. | NF-κB related gene | NF-kappa B signaling pathway |
| TRADD | TNFRSF1A Associated Via Death Domain | The nuclear form acts as a tumor suppressor by preventing ubiquitination and degradation of isoform p19ARF/ARF of CDKN2A by TRIP12: acts by interacting with TRIP12, leading to disrupt interaction between TRIP12 and isoform p19ARF/ARF of CDKN2A (By similarity). Adapter molecule for TNFRSF1A/TNFR1 that specifically associates with the cytoplasmic domain of activated TNFRSF1A/TNFR1 mediating its interaction with FADD. Overexpression of TRADD leads to two major TNF-induced responses, apoptosis and activation of NF-kappa-B. | NF-κB related gene | NF-kappa B signaling pathway |
| RIPK1 | Receptor Interacting Serine/Threonine Kinase 1 | Serine-threonine kinase which is a key regulator of TNF-mediated apoptosis, necroptosis and inflammatory pathways (PubMed:25459879, PubMed:31827281, PubMed:31827280). Exhibits kinase activity-dependent functions that trigger cell death and kinase-independent scaffold functions regulating inflammatory signaling and cell survival (PubMed:11101870, PubMed:25459879). Initiates ripoptocide which describes cell death that is dependent on RIPK1, be it apoptosis or necroptosis (PubMed:31457011). Upon binding of TNF to TNFR1, RIPK1 is recruited to the TNF-R1 signaling complex (TNF-RSC also known as complex I) where it acts as a scaffold protein promoting cell survival, in part, by activating the canonical NF-kB pathway. | NF-κB related gene | NF-kappa B signaling pathway |
| TNFSF14 | TNF Superfamily Member 14 | Cytokine that binds to TNFRSF3/LTBR. Binding to the decoy receptor TNFRSF6B modulates its effects. Acts as a ligand for TNFRSF14/HVEM. Upon binding to TNFRSF14/HVEM, delivers costimulatory signals to T cells, leading to T cell proliferation and IFNG production. | NF-κB related gene | NF-kappa B signaling pathway |
| TNFRSF11A | TNF Receptor Superfamily Member 11a | Receptor for TNFSF11/RANKL/TRANCE/OPGL; essential for RANKL-mediated osteoclastogenesis. Involved in the regulation of interactions between T-cells and dendritic cells. | NF-κB related gene | NF-kappa B signaling pathway |
| CFLAR | CASP8 And FADD Like Apoptosis Regulator | Apoptosis regulator protein which may function as a crucial link between cell survival and cell death pathways in mammalian cells. Acts as an inhibitor of TNFRSF6 mediated apoptosis. | NF-κB related gene | NF-kappa B signaling pathway |
| BCL10 | BCL10 immune signaling adaptor | Involved in adaptive immune response (PubMed:25365219). Promotes apoptosis, pro-caspase-9 maturation and activation of NF-kappa-B via NIK and IKK. May be an adapter protein between upstream TNFR1-TRADD-RIP complex and the downstream NIK-IKK-IKAP complex. | NF-κB related gene | NF-kappa B signaling pathway |
| MAP3K14 | mitogen-activated protein kinase kinase kinase 14 | SNPs in MAP3K14 involved in the NF-κB signaling pathway influence bone mineral density, geometry and turnover in a population-based cohort of middle aged and elderly men. | NF-κB related gene | NF-kappa B signaling pathway |
| TAB1 | TGF-Beta Activated Kinase 1 (MAP3K7) Binding Protein 1 | May be an important signaling intermediate between TGFB receptors and MAP3K7/TAK1. May play an important role in mammalian embryogenesis. | NF-κB related gene | NF-kappa B signaling pathway |
| TNFSF13B | TNF Superfamily Member 13 | Cytokine that binds to TNFRSF13B/TACI and TNFRSF17/BCMA. TNFSF13/APRIL binds to the same 2 receptors. Together, they form a 2 ligands -2 receptors pathway involved in the stimulation of B- and T-cell function and the regulation of humoral immunity. A third B-cell specific BAFF-receptor (BAFFR/BR3) promotes the survival of mature B-cells and the B-cell response. | NF-κB related gene | NF-kappa B signaling pathway |
| TAB2 | TGF-Beta Activated Kinase 1 (MAP3K7) Binding Protein 2 | Adapter linking MAP3K7/TAK1 and TRAF6. Promotes MAP3K7 activation in the IL1 signaling pathway. The binding of 'Lys-63'-linked polyubiquitin chains to TAB2 promotes autophosphorylation of MAP3K7 at 'Thr-187'. Involved in heart development. | NF-κB related gene | NF-kappa B signaling pathway |
| DDX58 | DExD/H-box helicase 58 | Aging compromises both the primary and secondary RIG-I signaling pathways that govern expression of type I IFN genes, thereby impairing antiviral resistance to IAV. | NF-κB related gene | NF-kappa B signaling pathway |
| CARD10 | Caspase Recruitment Domain Family Member 10 | Activates NF-kappa-B via BCL10 and IKK. | NF-κB related gene | NF-kappa B signaling pathway |
| PIAS4 | Protein Inhibitor Of Activated STAT 4 | Functions as an E3-type small ubiquitin-like modifier (SUMO) ligase, stabilizing the interaction between UBE2I and the substrate, and as a SUMO-tethering factor. Plays a crucial role as a transcriptional coregulation in various cellular pathways, including the STAT pathway, the p53/TP53 pathway, the Wnt pathway and the steroid hormone signaling pathway. Involved in gene silencing. Mediates sumoylation of CEBPA, PARK7, HERC2, MYB, TCF4 and RNF168. In Wnt signaling, represses LEF1 and enhances TCF4 transcriptional activities through promoting their sumoylations. Enhances the sumoylation of MTA1 and may participate in its paralog-selective sumoylation. | NF-κB related gene | NF-kappa B signaling pathway |
| PIDD1 | P53-Induced Death Domain Protein 1 | Component of the DNA damage/stress response pathway that functions downstream of p53/TP53 and can either promote cell survival or apoptosis (PubMed:10973264, PubMed:15073321, PubMed:16360037, PubMed:17159900). Associated with CRADD and the CASP2 caspase, it forms the PIDDosome a complex that activates CASP2 and triggers apoptosis (PubMed:15073321, PubMed:17159900). Associated with IKBKG and RIPK1, it enhances sumoylation and ubiquitination of IKBKG which is important for activation of the transcription factor NF-kappa-B (PubMed:16360037, PubMed:17159900). | NF-κB related gene | NF-kappa B signaling pathway |
| PARP1 | poly (ADP-ribose) polymerase 1 | PARP1 is poly-ADP-ribosyltransferase that mediates poly-ADP-ribosylation of proteins and plays a key role in DNA repair. | genomic instability | NF-kappa B signaling pathway |
| BIRC2 | baculoviral IAP repeat containing 2 | Multi-functional protein which regulates not only caspases and apoptosis, but also modulates inflammatory signaling and immunity, mitogenic kinase signaling, and cell proliferation, as well as cell invasion and metastasis. | NF-κB related gene | NF-kappa B signaling pathway |
| BIRC3 | Baculoviral IAP Repeat Containing 3 | Multi-functional protein which regulates not only caspases and apoptosis, but also modulates inflammatory signaling and immunity, mitogenic kinase signaling and cell proliferation, as well as cell invasion and metastasis. | NF-κB related gene | NF-kappa B signaling pathway |
| XIAP | X-Linked Inhibitor Of Apoptosis | Multi-functional protein which regulates not only caspases and apoptosis, but also modulates inflammatory signaling and immunity, copper homeostasis, mitogenic kinase signaling, cell proliferation, as well as cell invasion and metastasis. Acts as a direct caspase inhibitor. Directly bind to the active site pocket of CASP3 and CASP7 and obstructs substrate entry. Inactivates CASP9 by keeping it in a monomeric, inactive state. Acts as an E3 ubiquitin-protein ligase regulating NF-kappa-B signaling and the target proteins for its E3 ubiquitin-protein ligase activity include: RIPK1, CASP3, CASP7, CASP8, CASP9, MAP3K2/MEKK2, DIABLO/SMAC, AIFM1, CCS and BIRC5/survivin. Ubiquitinion of CCS leads to enhancement of its chaperone activity toward its physiologic target, SOD1, rather than proteasomal degradation. Ubiquitinion of MAP3K2/MEKK2 and AIFM1 does not lead to proteasomal degradation. | NF-κB related gene | NF-kappa B signaling pathway |
| BCL2A1 | BCL2 related protein A1 | Retards apoptosis induced by IL-3 deprivation. May function in the response of hemopoietic cells to external signals and in maintaining endothelial survival during infection. | NF-κB related gene | NF-kappa B signaling pathway |
| BCL2L1 | BCL2 like 1 | Potent inhibitor of cell death. Inhibits activation of caspases. Appears to regulate cell death by blocking the voltage-dependent anion channel (VDAC) by binding to it and preventing the release of the caspase activator, CYC1, from the mitochondrial membrane. | NF-κB related gene | NF-kappa B signaling pathway |
| BTK | Bruton Tyrosine Kinase | Non-receptor tyrosine kinase indispensable for B lymphocyte development, differentiation and signaling. Binding of antigen to the B-cell antigen receptor (BCR) triggers signaling that ultimately leads to B-cell activation. After BCR engagement and activation at the plasma membrane, phosphorylates PLCG2 at several sites, igniting the downstream signaling pathway through calcium mobilization, followed by activation of the protein kinase C (PKC) family members. | NF-κB related gene | NF-kappa B signaling pathway |
| CD14 | CD14 Molecule | Coreceptor for bacterial lipopolysaccharide (PubMed:1698311, PubMed:23264655). In concert with LBP, binds to monomeric lipopolysaccharide and delivers it to the LY96/TLR4 complex, thereby mediating the innate immune response to bacterial lipopolysaccharide (LPS). | NF-κB related gene | NF-kappa B signaling pathway |
| CD40LG | CD40 Ligand | Cytokine that acts as a ligand to CD40/TNFRSF5. Costimulates T-cell proliferation and cytokine production. | NF-κB related gene | NF-kappa B signaling pathway |
| CXCL1 | C-X-C Motif Chemokine Ligand 1 | Has chemotactic activity for neutrophils. May play a role in inflammation and exerts its effects on endothelial cells in an autocrine fashion. | senescence-associated secretory phenotype | NF-kappa B signaling pathway |
| CXCL2 | C-X-C Motif Chemokine Ligand 2 | Produced by activated monocytes and neutrophils and expressed at sites of inflammation. | senescence-associated secretory phenotype | NF-kappa B signaling pathway |
| IL1R1 | interleukin 1 receptor type 1 | IL-1β during the early stages following chronic cerebral hypoperfusion impedes OPC recruitment via IL-1R1, which inhibits white matter repair and functional recovery. IL-1R1 inhibitors may have potential uses in the treatment of SIVD. | NF-κB related gene | NF-kappa B signaling pathway |
| NFKBIA | nuclear factor of kappa light polypeptide gene enhancer in B-cells inhibitor, alpha | NFKBIA may be involved in inflammation, apoptosis, differentiation, and growth. | deregulated nutrient sensing | NF-kappa B signaling pathway |
| EDARADD | EDAR associated death domain | The methylation of site--in the promoters of the EDARADD genes--is linear with age over a range of five decades, which could be a model to estimate the age of a person, based on a biological sample alone. | NF-κB related gene | NF-kappa B signaling pathway |
| TICAM1 | Toll Like Receptor Adaptor Molecule 1 | Functions as sorting adapter in different signaling pathways to facilitate downstream signaling leading to type I interferon induction. In TLR4 signaling, physically bridges TLR4 and TICAM1 and functionally transmits signal to TICAM1 in early endosomes after endocytosis of TLR4. In TLR2 signaling, physically bridges TLR2 and MYD88 and is required for the TLR2-dependent movement of MYD88 to endosomes following ligand engagement. Involved in IL-18 signaling and is proposed to function as a sorting adapter for MYD88 in IL-18 signaling during adaptive immune response. Forms a complex with RAB11FIP2 that is recruited to the phagosomes to promote the activation of the actin-regulatory GTPases RAC1 and CDC42 and subsequent phagocytosis of Gram-negative bacteria. | NF-κB related gene | NF-kappa B signaling pathway |
| TNFRSF13C | TNF Receptor Superfamily Member 13C | B-cell receptor specific for TNFSF13B/TALL1/BAFF/BLyS. Promotes the survival of mature B-cells and the B-cell response. | NF-κB related gene | NF-kappa B signaling pathway |
| TIRAP | TIR Domain Containing Adaptor Protein | Adapter involved in TLR2 and TLR4 signaling pathways in the innate immune response. Acts via IRAK2 and TRAF-6, leading to the activation of NF-kappa-B, MAPK1, MAPK3 and JNK, and resulting in cytokine secretion and the inflammatory response. Positively regulates the production of TNF-alpha and interleukin-6. | NF-κB related gene | NF-kappa B signaling pathway |
| RICTOR | RPTOR independent companion of MTOR, complex 2 | Subunit of mTORC2, which regulates cell growth and survival in response to hormonal signals. mTORC2 is activated by growth factors, but, in contrast to mTORC1, seems to be nutrient-insensitive. mTORC2 seems to function upstream of Rho GTPases to regulate the actin cytoskeleton, probably by activating one or more Rho-type guanine nucleotide exchange factors. | deregulated nutrient sensing | mTOR signaling pathway |
| PRKCA | protein kinase C, alpha | PRKCA is a phorbol ester receptor involved in signal transduction. Gain-of-function mutations in Protein Kinase Cα (PKCα) may promote synaptic defects in Alzheimer's disease. | others | mTOR signaling pathway |
| TNF | tumor necrosis factor | A cytokine involved in the immune response, TNF can induce cell death in some tumour cells. It can also induce cellular proliferation and differentiation. | altered intercellular communication | mTOR signaling pathway |
| WNT2 | Wnt Family Member | Ligand for members of the frizzled family of seven transmembrane receptors. Functions in the canonical Wnt signaling pathway that results in activation of transcription factors of the TCF/LEF family (PubMed:20018874). Functions as upstream regulator of FGF10 expression. Plays an important role in embryonic lung development. May contribute to embryonic brain development by regulating the proliferation of dopaminergic precursors and neurons (By similarity). | senescence-associated secretory phenotype | mTOR signaling pathway |
| UBB | ubiquitin B | UBB exists either covalently attached to another protein, or free (unanchored). | loss of proteostasis | Mitophagy - animal |
| PTGES | Prostaglandin E Synthase | Terminal enzyme of the cyclooxygenase (COX)-2-mediated prostaglandin E2 (PGE2) biosynthetic pathway. Catalyzes the glutathione-dependent oxidoreduction of prostaglandin endoperoxide H2 (PGH2) to prostaglandin E2 (PGE2) in response to inflammatory stimuli (PubMed:18682561, PubMed:10377395, PubMed:12672824, PubMed:12460774, PubMed:10869354, PubMed:12244105). Plays a key role in inflammation response, fever and pain (By similarity). Catalyzes also the oxidoreduction of endocannabinoids into prostaglandin glycerol esters and PGG2 into 15-hydroperoxy-PGE2 (PubMed:12244105, PubMed:12672824). In addition, displays low glutathione transferase and glutathione-dependent peroxidase activities, toward 1-chloro-2,4-dinitrobenzene and 5-hydroperoxyicosatetraenoic acid (5-HPETE), respectively (PubMed:12672824). | senescence-associated secretory phenotype | Metabolic pathways |
| FGF23 | fibroblast growth factor 23 | Regulator of phosphate homeostasis. Inhibits renal tubular phosphate transport by reducing SLC34A1 levels. Upregulates EGR1 expression in the presence of KL. Acts directly on the parathyroid to decrease PTH secretion. Regulator of vitamin-D metabolism. Negatively regulates osteoblast differentiation and matrix mineralization. | others | MAPK signaling pathway |
| RPS6KA5 | Ribosomal Protein S6 Kinase A5 | Serine/threonine-protein kinase that is required for the mitogen or stress-induced phosphorylation of the transcription factors CREB1 and ATF1 and for the regulation of the transcription factors RELA, STAT3 and ETV1/ER81, and that contributes to gene activation by histone phosphorylation and functions in the regulation of inflammatory genes (PubMed:11909979, PubMed:12569367, PubMed:12763138, PubMed:9687510, PubMed:18511904, PubMed:9873047). Phosphorylates CREB1 and ATF1 in response to mitogenic or stress stimuli such as UV-C irradiation, epidermal growth factor (EGF) and anisomycin. | senescence-associated secretory phenotype | MAPK signaling pathway |
| FGF21 | fibroblast growth factor 21 | Stimulates glucose uptake in differentiated adipocytes via the induction of glucose transporter SLC2A1/GLUT1 expression (but not SLC2A4/GLUT4 expression). Activity requires the presence of KLB. | deregulated nutrient sensing | MAPK signaling pathway |
| AR | androgen receptor | Androgen-AR plays key roles in the development of insulin and leptin resistance, which suggests that AR may contribute to certain age-related diseases such as type 2 diabetes and cardiovascular disease. | deregulated nutrient sensing | MAPK signaling pathway |
| AREG | amphiregulin | Ligand of the EGF receptor/EGFR. Autocrine growth factor as well as a mitogen for a broad range of target cells including astrocytes, Schwann cells and fibroblasts. | senescence-associated secretory phenotype | MAPK signaling pathway |
| BDNF | brain-derived neurotrophic factor | BDNF is a growth factor that promotes neuronal survival and is involved in numerous neuronal responses. | cellular senescence | MAPK signaling pathway |
| CACNA1A | calcium channel, voltage-dependent, P/Q type, alpha 1A subunit | CACNA1A mediates calcium ions in a variety of processes such as cell division and gene expression. | others | MAPK signaling pathway |
| CDC42 | cell division cycle 42 | Probably involved in the organization of the actin cytoskeleton by acting downstream of CDC42, inducing actin filament assembly. Alters CDC42-induced cell shape changes. In activated T-cells, may play a role in CDC42-mediated F-actin accumulation at the immunological synapse. May play a role in early contractile events in phagocytosis in macrophages. | others | MAPK signaling pathway |
| DDIT3 | DNA-damage-inducible transcript 3 | DDIT3 prevents transcription in response to DNA damage. It also appears to be related to apoptosis. | genomic instability | MAPK signaling pathway |
| EGF | epidermal growth factor | EGF stimulates the growth of various epidermal and epithelial tissues in vivo and in vitro and of some fibroblasts in cell culture. | cellular senescence | MAPK signaling pathway |
| EGFR | epidermal growth factor receptor | Receptor tyrosine kinase binding ligands of the EGF family and activating several signaling cascades to convert extracellular cues into appropriate cellular responses. | cellular senescence | MAPK signaling pathway |
| ERBB2 | erb-b2 receptor tyrosine kinase 2 | Protein tyrosine kinase that is part of several cell surface receptor complexes, but that apparently needs a coreceptor for ligand binding. Essential component of a neuregulin-receptor complex, although neuregulins do not interact with it alone. | others | MAPK signaling pathway |
| FGF7 | fibroblast growth factor 7 | Hominis Placenta (HP) treatment significantly increased the expression of FGF-7, which plays pivotal roles to maintain anagen phase both protein and mRNA levels. | senescence-associated secretory phenotype | MAPK signaling pathway |
| FGFR1 | fibroblast growth factor receptor 1 | FGFR1 is the receptor of the fibroblast growth factor. | altered intercellular communication | MAPK signaling pathway |
| FGFR3 | Fibroblast Growth Factor Receptor 3 | Tyrosine-protein kinase that acts as cell-surface receptor for fibroblast growth factors and plays an essential role in the regulation of cell proliferation, differentiation and apoptosis. Plays an essential role in the regulation of chondrocyte differentiation, proliferation and apoptosis, and is required for normal skeleton development. Regulates both osteogenesis and postnatal bone mineralization by osteoblasts. Promotes apoptosis in chondrocytes, but can also promote cancer cell proliferation. Required for normal development of the inner ear. Phosphorylates PLCG1, CBL and FRS2. Ligand binding leads to the activation of several signaling cascades. Activation of PLCG1 leads to the production of the cellular signaling molecules diacylglycerol and inositol 1,4,5-trisphosphate. Phosphorylation of FRS2 triggers recruitment of GRB2, GAB1, PIK3R1 and SOS1, and mediates activation of RAS, MAPK1/ERK2, MAPK3/ERK1 and the MAP kinase signaling pathway, as well as of the AKT1 signaling pathway. Plays a role in the regulation of vitamin D metabolism. Mutations that lead to constitutive kinase activation or impair normal FGFR3 maturation, internalization and degradation lead to aberrant signaling. Over-expressed or constitutively activated FGFR3 promotes activation of PTPN11/SHP2, STAT1, STAT5A and STAT5B. Secreted isoform 3 retains its capacity to bind FGF1 and FGF2 and hence may interfere with FGF signaling. | others | MAPK signaling pathway |
| HSPA1A | heat shock 70kDa protein 1A, tissue-specific | Ensuring the correct folding of proteins. Acts as a cellular defense mechanism,the aging-associated variation of the levels of Hsp70 followed a different pattern in post-mitotic and mitotic tissues, being lower or higher in old comparing to adults, respectively. | loss of proteostasis | MAPK signaling pathway |
| HSPA1B | heat shock 70kDa protein 1B | Stress inducible, ATPase activity, tolerance of hyperthermia,ischemia/hypoxia, resistance to oxidative, UV and TNF stresses,protection against protein aggregation, regulation of HS response,protection of transcription/translation, tumorigenicity,antiapoptotic. | loss of proteostasis | MAPK signaling pathway |
| HSPA8 | heat shock 70kDa protein 8 | This gene encodes a member of the heat shock protein 70 family, which contains both heat-inducible and constitutively expressed members. This protein belongs to the latter group, which are also referred to as heat-shock cognate proteins. It functions as a chaperone, and binds to nascent polypeptides to facilitate correct folding. It also functions as an ATPase in the disassembly of clathrin-coated vesicles during transport of membrane components through the cell. | loss of proteostasis | MAPK signaling pathway |
| IGF2 | insulin-like growth factor 2 | The insulin-like growth factors possess growth-promoting activity. Major fetal growth hormone in mammals. Plays a key role in regulating fetoplacental development. | cellular senescence | MAPK signaling pathway |
| IL1B | interleukin 1 beta | Aged adipose B cells (AABs) express IL-1R, and inhibition of IL-1 signaling reduces their proliferation and increases lipolysis in aging. | senescence-associated secretory phenotype | MAPK signaling pathway |
| JUND | jun D proto-oncogene | His protein has been proposed to protect cells from p53-dependent senescence and apoptosis. Alternative translation initiation site usage results in the production of different isoforms. | cellular senescence | MAPK signaling pathway |
| MAPT | microtubule-associated protein tau | Promotes microtubule assembly and stability, and might be involved in the establishment and maintenance of neuronal polarity. | loss of proteostasis | MAPK signaling pathway |
| MAX | MYC associated factor X | A transcriptional regulator, MAX can act as pro- or anti-apoptotic. | cellular senescence | MAPK signaling pathway |
| MAP3K5 | mitogen-activated protein kinase kinase kinase 5 | It is involved in stress response and apoptosis. | cellular senescence | MAPK signaling pathway |
| MYD88 | Myeloid Differentiation Primary Response 88 | Adapter protein involved in the Toll-like receptor and IL-1 receptor signaling pathway in the innate immune response (PubMed:15361868, PubMed:18292575). Acts via IRAK1, IRAK2, IRF7 and TRAF6, leading to NF-kappa-B activation, cytokine secretion and the inflammatory response (PubMed:15361868, PubMed:24316379, PubMed:19506249). Increases IL-8 transcription (PubMed:9013863). Involved in IL-18-mediated signaling pathway. Activates IRF1 resulting in its rapid migration into the nucleus to mediate an efficient induction of IFN-beta, NOS2/INOS, and IL12A genes. MyD88-mediated signaling in intestinal epithelial cells is crucial for maintenance of gut homeostasis and controls the expression of the antimicrobial lectin REG3G in the small intestine (By similarity). | others | MAPK signaling pathway |
| NFKB2 | nuclear factor of kappa light polypeptide gene enhancer in B-cells 2 (p49/p100) | NF-kappa-B is a pleiotropic transcription factor present in almost all cell types and is the endpoint of a series of signal transduction events that are initiated by a vast array of stimuli related to many biological processes such as inflammation, immunity, differentiation, cell growth, tumorigenesis and apoptosis. | others | MAPK signaling pathway |
| NGF | nerve growth factor (beta polypeptide) | Nerve growth factor is important for the development and maintenance of the sympathetic and sensory nervous systems. | cellular senescence | MAPK signaling pathway |
| NGFR | nerve growth factor receptor | Low affinity receptor which can bind to NGF, BDNF, NTF3, and NTF4. Forms a heterodimeric receptor with SORCS2 that binds the precursor forms of NGF, BDNF and NTF3 with high affinity, and has much lower affinity for mature NGF and BDNF. Plays an important role in differentiation and survival of specific neuronal populations during development. | cellular senescence | MAPK signaling pathway |
| PDGFB | platelet-derived growth factor beta polypeptide | It is a growth factor that plays an essential role in the regulation of embryonic development, cell proliferation, cell migration, survival and chemotaxis. | cellular senescence | MAPK signaling pathway |
| PDGFRA | platelet-derived growth factor receptor, alpha polypeptide | Tyrosine-protein kinase that acts as a cell-surface receptor for PDGFA, PDGFB and PDGFC and plays an essential role in the regulation of embryonic development, cell proliferation, survival and chemotaxis. Depending on the context, promotes or inhibits cell proliferation and cell migration. It plays an important role in the differentiation of bone marrow-derived mesenchymal stem cells. It is required for normal skeleton development and cephalic closure during embryonic development. Required for normal development of the mucosa lining the gastrointestinal tract, and for recruitment of mesenchymal cells and normal development of intestinal villi. | altered intercellular communication | MAPK signaling pathway |
| PDGFRB | platelet-derived growth factor receptor, beta polypeptide | PDGFRB is a tyrosine-protein kinase that acts as cell-surface receptor for homodimeric PDGFB and PDGFD and for heterodimers formed by PDGFA and PDGFB, and plays an essential role in the regulation of embryonic development, cell proliferation, survival, differentiation, chemotaxis and migration. | others | MAPK signaling pathway |
| ADCY1 | Adenylate cyclase type 1 | Catalyzes the formation of the signaling molecule cAMP in response to G-protein signaling. Mediates responses to increased cellular Ca2+/calmodulin levels. May be involved in regulatory processes in the central nervous system. May play a role in memory and learning. Plays a role in the regulation of the circadian rhythm of daytime contrast sensitivity probably by modulating the rhythmic synthesis of cyclic AMP in the retina. | altered intercellular communication | Longevity regulating pathway |
| ADCY2 | Adenylate cyclase type 2 | Catalyzes the formation of the signaling molecule cAMP in response to G-protein signaling. Down-stream signaling cascades mediate changes in gene expression patterns and lead to increased IL6 production. Functions in signaling cascades downstream of the muscarinic acetylcholine receptors. | altered intercellular communication | Longevity regulating pathway |
| ADCY3 | Adenylate cyclase type 3 | Catalyzes the formation of the signaling molecule cAMP in response to G-protein signaling. Participates in signaling cascades triggered by odorant receptors via its function in cAMP biosynthesis. Required for the perception of odorants. Required for normal sperm motility and normal male fertility. Plays a role in regulating insulin levels and body fat accumulation in response to a high fat diet. | altered intercellular communication | Longevity regulating pathway |
| ADCY5 | adenylate cyclase 5 | Catalyzes the formation of the signaling molecule cAMP in response to G-protein signaling. Mediates signaling downstream of ADRB1. Regulates the increase of free cytosolic Ca2+ in response to increased blood glucose levels and contributes to the regulation of Ca2+-dependent insulin secretion. | altered intercellular communication | Longevity regulating pathway |
| ADCY6 | Adenylate cyclase type 6 | Catalyzes the formation of the signaling molecule cAMP downstream of G protein-coupled receptors. Functions in signaling cascades downstream of beta-adrenergic receptors in the heart and in vascular smooth muscle cells. Functions in signaling cascades downstream of the vasopressin receptor in the kidney and has a role in renal water reabsorption. Functions in signaling cascades downstream of PTH1R and plays a role in regulating renal phosphate excretion. Functions in signaling cascades downstream of the VIP and SCT receptors in pancreas and contributes to the regulation of pancreatic amylase and fluid secretion. Signaling mediates cAMP-dependent activation of protein kinase PKA. This promotes increased phosphorylation of various proteins, including AKT. Plays a role in regulating cardiac sarcoplasmic reticulum Ca2+ uptake and storage, and is required for normal heart ventricular contractibility. | altered intercellular communication | Longevity regulating pathway |
| ADCY7 | Adenylate cyclase type 7 | Catalyzes the formation of cAMP in response to activation of G protein-coupled receptors. Functions in signaling cascades activated namely by thrombin and sphingosine 1-phosphate and mediates regulation of cAMP synthesis through synergistic action of the stimulatory G alpha protein with GNA13. Also, during inflammation, mediates zymosan-induced increase intracellular cAMP, leading to protein kinase A pathway activation in order to modulate innate immune responses through heterotrimeric G proteins G (12/13). Functions in signaling cascades activated namely by dopamine and C5 alpha chain and mediates regulation of cAMP synthesis through synergistic action of the stimulatory G protein with G beta:gamma complex. | altered intercellular communication | Longevity regulating pathway |
| ADCY8 | Adenylate cyclase type 8 | Catalyzes the formation of cAMP in response to calcium entry leadings to cAMP signaling activation that affect processes suche as synaptic plasticity and insulin secretion. Plays a role in many brain functions, such as learning, memory, drug addiction, and anxiety modulation through regulation of synaptic plasticity by modulating long-term memory and long-term potentiation (LTP) through CREB transcription factor activity modulation. Plays a central role in insulin secretion by controlling glucose homeostasis through glucagon-like peptide 1 and glucose signaling pathway and maintains insulin secretion through calcium-dependent PKA activation leading to vesicle pool replenishment. Also, allows PTGER3 to induce potentiation of PTGER4-mediated PLA2 secretion by switching from a negative to a positive regulation, during the IL1B induced-dedifferentiation of smooth muscle cells. | altered intercellular communication | Longevity regulating pathway |
| ADCY9 | Adenylate cyclase type 9 | Adenylyl cyclase that catalyzes the formation of the signaling molecule cAMP in response to activation of G protein-coupled receptors. Contributes to signaling cascades activated by CRH (corticotropin-releasing factor), corticosteroids and beta-adrenergic receptors. | altered intercellular communication | Longevity regulating pathway |
| AKT1 | v-akt murine thymoma viral oncogene homolog 1 | AKT1 is one of 3 closely related serine/threonine-protein kinases (AKT1, AKT2 and AKT3) called the AKT kinase, and which regulate many processes including metabolism, proliferation, cell survival, growth and angiogenesis. | cellular senescence | Longevity regulating pathway |
| AKT2 | RAC-beta serine/threonine-protein kinase | AKT2 is one of 3 closely related serine/threonine-protein kinases (AKT1, AKT2 and AKT3) called the AKT kinase, and which regulate many processes including metabolism, proliferation, cell survival, growth and angiogenesis. AKT mediates insulin-stimulated protein synthesis by phosphorylating TSC2 at 'Ser-939' and 'Thr-1462', thereby activating mTORC1 signaling and leading to both phosphorylation of 4E-BP1 and in activation of RPS6KB1. AKT is involved in the phosphorylation of members of the FOXO factors (Forkhead family of transcription factors), leading to binding of 14-3-3 proteins and cytoplasmic localization. AKT phosphorylates 'Ser-454' on ATP citrate lyase (ACLY), thereby potentially regulating ACLY activity and fatty acid synthesis. AKT plays a role as key modulator of the AKT-mTOR signaling pathway controlling the tempo of the process of newborn neurons integration during adult neurogenesis, including correct neuron positioning, dendritic development and synapse formation. | deregulated nutrient sensing | Longevity regulating pathway |
| ATF4 | Cyclic AMP-dependent transcription factor ATF-4 | Transcription factor that binds the cAMP response element (CRE) (consensus: 5'-GTGACGT[AC][AG]-3') and acts both as a regulator of normal metabolic and redox processes, and as a master transcription factor during the integrated stress response (ISR). Binds to asymmetric CRE's as a heterodimer and to palindromic CRE's as a homodimer. Core effector of the ISR, which is required for adaptation to various stress, such as endoplasmic reticulum (ER) stress, amino acid starvation, mitochondrial stress or oxidative stress. Protects cells against metabolic consequences of ER oxidation by promoting expression of genes linked to amino acid sufficiency and resistance to oxidative stress. Regulates the induction of DDIT3/CHOP and asparagine synthetase (ASNS) in response to amino acid deprivation or endoplasmic reticulum (ER) stress. Together with DDIT3/CHOP, mediates ER-mediated cell death by promoting expression of genes involved in cellular amino acid metabolic processes, mRNA translation and the unfolded protein response (UPR) in response to ER stress. | others | Longevity regulating pathway |
| BAX | BCL2-associated X protein | BAX is an important player in apoptosis. | others | Longevity regulating pathway |
| CAMK4 | calcium/calmodulin-dependent protein kinase type IV | Calcium/calmodulin-dependent protein kinase that operates in the calcium-triggered CaMKK-CaMK4 signaling cascade and regulates, mainly by phosphorylation, the activity of several transcription activators, such as CREB1, MEF2D, JUN and RORA, which play pivotal roles in immune response, inflammation, and memory consolidation. In the thymus, regulates the CD4+/CD8+ double positive thymocytes selection threshold during T-cell ontogeny. In CD4 memory T-cells, is required to link T-cell antigen receptor (TCR) signaling to the production of IL2, IFNG and IL4 (through the regulation of CREB and MEF2). Regulates the differentiation and survival phases of osteoclasts and dendritic cells (DCs). Mediates DCs survival by linking TLR4 and the regulation of temporal expression of BCL2. | others | Longevity regulating pathway |
| CAT | catalase | Cardiac-specific overexpression prolongs lifespan in mice. Catalase protects cardiomyocytes from ageing-induced contractile defects and protein damage. | others | Longevity regulating pathway |
| CREB1 | cAMP responsive element binding protein 1 | This gene encodes a transcription factor that is a member of the leucine zipper family of DNA binding proteins. This protein binds as a homodimer to the cAMP-responsive element, an octameric palindrome. | genomic instability | Longevity regulating pathway |
| ATF2 | activating transcription factor 2 | TF2 is an important transcription factor involved in a variety of functions that may also be involved in oxidative stress response and cellular growth arrest and senescence. | cellular senescence | Longevity regulating pathway |
| ATF6B | Cyclic AMP-dependent transcription factor ATF-6 beta | Transcriptional factor that acts in the unfolded protein response (UPR) pathway by activating UPR target genes induced during ER stress. Binds DNA on the 5'-CCAC[GA]-3' half of the ER stress response element (ERSE) (5'-CCAATN9CCAC[GA]-3') when NF-Y is bound to ERSE. | loss of proteostasis | Longevity regulating pathway |
| EIF4E | eukaryotic translation initiation factor 4E | Recognizes and binds the 7-methylguanosine-containing mRNA cap during an early step in the initiation of protein synthesis and facilitates ribosome binding by inducing the unwinding of the mRNAs secondary structures. Component of the CYFIP1-EIF4E-FMR1 complex which binds to the mRNA cap and mediates translational repression. In the CYFIP1-EIF4E-FMR1 complex this subunit mediates the binding to the mRNA cap. | others | Longevity regulating pathway |
| EIF4EBP1 | eukaryotic translation initiation factor 4E-binding protein 1 | Repressor of translation initiation that regulates EIF4E activity by preventing its assembly into the eIF4F complex: hypophosphorylated form competes with EIF4G1/EIF4G3 and strongly binds to EIF4E, leading to repress translation. In contrast, hyperphosphorylated form dissociates from EIF4E, allowing interaction between EIF4G1/EIF4G3 and EIF4E, leading to initiation of translation. Mediates the regulation of protein translation by hormones, growth factors and other stimuli that signal through the MAP kinase and mTORC1 pathways. | others | Longevity regulating pathway |
| FOXO1 | forkhead box O1 | FOXO1 negatively regulates skeletal muscle mass and type I fiber gene expression and leads to impaired skeletal muscle function. | others | Longevity regulating pathway |
| FOXO3 | forkhead box O3 | A transcription factor of the Fox family, FOXO3, is crucial in development. | others | Longevity regulating pathway |
| MTOR | mechanistic target of rapamycin (serine/threonine kinase) | The MTOR kinase belongs to the target of rapamycin group of enzymes which regulate cellular growth and proliferation. In human cell cultures MTOR inhibition supresses the senescence associated secretory phenotype (SASP), which can disrupt tissues and contribute to age-related pathologies, including cancer. | deregulated nutrient sensing | Longevity regulating pathway |
| HRAS | harvey rat sarcoma viral oncogene homolog | It is involved in the activation of Ras protein signal transduction. | cellular senescence | Longevity regulating pathway |
| IGF1 | insulin-like growth factor 1 (somatomedin C) | The insulin-like growth factors, isolated from plasma, are structurally and functionally related to insulin but have a much higher growth-promoting activity. | cellular senescence | Longevity regulating pathway |
| IGF1R | insulin-like growth factor 1 receptor | Receptor tyrosine kinase which mediates actions of insulin-like growth factor 1 (IGF1). Binds IGF1 with high affinity and IGF2 and insulin (INS) with a lower affinity. | others | Longevity regulating pathway |
| INS | insulin | Insulin decreases blood glucose concentration. It increases cell permeability to monosaccharides, amino acids and fatty acids. | cellular senescence | Longevity regulating pathway |
| INSR | insulin receptor | Receptor tyrosine kinase which mediates the pleiotropic actions of insulin. Binding of insulin leads to phosphorylation of several intracellular substrates, including, insulin receptor substrates (IRS1, 2, 3, 4), SHC, GAB1, CBL and other signaling intermediates. | cellular senescence | Longevity regulating pathway |
| IRS1 | insulin receptor substrate 1 | May mediate the control of various cellular processes by insulin. | cellular senescence | Longevity regulating pathway |
| NFKB1 | nuclear factor of kappa light polypeptide gene enhancer in B-cells 1 | Chronic, progressive low-grade inflammation induced by knockout of the nfkb1 subunit of the transcription factor NF-κB induces premature ageing. | altered intercellular communication | Longevity regulating pathway |
| NRAS | GTPase Nras | Ras proteins bind GDP/GTP and possess intrinsic GTPase activity. | others | Longevity regulating pathway |
| CREB3L1 | cyclic AMP-responsive element-binding protein 3-like protein 1 | Transcription factor involved in unfolded protein response (UPR). Binds the DNA consensus sequence 5'-GTGXGCXGC-3'. In the absence of endoplasmic reticulum (ER) stress, inserted into ER membranes, with N-terminal DNA-binding and transcription activation domains oriented toward the cytosolic face of the membrane. In response to ER stress, transported to the Golgi, where it is cleaved in a site-specific manner by resident proteases S1P/MBTPS1 and S2P/MBTPS2. The released N-terminal cytosolic domain is translocated to the nucleus to effect transcription of specific target genes. Plays a critical role in bone formation through the transcription of COL1A1, and possibly COL1A2, and the secretion of bone matrix proteins. Directly binds to the UPR element (UPRE)-like sequence in an osteoblast-specific COL1A1 promoter region and induces its transcription. Does not regulate COL1A1 in other tissues, such as skin. | loss of proteostasis | Longevity regulating pathway |
| EHMT1 | histone-lysine N-methyltransferase EHMT1 | Histone methyltransferase that specifically mono- and dimethylates 'Lys-9' of histone H3 (H3K9me1 and H3K9me2, respectively) in euchromatin. H3K9me represents a specific tag for epigenetic transcriptional repression by recruiting HP1 proteins to methylated histones. Also weakly methylates 'Lys-27' of histone H3 (H3K27me). Also required for DNA methylation, the histone methyltransferase activity is not required for DNA methylation, suggesting that these 2 activities function independently. Probably targeted to histone H3 by different DNA-binding proteins like E2F6, MGA, MAX and/or DP1. During G0 phase, it probably contributes to silencing of MYC- and E2F-responsive genes, suggesting a role in G0/G1 transition in cell cycle. In addition to the histone methyltransferase activity, also methylates non-histone proteins: mediates dimethylation of 'Lys-373' of p53/TP53. | epigenetic alterations | Longevity regulating pathway |
| SESN3 | Sestrin-3 | SESN3 may function as an intracellular leucine sensor that negatively regulates the TORC1 signaling pathway. SESN3 may also regulate the insulin-receptor signaling pathway through activation of TORC2. This metabolic regulator may also play a role in protection against oxidative and genotoxic stresses. | altered intercellular communication | Longevity regulating pathway |
| CREB3L3 | cyclic AMP-responsive element-binding protein 3-like protein 3 | Transcription factor that may act during endoplasmic reticulum stress by activating unfolded protein response target genes. Activated in response to cAMP stimulation. In vitro, binds to the cAMP response element (CRE) and box-B element. Activates transcription through box-B element. Activates transcription through CRE. Seems to function synergistically with ATF6. In acute inflammatory response, may activate expression of acute phase response (APR) genes. May be involved in growth suppression. | loss of proteostasis | Longevity regulating pathway |
| CREB3L4 | cyclic AMP-responsive element-binding protein 3-like protein 4 | Transcriptional activator that may play a role in the unfolded protein response. Binds to the UPR element (UPRE) but not to CRE element. Preferentially binds DNA with to the consensus sequence 5'-T[GT]ACGT[GA][GT]-3' and has transcriptional activation activity from UPRE. Binds to NF-kappa-B site and has transcriptional activation activity from NF-kappa-B-containing regulatory elements. | loss of proteostasis | Longevity regulating pathway |
| ADCY4 | Adenylate cyclase type 4 | Catalyzes the formation of the signaling molecule cAMP in response to G-protein signaling. | altered intercellular communication | Longevity regulating pathway |
| EIF4E1B | eukaryotic translation initiation factor 4E type 1B | Recognizes and binds the 7-methylguanosine-containing mRNA cap during an early step in the initiation of protein synthesis and facilitates ribosome binding by inducing the unwinding of the mRNAs secondary structure. | others | Longevity regulating pathway |
| AKT1S1 | AKT1 substrate 1 | Subunit of mTORC1, which regulates cell growth and survival in response to nutrient and hormonal signals. Activated mTORC1 up-regulates protein synthesis by phosphorylating key regulators of mRNA translation and ribosome synthesis. mTORC1 phosphorylates EIF4EBP1 and releases it from inhibiting the elongation initiation factor 4E (eiF4E). mTORC1 phosphorylates and activates S6K1 at 'Thr-389', which then promotes protein synthesis by phosphorylating PDCD4 and targeting it for degradation. May also play a role in nerve growth factor-mediated neuroprotection. | deregulated nutrient sensing | Longevity regulating pathway |
| IRS4 | insulin receptor substrate 4 | It acts as an interface between multiple growth factor receptors possessing tyrosine kinase activity, such as insulin receptor, IGF1R and FGFR1, and a complex network of intracellular signaling molecules containing SH2 domains. It is involved in the IGF1R mitogenic signaling pathway. It promotes the AKT1 signaling pathway and BAD phosphorylation during insulin stimulation without activation of RPS6KB1 or the inhibition of apoptosis. | altered intercellular communication | Longevity regulating pathway |
| SESN2 | Sestrin-2 | SESN2 functions as an intracellular leucine sensor that negatively regulates the TORC1 signaling pathway through the GATOR complex. In absence of leucine, SESN2 binds the GATOR subcomplex GATOR2 and prevents TORC1 signaling. This stress-inducible metabolic regulator also plays a role in protection against oxidative and genotoxic stresses. SESN2 may negatively regulate protein translation in response to endoplasmic reticulum stress, via TORC1. | others | Longevity regulating pathway |
| PIK3CA | phosphatidylinositol-4,5-bisphosphate 3-kinase, catalytic subunit alpha | Phosphoinositide-3-kinase (PI3K) that phosphorylates PtdIns (Phosphatidylinositol), PtdIns4P (Phosphatidylinositol 4-phosphate) and PtdIns (4,5) P2 (Phosphatidylinositol 4,5-bisphosphate) to generate phosphatidylinositol 3,4,5-trisphosphate (PIP3). PIP3 plays a key role by recruiting PH domain-containing proteins to the membrane, including AKT1 and PDPK1, activating signaling cascades involved in cell growth, survival, proliferation, motility and morphology. It participates in cellular signaling in response to various growth factors. It is involved in the activation of AKT1 upon stimulation by receptor tyrosine kinases ligands such as EGF, insulin, IGF1, VEGFA and PDGF. | altered intercellular communication | Longevity regulating pathway |
| PIK3CB | phosphatidylinositol-4,5-bisphosphate 3-kinase, catalytic subunit beta | PIK3CB is a phosphoinositide-3-kinase (PI3K) that phosphorylates PtdIns (Phosphatidylinositol), PtdIns4P (Phosphatidylinositol 4-phosphate) and PtdIns (4,5) P2 (Phosphatidylinositol 4,5-bisphosphate) to generate phosphatidylinositol 3,4,5-trisphosphate (PIP3). PIP3 plays a key role by recruiting PH domain-containing proteins to the membrane, including AKT1 and PDPK1, activating signaling cascades involved in cell growth, survival, proliferation, motility and morphology. | cellular senescence | Longevity regulating pathway |
| PIK3CD | Phosphatidylinositol 4,5-bisphosphate 3-kinase catalytic subunit delta isoform | Phosphoinositide-3-kinase (PI3K) that phosphorylates PtdIns(4,5)P2 (Phosphatidylinositol 4,5-bisphosphate) to generate phosphatidylinositol 3,4,5-trisphosphate (PIP3). PIP3 plays a key role by recruiting PH domain-containing proteins to the membrane, including AKT1 and PDPK1, activating signaling cascades involved in cell growth, survival, proliferation, motility and morphology. Mediates immune responses. It plays a role in B-cell development, proliferation, migration, and function. It is required for B-cell receptor (BCR) signaling. | altered intercellular communication | Longevity regulating pathway |
| PIK3R1 | phosphoinositide-3-kinase, regulatory subunit 1 (alpha) | Together with PIK3CB, PIK3R1 is involved in insulin (INS) signaling and energy metabolism. | mitochondrial dysfunction | Longevity regulating pathway |
| PIK3R2 | Phosphatidylinositol 3-kinase regulatory subunit beta | Regulatory subunit of phosphoinositide-3-kinase (PI3K), a kinase that phosphorylates PtdIns (4,5) P2 (Phosphatidylinositol 4,5-bisphosphate) to generate phosphatidylinositol 3,4,5-trisphosphate (PIP3). PIP3 plays a key role by recruiting PH domain-containing proteins to the membrane, including AKT1 and PDPK1, activating signaling cascades involved in cell growth, survival, proliferation, motility and morphology. Binds to activated (phosphorylated) protein-tyrosine kinases, through its SH2 domain, and acts as an adapter, mediating the association of the p110 catalytic unit to the plasma membrane. Indirectly regulates autophagy. Promotes nuclear translocation of XBP1 isoform 2 in a ER stress- and/or insulin-dependent manner during metabolic overloading in the liver and hence plays a role in glucose tolerance improvement. | altered intercellular communication | Longevity regulating pathway |
| PPARG | peroxisome proliferator-activated receptor gamma | Once activated by a ligand, the nuclear receptor binds to DNA specific PPAR response elements (PPRE) and modulates the transcription of its target genes, such as acyl-CoA oxidase. It therefore controls the peroxisomal beta-oxidation pathway of fatty acids. It is a key regulator of adipocyte differentiation and glucose homeostasis. | deregulated nutrient sensing | Longevity regulating pathway |
| PRKAA1 | 5'-AMP-activated protein kinase catalytic subunit alpha-1 | Catalytic subunit of AMP-activated protein kinase (AMPK), an energy sensor protein kinase that plays a key role in regulating cellular energy metabolism. In response to reduction of intracellular ATP levels, AMPK activates energy-producing pathways and inhibits energy-consuming processes: inhibits protein, carbohydrate and lipid biosynthesis, as well as cell growth and proliferation. AMPK acts via direct phosphorylation of metabolic enzymes, and by longer-term effects via phosphorylation of transcription regulators. | deregulated nutrient sensing | Longevity regulating pathway |
| PRKAA2 | 5'-AMP-activated protein kinase catalytic subunit alpha-2 | Catalytic subunit of AMP-activated protein kinase (AMPK), an energy sensor protein kinase that plays a key role in regulating cellular energy metabolism. In response to reduction of intracellular ATP levels, AMPK activates energy-producing pathways and inhibits energy-consuming processes: inhibits protein, carbohydrate and lipid biosynthesis, as well as cell growth and proliferation. AMPK acts via direct phosphorylation of metabolic enzymes, and by longer-term effects via phosphorylation of transcription regulators. | deregulated nutrient sensing | Longevity regulating pathway |
| PRKAB1 | 5'-AMP-activated protein kinase subunit beta-1 | Non-catalytic subunit of AMP-activated protein kinase (AMPK), an energy sensor protein kinase that plays a key role in regulating cellular energy metabolism. In response to reduction of intracellular ATP levels, AMPK activates energy-producing pathways and inhibits energy-consuming processes: inhibits protein, carbohydrate and lipid biosynthesis, as well as cell growth and proliferation. AMPK acts via direct phosphorylation of metabolic enzymes, and by longer-term effects via phosphorylation of transcription regulators. | others | Longevity regulating pathway |
| PRKAB2 | 5'-AMP-activated protein kinase subunit beta-2 | Non-catalytic subunit of AMP-activated protein kinase (AMPK), an energy sensor protein kinase that plays a key role in regulating cellular energy metabolism. In response to reduction of intracellular ATP levels, AMPK activates energy-producing pathways and inhibits energy-consuming processes: inhibits protein, carbohydrate and lipid biosynthesis, as well as cell growth and proliferation. AMPK acts via direct phosphorylation of metabolic enzymes, and by longer-term effects via phosphorylation of transcription regulators. | others | Longevity regulating pathway |
| PRKACA | cAMP-dependent protein kinase catalytic subunit alpha | PRKACA phosphorylates a large number of substrates in the cytoplasm and the nucleus. It regulates the abundance of compartmentalized pools of its regulatory subunits through phosphorylation of PJA2 which binds and ubiquitinates these subunits, leading to their subsequent proteolysis. It is involved in the regulation of platelets in response to thrombin and collagen; maintains circulating platelets in a resting state by phosphorylating proteins in numerous platelet inhibitory pathways when in complex with NF-kappa-B (NFKB1 and NFKB2) and I-kappa-B-alpha (NFKBIA), but thrombin and collagen disrupt these complexes and free active PRKACA stimulates platelets and leads to platelet aggregation by phosphorylating VASP. | others | Longevity regulating pathway |
| PRKACB | cAMP-dependent protein kinase catalytic subunit beta | PRKACB mediates cAMP-dependent signaling triggered by receptor binding to GPCRs. PKA activation regulates diverse cellular processes such as cell proliferation, the cell cycle, differentiation and regulation of microtubule dynamics, chromatin condensation and decondensation, nuclear envelope disassembly and reassembly, as well as regulation of intracellular transport mechanisms and ion flux. It regulates the abundance of compartmentalized pools of its regulatory subunits through phosphorylation of PJA2 which binds and ubiquitinates these subunits, leading to their subsequent proteolysis. | altered intercellular communication | Longevity regulating pathway |
| PRKACG | cAMP-dependent protein kinase catalytic subunit gamma | PRKACG phosphorylates a large number of substrates in the cytoplasm and the nucleus. | others | Longevity regulating pathway |
| PRKAG1 | 5'-AMP-activated protein kinase subunit gamma-1 | AMP/ATP-binding subunit of AMP-activated protein kinase (AMPK), an energy sensor protein kinase that plays a key role in regulating cellular energy metabolism. In response to reduction of intracellular ATP levels, AMPK activates energy-producing pathways and inhibits energy-consuming processes: inhibits protein, carbohydrate and lipid biosynthesis, as well as cell growth and proliferation. AMPK acts via direct phosphorylation of metabolic enzymes, and by longer-term effects via phosphorylation of transcription regulators. | others | Longevity regulating pathway |
| RELA | v-rel avian reticuloendotheliosis viral oncogene homolog A | NF-kappa B, which consists of two polypeptides, p50 (M(r) 50K) and p65/RelA (M(r) 65K), is thought to be a key regulator of genes involved in responses to infection, inflammation and stress. RelA controls inducible, but not basal, transcription in NF-kappa B-regulated pathways. | altered intercellular communication | Longevity regulating pathway |
| RHEB | GTP-binding protein Rheb | RHEB activates the protein kinase activity of mTORC1, and thereby plays a role in the regulation of apoptosis. RHEB stimulates the phosphorylation of S6K1 and EIF4EBP1 through activation of mTORC1 signaling. RHEB has low intrinsic GTPase activity. | others | Longevity regulating pathway |
| RPS6KB1 | ribosomal protein S6 kinase beta-1 | Serine/threonine-protein kinase that acts downstream of mTOR signaling in response to growth factors and nutrients to promote cell proliferation, cell growth and cell cycle progression. It regulates protein synthesis through phosphorylation of EIF4B, RPS6 and EEF2K, and contributes to cell survival by repressing the pro-apoptotic function of BAD. It also plays a role in feedback regulation of mTORC2 by mTORC1 by phosphorylating RICTOR, resulting in the inhibition of mTORC2 and AKT1 signaling. It mediates cell survival by phosphorylating the pro-apoptotic protein BAD and suppressing its pro-apoptotic function. | others | Longevity regulating pathway |
| RPS6KB2 | ribosomal protein S6 kinase beta-2 | It phosphorylates specifically ribosomal protein S6. It seems to act downstream of mTOR signaling in response to growth factors and nutrients to promote cell proliferation, cell growth and cell cycle progression in an alternative pathway regulated by MEAK7. | others | Longevity regulating pathway |
| SOD2 | superoxide dismutase 2, mitochondrial | Overexpression of SOD2 in aging mouse hearts demonstrated a protective role against oxidative stress, fibrosis and apoptosis. | others | Longevity regulating pathway |
| STK11 | serine/threonine kinase 11 | STK11 appears to be involved in TP53-related apoptosis and regulates specific p53-dependent apoptosis pathways. | cellular senescence | Longevity regulating pathway |
| TP53 | tumor protein p53 | TP53 acts as a tumor suppressor in many tumor types; induces growth arrest or apoptosis depending on the physiological circumstances and cell type. TP53 is involved in cell cycle regulation as a trans-activator that acts to negatively regulate cell division by controlling a set of genes required for this process. | others | Longevity regulating pathway |
| TSC1 | Hamartin | In complex with TSC2, TSC1 inhibits the nutrient-mediated or growth factor-stimulated phosphorylation of S6K1 and EIF4EBP1 by negatively regulating mTORC1 signaling. TSC1 is involved in microtubule-mediated protein transport, but this seems to be due to unregulated mTOR signaling. TSC1 acts as a co-chaperone for HSP90AA1 facilitating HSP90AA1 chaperoning of protein clients such as kinases, TSC2 and glucocorticoid receptor NR3C1. | deregulated nutrient sensing | Longevity regulating pathway |
| TSC2 | Tuberin | In complex with TSC1, this tumor suppressor inhibits the nutrient-mediated or growth factor-stimulated phosphorylation of S6K1 and EIF4EBP1 by negatively regulating mTORC1 signaling. TSC2 acts as a GTPase-activating protein (GAP) for the small GTPase RHEB, a direct activator of the protein kinase activity of mTORC1. TSC2 may also play a role in microtubule-mediated protein transport. | deregulated nutrient sensing | Longevity regulating pathway |
| ULK1 | Serine/threonine-protein kinase ULK1 | ULK1 acts upstream of phosphatidylinositol 3-kinase PIK3C3 to regulate the formation of autophagophores, the precursors of autophagosomes. ULK1 is part of regulatory feedback loops in autophagy: acts both as a downstream effector and negative regulator of mammalian target of rapamycin complex 1 (mTORC1) via interaction with RPTOR. ULK1 is activated via phosphorylation by AMPK and also acts as a regulator of AMPK by mediating phosphorylation of AMPK subunits PRKAA1, PRKAB2 and PRKAG1, leading to negatively regulate AMPK activity. | others | Longevity regulating pathway |
| KRAS | GTPase Kras | Ras proteins bind GDP/GTP and possess intrinsic GTPase activity. It plays an important role in the regulation of cell proliferation. It plays a role in promoting oncogenic events by inducing transcriptional silencing of tumor suppressor genes (TSGs) in colorectal cancer (CRC) cells in a ZNF304-dependent manner. | others | Longevity regulating pathway |
| PIK3R3 | Phosphatidylinositol 3-kinase regulatory subunit gamma | PIK3R3 binds to activated (phosphorylated) protein-tyrosine kinases through its SH2 domain and regulates their kinase activity. During insulin stimulation, it also binds to IRS-1. | altered intercellular communication | Longevity regulating pathway |
| IRS2 | insulin receptor substrate 2 | May mediate the control of various cellular processes by insulin. | cellular senescence | Longevity regulating pathway |
| KL | klotho | KL may have weak glycosidase activity towards glucuronylated steroids. | others | Longevity regulating pathway |
| ADIPOQ | Adiponectin receptor protein 1 | Receptor for ADIPOQ, an essential hormone secreted by adipocytes that regulates glucose and lipid metabolism. Required for normal glucose and fat homeostasis and for maintaining a normal body weight. ADIPOQ-binding activates a signaling cascade that leads to increased AMPK activity, and ultimately to increased fatty acid oxidation, increased glucose uptake and decreased gluconeogenesis. | deregulated nutrient sensing | Longevity regulating pathway |
| EIF4E2 | eukaryotic translation initiation factor 4E type 2 | Recognizes and binds the 7-methylguanosine-containing mRNA cap during an early step in the initiation. Acts as a repressor of translation initiation. In contrast to EIF4E, it is unable to bind eIF4G (EIF4G1, EIF4G2 or EIF4G3), suggesting that it acts by competing with EIF4E and block assembly of eIF4F at the cap. | others | Longevity regulating pathway |
| ATG5 | Autophagy protein 5 | Involved in autophagic vesicle formation. Conjugation with ATG12, through a ubiquitin-like conjugating system involving ATG7 as an E1-like activating enzyme and ATG10 as an E2-like conjugating enzyme, is essential for its function. The ATG12-ATG5 conjugate acts as an E3-like enzyme which is required for lipidation of ATG8 family proteins and their association to the vesicle membranes. Involved in mitochondrial quality control after oxidative damage, and in subsequent cellular longevity. Plays a critical role in multiple aspects of lymphocyte development and is essential for both B and T lymphocyte survival and proliferation. Required for optimal processing and presentation of antigens for MHC II. Involved in the maintenance of axon morphology and membrane structures, as well as in normal adipocyte differentiation. Promotes primary ciliogenesis through removal of OFD1 from centriolar satellites and degradation of IFT20 via the autophagic pathway. | others | Longevity regulating pathway |
| CREB5 | cyclic AMP-responsive element-binding protein 5 | Binds to the cAMP response element and activates transcription. | others | Longevity regulating pathway |
| ATG13 | Autophagy-related protein 13 | Autophagy factor required for autophagosome formation and mitophagy. Target of the TOR kinase signaling pathway that regulates autophagy through the control of the phosphorylation status of ATG13 and ULK1, and the regulation of the ATG13-ULK1-RB1CC1 complex. Through its regulation of ULK1 activity, plays a role in the regulation of the kinase activity of mTORC1 and cell proliferation. | others | Longevity regulating pathway |
| RB1CC1 | RB1-inducible coiled-coil protein 1 | RB1CC1 is involved in autophagy. RB1CC1 regulates early events but also late events of autophagosome formation through direct interaction with Atg16L1. RB1CC1 is involved in repair of DNA damage caused by ionizing radiation, which subsequently improves cell survival by decreasing apoptosis. | others | Longevity regulating pathway |
| AKT3 | RAC-gamma serine/threonine-protein kinase | AKT3 is one of 3 closely related serine/threonine-protein kinases (AKT1, AKT2 and AKT3) called the AKT kinase, and which regulate many processes including metabolism, proliferation, cell survival, growth and angiogenesis. This is mediated through serine and/or threonine phosphorylation of a range of downstream substrates. AKT3 is the least studied AKT isoform. It plays an important role in brain development and is crucial for the viability of malignant glioma cells. AKT3 isoform may also be the key molecule in up-regulation and down-regulation of MMP13 via IL13. Required for the coordination of mitochondrial biogenesis with growth factor-induced increases in cellular energy demands. Down-regulation by RNA interference reduces the expression of the phosphorylated form of BAD, resulting in the induction of caspase-dependent apoptosis. | mitochondrial dysfunction | Longevity regulating pathway |
| CREB3 | cyclic AMP-responsive element-binding protein 3 | Endoplasmic reticulum (ER)-bound sequence-specific transcription factor that directly binds DNA and activates transcription. Plays a role in the unfolded protein response (UPR), promoting cell survival versus ER stress-induced apoptotic cell death. Also involved in cell proliferation, migration and differentiation, tumor suppression and inflammatory gene expression. Acts as a positive regulator of LKN-1/CCL15-induced chemotaxis signaling of leukocyte cell migration. Associates with chromatin to the HERPUD1 promoter. Also induces transcriptional activation of chemokine receptors. | loss of proteostasis | Longevity regulating pathway |
| CAMKK2 | Calcium/calmodulin-dependent protein kinase kinase 2 | Calcium/calmodulin-dependent protein kinase belonging to a proposed calcium-triggered signaling cascade involved in a number of cellular processes. Isoform 1, isoform 2 and isoform 3 phosphorylate CAMK1 and CAMK4. Isoform 3 phosphorylates CAMK1D. Isoform 4, isoform 5 and isoform 6 lacking part of the calmodulin-binding domain are inactive. Efficiently phosphorylates 5'-AMP-activated protein kinase (AMPK) trimer, including that consisting of PRKAA1, PRKAB1 and PRKAG1. This phosphorylation is stimulated in response to Ca2+ signals. Seems to be involved in hippocampal activation of CREB1. May play a role in neurite growth. Isoform 3 may promote neurite elongation, while isoform 1 may promoter neurite branching. | others | Longevity regulating pathway |
| PPARGC1A | peroxisome proliferator-activated receptor gamma, coactivator 1 alpha | Transcriptional coactivator for steroid receptors and nuclear receptors. It greatly increases the transcriptional activity of PPARG and thyroid hormone receptor on the uncoupling protein promoter. It can regulate key mitochondrial genes that contribute to the program of adaptive thermogenesis. It plays an essential role in metabolic reprogramming in response to dietary availability through coordination of the expression of a wide array of genes involved in glucose and fatty acid metabolism. | altered intercellular communication | Longevity regulating pathway |
| EHMT2 | euchromatic histone lysine methyltransferase 2 | Histone methyltransferase that specifically mono- and dimethylates 'Lys-9' of histone H3 (H3K9me1 and H3K9me2, respectively) in euchromatin. H3K9me represents a specific tag for epigenetic transcriptional repression by recruiting HP1 proteins to methylated histones. Also mediates monomethylation of 'Lys-56' of histone H3 (H3K56me1) in G1 phase, leading to promote interaction between histone H3 and PCNA and regulating DNA replication. Also weakly methylates 'Lys-27' of histone H3 (H3K27me). Also required for DNA methylation, the histone methyltransferase activity is not required for DNA methylation, suggesting that these 2 activities function independently. Probably targeted to histone H3 by different DNA-binding proteins like E2F6, MGA, MAX and/or DP1. May also methylate histone H1. In addition to the histone methyltransferase activity, also methylates non-histone proteins: mediates dimethylation of 'Lys-373' of p53/TP53. Also methylates CDYL, WIZ, ACIN1, DNMT1, HDAC1, ERCC6, KLF12 and itself. | epigenetic alterations | Longevity regulating pathway |
| SIRT1 | sirtuin 1 | SIRT1 is a NAD-dependent deacetylase, which can regulate a number of processes by deacetylating key proteins, such as TP53. | epigenetic alterations | Longevity regulating pathway |
| APPL1 | DCC-interacting protein 13-alpha | Multifunctional adapter protein that binds to various membrane receptors, nuclear factors and signaling proteins to regulate many processes, such as cell proliferation, immune response, endosomal trafficking and cell metabolism. Regulates signaling pathway leading to cell proliferation through interaction with RAB5A and subunits of the NuRD/MeCP1 complex. Functions as a positive regulator of innate immune response via activation of AKT1 signaling pathway by forming a complex with APPL1 and PIK3R1. Involved in trafficking of the TGFBR1 from the endosomes to the nucleus via microtubules in a TRAF6-dependent manner. Plays a role in cell metabolism by regulating adiponecting and insulin signaling pathways. Required for fibroblast migration through HGF cell signaling. | altered intercellular communication | Longevity regulating pathway |
| SESN1 | Sestrin-1 | SESN1 functions as an intracellular leucine sensor that negatively regulates the TORC1 signaling pathway through the GATOR complex. In absence of leucine, SESN1 binds the GATOR subcomplex GATOR2 and prevents TORC1 signaling. Binding of leucine to SESN2 disrupts its interaction with GATOR2 thereby activating the TORC1 signaling pathway. This stress-inducible metabolic regulator may also play a role in protection against oxidative and genotoxic stresses. | others | Longevity regulating pathway |
| ADIPOR1 | Adiponectin receptor protein 1 | Receptor for ADIPOQ, an essential hormone secreted by adipocytes that regulates glucose and lipid metabolism. Required for normal glucose and fat homeostasis and for maintaining a normal body weight. ADIPOQ-binding activates a signaling cascade that leads to increased AMPK activity, and ultimately to increased fatty acid oxidation, increased glucose uptake and decreased gluconeogenesis. | deregulated nutrient sensing | Longevity regulating pathway |
| PRKAG2 | 5'-AMP-activated protein kinase subunit gamma-2 | AMP/ATP-binding subunit of AMP-activated protein kinase (AMPK), an energy sensor protein kinase that plays a key role in regulating cellular energy metabolism. In response to reduction of intracellular ATP levels, AMPK activates energy-producing pathways and inhibits energy-consuming processes: inhibits protein, carbohydrate and lipid biosynthesis, as well as cell growth and proliferation. AMPK acts via direct phosphorylation of metabolic enzymes, and by longer-term effects via phosphorylation of transcription regulators. | others | Longevity regulating pathway |
| PRKAG3 | 5'-AMP-activated protein kinase subunit gamma-3 | AMP/ATP-binding subunit of AMP-activated protein kinase (AMPK), an energy sensor protein kinase that plays a key role in regulating cellular energy metabolism. In response to reduction of intracellular ATP levels, AMPK activates energy-producing pathways and inhibits energy-consuming processes: inhibits protein, carbohydrate and lipid biosynthesis, as well as cell growth and proliferation. AMPK acts via direct phosphorylation of metabolic enzymes, and by longer-term effects via phosphorylation of transcription regulators. | others | Longevity regulating pathway |
| RPTOR | regulatory-associated protein of mTOR | It involved in the control of the mammalian target of rapamycin complex 1 (mTORC1) activity which regulates cell growth and survival, and autophagy in response to nutrient and hormonal signals; functions as a scaffold for recruiting mTORC1 substrates. mTORC1 is activated in response to growth factors or amino acids. Growth factor-stimulated mTORC1 activation involves a AKT1-mediated phosphorylation of TSC1-TSC2, which leads to the activation of the RHEB GTPase that potently activates the protein kinase activity of mTORC1. | deregulated nutrient sensing | Longevity regulating pathway |
| ATG101 | Autophagy-related protein 101 | Autophagy factor required for autophagosome formation. Stabilizes ATG13, protecting it from proteasomal degradation. | others | Longevity regulating pathway |
| CREB3L2 | cyclic AMP-responsive element-binding protein 3-like protein 2 | Transcription factor involved in unfolded protein response (UPR). In the absence of endoplasmic reticulum (ER) stress, inserted into ER membranes, with N-terminal DNA-binding and transcription activation domains oriented toward the cytosolic face of the membrane. In response to ER stress, transported to the Golgi, where it is cleaved in a site-specific manner by resident proteases S1P/MBTPS1 and S2P/MBTPS2. The released N-terminal cytosolic domain is translocated to the nucleus to effect transcription of specific target genes. Plays a critical role in chondrogenesis by activating the transcription of SEC23A, which promotes the transport and secretion of cartilage matrix proteins, and possibly that of ER biogenesis-related genes. In a neuroblastoma cell line, protects cells from ER stress-induced death. In vitro activates transcription of target genes via direct binding to the CRE site. | loss of proteostasis | Longevity regulating pathway |
| ADIPOR2 | Adiponectin receptor protein 2 | Receptor for ADIPOQ, an essential hormone secreted by adipocytes that regulates glucose and lipid metabolism. Required for normal body fat and glucose homeostasis. ADIPOQ-binding activates a signaling cascade that leads to increased PPARA activity, and ultimately to increased fatty acid oxidation and glucose uptake. Has intermediate affinity for globular and full-length adiponectin. Required for normal revascularization after chronic ischemia caused by severing of blood vessels. | deregulated nutrient sensing | Longevity regulating pathway |
| PTPN11 | protein tyrosine phosphatase, non-receptor type 11 | PTPN11 acts downstream of various receptor and cytoplasmic protein tyrosine kinases to participate in the signal transduction from the cell surface to the nucleus. | others | JAK-STAT signaling pathway |
| CTF1 | cardiotrophin 1 | Induce cardiac myocyte hypertrophy in vitro. Binds to and activates the ILST/gp130 receptor. | others | JAK-STAT signaling pathway |
| EPOR | erythropoietin receptor | Receptor for erythropoietin. Mediates erythropoietin-induced erythroblast proliferation and differentiation. Upon EPO stimulation, EPOR dimerizes triggering the JAK2/STAT5 signaling cascade. | cellular senescence | JAK-STAT signaling pathway |
| IFNB1 | interferon beta | It has antiviral, antibacterial and anticancer activities. | others | JAK-STAT signaling pathway |
| IL2 | interleukin 2 | Produced by T-cells in response to antigenic or mitogenic stimulation, this protein is required for T-cell proliferation and other activities crucial to regulation of the immune response. | cellular senescence | JAK-STAT signaling pathway |
| IL2RG | interleukin 2 receptor, gamma | Common subunit for the receptors for a variety of interleukins. | others | JAK-STAT signaling pathway |
| IL7 | interleukin 7 | It stimulates the proliferation of lymphoid progenitors, inhibit aging. | cellular senescence | JAK-STAT signaling pathway |
| IL7R | interleukin 7 receptor | Receptor for interleukin-7. Also acts as a receptor for thymic stromal lymphopoietin (TSLP). | cellular senescence | JAK-STAT signaling pathway |
| LEP | leptin | LEP is secreted from adipose tissues and is involved in food intake and energy expenditure. | deregulated nutrient sensing | JAK-STAT signaling pathway |
| LEPR | leptin receptor | Deletion of LEPR in neurons of mice results in obesity. | deregulated nutrient sensing | JAK-STAT signaling pathway |
| FBP1 | fructose-bisphosphatase 1 | Catalyzes the hydrolysis of fructose 1,6-bisphosphate to fructose 6-phosphate in the presence of divalent cations, acting as a rate-limiting enzyme in gluconeogenesis. Plays a role in regulating glucose sensing and insulin secretion of pancreatic beta-cells. | others | Insulin signaling pathway |
| GCK | glucokinase | Catalyzes the phosphorylation of hexose, such as D-glucose, D-fructose and D-mannose, to hexose 6-phosphate. | others | Insulin signaling pathway |
| GRB2 | growth factor receptor-bound protein 2 | GRB2 has a role as a signal transducer of many pathways, including pathways and genes previously related to ageing such as INS/IGF1 signaling and SHC1. | deregulated nutrient sensing | Insulin signaling pathway |
| GSK3B | glycogen synthase kinase 3 beta | Adult overexpression of GSK3B in the brain of resulted in neurodegeneration. GSK-3beta has been postulated to mediate Alzheimer's disease tau hyperphosphorylation, beta-amyloid-induced neurotoxicity and presenilin-1 mutation pathogenic effects. | others | Insulin signaling pathway |
| IKBKB | inhibitor of kappa light polypeptide gene enhancer in B-cells, kinase beta | Serine kinase that plays an essential role in the NF-kappa-B signaling pathway which is activated by multiple stimuli such as inflammatory cytokines, bacterial or viral products, DNA damages or other cellular stresses. Acts as part of the canonical IKK complex in the conventional pathway of NF-kappa-B activation. Phosphorylates inhibitors of NF-kappa-B on 2 critical serine residues. | altered intercellular communication | Insulin signaling pathway |
| PCK1 | phosphoenolpyruvate carboxykinase 1 (soluble) | PCK1 regulates cataplerosis and anaplerosis, the processes that control the levels of metabolic intermediates in the citric acid cycle. At low glucose levels, it catalyzes the cataplerotic conversion of oxaloacetate (OAA) to phosphoenolpyruvate (PEP), the rate-limiting step in the metabolic pathway that produces glucose from lactate and other precursors derived from the citric acid cycle. At high glucose levels, it catalyzes the anaplerotic conversion of phosphoenolpyruvate to oxaloacetate. | others | Insulin signaling pathway |
| PDPK1 | 3-phosphoinositide dependent protein kinase 1 | PDK1 functions as a master kinase, phosphorylating and activating PKB/Akt, S6K and RSK. PDK1 is essential for embryonic development, and regulates cell size independently of cell number or proliferation, as well as insulin's ability to activate PKB, S6K and RSK. | others | Insulin signaling pathway |
| PPP1CA | protein phosphatase 1, catalytic subunit, alpha isozyme | It is involved in different cellular processes including cell division, protein synthesis, and synaptic plasticity. | loss of proteostasis | Insulin signaling pathway |
| MAPK3 | mitogen-activated protein kinase 3 | It is involved in stress response signaling and maybe in cell cycle control. | stem cell exhaustion | Insulin signaling pathway |
| MAPK8 | mitogen-activated protein kinase 8 | MAPK8, also known as JNK1, encodes many transcripts and is an important player in stress response. Overexpression of JNK in roundworms also increases lifespan. | deregulated nutrient sensing | Insulin signaling pathway |
| MAPK9 | mitogen-activated protein kinase 9 | Also called JNK2, MAPK9 encodes many transcripts and is an important player in stress response. Mice without MAPK8 and MAPK9 die at embryonic stages. | stem cell exhaustion | Insulin signaling pathway |
| PTPN1 | protein tyrosine phosphatase, non-receptor type 1 | PTPN1 is a tyrosine-protein phosphatase which acts as a regulator of endoplasmic reticulum unfolded protein response. | loss of proteostasis | Insulin signaling pathway |
| SHC1 | SHC (Src homology 2 domain containing) transforming protein 1 | Signaling adapter that couples activated growth factor receptors to signaling pathways. SHC1 participates in a signaling cascade initiated by activated KIT and KITLG/SCF. | others | Insulin signaling pathway |
| SOCS2 | suppressor of cytokine signaling 2 | SOCS family proteins form part of a classical negative feedback system that regulates cytokine signal transduction. SOCS2 appears to be a negative regulator in the growth hormone/IGF1 signaling pathway. Probable substrate recognition component of a SCF-like ECS (Elongin BC-CUL2/5-SOCS-box protein) E3 ubiquitin-protein ligase complex which mediates the ubiquitination and subsequent proteasomal degradation of target proteins. | altered intercellular communication | Insulin signaling pathway |
| CEBPB | CCAAT/enhancer binding protein (C/EBP), beta | Like CEBPA, CEBPB is a transcription factor involved in fat metabolism. CEBPB also appears to play a role in liver regeneration. | others | IL-17 signaling pathway |
| MMP1 | matrix metallopeptidase 1 | Its expression level in skin fibroblasts was altered in the process of aging, and the cell growth rate was reduced. | senescence-associated secretory phenotype | IL-17 signaling pathway |
| MMP3 | Matrix Metallopeptidase 3 | Can degrade fibronectin, laminin, gelatins of type I, III, IV, and V; collagens III, IV, X, and IX, and cartilage proteoglycans. Activates procollagenase. | senescence-associated secretory phenotype | IL-17 signaling pathway |
| MMP13 | Matrix Metallopeptidase 13 | Plays a role in the degradation of extracellular matrix proteins including fibrillar collagen, fibronectin, TNC and ACAN. Cleaves triple helical collagens, including type I, type II and type III collagen, but has the highest activity with soluble type II collagen. Can also degrade collagen type IV, type XIV and type X. May also function by activating or degrading key regulatory proteins, such as TGFB1 and CCN2. Plays a role in wound healing, tissue remodeling, cartilage degradation, bone development, bone mineralization and ossification. Required for normal embryonic bone development and ossification. Plays a role in the healing of bone fractures via endochondral ossification. Plays a role in wound healing, probably by a mechanism that involves proteolytic activation of TGFB1 and degradation of CCN2. Plays a role in keratinocyte migration during wound healing. May play a role in cell migration and in tumor cell invasion. | senescence-associated secretory phenotype | IL-17 signaling pathway |
| CREBBP | CREB binding protein | Acetylates histones, giving a specific tag for transcriptional activation. Also acetylates non-histone proteins, like DDX21, FBL, IRF2, MAFG, NCOA3, POLR1E/PAF53 and FOXO1. Binds specifically to phosphorylated CREB and enhances its transcriptional activity toward cAMP-responsive genes. Acts as a coactivator of ALX1. Acts as a circadian transcriptional coactivator which enhances the activity of the circadian transcriptional activators: NPAS2-ARNTL/BMAL1 and CLOCK-ARNTL/BMAL1 heterodimers. | others | HIF-1 signaling pathway |
| ENO1 | enolase 1 | Glycolytic enzyme the catalyzes the conversion of 2-phosphoglycerate to phosphoenolpyruvate. In addition to glycolysis, involved in various processes such as growth control, hypoxia tolerance and allergic responses. | others | HIF-1 signaling pathway |
| ENO2 | enolase 2 | Has neurotrophic and neuroprotective properties on a broad spectrum of central nervous system (CNS) neurons. | others | HIF-1 signaling pathway |
| GAPDH | glyceraldehyde-3-phosphate dehydrogenase | Has both glyceraldehyde-3-phosphate dehydrogenase and nitrosylase activities, thereby playing a role in glycolysis and nuclear functions, respectively. Participates in nuclear events including transcription, RNA transport, DNA replication and apoptosis. | others | HIF-1 signaling pathway |
| HIF1A | hypoxia inducible factor 1, alpha subunit (basic helix-loop-helix transcription factor) | Functions as a master transcriptional regulator of the adaptive response to hypoxia. | others | HIF-1 signaling pathway |
| HK3 | hexokinase 3 | Catalyzes the phosphorylation of hexose, such as D-glucose and D-fructose, to hexose 6-phosphate (D-glucose 6-phosphate and D-fructose 6-phosphate. | others | HIF-1 signaling pathway |
| TIMP1 | TIMP Metallopeptidase Inhibitor 1 | Metalloproteinase inhibitor that functions by forming one to one complexes with target metalloproteinases, such as collagenases, and irreversibly inactivates them by binding to their catalytic zinc cofactor. Acts on MMP1, MMP2, MMP3, MMP7, MMP8, MMP9, MMP10, MMP11, MMP12, MMP13 and MMP16. Does not act on MMP14. Also functions as a growth factor that regulates cell differentiation, migration and cell death and activates cellular signaling cascades via CD63 and ITGB1. Plays a role in integrin signaling. Mediates erythropoiesis in vitro; but, unlike IL3, it is species-specific, stimulating the growth and differentiation of only human and murine erythroid progenitors. | loss of proteostasis | HIF-1 signaling pathway |
| VEGFA | vascular endothelial growth factor A | Growth factor active in angiogenesis, vasculogenesis and endothelial cell growth. VEGFA induces endothelial cell proliferation, promotes cell migration, inhibits apoptosis and induces permeabilization of blood vessels. VEGFA binds to the FLT1/VEGFR1 and KDR/VEGFR2 receptors, heparan sulfate and heparin. | others | HIF-1 signaling pathway |
| PLCG2 | phospholipase C, gamma 2 (phosphatidylinositol-specific) | The production of the second messenger molecules diacylglycerol (DAG) and inositol 1,4,5-trisphosphate (IP3) is mediated by activated phosphatidylinositol-specific phospholipase C enzymes. It is a crucial enzyme in transmembrane signaling. | others | Growth hormone synthesis, secretion and action |
| POU1F1 | POU class 1 homeobox 1 | POU1F1 is a transcription factor involved in the specification of the lactotrope, somatotrope, and thyrotrope phenotypes in the developing anterior pituitary. | loss of proteostasis | Growth hormone synthesis, secretion and action |
| PTK2 | protein tyrosine kinase 2 | PTK2 is necessary for fitness, egulating cell migration, adhesion, spreading, reorganization of the actin cytoskeleton. | altered intercellular communication | Growth hormone synthesis, secretion and action |
| SST | somatostatin | SST inhibits the secretion of pituitary hormones, including that of growth hormone/somatotropin (GH1), PRL, ACTH, luteinizing hormone (LH) and TSH. | others | Growth hormone synthesis, secretion and action |
| SSTR3 | somatostatin receptor 3 | It has been associated with melanoma. | others | Growth hormone synthesis, secretion and action |
| STAT3 | signal transducer and activator of transcription 3 (acute-phase response factor) | Signal transducer and transcription activator that mediates cellular responses to interleukins, KITLG/SCF, LEP and other growth factors. | cellular senescence | Growth hormone synthesis, secretion and action |
| STAT5A | signal transducer and activator of transcription 5A | STAT5A carries out a dual function: signal transduction and activation of transcription. STAT5A mediates cellular responses to the cytokine KITLG/SCF and other growth factors. | cellular senescence | Growth hormone synthesis, secretion and action |
| STAT5B | signal transducer and activator of transcription 5B | STAT5B carries out a dual function: signal transduction and activation of transcription. STAT5B mediates cellular responses to the cytokine KITLG/SCF and other growth factors. STAT5B binds to the GAS element and activates PRL-induced transcription. STAT5B positively regulates hematopoietic/erythroid differentiation. | cellular senescence | Growth hormone synthesis, secretion and action |
| EP300 | E1A binding protein p300 | EP300 mediates many transcriptional events including DNA repair. EP300 has been associated with several other proteins that may be involved in ageing, such as WRN. | genomic instability | Growth hormone synthesis, secretion and action |
| FOS | FBJ murine osteosarcoma viral oncogene homolog | Nuclear phosphoprotein which forms a tight but non-covalently linked complex with the JUN/AP-1 transcription factor. In the heterodimer, FOS and JUN/AP-1 basic regions each seems to interact with symmetrical DNA half sites. | cellular senescence | Growth hormone synthesis, secretion and action |
| GH1 | growth hormone 1 | It plays an important role in growth control. Its major role in stimulating body growth is to stimulate the liver and other tissues to secrete IGF-1. | cellular senescence | Growth hormone synthesis, secretion and action |
| GHR | growth hormone receptor | Receptor for pituitary gland growth hormone involved in regulating postnatal body growth. On ligand binding, couples to the JAK2/STAT5 pathway. | loss of proteostasis | Growth hormone synthesis, secretion and action |
| GHRH | growth hormone releasing hormone | GRF is released by the hypothalamus and acts on the adenohypophyse to stimulate the secretion of growth hormone. | loss of proteostasis | Growth hormone synthesis, secretion and action |
| GHRHR | growth hormone releasing hormone receptor | Receptor for GRF, coupled to G proteins which activate adenylyl cyclase. Stimulates somatotroph cell growth, growth hormone gene transcription and growth hormone secretion. | deregulated nutrient sensing | Growth hormone synthesis, secretion and action |
| JAK2 | janus kinase 2 | Knockdown or pharmacological inhibition of the JAK-STAT enhances satellite stem cell division potential, resulting in better muscle regeneration. Upon JAK inhibition there was a reduction in inflammatory cytokines and chemokines. | stem cell exhaustion | Growth hormone synthesis, secretion and action |
| EGR1 | early growth response 1 | Transcriptional regulator. Recognizes and binds to the DNA sequence 5'-GCG(T/G) GGGCG-3'(EGR-site) in the promoter region of target genes. | cellular senescence | GnRH signaling pathway |
| PTK2B | protein tyrosine kinase 2 beta | Involved in stress response and signal transduction, PTK2B is an important player in a variety of processes including the regulation of ion channels by calcium and MAPK signaling. | cellular senescence | GnRH signaling pathway |
| PIGF | Phosphatidylinositol Glycan Anchor Biosynthesis Class F | Involved in GPI-anchor biosynthesis through the transfer of ethanolamine phosphate to the third mannose of GPI. | others | Glycosylphosphatidylinositol (GPI)-anchor biosynthesis |
| PSAT1 | Phosphoserine Aminotransferase 1 | Catalyzes the reversible conversion of 3-phosphohydroxypyruvate to phosphoserine and of 3-hydroxy-2-oxo-4-phosphonooxybutanoate to phosphohydroxythreonine. | others | Glycine, serine and threonine metabolism |
| FOXO4 | forkhead box O4 | FOXO4 plays a role in development and in insulin (INS) signaling. | deregulated nutrient sensing | FoxO signaling pathway |
| MMP2 | Matrix Metallopeptidase 2 | Ubiquitinous metalloproteinase that is involved in diverse functions such as remodeling of the vasculature, angiogenesis, tissue repair, tumor invasion, inflammation, and atherosclerotic plaque rupture. As well as degrading extracellular matrix proteins, can also act on several nonmatrix proteins such as big endothelial 1 and beta-type CGRP promoting vasoconstriction. Also cleaves KISS at a Gly-\|-Leu bond. Appears to have a role in myocardial cell death pathways. Contributes to myocardial oxidative stress by regulating the activity of GSK3beta. Cleaves GSK3beta in vitro. Involved in the formation of the fibrovascular tissues in association with MMP14. | senescence-associated secretory phenotype | Endocrine resistance |
| IL2RB | Interleukin 2 Receptor Subunit Beta | Receptor for interleukin-2. This beta subunit is involved in receptor mediated endocytosis and transduces the mitogenic signals of IL2. Probably in association with IL15RA, involved in the stimulation of neutrophil phagocytosis by IL15. | others | Cytokine-cytokine receptor interaction |
| IL6ST | interleukin 6 signal transducer | miR-126a-3p also modulates the expression of IRS1, IRS2, IL6ST and PIK3R2, all targets that enforce the hypothesis that senescent endothelial cells may reduce the proliferative ability and the stemness phenotype of bone marrow-derived mesenchymal stem cells. | senescence-associated secretory phenotype | Cytokine-cytokine receptor interaction |
| IL15 | interleukin 15 | Endurance exercise attenuates age-associated changes to skin in humans and mice and exercise-induced IL-15 is a novel regulator of mitochondrial function in aging skin. | senescence-associated secretory phenotype | Cytokine-cytokine receptor interaction |
| TNFRSF11B | TNF Receptor Superfamily Member 11b | Acts as decoy receptor for TNFSF11/RANKL and thereby neutralizes its function in osteoclastogenesis. Inhibits the activation of osteoclasts and promotes osteoclast apoptosis in vitro. Bone homeostasis seems to depend on the local ratio between TNFSF11 and TNFRSF11B. May also play a role in preventing arterial calcification. May act as decoy receptor for TNFSF10/TRAIL and protect against apoptosis. TNFSF10/TRAIL binding blocks the inhibition of osteoclastogenesis. | others | Cytokine-cytokine receptor interaction |
| TNFRSF1B | TNF Receptor Superfamily Member 1B | Receptor with high affinity for TNFSF2/TNF-alpha and approximately 5-fold lower affinity for homotrimeric TNFSF1/lymphotoxin-alpha. The TRAF1/TRAF2 complex recruits the apoptotic suppressors BIRC2 and BIRC3 to TNFRSF1B/TNFR2. This receptor mediates most of the metabolic effects of TNF-alpha. Isoform 2 blocks TNF-alpha-induced apoptosis, which suggests that it regulates TNF-alpha function by antagonizing its biological activity. | senescence-associated secretory phenotype | Cytokine-cytokine receptor interaction |
| TNFRSF10C | TNF Receptor Superfamily Member 10c | Receptor for the cytotoxic ligand TRAIL. Lacks a cytoplasmic death domain and hence is not capable of inducing apoptosis. May protect cells against TRAIL mediated apoptosis by competing with TRAIL-R1 and R2 for binding to the ligand. | senescence-associated secretory phenotype | Cytokine-cytokine receptor interaction |
| PLAUR | Plasminogen Activator, Urokinase Receptor | Acts as a receptor for urokinase plasminogen activator. Plays a role in localizing and promoting plasmin formation. Mediates the proteolysis-independent signal transduction activation effects of U-PA. It is subject to negative-feedback regulation by U-PA which cleaves it into an inactive form. | others | Complement and coagulation cascades |
| SERPINB2 | Monocyte Arg-Serpin | Inhibits urokinase-type plasminogen activator. The monocyte derived PAI-2 is distinct from the endothelial cell-derived PAI-1. | senescence-associated secretory phenotype | Complement and coagulation cascades |
| RORA | RAR Related Orphan Receptor A | Nuclear receptor that binds DNA as a monomer to ROR response elements (RORE) containing a single core motif half-site 5'-AGGTCA-3' preceded by a short A-T-rich sequence. Key regulator of embryonic development, cellular differentiation, immunity, circadian rhythm as well as lipid, steroid, xenobiotics and glucose metabolism. Considered to have intrinsic transcriptional activity, have some natural ligands like oxysterols that act as agonists (25-hydroxycholesterol) or inverse agonists (7-oxygenated sterols), enhancing or repressing the transcriptional activity, respectively. | others | Circadian rhythm |
| RB1 | retinoblastoma 1 | Rb is essential for normal mouse development. | others | Cellular senescence |
| TGFB1 | transforming growth factor, beta 1 | The variability of the TGF-beta1 gene may affect longevity by playing a role in inflamm-aging. | altered intercellular communication | Cellular senescence |
| SQSTM1 | sequestosome 1 | Autophagy receptor required for selective macroautophagy (aggrephagy). SQSTM1 functions as a bridge between polyubiquitinated cargo and autophagosomes. SQSTM1 interacts directly with both the cargo to become degraded and an autophagy modifier of the MAP1 LC3 family. Along with WDFY3, SQSTM1 is involved in the formation and autophagic degradation of cytoplasmic ubiquitin-containing inclusions (p62 bodies, ALIS/aggresome-like induced structures). | deregulated nutrient sensing | Cellular senescence |
| CCNA2 | cyclin A2 | Involved in cell cycle control， binds and activates CDK2 and promotes transition through G1/S and G2/M. | stem cell exhaustion | Cellular senescence |
| CDKN2B | cyclin-dependent kinase inhibitor 2B (p15, inhibits CDK4) | Interacts strongly with CDK4 and CDK6. Potent inhibitor. Potential effector of TGF-beta induced cell cycle arrest. | others | Cellular senescence |
| MAPK14 | mitogen-activated protein kinase 14 | It plays an important role in the cascades of cellular responses. | stem cell exhaustion | Cellular senescence |
| E2F1 | E2F transcription factor 1 | Transcription activator that binds DNA cooperatively with DP proteins through the E2 recognition site, 5'-TTTC[CG]CGC-3' found in the promoter region of a number of genes whose products are involved in cell cycle regulation or in DNA replication. | genomic instability | Cellular senescence |
| FOXM1 | forkhead box M1 | Increased Foxm1b levels are essential for GH to stimulate hepatocyte proliferation. Increased expression of FOXM1 in old mice restored hepatocyte proliferation to levels found in young animals. | deregulated nutrient sensing | Cellular senescence |
| IL6 | interleukin 6 | Transgenic mice expressing high levels of IL6 have a reduced growth rate and low levels of IGF1. IL-6-mediated decrease in IGF-I production represents a major mechanism by which chronic inflammation affects growth. | deregulated nutrient sensing | Cellular senescence |
| CXCL8 | C-X-C Motif Chemokine Ligand 8 | IL-8 is a chemotactic factor that attracts neutrophils, basophils, and T-cells, but not monocytes. It is also involved in neutrophil activation. It is released from several cell types in response to an inflammatory stimulus. | senescence-associated secretory phenotype | Cellular senescence |
| MYC | v-myc avian myelocytomatosis viral oncogene homolog | Transcription factor that binds DNA in a non-specific manner, yet also specifically recognizes the core sequence 5'-CAC[GA]TG-3'. Activates the transcription of growth-related genes. | cellular senescence | Cellular senescence |
| NBN | nibrin | Component of the MRE11-RAD50-NBN (MRN complex) which plays a critical role in the cellular response to DNA damage and the maintenance of chromosome integrity. | genomic instability | Cellular senescence |
| ABL1 | ABL proto-oncogene 1, non-receptor tyrosine kinase | c-Abl stimulates p73-mediated transactivation and apoptosis. This regulation of p73 by c-Abl in response to DNA damage is also demonstrated by a failure of ionizing-radiation-induced apoptosis after disruption of the c-Abl-p73 interaction. | genomic instability | Cell cycle |
| BUB1B | BUB1 mitotic checkpoint serine/threonine kinase B | BUB1B is a mitotic checkpoint that controls chromosome segregation and maintains genetic stability. Inhibit aging. | genomic instability | Cell cycle |
| CDK7 | cyclin-dependent kinase 7 | Serine/threonine kinase involved in cell cycle control and in RNA polymerase II-mediated RNA transcription. Cyclin-dependent kinases (CDKs) are activated by the binding to a cyclin and mediate the progression through the cell cycle. Each different complex controls a specific transition between 2 subsequent phases in the cell cycle. Required for both activation and complex formation of CDK1/cyclin-B during G2-M transition, and for activation of CDK2/cyclins during G1-S transition (but not complex formation). Its expression and activity are constant throughout the cell cycle. Upon DNA damage, triggers p53/TP53 activation by phosphorylation, but is inactivated in turn by p53/TP53; this feedback loop may lead to an arrest of the cell cycle and of the transcription, helping in cell recovery, or to apoptosis. Required for DNA-bound peptides-mediated transcription and cellular growth inhibition. | others | Cell cycle |
| HDAC1 | histone deacetylase 1 | HDAC1 performs the deacetylation of histones and is an important player in may processes such as development, transcriptional regulation, and cell cycle progression. | epigenetic alterations | Cell cycle |
| HDAC2 | histone deacetylase 2 | HDAC2 performs the deacetylation of histones and is an important player in may processes such as development, transcriptional regulation, and cell cycle progression. | cellular senescence | Cell cycle |
| PCNA | proliferating cell nuclear antigen | PCNA is an important player in DNA replication and, probably, in DNA repair, and it interacts with WRN. | genomic instability | Cell cycle |
| PRKDC | protein kinase, DNA-activated, catalytic polypeptide | DNA-PK phosphorylates threonines 5 and 7 of HSP90α, decreasing its chaperone function for clients such as AMP-activated protein kinase (AMPK), which is critical for mitochondrial biogenesis and energy metabolism. | mitochondrial dysfunction | Cell cycle |
| TFDP1 | transcription factor Dp-1 | TFDP1 is a transcription factor that may play a role in cell cycle regulation and apoptosis. | cellular senescence | Cell cycle |
| YWHAZ | tyrosine 3-monooxygenase/tryptophan 5-monooxygenase activation protein, zeta | YWHAZ interacts with so many proteins, including many associated with ageing such as the INS/IGF1 pathway, may be involved in stem cell development. | stem cell exhaustion | Cell cycle |
| BUB3 | BUB3 mitotic checkpoint protein | It has a dual function in spindle-assembly checkpoint signaling and in promoting the establishment of correct kinetochore-microtubule (K-MT) attachments. Promotes the formation of stable end-on bipolar attachments. Necessary for kinetochore localization of BUB1. Regulates chromosome segregation during oocyte meiosis. The BUB1/BUB3 complex plays a role in the inhibition of anaphase-promoting complex or cyclosome (APC/C) when spindle-assembly checkpoint is activated and inhibits the ubiquitin ligase activity of APC/C by phosphorylating its activator CDC20. This complex can also phosphorylate MAD1L1. | others | Cell cycle |
| PECAM1 | Platelet And Endothelial Cell Adhesion Molecule 1 | Cell adhesion molecule which is required for leukocyte transendothelial migration (TEM) under most inflammatory conditions (PubMed:19342684, PubMed:17580308). Tyr-690 plays a critical role in TEM and is required for efficient trafficking of PECAM1 to and from the lateral border recycling compartment (LBRC) and is also essential for the LBRC membrane to be targeted around migrating leukocytes (PubMed:19342684). Trans-homophilic interaction may play a role in endothelial cell-cell adhesion via cell junctions (PubMed:27958302). Heterophilic interaction with CD177 plays a role in transendothelial migration of neutrophils (PubMed:17580308). Homophilic ligation of PECAM1 prevents macrophage-mediated phagocytosis of neighboring viable leukocytes by transmitting a detachment signal (PubMed:12110892). Promotes macrophage-mediated phagocytosis of apoptotic leukocytes by tethering them to the phagocytic cells; PECAM1-mediated detachment signal appears to be disabled in apoptotic leukocytes (PubMed:12110892). Modulates bradykinin receptor BDKRB2 activation (PubMed:18672896). Regulates bradykinin- and hyperosmotic shock-induced ERK1/2 activation in endothelial cells (PubMed:18672896). Induces susceptibility to atherosclerosis (By similarity). | senescence-associated secretory phenotype | Cell adhesion molecules |
| MYLK | Myosin Light Chain Kinase | Calcium/calmodulin-dependent myosin light chain kinase implicated in smooth muscle contraction via phosphorylation of myosin light chains (MLC). Also regulates actin-myosin interaction through a non-kinase activity. Phosphorylates PTK2B/PYK2 and myosin light-chains. Involved in the inflammatory response (e.g. apoptosis, vascular permeability, leukocyte diapedesis), cell motility and morphology, airway hyperreactivity and other activities relevant to asthma. Required for tonic airway smooth muscle contraction that is necessary for physiological and asthmatic airway resistance. Necessary for gastrointestinal motility. | others | Calcium signaling pathway |
| HMGB1 | high mobility group box 1 | It is related to DNA unwinding, stress response, and maintenance of genome stability. | genomic instability | Autophagy - animal |
| PRKCD | protein kinase C, delta | Calcium-independent, phospholipid- and diacylglycerol (DAG)-dependent serine/threonine-protein kinase that plays contrasting roles in cell death and cell survival by functioning as a pro-apoptotic protein during DNA damage-induced apoptosis, but acting as an anti-apoptotic protein during cytokine receptor-initiated cell death, is involved in tumor suppression as well as survival of several cancers, is required for oxygen radical production by NADPH oxidase and acts as positive or negative regulator in platelet functional responses. | cellular senescence | Autophagy |
| AIFM1 | apoptosis-inducing factor, mitochondrion-associated, 1 | AIFM1 is an important player in apoptosis. Like cytochrome c, AIF is localized to mitochondria and released in response to death stimuli. | mitochondrial dysfunction | Apoptosis |
| HTRA2 | HtrA serine peptidase 2 | Serine protease that shows proteolytic activity against a non-specific substrate beta-casein. Promotes or induces cell death either by direct binding to and inhibition of BIRC proteins (also called inhibitor of apoptosis proteins, IAPs), leading to an increase in caspase activity, or by a BIRC inhibition-independent, caspase-independent and serine protease activity-dependent mechanism. Cleaves THAP5 and promotes its degradation during apoptosis. Isoform 2 seems to be proteolytically inactive. | loss of proteostasis | Apoptosis |
| BAK1 | BCL2-antagonist/killer 1 | Plays a role in the mitochondrial apoptosic process. Upon arrival of cell death signals, promotes mitochondrial outer membrane (MOM) permeabilization by oligomerizing to form pores within the MOM. This releases apoptogenic factors into the cytosol, including cytochrome c, promoting the activation of caspase 9 which in turn processes and activates the effector caspases. | mitochondrial dysfunction | Apoptosis |
| LMNA | lamin A/C | Lamins are components of the nuclear lamina, a fibrous layer on the nucleoplasmic side of the inner nuclear membrane, which is thought to provide a framework for the nuclear envelope and may also interact with chromatin. | genomic instability | Apoptosis |
| LMNB1 | lamin B1 | Lamins are components of the nuclear lamina, a fibrous layer on the nucleoplasmic side of the inner nuclear membrane, which is thought to provide a framework for the nuclear envelope and may also interact with chromatin. | cellular senescence | Apoptosis |
| DCTN1 | Dynactin Subunit 1 | DCTN1 plays a key role in dynein-mediated retrograde transport of vesicles and organelles along microtubules by recruiting and tethering dynein to microtubules. Binds to both dynein and microtubules providing a link between specific cargos, microtubules and dynein. Essential for targeting dynein to microtubule plus ends, recruiting dynein to membranous cargos and enhancing dynein processivity (the ability to move along a microtubule for a long distance without falling off the track). Can also act as a brake to slow the dynein motor during motility along the microtubule (PubMed:25185702). Can regulate microtubule stability by promoting microtubule formation, nucleation and polymerization and by inhibiting microtubule catastrophe in neurons. Inhibits microtubule catastrophe by binding both to microtubules and to tubulin, leading to enhanced microtubule stability along the axon (PubMed:23874158). Plays a role in metaphase spindle orientation (PubMed:22327364). Plays a role in centriole cohesion and subdistal appendage organization and function. Its recruitment to the centriole in a KIF3A-dependent manner is essential for the maintenance of centriole cohesion and the formation of subdistal appendage. Also required for microtubule anchoring at the mother centriole (PubMed:23386061). Plays a role in primary cilia formation. | others | Amyotrophic lateral sclerosis (ALS) |
| AARS1 | Alanyl-TRNA Synthetase 1 | Catalyzes the attachment of alanine to tRNA (Ala) in a two-step reaction: alanine is first activated by ATP to form Ala-AMP and then transferred to the acceptor end of tRNA (Ala) (PubMed:27622773, PubMed:27911835, PubMed:28493438). Also edits incorrectly charged tRNA (Ala) via its editing domain. | loss of proteostasis | Aminoacyl-tRNA biosynthesis |
| APOE | apolipoprotein E | APOE activates a non-canonical MAP kinase cascade that enhances APP transcription and amyloid-β synthesis. Several polymorphisms in the APOE gene have been associated with Alzheimer’s disease. | others | Alzheimer disease |
| APP | amyloid beta (A4) precursor protein | APP is an important player in Alzheimer's disease. | others | Alzheimer disease |
| IL1A | interleukin 1 alpha | Reduced IL1A diminished NF-κB transcriptional activity, which controls much of the SASP; exogenous IL1A restored IL6 secretion to rapamycin-treated cells. | senescence-associated secretory phenotype | Alzheimer disease |
| SDHC | succinate dehydrogenase complex, subunit C, integral membrane protein, 15kDa | A member of the mitochondrial electron transport chain, SDHC homologues have been implicated in ageing of lower life forms. Mutations in the human SDHC gene have been associated with mitochondrial respiratory chain deficiency. | mitochondrial dysfunction | Alzheimer disease |
| TNFRSF1A | TNF Receptor Superfamily Member 1A | Receptor for TNFSF2/TNF-alpha and homotrimeric TNFSF1/lymphotoxin-alpha. The adapter molecule FADD recruits caspase-8 to the activated receptor. The resulting death-inducing signaling complex (DISC) performs caspase-8 proteolytic activation which initiates the subsequent cascade of caspases (aspartate-specific cysteine proteases) mediating apoptosis. Contributes to the induction of non-cytocidal TNF effects including anti-viral state and activation of the acid sphingomyelinase. | senescence-associated secretory phenotype | MAPK signaling pathway |
| VEGFC | Vascular Endothelial Growth Factor C | Growth factor active in angiogenesis, and endothelial cell growth, stimulating their proliferation and migration and also has effects on the permeability of blood vessels. May function in angiogenesis of the venous and lymphatic vascular systems during embryogenesis, and also in the maintenance of differentiated lymphatic endothelium in adults. Binds and activates KDR/VEGFR2 and FLT4/VEGFR3 receptors. | senescence-associated secretory phenotype | MAPK signaling pathway |
| GRIA2 | Glutamate Ionotropic Receptor AMPA Type Subunit 2 | Receptor for glutamate that functions as ligand-gated ion channel in the central nervous system and plays an important role in excitatory synaptic transmission. L-glutamate acts as an excitatory neurotransmitter at many synapses in the central nervous system. Binding of the excitatory neurotransmitter L-glutamate induces a conformation change, leading to the opening of the cation channel, and thereby converts the chemical signal to an electrical impulse. The receptor then desensitizes rapidly and enters a transient inactive state, characterized by the presence of bound agonist. In the presence of CACNG4 or CACNG7 or CACNG8, shows resensitization which is characterized by a delayed accumulation of current flux upon continued application of glutamate. Through complex formation with NSG1, GRIP1 and STX12 controls the intracellular fate of AMPAR and the endosomal sorting of the GRIA2 subunit toward recycling and membrane targeting. | others | cAMP signaling pathway |
