# Supplementary material for: Identification and validation of a 7-genes prognostic signature for adult acute myeloid leukemia based on aging-related genes
Source: Aging (Albany NY). 2023 Jun 26;15(12):5826–53. doi: 10.18632/aging.204843 (PMC10333094; doi:10.18632/aging.204843)
Supplement: Supplementary Table 7 [file aging-15-204843-s008.pdf]

Supplementary Table 7. Pearson correlation analysis for scanning candidate therapeutic drugs.

Drug	Correlation coefficient	P value
Temozolomide_1375	-0.498212053	7.55E-11
PF-4708671_1129	-0.456825471	3.73E-09
Dactolisib_1057	-0.449715456	6.92E-09
5-Fluorouracil_1073	-0.433863895	2.62E-08
Entospletinib_1630	-0.431496025	3.18E-08
GNE-317_1926	-0.428702113	3.98E-08
Docetaxel_1007	-0.422628101	6.46E-08
Buparlisib_1873	-0.404751894	0.000000254
Pictilisib_1058	-0.401218856	0.00000033
VE821_2111	-0.398435368	0.000000405
MK-1775_1179	-0.384249796	0.00000111
AZD6738_1917	-0.383087068	0.0000012
Ribociclib_1632	-0.378656045	0.00000163
I-BRD9_1928	-0.378593752	0.00000164
Epirubicin_1511	-0.374221827	0.00000221
Axitinib_1021	-0.371145192	0.00000271
Bortezomib_1191	-0.366131208	0.00000378
YK-4-279_1239	-0.365077256	0.00000405
BDP-00009066_1866	-0.361748895	0.00000502
Camptothecin_1003	-0.357320265	0.00000667
Dabrafenib_1373	-0.351902382	0.00000938
BMS-345541_1249	-0.347824221	0.0000121
LGK974_1598	-0.347573101	0.0000123
Pevonedistat_1529	-0.346222958	0.0000133
Rapamycin_1084	-0.344928758	0.0000144
Luminespib_1559	-0.327708687	0.00004
BMS-536924_1091	-0.32390617	0.0000497
Leflunomide_1578	-0.321775903	0.000056
Wnt-C59_1622	-0.315870815	0.0000779
Dihydrorotenone_1827	-0.314568802	0.0000836
OTX015_1626	-0.31079521	0.000102746
Wee1 Inhibitor_1046	-0.309397471	0.000110801
Palbociclib_1054	-0.305193518	0.000138723
Ulixertinib_2047	-0.305018131	0.000140019
Taselisib_1561	-0.304662036	0.000142686
Sinularin_1838	-0.304160416	0.000146524
Vincristine_1818	-0.301715921	0.000166641
