# Supplementary material for: Identification and validation of a 7-genes prognostic signature for adult acute myeloid leukemia based on aging-related genes
Source: Aging (Albany NY). 2023 Jun 26;15(12):5826–53. doi: 10.18632/aging.204843 (PMC10333094; doi:10.18632/aging.204843)
Supplement: Supplementary Table 8 [file aging-15-204843-s009.docx]

**Supplementary Table 8. Predicted IC50 of candidate therapeutic drugs.**

| **TCGA ID** | Group | Bortezomib_1191 | Docetaxel_1007 | Camptothecin_1003 | Luminespib_1559 | Rapamycin_1084 | Vincristine_1818 | Dactolisib_1057 | Epirubicin_1511 |
| --- | --- | --- | --- | --- | --- | --- | --- | --- | --- |
| TCGA-AB-2805-03A-01T-0734-13 | Low risk | 0.007256 | 0.0124394 | 0.1081766 | 0.1018626 | 0.109469 | 0.1349434 | 0.1689514 | 0.2923115 |
| TCGA-AB-2806-03A-01T-0734-13 | Low risk | 0.0050627 | 0.0108108 | 0.1009345 | 0.0887672 | 0.1116202 | 0.0844995 | 0.283497 | 0.3572642 |
| TCGA-AB-2808-03A-01T-0734-13 | Low risk | 0.0072686 | 0.0088593 | 0.0714827 | 0.0810636 | 0.1317143 | 0.1019483 | 0.3055986 | 0.3691643 |
| TCGA-AB-2810-03A-01T-0736-13 | High risk | 0.0134547 | 0.0090525 | 0.0856109 | 0.1541706 | 0.1790449 | 0.3050084 | 0.327912 | 0.4841168 |
| TCGA-AB-2811-03B-01T-0760-13 | High risk | 0.0140546 | 0.0072944 | 0.0483633 | 0.1072326 | 0.1435358 | 0.2428874 | 0.1651565 | 0.2861613 |
| TCGA-AB-2812-03A-01T-0734-13 | Low risk | 0.0102855 | 0.0161083 | 0.1120125 | 0.0905693 | 0.1880326 | 0.2441203 | 0.3736115 | 0.6252698 |
| TCGA-AB-2813-03A-01T-0736-13 | High risk | 0.0108601 | 0.0110068 | 0.1267214 | 0.1988722 | 0.2395982 | 0.3393187 | 0.2846963 | 0.5260026 |
| TCGA-AB-2814-03A-01T-0734-13 | High risk | 0.0079466 | 0.0101894 | 0.0982808 | 0.0695976 | 0.1303935 | 0.1143879 | 0.2021802 | 0.4113384 |
| TCGA-AB-2815-03A-01T-0734-13 | Low risk | 0.0096998 | 0.0181475 | 0.1817663 | 0.1168989 | 0.2535246 | 0.344074 | 0.3166676 | 0.5214632 |
| TCGA-AB-2817-03A-01T-0736-13 | High risk | 0.006863 | 0.0114792 | 0.040715 | 0.0581328 | 0.0611585 | 0.0732754 | 0.114485 | 0.1665125 |
| TCGA-AB-2818-03A-01T-0734-13 | Low risk | 0.0075649 | 0.0089478 | 0.1018536 | 0.0695247 | 0.0806041 | 0.0925389 | 0.1652249 | 0.3516724 |
| TCGA-AB-2819-03A-01T-0734-13 | Low risk | 0.0054305 | 0.0082439 | 0.0596585 | 0.0738803 | 0.0884051 | 0.1024981 | 0.1950285 | 0.2329692 |
| TCGA-AB-2820-03A-01T-0735-13 | High risk | 0.0065437 | 0.0155051 | 0.0743461 | 0.0864878 | 0.1422609 | 0.3299232 | 0.1954282 | 0.4317865 |
| TCGA-AB-2821-03A-01T-0735-13 | Low risk | 0.0080816 | 0.0111418 | 0.0820191 | 0.1300756 | 0.0727032 | 0.1610672 | 0.1625504 | 0.4665755 |
| TCGA-AB-2822-03A-01T-0734-13 | Low risk | 0.006748 | 0.0160526 | 0.1005749 | 0.1441604 | 0.1338774 | 0.2032611 | 0.2730485 | 0.5926861 |
| TCGA-AB-2823-03A-01T-0736-13 | High risk | 0.0057114 | 0.0098677 | 0.164452 | 0.0613575 | 0.1351679 | 0.1288187 | 0.2152127 | 0.3645171 |
| TCGA-AB-2825-03A-01T-0736-13 | High risk | 0.0067133 | 0.0075213 | 0.1128062 | 0.0747933 | 0.0837384 | 0.0863787 | 0.1447301 | 0.2390063 |
| TCGA-AB-2826-03A-01T-0734-13 | Low risk | 0.015187 | 0.0141037 | 0.1311948 | 0.1622426 | 0.27106 | 0.457941 | 0.3135425 | 0.4045918 |
| TCGA-AB-2828-03A-01T-0734-13 | Low risk | 0.0080198 | 0.009836 | 0.1183516 | 0.0866644 | 0.1766383 | 0.1256232 | 0.216669 | 0.320241 |
| TCGA-AB-2830-03A-01T-0736-13 | High risk | 0.0055626 | 0.0097807 | 0.0667021 | 0.0857775 | 0.0822519 | 0.1251119 | 0.1327404 | 0.3245026 |
| TCGA-AB-2834-03A-01T-0734-13 | Low risk | 0.0098387 | 0.0126684 | 0.19317 | 0.1337872 | 0.2393589 | 0.2588408 | 0.3728496 | 0.4933062 |
| TCGA-AB-2835-03A-01T-0736-13 | High risk | 0.0075318 | 0.0058368 | 0.097664 | 0.0664655 | 0.1137225 | 0.1148251 | 0.1798837 | 0.2369303 |
| TCGA-AB-2836-03A-01T-0736-13 | High risk | 0.0055979 | 0.0068577 | 0.0559769 | 0.0554874 | 0.0601208 | 0.0745561 | 0.1276027 | 0.2097865 |
| TCGA-AB-2839-03A-01T-0734-13 | Low risk | 0.0072257 | 0.0146767 | 0.1293526 | 0.113798 | 0.1711631 | 0.2119761 | 0.3226267 | 0.597482 |
| TCGA-AB-2840-03A-01T-0734-13 | Low risk | 0.0106187 | 0.0181919 | 0.1661319 | 0.1085844 | 0.1551269 | 0.3057921 | 0.301247 | 0.5584793 |
| TCGA-AB-2841-03B-01T-0760-13 | Low risk | 0.0134068 | 0.0189935 | 0.1983722 | 0.2449221 | 0.285223 | 0.8397041 | 0.3231523 | 0.6593425 |
| TCGA-AB-2842-03A-01T-0734-13 | High risk | 0.0126849 | 0.0240049 | 0.2692122 | 0.1250115 | 0.3391799 | 0.5059801 | 0.4213355 | 0.7043668 |
| TCGA-AB-2843-03A-01T-0736-13 | High risk | 0.00555 | 0.0064668 | 0.0687803 | 0.063708 | 0.0513816 | 0.052441 | 0.1215659 | 0.2149134 |
| TCGA-AB-2844-03A-01T-0736-13 | Low risk | 0.006524 | 0.0054742 | 0.0554306 | 0.0554848 | 0.058848 | 0.0502701 | 0.1644019 | 0.2569475 |
| TCGA-AB-2845-03B-01T-0748-13 | High risk | 0.0050652 | 0.0050615 | 0.0362808 | 0.0696377 | 0.062708 | 0.0382516 | 0.1869267 | 0.2068656 |
| TCGA-AB-2846-03A-01T-0736-13 | High risk | 0.0065931 | 0.0082004 | 0.0868784 | 0.0784035 | 0.0921026 | 0.0799604 | 0.1801794 | 0.3043832 |
| TCGA-AB-2847-03A-01T-0736-13 | High risk | 0.0066951 | 0.0067444 | 0.077157 | 0.0463945 | 0.0937423 | 0.0899984 | 0.1287027 | 0.2776955 |
| TCGA-AB-2849-03A-01T-0734-13 | Low risk | 0.0090173 | 0.0291549 | 0.2172838 | 0.1157641 | 0.18798 | 0.1960531 | 0.4865064 | 0.6024659 |
| TCGA-AB-2851-03A-01T-0736-13 | High risk | 0.0068457 | 0.0097093 | 0.0974835 | 0.1031282 | 0.1112396 | 0.1658571 | 0.1692427 | 0.2733569 |
| TCGA-AB-2853-03A-01T-0734-13 | Low risk | 0.006486 | 0.0073981 | 0.0657593 | 0.086693 | 0.1115534 | 0.1334095 | 0.2200172 | 0.332537 |
| TCGA-AB-2856-03A-01T-0736-13 | High risk | 0.0063289 | 0.0047156 | 0.0459354 | 0.0678481 | 0.0812958 | 0.110175 | 0.1027444 | 0.161884 |
| TCGA-AB-2857-03A-01T-0736-13 | High risk | 0.0045512 | 0.0080467 | 0.061261 | 0.0742592 | 0.055003 | 0.0871897 | 0.1314777 | 0.3595543 |
| TCGA-AB-2858-03A-01T-0736-13 | Low risk | 0.0061166 | 0.0103758 | 0.0697566 | 0.1322879 | 0.1223366 | 0.1400838 | 0.2337309 | 0.2938359 |
| TCGA-AB-2859-03A-01T-0736-13 | High risk | 0.0054295 | 0.0097054 | 0.0704705 | 0.0791828 | 0.065099 | 0.1182068 | 0.1315631 | 0.3363473 |
| TCGA-AB-2861-03A-01T-0736-13 | Low risk | 0.0097744 | 0.0054882 | 0.0510471 | 0.0557177 | 0.062106 | 0.0944895 | 0.0876958 | 0.1355196 |
| TCGA-AB-2862-03A-01T-0736-13 | Low risk | 0.0070376 | 0.0088566 | 0.0962948 | 0.0778639 | 0.0978642 | 0.1334749 | 0.1622876 | 0.3186985 |
| TCGA-AB-2863-03A-01T-0734-13 | High risk | 0.0056619 | 0.0090378 | 0.067924 | 0.0967048 | 0.0999814 | 0.1290298 | 0.1658212 | 0.4204413 |
| TCGA-AB-2865-03A-01T-0736-13 | High risk | 0.0051754 | 0.0111605 | 0.1066682 | 0.0973546 | 0.1067808 | 0.1528182 | 0.2013176 | 0.2682967 |
| TCGA-AB-2866-03A-01T-0736-13 | High risk | 0.0086707 | 0.0087758 | 0.0682159 | 0.0700814 | 0.0816005 | 0.1499762 | 0.1646665 | 0.213346 |
| TCGA-AB-2867-03A-01T-0734-13 | Low risk | 0.0084411 | 0.0152869 | 0.1035478 | 0.1967287 | 0.2491168 | 0.2180028 | 0.4666715 | 0.5639418 |
| TCGA-AB-2869-03A-01T-0735-13 | Low risk | 0.0048303 | 0.0066081 | 0.0407945 | 0.0567739 | 0.054039 | 0.0612983 | 0.1316763 | 0.2679886 |
| TCGA-AB-2870-03A-01T-0735-13 | High risk | 0.0054807 | 0.0081108 | 0.057153 | 0.0833921 | 0.0912403 | 0.0752826 | 0.1708908 | 0.2274663 |
| TCGA-AB-2871-03A-01T-0735-13 | Low risk | 0.0049811 | 0.00733 | 0.0423571 | 0.0655142 | 0.0465478 | 0.0670915 | 0.1089163 | 0.2224883 |
| TCGA-AB-2872-03A-01T-0735-13 | Low risk | 0.0161999 | 0.0164836 | 0.1868383 | 0.1991611 | 0.2054889 | 0.4006539 | 0.2861386 | 0.7788818 |
| TCGA-AB-2873-03A-01T-0735-13 | High risk | 0.0125339 | 0.0156819 | 0.186684 | 0.1724507 | 0.1367852 | 0.3541681 | 0.2183347 | 0.4572552 |
| TCGA-AB-2874-03A-01T-0735-13 | Low risk | 0.0069787 | 0.0099134 | 0.0682931 | 0.0730832 | 0.1298794 | 0.1068752 | 0.2535848 | 0.3338341 |
| TCGA-AB-2875-03A-01T-0735-13 | Low risk | 0.0043861 | 0.0076703 | 0.0678004 | 0.0796107 | 0.0835493 | 0.0601687 | 0.1794979 | 0.2371732 |
| TCGA-AB-2876-03A-01T-0734-13 | Low risk | 0.0076618 | 0.0104953 | 0.0690313 | 0.1197175 | 0.1806807 | 0.16781 | 0.3998667 | 0.4403655 |
| TCGA-AB-2877-03A-01T-0735-13 | Low risk | 0.0077407 | 0.006713 | 0.0563538 | 0.0745908 | 0.0656233 | 0.1049844 | 0.1840091 | 0.2672105 |
| TCGA-AB-2878-03A-01T-0734-13 | Low risk | 0.0128358 | 0.0153126 | 0.1322657 | 0.2180898 | 0.2978215 | 0.4689472 | 0.3350078 | 0.8553268 |
| TCGA-AB-2880-03A-01T-0735-13 | Low risk | 0.0049288 | 0.0051371 | 0.04567 | 0.0697402 | 0.08293 | 0.0458216 | 0.189135 | 0.2441981 |
| TCGA-AB-2881-03A-01T-0735-13 | High risk | 0.0054683 | 0.0084991 | 0.0612402 | 0.0733007 | 0.0989991 | 0.09755 | 0.1343025 | 0.2531341 |
| TCGA-AB-2882-03A-01T-0740-13 | Low risk | 0.0076538 | 0.0172852 | 0.116113 | 0.1554815 | 0.116531 | 0.2678175 | 0.2162004 | 0.501187 |
| TCGA-AB-2883-03A-01T-0734-13 | Low risk | 0.0194113 | 0.0201118 | 0.1526619 | 0.1353736 | 0.1461067 | 0.5177031 | 0.0972679 | 0.4654353 |
| TCGA-AB-2884-03A-01T-0735-13 | High risk | 0.0064933 | 0.0070953 | 0.0853283 | 0.0874459 | 0.0690611 | 0.0947399 | 0.1460559 | 0.2369287 |
| TCGA-AB-2885-03A-01T-0735-13 | High risk | 0.0059979 | 0.0084488 | 0.0503278 | 0.0543787 | 0.0841254 | 0.2446374 | 0.1317159 | 0.2863396 |
| TCGA-AB-2886-03A-01T-0735-13 | Low risk | 0.0042738 | 0.0082976 | 0.0625164 | 0.0734578 | 0.0780188 | 0.050647 | 0.186508 | 0.2668715 |
| TCGA-AB-2888-03B-01T-0748-13 | High risk | 0.0071289 | 0.0072722 | 0.0598321 | 0.0764117 | 0.0889469 | 0.0904228 | 0.1465024 | 0.1777893 |
| TCGA-AB-2889-03A-01T-0735-13 | Low risk | 0.0068736 | 0.0095139 | 0.0865276 | 0.0916361 | 0.1284965 | 0.1318251 | 0.185867 | 0.3341953 |
| TCGA-AB-2890-03A-01T-0735-13 | High risk | 0.0060803 | 0.0128257 | 0.0912079 | 0.1040712 | 0.1010665 | 0.1379193 | 0.1858298 | 0.2667547 |
| TCGA-AB-2891-03A-01T-0735-13 | High risk | 0.0043542 | 0.0049034 | 0.0575744 | 0.0717269 | 0.0578756 | 0.0335673 | 0.1386041 | 0.1489463 |
| TCGA-AB-2892-03A-01T-0734-13 | Low risk | 0.0124753 | 0.0216202 | 0.2122415 | 0.1953201 | 0.3585974 | 0.4598733 | 0.3181606 | 0.6554368 |
| TCGA-AB-2893-03A-01T-0734-13 | High risk | 0.0083548 | 0.0092325 | 0.128567 | 0.0582575 | 0.1313858 | 0.2816531 | 0.1518965 | 0.223893 |
| TCGA-AB-2894-03A-01T-0734-13 | Low risk | 0.010482 | 0.0142256 | 0.0949655 | 0.0450603 | 0.0905237 | 0.1196984 | 0.153601 | 0.2701965 |
| TCGA-AB-2895-03A-01T-0735-13 | High risk | 0.0051986 | 0.0086474 | 0.0586258 | 0.0603043 | 0.0532912 | 0.0986086 | 0.1540527 | 0.3274378 |
| TCGA-AB-2896-03B-01T-0751-13 | High risk | 0.0056808 | 0.0060561 | 0.0367941 | 0.0644184 | 0.0529813 | 0.0732016 | 0.124887 | 0.1964663 |
| TCGA-AB-2897-03A-01T-0735-13 | High risk | 0.0099341 | 0.0137623 | 0.1555494 | 0.1508632 | 0.2249469 | 0.3246875 | 0.3515078 | 0.7226663 |
| TCGA-AB-2898-03A-01T-0735-13 | High risk | 0.0089312 | 0.0132857 | 0.151619 | 0.1778959 | 0.1460012 | 0.2346981 | 0.2487508 | 0.6175468 |
| TCGA-AB-2899-03A-01T-0736-13 | Low risk | 0.00761 | 0.0145576 | 0.0803036 | 0.0903128 | 0.0629851 | 0.0904889 | 0.1453392 | 0.3012515 |
| TCGA-AB-2900-03A-01T-0735-13 | High risk | 0.0088911 | 0.0089441 | 0.1108057 | 0.0983039 | 0.1228405 | 0.182087 | 0.2143864 | 0.3919225 |
| TCGA-AB-2901-03A-01T-0735-13 | High risk | 0.0050216 | 0.0107278 | 0.1959858 | 0.0696828 | 0.1133884 | 0.3652742 | 0.1593366 | 0.348888 |
| TCGA-AB-2908-03A-01T-0740-13 | High risk | 0.0082637 | 0.0097004 | 0.0796464 | 0.155839 | 0.2116759 | 0.4827699 | 0.2506979 | 0.6282245 |
| TCGA-AB-2910-03A-01T-0740-13 | High risk | 0.0067593 | 0.0068773 | 0.0706607 | 0.0444454 | 0.0719644 | 0.0897375 | 0.1085084 | 0.1376772 |
| TCGA-AB-2911-03A-01T-0734-13 | Low risk | 0.0155866 | 0.0260055 | 0.1955687 | 0.2494423 | 0.3857542 | 0.7204429 | 0.345788 | 0.7162645 |
| TCGA-AB-2912-03A-01T-0734-13 | High risk | 0.0130274 | 0.0175309 | 0.169693 | 0.1355846 | 0.1793154 | 0.2531153 | 0.2183173 | 0.5288379 |
| TCGA-AB-2913-03A-01T-0734-13 | Low risk | 0.0120617 | 0.0276153 | 0.1473607 | 0.2191701 | 0.2143261 | 0.4752131 | 0.2844639 | 0.7560436 |
| TCGA-AB-2914-03A-01T-0734-13 | Low risk | 0.0086003 | 0.0216308 | 0.1482624 | 0.1823298 | 0.2760417 | 0.2363493 | 0.3605773 | 0.589316 |
| TCGA-AB-2915-03A-01T-0740-13 | High risk | 0.0059728 | 0.0052353 | 0.0516128 | 0.0583014 | 0.0631139 | 0.0772951 | 0.1167984 | 0.2209408 |
| TCGA-AB-2916-03A-01T-0734-13 | Low risk | 0.0123254 | 0.0264305 | 0.1507549 | 0.2824697 | 0.2898626 | 0.5066239 | 0.2819369 | 0.6150956 |
| TCGA-AB-2917-03A-01T-0734-13 | Low risk | 0.0102478 | 0.0329801 | 0.1330063 | 0.2276558 | 0.2228358 | 0.37461 | 0.520822 | 0.6894972 |
| TCGA-AB-2918-03A-01T-0740-13 | High risk | 0.0066974 | 0.0058396 | 0.0463436 | 0.0739464 | 0.0827787 | 0.0673915 | 0.147073 | 0.2428579 |
| TCGA-AB-2919-03A-01T-0740-13 | Low risk | 0.0050543 | 0.0047218 | 0.0471774 | 0.0511111 | 0.0397957 | 0.0442818 | 0.1148984 | 0.2130898 |
| TCGA-AB-2920-03B-01T-0760-13 | High risk | 0.0102775 | 0.0121081 | 0.0465308 | 0.1526069 | 0.2252566 | 0.2211049 | 0.1741896 | 0.4100751 |
| TCGA-AB-2921-03A-01T-0740-13 | High risk | 0.0048436 | 0.0057195 | 0.037738 | 0.0517449 | 0.0470604 | 0.0682687 | 0.1085469 | 0.1515115 |
| TCGA-AB-2924-03A-01T-0740-13 | High risk | 0.0062852 | 0.0059318 | 0.0618434 | 0.0764519 | 0.0789359 | 0.0829092 | 0.1552787 | 0.2375936 |
| TCGA-AB-2925-03A-01T-0735-13 | High risk | 0.0087367 | 0.0073348 | 0.0765474 | 0.0715643 | 0.0921221 | 0.1805446 | 0.1467466 | 0.2219578 |
| TCGA-AB-2927-03A-01T-0740-13 | High risk | 0.0108959 | 0.0199961 | 0.0946867 | 0.1335909 | 0.1163409 | 0.3318554 | 0.1752068 | 0.3708455 |
| TCGA-AB-2928-03A-01T-0740-13 | High risk | 0.0047646 | 0.0072211 | 0.0401598 | 0.0462647 | 0.067818 | 0.1122965 | 0.1367658 | 0.2024432 |
| TCGA-AB-2929-03A-01T-0735-13 | High risk | 0.0037242 | 0.0098303 | 0.0609844 | 0.0550425 | 0.0510634 | 0.0816922 | 0.1264159 | 0.1577633 |
| TCGA-AB-2930-03A-01T-0740-13 | High risk | 0.0049426 | 0.003966 | 0.035262 | 0.0510061 | 0.0602756 | 0.033728 | 0.1289319 | 0.16404 |
| TCGA-AB-2931-03A-01T-0740-13 | High risk | 0.0046626 | 0.0050274 | 0.0436175 | 0.0495004 | 0.0532448 | 0.0762377 | 0.1089119 | 0.1991907 |
| TCGA-AB-2932-03A-01T-0740-13 | High risk | 0.0073701 | 0.0065954 | 0.080457 | 0.073406 | 0.0892712 | 0.1222825 | 0.1641101 | 0.2593552 |
| TCGA-AB-2933-03A-01T-0734-13 | High risk | 0.0132017 | 0.0258607 | 0.1265245 | 0.2371127 | 0.2333916 | 0.3633691 | 0.4048614 | 0.4860076 |
| TCGA-AB-2934-03A-01T-0740-13 | High risk | 0.0082372 | 0.0098945 | 0.0684727 | 0.1137696 | 0.0916163 | 0.2237072 | 0.1499559 | 0.2427274 |
| TCGA-AB-2935-03A-01T-0740-13 | High risk | 0.0071025 | 0.0055806 | 0.059537 | 0.0521607 | 0.0456023 | 0.0757165 | 0.0772846 | 0.2476876 |
| TCGA-AB-2936-03A-01T-0740-13 | Low risk | 0.0066088 | 0.0187513 | 0.0859144 | 0.2136882 | 0.0978861 | 0.1886631 | 0.1945639 | 0.3632824 |
| TCGA-AB-2937-03A-01T-0734-13 | Low risk | 0.0168718 | 0.0786288 | 0.4014264 | 0.8727128 | 1.2333575 | 3.5141083 | 0.8784905 | 1.3028027 |
| TCGA-AB-2938-03A-01T-0736-13 | High risk | 0.0042526 | 0.0070875 | 0.1858558 | 0.102303 | 0.0749685 | 0.1942306 | 0.1100961 | 0.6711243 |
| TCGA-AB-2939-03A-01T-0740-13 | High risk | 0.0052017 | 0.00749 | 0.0696495 | 0.0334787 | 0.064501 | 0.0881189 | 0.1315365 | 0.2211849 |
| TCGA-AB-2940-03A-01T-0736-13 | Low risk | 0.0054685 | 0.0072485 | 0.0796282 | 0.0696064 | 0.0855278 | 0.0769492 | 0.181699 | 0.3506303 |
| TCGA-AB-2941-03A-01T-0740-13 | High risk | 0.0086215 | 0.0079478 | 0.1749892 | 0.2884568 | 0.0838179 | 0.3548292 | 0.1470945 | 1.4104309 |
| TCGA-AB-2942-03A-01T-0734-13 | Low risk | 0.0088616 | 0.0116022 | 0.0785812 | 0.1242903 | 0.1940683 | 0.18321 | 0.207157 | 0.3693925 |
| TCGA-AB-2943-03A-01T-0740-13 | High risk | 0.0054467 | 0.0097803 | 0.0729079 | 0.1402868 | 0.1433439 | 0.2298606 | 0.2539021 | 0.5059091 |
| TCGA-AB-2944-03A-01T-0740-13 | High risk | 0.0051145 | 0.0083465 | 0.0567655 | 0.0534128 | 0.1067028 | 0.1499806 | 0.1298139 | 0.2296364 |
| TCGA-AB-2946-03A-01T-0740-13 | Low risk | 0.0056771 | 0.0058813 | 0.0459238 | 0.077999 | 0.0781126 | 0.0697225 | 0.2113682 | 0.3115911 |
| TCGA-AB-2948-03A-01T-0740-13 | High risk | 0.0089787 | 0.0065411 | 0.0612515 | 0.1086697 | 0.101341 | 0.098997 | 0.1683773 | 0.249345 |
| TCGA-AB-2949-03B-01T-0748-13 | High risk | 0.0061887 | 0.005617 | 0.0515282 | 0.070444 | 0.0814626 | 0.0891175 | 0.1843785 | 0.248814 |
| TCGA-AB-2950-03A-01T-0735-13 | Low risk | 0.0070355 | 0.0144938 | 0.1578659 | 0.133581 | 0.1240616 | 0.2303594 | 0.2556917 | 0.4552851 |
| TCGA-AB-2952-03B-01T-0760-13 | High risk | 0.0091914 | 0.0095328 | 0.0592718 | 0.1537146 | 0.2011627 | 0.2306967 | 0.2249632 | 0.3604066 |
| TCGA-AB-2955-03A-01T-0734-13 | Low risk | 0.0073274 | 0.0119629 | 0.078897 | 0.1248917 | 0.1777155 | 0.1505896 | 0.2734209 | 0.5088734 |
| TCGA-AB-2956-03A-01T-0740-13 | High risk | 0.0059751 | 0.0063157 | 0.0741708 | 0.0321808 | 0.0421585 | 0.0810689 | 0.1094693 | 0.1315217 |
| TCGA-AB-2959-03A-01T-0734-13 | Low risk | 0.009183 | 0.037044 | 0.1114996 | 0.1797125 | 0.1587248 | 0.273235 | 0.3513183 | 0.5396975 |
| TCGA-AB-2963-03A-01T-0734-13 | Low risk | 0.0074484 | 0.0073416 | 0.0793649 | 0.0993144 | 0.1311328 | 0.1140342 | 0.2261698 | 0.3566148 |
| TCGA-AB-2965-03A-01T-0734-13 | Low risk | 0.0091071 | 0.0125218 | 0.114796 | 0.0864404 | 0.1518722 | 0.2283727 | 0.2255012 | 0.3464433 |
| TCGA-AB-2966-03A-01T-0734-13 | Low risk | 0.0058394 | 0.0183188 | 0.0746609 | 0.1200826 | 0.1819318 | 0.260115 | 0.3164786 | 0.3577721 |
| TCGA-AB-2970-03A-01T-0734-13 | Low risk | 0.0088365 | 0.0160737 | 0.117392 | 0.1023326 | 0.1321823 | 0.1734894 | 0.1948911 | 0.4218333 |
| TCGA-AB-2971-03A-01T-0734-13 | Low risk | 0.010433 | 0.0137672 | 0.0736852 | 0.1131417 | 0.1383216 | 0.1554705 | 0.175852 | 0.3616546 |
| TCGA-AB-2973-03A-01T-0734-13 | Low risk | 0.0211985 | 0.0366887 | 0.2955851 | 0.380543 | 0.3609461 | 1.1568205 | 0.3047354 | 0.5718343 |
| TCGA-AB-2975-03A-01T-0734-13 | Low risk | 0.0109278 | 0.0113706 | 0.0489519 | 0.1483719 | 0.1634758 | 0.211371 | 0.2431223 | 0.3869112 |
| TCGA-AB-2976-03A-01T-0734-13 | Low risk | 0.0115004 | 0.014401 | 0.1054162 | 0.1388059 | 0.1431214 | 0.3124064 | 0.2648452 | 0.6114155 |
| TCGA-AB-2977-03B-01T-0760-13 | Low risk | 0.0134181 | 0.0092657 | 0.0788772 | 0.1517976 | 0.1559896 | 0.6126791 | 0.1391728 | 0.3543687 |
| TCGA-AB-2979-03B-01T-0760-13 | Low risk | 0.0051715 | 0.0050909 | 0.0420752 | 0.0990689 | 0.1356277 | 0.0668655 | 0.1942927 | 0.2522983 |
| TCGA-AB-2980-03A-01T-0734-13 | Low risk | 0.0075955 | 0.0104458 | 0.1073707 | 0.0937771 | 0.1554844 | 0.2638658 | 0.2336821 | 0.4554946 |
| TCGA-AB-2981-03B-01T-0748-13 | High risk | 0.0061972 | 0.0079406 | 0.0494534 | 0.081589 | 0.0895136 | 0.117201 | 0.1498421 | 0.1596393 |
| TCGA-AB-2982-03B-01T-0748-13 | Low risk | 0.0089946 | 0.0127677 | 0.1406215 | 0.1550923 | 0.0940285 | 0.2256517 | 0.1848199 | 0.4638923 |
| TCGA-AB-2983-03A-01T-0734-13 | High risk | 0.0081048 | 0.0162361 | 0.1002673 | 0.1047044 | 0.1169367 | 0.1632561 | 0.1993416 | 0.3799896 |
| TCGA-AB-2984-03A-01T-0734-13 | Low risk | 0.0344958 | 0.0371342 | 0.3948852 | 0.1812898 | 0.5563382 | 1.6613923 | 0.6908693 | 1.0538934 |
| TCGA-AB-2986-03A-01T-0734-13 | Low risk | 0.0093582 | 0.0100118 | 0.0742321 | 0.1414015 | 0.1704797 | 0.3654628 | 0.2087112 | 0.4261014 |
| TCGA-AB-2987-03A-01T-0734-13 | High risk | 0.0096229 | 0.0208604 | 0.1328657 | 0.1514938 | 0.1423102 | 0.4510333 | 0.1635275 | 0.2719609 |
| TCGA-AB-2988-03B-01T-0748-13 | High risk | 0.0048675 | 0.005531 | 0.0448284 | 0.0619462 | 0.0388827 | 0.0575421 | 0.1239844 | 0.2233008 |
| TCGA-AB-2990-03B-01T-0748-13 | Low risk | 0.0123108 | 0.0074517 | 0.0581407 | 0.1175443 | 0.1033962 | 0.2466641 | 0.1861172 | 0.3229406 |
| TCGA-AB-2991-03A-01T-0735-13 | High risk | 0.0070775 | 0.0112099 | 0.1295914 | 0.084766 | 0.1392867 | 0.133074 | 0.2784602 | 0.4182122 |
| TCGA-AB-2992-03A-01T-0735-13 | Low risk | 0.0145375 | 0.0149281 | 0.1321578 | 0.2699263 | 0.2196237 | 0.3716409 | 0.2856732 | 0.5268496 |
| TCGA-AB-2994-03A-01T-0735-13 | Low risk | 0.0071126 | 0.0093127 | 0.1163202 | 0.0647657 | 0.1210321 | 0.1401015 | 0.1756307 | 0.2927708 |
| TCGA-AB-2995-03A-01T-0735-13 | High risk | 0.0068711 | 0.0106697 | 0.058631 | 0.1147707 | 0.084997 | 0.1542212 | 0.1520283 | 0.393595 |
| TCGA-AB-2996-03A-01T-0735-13 | Low risk | 0.0091091 | 0.0101475 | 0.1108336 | 0.1423274 | 0.1006213 | 0.1802195 | 0.1805335 | 0.5596957 |
| TCGA-AB-2998-03A-01T-0735-13 | High risk | 0.0058341 | 0.0116142 | 0.1503002 | 0.0661303 | 0.1098263 | 0.1580088 | 0.2935605 | 0.3691814 |
| TCGA-AB-2999-03B-01T-0748-13 | Low risk | 0.0109614 | 0.0115108 | 0.1347447 | 0.1441588 | 0.1441322 | 0.2809922 | 0.2450234 | 0.4827936 |
| TCGA-AB-3000-03A-01T-0736-13 | Low risk | 0.0046261 | 0.0060549 | 0.0576848 | 0.0659882 | 0.0729291 | 0.0457209 | 0.1963588 | 0.2406634 |
| TCGA-AB-3001-03A-01T-0736-13 | Low risk | 0.007825 | 0.0131786 | 0.2156524 | 0.1253762 | 0.1344162 | 0.1286295 | 0.2703311 | 0.6056985 |
| TCGA-AB-3002-03A-01T-0736-13 | High risk | 0.0108133 | 0.0092501 | 0.0593059 | 0.0865435 | 0.1643877 | 0.1369879 | 0.2871656 | 0.3413436 |
| TCGA-AB-3007-03A-01T-0736-13 | High risk | 0.0070106 | 0.0119784 | 0.1274272 | 0.0789762 | 0.0827778 | 0.1968198 | 0.1444912 | 0.3943455 |
| TCGA-AB-3008-03A-01T-0736-13 | Low risk | 0.004729 | 0.0063726 | 0.0479679 | 0.092905 | 0.0909509 | 0.053889 | 0.2329805 | 0.3055516 |
| TCGA-AB-3009-03A-01T-0736-13 | High risk | 0.0061046 | 0.008661 | 0.0854325 | 0.036197 | 0.0968833 | 0.0470519 | 0.1917571 | 0.2452823 |
| TCGA-AB-3011-03A-01T-0736-13 | Low risk | 0.0068634 | 0.0089861 | 0.0719299 | 0.0724211 | 0.0562651 | 0.0929095 | 0.2053708 | 0.4146418 |
| TCGA-AB-3012-03A-01T-0736-13 | Low risk | 0.0086591 | 0.0117098 | 0.1861971 | 0.1013888 | 0.1429116 | 0.201667 | 0.2475525 | 0.6243045 |
| Median IC 50 | NA | 0.007256 | 0.0097807 | 0.0803036 | 0.0916361 | 0.1115534 | 0.1499806 | 0.186508 | 0.3506303 |

**Supplementary Table 8 (Continue)**

| **TCGA ID** | Group | MK-1775_1179 | GNE-317_1926 | Pevonedistat_1529 | Dihydrorotenone_1827 | Buparlisib_1873 | Pictilisib_1058 | Wee1 Inhibitor_1046 |
| --- | --- | --- | --- | --- | --- | --- | --- | --- |
| TCGA-AB-2805-03A-01T-0734-13 | Low risk | 1.5452318 | 1.5212155 | 1.1626138 | 2.6325598 | 2.7917524 | 4.0634765 | 5.5256498 |
| TCGA-AB-2806-03A-01T-0734-13 | Low risk | 1.3370989 | 2.1856467 | 1.3357091 | 2.2510768 | 2.4806902 | 4.8736635 | 4.5723351 |
| TCGA-AB-2808-03A-01T-0734-13 | Low risk | 1.4367823 | 2.1002939 | 1.3988157 | 1.9357074 | 2.6641491 | 6.8712819 | 6.126989 |
| TCGA-AB-2810-03A-01T-0736-13 | High risk | 1.6451111 | 1.957962 | 1.3522185 | 2.3451223 | 3.5371928 | 5.4415502 | 9.3655299 |
| TCGA-AB-2811-03B-01T-0760-13 | High risk | 1.1142922 | 1.3287902 | 1.4749598 | 4.5143911 | 3.713547 | 3.4958052 | 7.9285177 |
| TCGA-AB-2812-03A-01T-0734-13 | Low risk | 2.3956658 | 2.2557062 | 3.5440447 | 3.1668144 | 3.1719206 | 6.4761189 | 8.3380208 |
| TCGA-AB-2813-03A-01T-0736-13 | High risk | 3.8395302 | 2.1027382 | 4.4256115 | 3.1499391 | 3.4859449 | 5.0312557 | 14.555987 |
| TCGA-AB-2814-03A-01T-0734-13 | High risk | 2.3163125 | 1.6767336 | 2.1767138 | 2.9903266 | 2.2119121 | 3.5272303 | 6.7291846 |
| TCGA-AB-2815-03A-01T-0734-13 | Low risk | 2.4637598 | 2.0117352 | 2.8475172 | 4.0219794 | 3.5971629 | 5.2320939 | 8.2110448 |
| TCGA-AB-2817-03A-01T-0736-13 | High risk | 0.9076318 | 1.200076 | 1.7494883 | 2.2638611 | 1.9309866 | 2.9305015 | 3.8128341 |
| TCGA-AB-2818-03A-01T-0734-13 | Low risk | 1.5223398 | 1.442481 | 1.3471296 | 2.2693362 | 2.3865774 | 3.3262149 | 6.848675 |
| TCGA-AB-2819-03A-01T-0734-13 | Low risk | 1.4674921 | 1.6954329 | 1.1006097 | 2.3203932 | 2.5485374 | 5.0895949 | 4.3044833 |
| TCGA-AB-2820-03A-01T-0735-13 | High risk | 2.9337405 | 1.9674404 | 2.7416128 | 2.5456335 | 2.775466 | 4.3734097 | 8.9968001 |
| TCGA-AB-2821-03A-01T-0735-13 | Low risk | 1.7381489 | 1.4598565 | 2.2115347 | 2.0884021 | 2.052208 | 2.736249 | 6.1942494 |
| TCGA-AB-2822-03A-01T-0734-13 | Low risk | 2.058844 | 2.1913224 | 1.3704577 | 3.1548999 | 2.9157578 | 5.0023523 | 7.2540866 |
| TCGA-AB-2823-03A-01T-0736-13 | High risk | 1.9266546 | 1.7876241 | 2.6689407 | 2.8018384 | 2.4403504 | 4.4890104 | 8.2015049 |
| TCGA-AB-2825-03A-01T-0736-13 | High risk | 1.5118858 | 1.3548919 | 1.0871135 | 2.1696411 | 2.3209352 | 2.6772223 | 5.3938894 |
| TCGA-AB-2826-03A-01T-0734-13 | Low risk | 3.2434861 | 1.8867468 | 2.3025674 | 3.3133596 | 3.9102959 | 5.9681907 | 11.559584 |
| TCGA-AB-2828-03A-01T-0734-13 | Low risk | 1.7905644 | 1.5667203 | 1.5474152 | 2.7886745 | 2.7625881 | 4.0341712 | 4.6888955 |
| TCGA-AB-2830-03A-01T-0736-13 | High risk | 1.9356559 | 1.3359457 | 1.8710491 | 2.4566885 | 2.3557445 | 2.7834224 | 8.0210629 |
| TCGA-AB-2834-03A-01T-0734-13 | Low risk | 1.6696775 | 2.3212847 | 1.8318257 | 2.0098346 | 3.286557 | 5.186198 | 7.2362368 |
| TCGA-AB-2835-03A-01T-0736-13 | High risk | 1.5623766 | 1.5107922 | 0.8285788 | 2.3507294 | 2.8052857 | 4.3342114 | 5.537917 |
| TCGA-AB-2836-03A-01T-0736-13 | High risk | 1.2062129 | 1.2979033 | 0.8319798 | 1.9504389 | 2.1525966 | 2.5551397 | 5.5625062 |
| TCGA-AB-2839-03A-01T-0734-13 | Low risk | 1.6973838 | 2.2138929 | 2.5978619 | 3.2259588 | 2.8980107 | 6.3385779 | 7.646584 |
| TCGA-AB-2840-03A-01T-0734-13 | Low risk | 3.3554147 | 2.1339238 | 6.1471149 | 2.6587286 | 3.2908672 | 5.0839608 | 12.767217 |
| TCGA-AB-2841-03B-01T-0760-13 | Low risk | 4.1888698 | 2.3422677 | 9.2741648 | 4.3057094 | 4.1653179 | 6.5593101 | 26.764076 |
| TCGA-AB-2842-03A-01T-0734-13 | High risk | 3.2132977 | 2.1137974 | 3.6299999 | 3.4869481 | 3.475416 | 6.1034552 | 10.976898 |
| TCGA-AB-2843-03A-01T-0736-13 | High risk | 0.908209 | 1.2457473 | 1.0349657 | 1.6939564 | 1.9070605 | 2.9803916 | 4.39878 |
| TCGA-AB-2844-03A-01T-0736-13 | Low risk | 1.062422 | 1.5456591 | 0.8036048 | 1.5362841 | 1.9936942 | 3.0170843 | 4.5517141 |
| TCGA-AB-2845-03B-01T-0748-13 | High risk | 0.8524142 | 1.5822167 | 0.6837147 | 1.7588191 | 2.0520268 | 3.4556498 | 3.8795309 |
| TCGA-AB-2846-03A-01T-0736-13 | High risk | 1.2005719 | 1.4566422 | 1.3115208 | 2.5444434 | 2.2530525 | 3.2338814 | 4.6051206 |
| TCGA-AB-2847-03A-01T-0736-13 | High risk | 1.3951886 | 1.3037545 | 1.3974954 | 1.6784534 | 1.8565408 | 2.3922669 | 5.3717114 |
| TCGA-AB-2849-03A-01T-0734-13 | Low risk | 2.4975455 | 3.0394776 | 7.7945547 | 1.9593381 | 2.9033726 | 6.0445747 | 10.775766 |
| TCGA-AB-2851-03A-01T-0736-13 | High risk | 1.8017816 | 1.3696856 | 1.2738847 | 2.7803403 | 2.5038249 | 2.8693326 | 8.5741149 |
| TCGA-AB-2853-03A-01T-0734-13 | Low risk | 1.3089776 | 1.8245264 | 1.3221347 | 2.7449585 | 2.2827708 | 4.8004478 | 5.3858028 |
| TCGA-AB-2856-03A-01T-0736-13 | High risk | 1.4317235 | 1.092366 | 0.7646823 | 2.3575431 | 1.9829839 | 3.093219 | 6.5509654 |
| TCGA-AB-2857-03A-01T-0736-13 | High risk | 0.9880192 | 1.4434367 | 1.9672133 | 0.9001123 | 1.9815885 | 4.2826463 | 5.0514875 |
| TCGA-AB-2858-03A-01T-0736-13 | Low risk | 2.1886455 | 1.9304003 | 1.9157615 | 2.4138821 | 2.7989777 | 5.4186332 | 8.0766899 |
| TCGA-AB-2859-03A-01T-0736-13 | High risk | 1.1291755 | 1.2852941 | 1.3852809 | 2.268754 | 1.934012 | 2.7210074 | 5.5641798 |
| TCGA-AB-2861-03A-01T-0736-13 | Low risk | 1.633345 | 1.1248133 | 0.4940052 | 1.4892223 | 2.0804822 | 2.5040036 | 4.4764835 |
| TCGA-AB-2862-03A-01T-0736-13 | Low risk | 1.9636124 | 1.4856245 | 2.8013581 | 2.0264017 | 2.4055188 | 3.4739908 | 8.4174535 |
| TCGA-AB-2863-03A-01T-0734-13 | High risk | 1.1644709 | 1.9067844 | 0.8236073 | 2.0620115 | 2.2679659 | 4.9458383 | 4.0291534 |
| TCGA-AB-2865-03A-01T-0736-13 | High risk | 1.3580226 | 1.6138276 | 2.0256185 | 3.1944512 | 2.1353103 | 3.4092966 | 6.3715405 |
| TCGA-AB-2866-03A-01T-0736-13 | High risk | 1.3883873 | 1.4521774 | 0.7456352 | 2.7358875 | 2.5976214 | 3.1675754 | 6.4116457 |
| TCGA-AB-2867-03A-01T-0734-13 | Low risk | 1.6559463 | 2.308546 | 3.6090813 | 3.0447372 | 3.4929068 | 8.2985829 | 5.9968303 |
| TCGA-AB-2869-03A-01T-0735-13 | Low risk | 1.1264697 | 1.4553552 | 1.3339444 | 1.4546566 | 1.9106734 | 2.8394375 | 4.8551839 |
| TCGA-AB-2870-03A-01T-0735-13 | High risk | 0.8718783 | 1.5387186 | 1.2398469 | 2.5594058 | 2.0990834 | 3.4405811 | 4.1439162 |
| TCGA-AB-2871-03A-01T-0735-13 | Low risk | 1.0260847 | 1.2607678 | 1.1296413 | 1.8189734 | 1.713894 | 2.6707803 | 4.8285621 |
| TCGA-AB-2872-03A-01T-0735-13 | Low risk | 3.1685965 | 2.1137531 | 12.203757 | 3.4749826 | 3.2300659 | 3.5560855 | 19.862769 |
| TCGA-AB-2873-03A-01T-0735-13 | High risk | 2.9560889 | 1.6071838 | 2.7567676 | 2.1507163 | 3.1161112 | 3.8163904 | 10.403853 |
| TCGA-AB-2874-03A-01T-0735-13 | Low risk | 1.2291511 | 1.7782773 | 1.7608251 | 2.1331269 | 2.1074132 | 4.0087196 | 5.3626449 |
| TCGA-AB-2875-03A-01T-0735-13 | Low risk | 1.1594604 | 1.8186448 | 1.5354637 | 2.5180141 | 2.2050252 | 3.906643 | 4.7016868 |
| TCGA-AB-2876-03A-01T-0734-13 | Low risk | 2.1151902 | 2.2221133 | 1.828969 | 3.1250379 | 3.2473823 | 7.1473208 | 6.8909033 |
| TCGA-AB-2877-03A-01T-0735-13 | Low risk | 1.0875711 | 1.7664637 | 1.1335475 | 1.228515 | 2.3285354 | 3.7069312 | 4.6216738 |
| TCGA-AB-2878-03A-01T-0734-13 | Low risk | 4.0689017 | 2.8034492 | 6.2531105 | 2.3083021 | 3.9763469 | 9.6250119 | 14.151171 |
| TCGA-AB-2880-03A-01T-0735-13 | Low risk | 0.7514077 | 1.6620244 | 1.0403678 | 2.233153 | 2.0957512 | 4.386684 | 3.7927094 |
| TCGA-AB-2881-03A-01T-0735-13 | High risk | 1.0576925 | 1.389093 | 1.128938 | 2.9678487 | 2.224391 | 3.7049267 | 4.5522271 |
| TCGA-AB-2882-03A-01T-0740-13 | Low risk | 1.9269788 | 1.7236544 | 6.4337144 | 2.0213266 | 2.3246011 | 3.2149627 | 11.08075 |
| TCGA-AB-2883-03A-01T-0734-13 | Low risk | 5.644151 | 1.1124327 | 7.0705078 | 3.8151056 | 3.3601973 | 2.1983902 | 17.775913 |
| TCGA-AB-2884-03A-01T-0735-13 | High risk | 1.1619756 | 1.3779622 | 0.9095994 | 1.9635401 | 2.2197104 | 2.904216 | 4.6955602 |
| TCGA-AB-2885-03A-01T-0735-13 | High risk | 1.1963619 | 1.782438 | 1.9345006 | 2.2368163 | 2.0856858 | 3.486162 | 8.1677788 |
| TCGA-AB-2886-03A-01T-0735-13 | Low risk | 1.0633437 | 1.961025 | 1.0943576 | 2.1281922 | 2.3756969 | 3.7481453 | 4.2819689 |
| TCGA-AB-2888-03B-01T-0748-13 | High risk | 1.6510183 | 1.2716218 | 0.8097602 | 2.0553087 | 2.5428307 | 3.8264986 | 4.8943286 |
| TCGA-AB-2889-03A-01T-0735-13 | Low risk | 1.4457065 | 1.5755036 | 1.5586306 | 3.0047497 | 2.3635434 | 3.6125484 | 6.3175355 |
| TCGA-AB-2890-03A-01T-0735-13 | High risk | 0.9606696 | 1.5350087 | 2.4881474 | 2.7079405 | 2.4456634 | 4.1949254 | 4.9546307 |
| TCGA-AB-2891-03A-01T-0735-13 | High risk | 1.1563497 | 1.1991496 | 0.805588 | 1.8404968 | 1.6638459 | 2.6474102 | 5.4057085 |
| TCGA-AB-2892-03A-01T-0734-13 | Low risk | 3.7957735 | 1.9445351 | 5.0392756 | 4.3251477 | 4.0042999 | 6.7780059 | 12.262523 |
| TCGA-AB-2893-03A-01T-0734-13 | High risk | 2.463271 | 1.3497762 | 0.7178216 | 2.8218049 | 2.8616653 | 4.4127684 | 8.3460398 |
| TCGA-AB-2894-03A-01T-0734-13 | Low risk | 2.1296805 | 1.3255544 | 1.7762983 | 2.052182 | 2.4681378 | 2.9288895 | 5.8704503 |
| TCGA-AB-2895-03A-01T-0735-13 | High risk | 1.0528296 | 1.5462619 | 1.7755787 | 2.3871311 | 1.8386767 | 2.4755784 | 5.4450079 |
| TCGA-AB-2896-03B-01T-0751-13 | High risk | 0.9423711 | 1.4354148 | 0.7698821 | 2.3557229 | 1.9600234 | 3.3194453 | 4.5915362 |
| TCGA-AB-2897-03A-01T-0735-13 | High risk | 3.1284743 | 2.1497238 | 4.3788772 | 2.7110089 | 3.0047875 | 6.0228705 | 16.65719 |
| TCGA-AB-2898-03A-01T-0735-13 | High risk | 2.0216698 | 1.8799054 | 2.1818672 | 2.2835626 | 2.5413323 | 3.2110424 | 10.256522 |
| TCGA-AB-2899-03A-01T-0736-13 | Low risk | 1.4290642 | 1.3212244 | 2.0843583 | 2.0268982 | 2.4449771 | 1.8667163 | 5.1168492 |
| TCGA-AB-2900-03A-01T-0735-13 | High risk | 1.4325486 | 1.786905 | 1.1085881 | 2.9343188 | 2.786379 | 4.5842412 | 6.9670191 |
| TCGA-AB-2901-03A-01T-0735-13 | High risk | 0.9538356 | 1.998652 | 1.4882014 | 3.9769182 | 2.4427844 | 5.1990521 | 5.8720848 |
| TCGA-AB-2908-03A-01T-0740-13 | High risk | 3.9990524 | 1.9587431 | 4.8500528 | 2.7454032 | 2.8153133 | 6.4506471 | 17.4864 |
| TCGA-AB-2910-03A-01T-0740-13 | High risk | 1.7557408 | 1.2768846 | 0.8370625 | 1.0547431 | 2.005429 | 2.3042105 | 4.2678211 |
| TCGA-AB-2911-03A-01T-0734-13 | Low risk | 4.024402 | 2.1023545 | 10.310185 | 3.9424753 | 4.7358483 | 5.8754996 | 18.286126 |
| TCGA-AB-2912-03A-01T-0734-13 | High risk | 2.3372359 | 1.865952 | 2.8517599 | 3.2775029 | 3.3016274 | 5.516514 | 6.5978548 |
| TCGA-AB-2913-03A-01T-0734-13 | Low risk | 4.7298973 | 2.2975751 | 4.6712764 | 3.5348129 | 3.6643624 | 6.0713487 | 16.489027 |
| TCGA-AB-2914-03A-01T-0734-13 | Low risk | 1.8551158 | 2.2469806 | 3.8403247 | 4.025739 | 3.3245517 | 7.3878708 | 6.4232165 |
| TCGA-AB-2915-03A-01T-0740-13 | High risk | 1.0030634 | 1.2738028 | 0.8008125 | 1.9856217 | 1.7549807 | 3.1294658 | 5.0609958 |
| TCGA-AB-2916-03A-01T-0734-13 | Low risk | 5.8707617 | 1.8256121 | 4.8311692 | 3.7044218 | 4.2273103 | 6.6613 | 16.247974 |
| TCGA-AB-2917-03A-01T-0734-13 | Low risk | 1.6966858 | 2.6907452 | 5.2604175 | 2.110676 | 3.2994291 | 7.5914949 | 6.8872975 |
| TCGA-AB-2918-03A-01T-0740-13 | High risk | 1.1385339 | 1.5637759 | 0.7400521 | 2.3091704 | 2.0652775 | 3.5179879 | 5.049292 |
| TCGA-AB-2919-03A-01T-0740-13 | Low risk | 0.6772255 | 1.4846635 | 0.8857338 | 1.2338132 | 1.7763856 | 2.1428196 | 3.4646558 |
| TCGA-AB-2920-03B-01T-0760-13 | High risk | 2.0347532 | 1.7514239 | 3.690289 | 2.9370552 | 2.6832085 | 4.0534377 | 10.795925 |
| TCGA-AB-2921-03A-01T-0740-13 | High risk | 1.174396 | 1.199485 | 0.5684022 | 2.1303026 | 1.8319592 | 3.1759083 | 4.7185116 |
| TCGA-AB-2924-03A-01T-0740-13 | High risk | 1.2045396 | 1.4288518 | 0.8654284 | 2.1000378 | 2.1201784 | 3.3094123 | 4.8804402 |
| TCGA-AB-2925-03A-01T-0735-13 | High risk | 2.2537512 | 1.3451823 | 0.8518653 | 1.9830322 | 2.547678 | 2.8642084 | 7.7806576 |
| TCGA-AB-2927-03A-01T-0740-13 | High risk | 2.9233159 | 1.4321 | 2.1400863 | 2.7243253 | 3.0016216 | 3.6989851 | 8.8191223 |
| TCGA-AB-2928-03A-01T-0740-13 | High risk | 0.7292723 | 1.4139581 | 0.9127498 | 2.4529438 | 1.5879761 | 2.4822549 | 3.7769796 |
| TCGA-AB-2929-03A-01T-0735-13 | High risk | 0.7987748 | 1.1946764 | 1.3688102 | 1.6052452 | 1.7734338 | 2.7484475 | 5.2466762 |
| TCGA-AB-2930-03A-01T-0740-13 | High risk | 0.7126157 | 1.4150846 | 0.5463657 | 1.6558381 | 1.7875981 | 2.6443295 | 3.0802042 |
| TCGA-AB-2931-03A-01T-0740-13 | High risk | 0.8279433 | 1.4171617 | 0.8839829 | 2.3569907 | 1.9317182 | 3.3982484 | 4.0026628 |
| TCGA-AB-2932-03A-01T-0740-13 | High risk | 1.4353492 | 1.3512167 | 0.8629084 | 2.4342 | 2.3901829 | 2.9559072 | 6.9120019 |
| TCGA-AB-2933-03A-01T-0734-13 | High risk | 2.9441006 | 1.9474435 | 5.4628118 | 3.7394847 | 3.6824012 | 6.4836131 | 10.301867 |
| TCGA-AB-2934-03A-01T-0740-13 | High risk | 1.863506 | 1.4292385 | 1.8448613 | 2.9098113 | 2.6749389 | 2.9815353 | 8.1257905 |
| TCGA-AB-2935-03A-01T-0740-13 | High risk | 1.0761815 | 0.9168672 | 1.5901093 | 1.1934461 | 1.7367744 | 2.0344889 | 4.6085632 |
| TCGA-AB-2936-03A-01T-0740-13 | Low risk | 1.3513956 | 2.0305545 | 2.179217 | 2.0267708 | 2.8242873 | 4.5255659 | 5.6271913 |
| TCGA-AB-2937-03A-01T-0734-13 | Low risk | 9.9503931 | 3.4383843 | 41.693049 | 9.107773 | 8.6568707 | 12.560296 | 74.151046 |
| TCGA-AB-2938-03A-01T-0736-13 | High risk | 1.6738511 | 1.4660708 | 3.9251615 | 1.3435038 | 2.0829082 | 4.6112952 | 12.200875 |
| TCGA-AB-2939-03A-01T-0740-13 | High risk | 1.4659713 | 1.2781334 | 1.2436793 | 2.132291 | 1.8356218 | 2.1590306 | 5.6583213 |
| TCGA-AB-2940-03A-01T-0736-13 | Low risk | 1.1829527 | 1.7154017 | 1.610341 | 1.7238077 | 2.1384076 | 4.4012407 | 6.2685609 |
| TCGA-AB-2941-03A-01T-0740-13 | High risk | 1.9238119 | 3.2677286 | 4.3995523 | 1.8940884 | 3.6343751 | 8.9250239 | 15.425902 |
| TCGA-AB-2942-03A-01T-0734-13 | Low risk | 1.5720448 | 1.6410821 | 1.6876737 | 4.310278 | 3.1046802 | 5.365234 | 6.185936 |
| TCGA-AB-2943-03A-01T-0740-13 | High risk | 3.4571985 | 2.1514149 | 2.8533811 | 1.8870106 | 2.8402994 | 6.5092923 | 10.172473 |
| TCGA-AB-2944-03A-01T-0740-13 | High risk | 0.9941908 | 1.4080113 | 1.4770882 | 2.1899111 | 1.7550464 | 3.3520987 | 5.0660438 |
| TCGA-AB-2946-03A-01T-0740-13 | Low risk | 1.022529 | 1.6748606 | 0.8852761 | 2.0218992 | 2.183911 | 4.2450715 | 6.1257326 |
| TCGA-AB-2948-03A-01T-0740-13 | High risk | 1.255016 | 1.5056627 | 0.8985616 | 1.7638478 | 2.1938806 | 2.9141123 | 5.5736823 |
| TCGA-AB-2949-03B-01T-0748-13 | High risk | 0.8786608 | 1.5898542 | 0.8043126 | 1.870412 | 1.9124649 | 3.6968769 | 3.6785961 |
| TCGA-AB-2950-03A-01T-0735-13 | Low risk | 1.7965851 | 2.1366052 | 3.8067591 | 2.6603625 | 3.0744762 | 5.1531163 | 6.4684676 |
| TCGA-AB-2952-03B-01T-0760-13 | High risk | 1.9495447 | 1.9469384 | 2.5462133 | 1.7234017 | 2.9382292 | 4.2450739 | 10.717609 |
| TCGA-AB-2955-03A-01T-0734-13 | Low risk | 1.8989776 | 1.98968 | 6.2116601 | 3.8690618 | 2.8044088 | 6.0654246 | 6.6195952 |
| TCGA-AB-2956-03A-01T-0740-13 | High risk | 1.2394535 | 1.141694 | 0.6701505 | 1.7142374 | 1.7841112 | 2.1336448 | 5.1165801 |
| TCGA-AB-2959-03A-01T-0734-13 | Low risk | 1.8837746 | 2.6992468 | 4.7780831 | 3.3485468 | 3.743084 | 7.2050265 | 5.9026268 |
| TCGA-AB-2963-03A-01T-0734-13 | Low risk | 1.4299655 | 1.8742753 | 1.0664687 | 1.9490268 | 2.2778065 | 4.977918 | 5.4542431 |
| TCGA-AB-2965-03A-01T-0734-13 | Low risk | 2.5990219 | 1.7921868 | 1.8351012 | 2.6659393 | 3.1424165 | 5.5225216 | 7.4075491 |
| TCGA-AB-2966-03A-01T-0734-13 | Low risk | 1.8641453 | 2.1467662 | 4.0362348 | 4.1713442 | 3.0996363 | 6.6550839 | 7.3183875 |
| TCGA-AB-2970-03A-01T-0734-13 | Low risk | 1.7772938 | 1.6012435 | 2.9947157 | 2.9865335 | 2.8774426 | 3.5687123 | 5.8375329 |
| TCGA-AB-2971-03A-01T-0734-13 | Low risk | 1.9423018 | 1.5925 | 3.0330432 | 2.5288053 | 3.0703026 | 3.5109675 | 6.4570477 |
| TCGA-AB-2973-03A-01T-0734-13 | Low risk | 6.8916741 | 1.7805329 | 7.9997912 | 4.3076848 | 5.7714684 | 6.0619768 | 19.446019 |
| TCGA-AB-2975-03A-01T-0734-13 | Low risk | 1.9184765 | 2.000104 | 1.8175472 | 2.7219242 | 2.8854244 | 5.5029332 | 8.5925212 |
| TCGA-AB-2976-03A-01T-0734-13 | Low risk | 2.4616016 | 2.0860843 | 3.8150093 | 2.8179303 | 2.8997236 | 5.2706737 | 10.784836 |
| TCGA-AB-2977-03B-01T-0760-13 | Low risk | 2.825184 | 1.1320976 | 1.6108858 | 4.5963005 | 3.333652 | 3.6436846 | 18.431433 |
| TCGA-AB-2979-03B-01T-0760-13 | Low risk | 0.9904772 | 1.7070732 | 1.1146712 | 2.140825 | 2.1626589 | 3.7953809 | 5.7919573 |
| TCGA-AB-2980-03A-01T-0734-13 | Low risk | 2.4063343 | 2.0623538 | 4.5414721 | 2.3355046 | 2.7467702 | 7.5554702 | 8.8057673 |
| TCGA-AB-2981-03B-01T-0748-13 | High risk | 1.8045131 | 1.3256862 | 0.6889876 | 2.3535383 | 2.853921 | 3.7603669 | 6.5798944 |
| TCGA-AB-2982-03B-01T-0748-13 | Low risk | 3.0200941 | 1.5626719 | 4.2659395 | 2.0759683 | 2.5588077 | 4.3890397 | 11.761413 |
| TCGA-AB-2983-03A-01T-0734-13 | High risk | 2.2937564 | 1.6491725 | 2.0068152 | 2.950021 | 2.6540681 | 3.5029654 | 7.9992123 |
| TCGA-AB-2984-03A-01T-0734-13 | Low risk | 6.5160274 | 2.5494872 | 9.4010607 | 6.9022922 | 9.0174828 | 12.276038 | 52.928081 |
| TCGA-AB-2986-03A-01T-0734-13 | Low risk | 2.9104249 | 1.5103268 | 2.5431671 | 3.4985972 | 3.0811037 | 6.1972068 | 12.475676 |
| TCGA-AB-2987-03A-01T-0734-13 | High risk | 6.0798798 | 1.1593585 | 2.5610654 | 2.9500264 | 3.9612454 | 4.9950821 | 14.994458 |
| TCGA-AB-2988-03B-01T-0748-13 | High risk | 0.7932179 | 1.4262361 | 0.7597314 | 1.9727326 | 1.7891719 | 2.7250713 | 3.4143831 |
| TCGA-AB-2990-03B-01T-0748-13 | Low risk | 1.7330671 | 1.7138389 | 1.5910177 | 2.0708034 | 2.8517614 | 4.3386202 | 7.3614886 |
| TCGA-AB-2991-03A-01T-0735-13 | High risk | 2.0563293 | 1.9525373 | 3.678613 | 2.2620141 | 2.5446739 | 4.7135711 | 9.0679219 |
| TCGA-AB-2992-03A-01T-0735-13 | Low risk | 2.7278964 | 1.9308116 | 4.7978477 | 2.5174266 | 2.9540695 | 3.5346814 | 12.32188 |
| TCGA-AB-2994-03A-01T-0735-13 | Low risk | 1.2880983 | 1.7560615 | 2.4656999 | 2.4562692 | 2.7138614 | 4.0725786 | 7.0254101 |
| TCGA-AB-2995-03A-01T-0735-13 | High risk | 1.4365575 | 1.6203863 | 2.3788459 | 1.7925549 | 2.1123618 | 3.1728516 | 6.4774783 |
| TCGA-AB-2996-03A-01T-0735-13 | Low risk | 1.6411882 | 1.6286983 | 2.8661998 | 2.334688 | 2.0973702 | 2.6091952 | 8.1161588 |
| TCGA-AB-2998-03A-01T-0735-13 | High risk | 1.9443424 | 2.0917464 | 1.917863 | 2.355225 | 2.5815312 | 5.027482 | 9.1843606 |
| TCGA-AB-2999-03B-01T-0748-13 | Low risk | 3.5534094 | 1.8647994 | 3.3726593 | 2.2939865 | 3.0644626 | 5.3275386 | 13.156569 |
| TCGA-AB-3000-03A-01T-0736-13 | Low risk | 1.037182 | 1.6915893 | 0.8023329 | 1.8840115 | 2.1127813 | 3.2112792 | 5.120005 |
| TCGA-AB-3001-03A-01T-0736-13 | Low risk | 2.6120544 | 1.8862512 | 3.8881971 | 2.0691403 | 2.4405946 | 3.9380672 | 11.491893 |
| TCGA-AB-3002-03A-01T-0736-13 | High risk | 1.1713534 | 1.7810523 | 2.0198937 | 2.7959267 | 2.6629566 | 4.4506535 | 4.7564447 |
| TCGA-AB-3007-03A-01T-0736-13 | High risk | 2.9118374 | 1.3689832 | 2.2123485 | 1.7881205 | 2.0969103 | 3.0159836 | 10.456837 |
| TCGA-AB-3008-03A-01T-0736-13 | Low risk | 0.8856763 | 1.9909459 | 1.282871 | 1.7853539 | 2.0332999 | 4.2461155 | 4.2908114 |
| TCGA-AB-3009-03A-01T-0736-13 | High risk | 0.991304 | 1.7167566 | 1.6227189 | 2.7549437 | 1.8712148 | 3.209385 | 4.1111474 |
| TCGA-AB-3011-03A-01T-0736-13 | Low risk | 0.9943047 | 1.798941 | 1.6225198 | 1.4592326 | 2.2498328 | 3.023725 | 4.5562867 |
| TCGA-AB-3012-03A-01T-0736-13 | Low risk | 2.8863863 | 1.7858244 | 3.8728762 | 2.5347706 | 2.5082978 | 4.1809333 | 13.403 |
| Median IC 50 | NA | 1.6559463 | 1.6767336 | 1.8175472 | 2.3535383 | 2.5413323 | 3.9380672 | 6.4774783 |

**Supplementary Table 8 (Continue)**

| **TCGA ID** | Group | AZD6738_1917 | Taselisib_1561 | BMS-536924_1091 | Ulixertinib_2047 | YK-4-279_1239 | BDP-00009066_1866 |
| --- | --- | --- | --- | --- | --- | --- | --- |
| TCGA-AB-2805-03A-01T-0734-13 | Low risk | 7.6706175 | 6.0726261 | 9.1815512 | 8.7553354 | 8.7385935 | 9.0521664 |
| TCGA-AB-2806-03A-01T-0734-13 | Low risk | 8.6591645 | 6.7477303 | 9.0306921 | 4.1907908 | 8.5522527 | 8.1925435 |
| TCGA-AB-2808-03A-01T-0734-13 | Low risk | 5.6677115 | 12.661708 | 9.2979425 | 7.0488994 | 6.2215546 | 8.7689772 |
| TCGA-AB-2810-03A-01T-0736-13 | High risk | 7.5159067 | 19.899881 | 11.479158 | 11.095728 | 16.26252 | 18.058138 |
| TCGA-AB-2811-03B-01T-0760-13 | High risk | 6.691024 | 11.570992 | 14.856057 | 10.792318 | 16.55332 | 14.345303 |
| TCGA-AB-2812-03A-01T-0734-13 | Low risk | 10.917291 | 14.852639 | 12.259045 | 8.4319307 | 18.641898 | 11.929944 |
| TCGA-AB-2813-03A-01T-0736-13 | High risk | 10.5664 | 5.9798064 | 8.6677694 | 20.893974 | 14.039388 | 15.091358 |
| TCGA-AB-2814-03A-01T-0734-13 | High risk | 8.8289583 | 9.1348765 | 8.2766598 | 7.1255628 | 10.261196 | 10.102959 |
| TCGA-AB-2815-03A-01T-0734-13 | Low risk | 8.0611044 | 6.7953087 | 10.19895 | 16.92334 | 15.669498 | 14.17932 |
| TCGA-AB-2817-03A-01T-0736-13 | High risk | 3.7850707 | 4.3536581 | 5.9066078 | 7.1282244 | 6.4318193 | 7.0945298 |
| TCGA-AB-2818-03A-01T-0734-13 | Low risk | 8.8848962 | 4.5208071 | 9.0306238 | 6.3802712 | 6.6207081 | 8.020314 |
| TCGA-AB-2819-03A-01T-0734-13 | Low risk | 5.5106834 | 9.5144714 | 9.201303 | 7.3802291 | 9.4639574 | 8.7954569 |
| TCGA-AB-2820-03A-01T-0735-13 | High risk | 13.334359 | 12.444251 | 6.3081448 | 8.2906299 | 9.9406404 | 9.2748311 |
| TCGA-AB-2821-03A-01T-0735-13 | Low risk | 6.1602257 | 4.2963728 | 6.0470204 | 9.1207361 | 7.5425949 | 8.9274049 |
| TCGA-AB-2822-03A-01T-0734-13 | Low risk | 11.499199 | 16.090449 | 10.90588 | 7.421184 | 10.722308 | 8.9231432 |
| TCGA-AB-2823-03A-01T-0736-13 | High risk | 11.11238 | 10.442364 | 6.0872032 | 7.0065093 | 9.8145563 | 9.2453715 |
| TCGA-AB-2825-03A-01T-0736-13 | High risk | 6.6569406 | 3.1902608 | 5.2237403 | 6.9586013 | 6.3777561 | 8.9458051 |
| TCGA-AB-2826-03A-01T-0734-13 | Low risk | 9.5186731 | 10.20316 | 12.111935 | 18.012655 | 21.636971 | 15.947758 |
| TCGA-AB-2828-03A-01T-0734-13 | Low risk | 6.2559887 | 5.0796176 | 6.7706811 | 8.7756499 | 7.8637765 | 9.6217357 |
| TCGA-AB-2830-03A-01T-0736-13 | High risk | 8.4000309 | 5.6489471 | 5.2355116 | 8.0976144 | 8.0472 | 10.886135 |
| TCGA-AB-2834-03A-01T-0734-13 | Low risk | 6.7932271 | 6.3946304 | 10.208719 | 15.060753 | 12.415136 | 14.797579 |
| TCGA-AB-2835-03A-01T-0736-13 | High risk | 5.4708891 | 6.2711925 | 6.4083703 | 8.541341 | 6.1736777 | 10.594604 |
| TCGA-AB-2836-03A-01T-0736-13 | High risk | 5.4127126 | 3.465498 | 7.8414513 | 7.3334179 | 5.1564395 | 8.1395462 |
| TCGA-AB-2839-03A-01T-0734-13 | Low risk | 11.42667 | 17.740737 | 10.787276 | 8.0124877 | 10.99981 | 10.651736 |
| TCGA-AB-2840-03A-01T-0734-13 | Low risk | 9.5186876 | 10.820624 | 11.525187 | 19.433084 | 25.075036 | 16.211753 |
| TCGA-AB-2841-03B-01T-0760-13 | Low risk | 17.245912 | 18.017078 | 10.434377 | 19.373682 | 31.3979 | 21.749788 |
| TCGA-AB-2842-03A-01T-0734-13 | High risk | 13.822926 | 9.2312413 | 12.366738 | 17.164722 | 29.110794 | 16.7752 |
| TCGA-AB-2843-03A-01T-0736-13 | High risk | 3.1804303 | 5.7373085 | 4.6192096 | 9.144568 | 4.5628913 | 9.8005965 |
| TCGA-AB-2844-03A-01T-0736-13 | Low risk | 4.7121192 | 5.1413634 | 5.8448183 | 4.9965974 | 4.1124742 | 7.3874968 |
| TCGA-AB-2845-03B-01T-0748-13 | High risk | 4.563243 | 6.3684355 | 5.2226227 | 4.9242893 | 3.2994972 | 7.9110853 |
| TCGA-AB-2846-03A-01T-0736-13 | High risk | 5.4888918 | 4.5912011 | 6.4902532 | 7.2766119 | 5.3332372 | 8.6887943 |
| TCGA-AB-2847-03A-01T-0736-13 | High risk | 5.133259 | 5.1473859 | 6.2641197 | 11.032567 | 6.6090645 | 9.1907012 |
| TCGA-AB-2849-03A-01T-0734-13 | Low risk | 10.78703 | 8.4080248 | 19.383425 | 15.232988 | 18.593927 | 14.745275 |
| TCGA-AB-2851-03A-01T-0736-13 | High risk | 10.065869 | 4.5935288 | 7.1761608 | 8.0776387 | 8.1931369 | 9.4685741 |
| TCGA-AB-2853-03A-01T-0734-13 | Low risk | 9.6133126 | 11.595308 | 9.1999106 | 5.9606816 | 7.7376741 | 7.7277197 |
| TCGA-AB-2856-03A-01T-0736-13 | High risk | 5.1075682 | 9.0729141 | 4.4756141 | 7.1456965 | 5.1050871 | 9.4579656 |
| TCGA-AB-2857-03A-01T-0736-13 | High risk | 3.2327971 | 6.4109264 | 8.9864047 | 7.543526 | 5.2410103 | 9.1901157 |
| TCGA-AB-2858-03A-01T-0736-13 | Low risk | 9.4657646 | 13.068562 | 8.7722229 | 5.6006546 | 9.7283426 | 9.9862409 |
| TCGA-AB-2859-03A-01T-0736-13 | High risk | 9.8509494 | 6.1399523 | 6.9046055 | 5.3124471 | 7.1678658 | 6.9874615 |
| TCGA-AB-2861-03A-01T-0736-13 | Low risk | 3.656475 | 6.1141245 | 4.2868468 | 4.4379872 | 8.0988275 | 8.0493929 |
| TCGA-AB-2862-03A-01T-0736-13 | Low risk | 8.4193644 | 10.023996 | 7.1596396 | 9.6888659 | 10.674621 | 12.187023 |
| TCGA-AB-2863-03A-01T-0734-13 | High risk | 4.3245485 | 12.328076 | 7.6269649 | 6.9341325 | 8.5990474 | 6.5961398 |
| TCGA-AB-2865-03A-01T-0736-13 | High risk | 8.1475173 | 6.5919716 | 8.9613221 | 10.332268 | 6.5801792 | 7.9287822 |
| TCGA-AB-2866-03A-01T-0736-13 | High risk | 6.1329055 | 4.8448025 | 5.861358 | 8.424035 | 6.5581201 | 9.1301912 |
| TCGA-AB-2867-03A-01T-0734-13 | Low risk | 9.5133615 | 16.814619 | 10.937474 | 11.006603 | 10.984262 | 11.822696 |
| TCGA-AB-2869-03A-01T-0735-13 | Low risk | 5.7602985 | 6.0564345 | 6.027477 | 5.4732284 | 4.8926814 | 8.2846557 |
| TCGA-AB-2870-03A-01T-0735-13 | High risk | 4.4837666 | 4.3338886 | 5.3037386 | 8.5798032 | 4.5141258 | 8.8359744 |
| TCGA-AB-2871-03A-01T-0735-13 | Low risk | 6.446238 | 5.8648438 | 5.2206218 | 4.2783218 | 4.7420811 | 6.4663312 |
| TCGA-AB-2872-03A-01T-0735-13 | Low risk | 14.961628 | 5.5624554 | 8.973851 | 14.593307 | 20.750387 | 17.782091 |
| TCGA-AB-2873-03A-01T-0735-13 | High risk | 8.3936591 | 4.4250655 | 10.146776 | 12.678093 | 14.56756 | 15.064347 |
| TCGA-AB-2874-03A-01T-0735-13 | Low risk | 8.0698042 | 4.6688825 | 4.4389348 | 5.6572905 | 6.2860188 | 6.8943018 |
| TCGA-AB-2875-03A-01T-0735-13 | Low risk | 6.7177134 | 6.4862673 | 5.2118057 | 4.3587814 | 4.7119022 | 7.6956493 |
| TCGA-AB-2876-03A-01T-0734-13 | Low risk | 11.330609 | 16.377997 | 9.539123 | 7.4310625 | 8.6255895 | 9.4495394 |
| TCGA-AB-2877-03A-01T-0735-13 | Low risk | 6.1116879 | 8.3555515 | 8.8474386 | 5.4584779 | 6.4795759 | 11.32493 |
| TCGA-AB-2878-03A-01T-0734-13 | Low risk | 12.972334 | 24.199151 | 14.484811 | 18.613096 | 34.975798 | 11.867553 |
| TCGA-AB-2880-03A-01T-0735-13 | Low risk | 4.5317311 | 9.6594959 | 5.3394466 | 5.2184258 | 2.905121 | 7.7034713 |
| TCGA-AB-2881-03A-01T-0735-13 | High risk | 5.6788455 | 8.1178811 | 7.354539 | 7.2051014 | 5.2299615 | 7.5317476 |
| TCGA-AB-2882-03A-01T-0740-13 | Low risk | 11.97118 | 4.7488016 | 6.5533575 | 9.1891283 | 10.771196 | 10.37423 |
| TCGA-AB-2883-03A-01T-0734-13 | Low risk | 10.426651 | 4.4554421 | 9.5414492 | 20.324968 | 68.855889 | 24.15452 |
| TCGA-AB-2884-03A-01T-0735-13 | High risk | 6.0224212 | 5.6893923 | 6.3266151 | 5.5612119 | 5.4539547 | 8.4479506 |
| TCGA-AB-2885-03A-01T-0735-13 | High risk | 10.048022 | 4.9546139 | 5.1983238 | 8.6394326 | 4.9920419 | 7.5462142 |
| TCGA-AB-2886-03A-01T-0735-13 | Low risk | 5.7147431 | 6.4242294 | 7.1785592 | 3.7335591 | 4.9142629 | 7.8279053 |
| TCGA-AB-2888-03B-01T-0748-13 | High risk | 6.675891 | 5.9163002 | 8.3921941 | 7.9832138 | 5.1212195 | 8.1918902 |
| TCGA-AB-2889-03A-01T-0735-13 | Low risk | 7.0180641 | 6.2730865 | 5.9580718 | 7.5235302 | 6.5769752 | 9.3057212 |
| TCGA-AB-2890-03A-01T-0735-13 | High risk | 6.2349025 | 7.8381733 | 6.9359008 | 10.059446 | 5.5070877 | 9.9879907 |
| TCGA-AB-2891-03A-01T-0735-13 | High risk | 5.1638828 | 3.5335838 | 5.812893 | 7.5783641 | 3.1122917 | 9.958492 |
| TCGA-AB-2892-03A-01T-0734-13 | Low risk | 13.005764 | 13.0455 | 11.118562 | 18.107721 | 25.317207 | 17.205229 |
| TCGA-AB-2893-03A-01T-0734-13 | High risk | 5.4267658 | 6.2708727 | 6.9826 | 14.082734 | 13.648039 | 13.765282 |
| TCGA-AB-2894-03A-01T-0734-13 | Low risk | 7.2520974 | 4.8380738 | 5.9218286 | 6.8669116 | 15.286661 | 8.2164282 |
| TCGA-AB-2895-03A-01T-0735-13 | High risk | 9.6886147 | 5.1496859 | 6.6924505 | 4.311555 | 5.2441723 | 6.7580723 |
| TCGA-AB-2896-03B-01T-0751-13 | High risk | 5.6987409 | 9.7078406 | 5.7474234 | 5.4900362 | 4.2921416 | 6.4517041 |
| TCGA-AB-2897-03A-01T-0735-13 | High risk | 17.239187 | 15.655401 | 8.6532242 | 11.459628 | 16.905172 | 15.465012 |
| TCGA-AB-2898-03A-01T-0735-13 | High risk | 8.8689904 | 5.8789223 | 10.862011 | 10.081512 | 10.042841 | 11.13328 |
| TCGA-AB-2899-03A-01T-0736-13 | Low risk | 4.5744045 | 1.4546307 | 6.0295446 | 11.68374 | 9.9824886 | 11.065355 |
| TCGA-AB-2900-03A-01T-0735-13 | High risk | 5.9540029 | 8.8780728 | 8.5953381 | 8.8374934 | 8.9723972 | 10.648447 |
| TCGA-AB-2901-03A-01T-0735-13 | High risk | 4.6681604 | 13.886553 | 8.4849568 | 7.0831008 | 7.6858498 | 7.6217982 |
| TCGA-AB-2908-03A-01T-0740-13 | High risk | 9.4836864 | 19.612239 | 12.202593 | 22.337565 | 12.382742 | 19.204613 |
| TCGA-AB-2910-03A-01T-0740-13 | High risk | 4.7129549 | 3.3850604 | 6.0142066 | 6.9844295 | 7.1652012 | 12.253267 |
| TCGA-AB-2911-03A-01T-0734-13 | Low risk | 15.428797 | 11.372387 | 16.115068 | 24.278965 | 38.953288 | 21.23043 |
| TCGA-AB-2912-03A-01T-0734-13 | High risk | 5.8389379 | 7.9288708 | 11.182678 | 12.691293 | 15.954459 | 14.125233 |
| TCGA-AB-2913-03A-01T-0734-13 | Low risk | 17.407507 | 17.228601 | 14.876611 | 13.050849 | 33.875349 | 14.53956 |
| TCGA-AB-2914-03A-01T-0734-13 | Low risk | 9.5848783 | 12.842775 | 9.7986264 | 9.9324489 | 13.601549 | 10.621204 |
| TCGA-AB-2915-03A-01T-0740-13 | High risk | 4.4285659 | 5.7302385 | 6.9000477 | 6.538454 | 3.2696517 | 6.7798695 |
| TCGA-AB-2916-03A-01T-0734-13 | Low risk | 27.658665 | 10.979206 | 13.036561 | 14.632691 | 21.933307 | 15.317105 |
| TCGA-AB-2917-03A-01T-0734-13 | Low risk | 9.3512936 | 13.420706 | 23.659553 | 10.099571 | 21.614523 | 14.446342 |
| TCGA-AB-2918-03A-01T-0740-13 | High risk | 4.5356903 | 8.9139316 | 5.7893531 | 5.5824516 | 4.264648 | 7.906978 |
| TCGA-AB-2919-03A-01T-0740-13 | Low risk | 4.1810058 | 4.7782495 | 5.847465 | 4.3354631 | 4.3805649 | 7.4429411 |
| TCGA-AB-2920-03B-01T-0760-13 | High risk | 8.3648225 | 8.9864026 | 6.3022772 | 14.324457 | 12.190521 | 9.1983688 |
| TCGA-AB-2921-03A-01T-0740-13 | High risk | 4.8448708 | 9.6441716 | 6.0871579 | 4.6238023 | 4.199377 | 6.7500411 |
| TCGA-AB-2924-03A-01T-0740-13 | High risk | 6.2264102 | 5.649017 | 6.0620489 | 4.5746246 | 4.4329059 | 8.521358 |
| TCGA-AB-2925-03A-01T-0735-13 | High risk | 6.2928893 | 5.5849976 | 5.9205676 | 9.1649773 | 11.688551 | 14.321181 |
| TCGA-AB-2927-03A-01T-0740-13 | High risk | 6.1513718 | 8.0482412 | 11.283038 | 16.435473 | 18.020063 | 16.251272 |
| TCGA-AB-2928-03A-01T-0740-13 | High risk | 4.8328489 | 4.6170863 | 5.703836 | 4.276056 | 4.545241 | 4.7140827 |
| TCGA-AB-2929-03A-01T-0735-13 | High risk | 4.8009611 | 3.9904829 | 7.0231319 | 4.7736531 | 3.7044439 | 6.7372253 |
| TCGA-AB-2930-03A-01T-0740-13 | High risk | 3.238347 | 4.5956605 | 5.573832 | 4.4800685 | 2.7194206 | 7.4014969 |
| TCGA-AB-2931-03A-01T-0740-13 | High risk | 4.8302953 | 9.8615462 | 5.6556101 | 5.2950402 | 3.414776 | 6.529715 |
| TCGA-AB-2932-03A-01T-0740-13 | High risk | 5.9438798 | 4.7730676 | 6.2981572 | 8.6654384 | 7.1573121 | 10.026387 |
| TCGA-AB-2933-03A-01T-0734-13 | High risk | 10.120506 | 11.043051 | 15.22431 | 21.972407 | 27.773759 | 17.72449 |
| TCGA-AB-2934-03A-01T-0740-13 | High risk | 6.9363098 | 7.6152004 | 8.6715665 | 10.206678 | 10.589958 | 11.608012 |
| TCGA-AB-2935-03A-01T-0740-13 | High risk | 4.2757749 | 2.2773821 | 4.8990184 | 6.8187347 | 5.8231361 | 6.5796861 |
| TCGA-AB-2936-03A-01T-0740-13 | Low risk | 6.8056369 | 10.241458 | 10.804545 | 9.2712393 | 9.8007288 | 11.444853 |
| TCGA-AB-2937-03A-01T-0734-13 | Low risk | 31.986637 | 43.712225 | 19.757881 | 39.869305 | 178.33511 | 30.384951 |
| TCGA-AB-2938-03A-01T-0736-13 | High risk | 5.4660037 | 11.269673 | 12.883252 | 10.907646 | 9.8820559 | 10.703904 |
| TCGA-AB-2939-03A-01T-0740-13 | High risk | 5.5781061 | 1.9139632 | 4.8995437 | 5.3997627 | 5.4822383 | 6.7014695 |
| TCGA-AB-2940-03A-01T-0736-13 | Low risk | 5.8299391 | 9.2327164 | 7.5923427 | 8.9192955 | 5.1017032 | 10.014042 |
| TCGA-AB-2941-03A-01T-0740-13 | High risk | 16.660349 | 37.42009 | 15.022217 | 7.6234866 | 16.122508 | 12.901527 |
| TCGA-AB-2942-03A-01T-0734-13 | Low risk | 6.6481212 | 13.011392 | 9.7551005 | 10.034657 | 9.7611782 | 9.7804781 |
| TCGA-AB-2943-03A-01T-0740-13 | High risk | 14.886271 | 16.742157 | 10.173693 | 9.4562202 | 7.6185889 | 11.607002 |
| TCGA-AB-2944-03A-01T-0740-13 | High risk | 5.5198414 | 7.5809873 | 7.7511205 | 5.7495233 | 4.9635204 | 6.5933385 |
| TCGA-AB-2946-03A-01T-0740-13 | Low risk | 5.7913683 | 9.0867097 | 6.7430343 | 5.6691804 | 3.8466698 | 8.3659278 |
| TCGA-AB-2948-03A-01T-0740-13 | High risk | 4.9410815 | 4.2826517 | 7.7049253 | 10.024856 | 5.8679097 | 10.011286 |
| TCGA-AB-2949-03B-01T-0748-13 | High risk | 3.6161026 | 9.9864681 | 8.7433201 | 6.4463664 | 4.2508153 | 7.6591028 |
| TCGA-AB-2950-03A-01T-0735-13 | Low risk | 8.0989023 | 10.809132 | 10.937199 | 8.3110142 | 13.624695 | 12.10683 |
| TCGA-AB-2952-03B-01T-0760-13 | High risk | 7.7437296 | 6.7664697 | 6.6196206 | 13.000981 | 11.367706 | 11.87259 |
| TCGA-AB-2955-03A-01T-0734-13 | Low risk | 14.420419 | 15.083292 | 8.9118868 | 8.2360104 | 7.9054579 | 10.059812 |
| TCGA-AB-2956-03A-01T-0740-13 | High risk | 4.4006483 | 2.7773011 | 5.398284 | 5.300565 | 4.9053415 | 8.1972632 |
| TCGA-AB-2959-03A-01T-0734-13 | Low risk | 7.1197931 | 12.823837 | 12.71766 | 13.269736 | 16.987213 | 13.29611 |
| TCGA-AB-2963-03A-01T-0734-13 | Low risk | 7.9961448 | 9.3221187 | 8.4378513 | 6.9100983 | 8.1712201 | 8.9868441 |
| TCGA-AB-2965-03A-01T-0734-13 | Low risk | 8.9888252 | 9.9025281 | 10.299946 | 10.92254 | 15.206669 | 10.882623 |
| TCGA-AB-2966-03A-01T-0734-13 | Low risk | 10.692239 | 17.052919 | 10.259261 | 11.65359 | 15.243361 | 10.617236 |
| TCGA-AB-2970-03A-01T-0734-13 | Low risk | 6.0307709 | 5.4446693 | 8.8630538 | 11.15069 | 11.459496 | 12.441623 |
| TCGA-AB-2971-03A-01T-0734-13 | Low risk | 7.1336878 | 6.0010829 | 9.9351853 | 9.4024563 | 13.122038 | 12.033898 |
| TCGA-AB-2973-03A-01T-0734-13 | Low risk | 22.322071 | 10.793121 | 17.486375 | 21.523581 | 59.646605 | 20.467513 |
| TCGA-AB-2975-03A-01T-0734-13 | Low risk | 10.597516 | 19.065593 | 8.3264061 | 12.866332 | 10.272713 | 10.782128 |
| TCGA-AB-2976-03A-01T-0734-13 | Low risk | 12.706496 | 13.584899 | 11.471855 | 12.368276 | 16.046164 | 11.79047 |
| TCGA-AB-2977-03B-01T-0760-13 | Low risk | 10.961085 | 17.424816 | 11.346675 | 15.783631 | 20.32218 | 13.0291 |
| TCGA-AB-2979-03B-01T-0760-13 | Low risk | 4.3075227 | 9.5323219 | 4.3588351 | 7.1672011 | 3.7736999 | 10.899011 |
| TCGA-AB-2980-03A-01T-0734-13 | Low risk | 7.6268912 | 20.915558 | 12.034341 | 12.755857 | 13.830264 | 12.66732 |
| TCGA-AB-2981-03B-01T-0748-13 | High risk | 8.0536553 | 6.8070701 | 10.051916 | 10.43054 | 7.611067 | 9.5106106 |
| TCGA-AB-2982-03B-01T-0748-13 | Low risk | 11.924019 | 9.9667586 | 10.233979 | 11.937304 | 13.497352 | 12.62718 |
| TCGA-AB-2983-03A-01T-0734-13 | High risk | 8.3072075 | 5.9263795 | 8.7412795 | 11.490375 | 12.654946 | 12.785499 |
| TCGA-AB-2984-03A-01T-0734-13 | Low risk | 17.432193 | 50.318039 | 34.2441 | 39.833852 | 187.61035 | 20.72963 |
| TCGA-AB-2986-03A-01T-0734-13 | Low risk | 11.204593 | 31.614526 | 10.138979 | 12.900422 | 18.824835 | 11.673029 |
| TCGA-AB-2987-03A-01T-0734-13 | High risk | 15.49776 | 9.3475928 | 15.521698 | 20.391422 | 26.570981 | 16.251449 |
| TCGA-AB-2988-03B-01T-0748-13 | High risk | 5.061045 | 6.1852475 | 5.8344603 | 4.096358 | 3.6314949 | 6.0146704 |
| TCGA-AB-2990-03B-01T-0748-13 | Low risk | 5.7975045 | 15.165704 | 9.8782225 | 11.360109 | 13.742908 | 12.787985 |
| TCGA-AB-2991-03A-01T-0735-13 | High risk | 10.570976 | 8.8879431 | 9.4909524 | 10.593738 | 9.7617878 | 12.368211 |
| TCGA-AB-2992-03A-01T-0735-13 | Low risk | 9.0606739 | 3.8127918 | 8.5770363 | 18.568017 | 13.634687 | 17.025177 |
| TCGA-AB-2994-03A-01T-0735-13 | Low risk | 7.5887072 | 6.2478345 | 6.832062 | 11.081451 | 7.5291465 | 11.373579 |
| TCGA-AB-2995-03A-01T-0735-13 | High risk | 6.9375561 | 8.3817385 | 8.8563266 | 8.2978608 | 9.599085 | 11.225639 |
| TCGA-AB-2996-03A-01T-0735-13 | Low risk | 6.1251188 | 4.1655391 | 5.8363404 | 10.276752 | 9.1184558 | 11.831088 |
| TCGA-AB-2998-03A-01T-0735-13 | High risk | 8.5940691 | 7.6755674 | 8.9318755 | 11.621121 | 9.3718235 | 10.84149 |
| TCGA-AB-2999-03B-01T-0748-13 | Low risk | 13.816827 | 18.531293 | 11.819372 | 11.113035 | 18.564906 | 13.088831 |
| TCGA-AB-3000-03A-01T-0736-13 | Low risk | 5.4843038 | 5.478966 | 4.7942685 | 5.7162625 | 3.7656198 | 8.0060504 |
| TCGA-AB-3001-03A-01T-0736-13 | Low risk | 12.000024 | 7.1296081 | 8.0961706 | 10.090111 | 11.798007 | 13.483057 |
| TCGA-AB-3002-03A-01T-0736-13 | High risk | 4.2828706 | 10.019333 | 9.1691187 | 12.638863 | 9.1174817 | 12.634532 |
| TCGA-AB-3007-03A-01T-0736-13 | High risk | 10.965966 | 5.4050104 | 7.7827354 | 9.0899117 | 13.042734 | 9.2556608 |
| TCGA-AB-3008-03A-01T-0736-13 | Low risk | 3.9320494 | 8.3545579 | 6.4383671 | 7.1223082 | 3.6622718 | 10.087266 |
| TCGA-AB-3009-03A-01T-0736-13 | High risk | 6.3209525 | 3.6837057 | 4.8833269 | 5.8073309 | 4.9774821 | 7.9171737 |
| TCGA-AB-3011-03A-01T-0736-13 | Low risk | 6.044559 | 4.6114997 | 7.4971905 | 5.5776838 | 5.4807127 | 10.23429 |
| TCGA-AB-3012-03A-01T-0736-13 | Low risk | 14.510899 | 8.8721926 | 8.0461288 | 11.153531 | 12.942838 | 12.955348 |
| Median IC 50 | NA | 7.1197931 | 7.9288708 | 8.4378513 | 8.7756499 | 9.1174817 | 10.102959 |

**Supplementary Table 8 (Continue)**

| **TCGA ID** | Group | OTX015_1626 | Axitinib_1021 | BMS-345541_1249 | Sinularin_1838 | Palbociclib_1054 | Entospletinib_1630 |
| --- | --- | --- | --- | --- | --- | --- | --- |
| TCGA-AB-2805-03A-01T-0734-13 | Low risk | 14.147068 | 19.608808 | 20.346254 | 32.300628 | 43.003658 | 33.202662 |
| TCGA-AB-2806-03A-01T-0734-13 | Low risk | 5.95417 | 26.341799 | 13.821107 | 24.373695 | 32.465977 | 43.150271 |
| TCGA-AB-2808-03A-01T-0734-13 | Low risk | 11.417608 | 22.258592 | 23.883062 | 28.479337 | 49.255659 | 44.645446 |
| TCGA-AB-2810-03A-01T-0736-13 | High risk | 12.992385 | 28.013845 | 55.785683 | 55.827909 | 61.191542 | 56.989429 |
| TCGA-AB-2811-03B-01T-0760-13 | High risk | 10.414103 | 35.847298 | 42.225461 | 59.981451 | 35.329475 | 40.182569 |
| TCGA-AB-2812-03A-01T-0734-13 | Low risk | 12.63264 | 27.769239 | 33.61268 | 41.725877 | 66.668403 | 62.402623 |
| TCGA-AB-2813-03A-01T-0736-13 | High risk | 23.154523 | 21.504837 | 64.334829 | 57.734198 | 96.996354 | 40.044875 |
| TCGA-AB-2814-03A-01T-0734-13 | High risk | 11.486292 | 27.146421 | 16.906959 | 31.487975 | 48.600978 | 45.605827 |
| TCGA-AB-2815-03A-01T-0734-13 | Low risk | 17.453872 | 25.233226 | 44.198495 | 45.975055 | 81.066408 | 41.264863 |
| TCGA-AB-2817-03A-01T-0736-13 | High risk | 8.8331772 | 17.463929 | 16.855145 | 22.90378 | 14.451735 | 32.33177 |
| TCGA-AB-2818-03A-01T-0734-13 | Low risk | 7.6764356 | 18.807818 | 22.18199 | 34.531879 | 34.903011 | 30.403253 |
| TCGA-AB-2819-03A-01T-0734-13 | Low risk | 12.730742 | 28.753125 | 21.78283 | 28.401998 | 36.671367 | 37.833623 |
| TCGA-AB-2820-03A-01T-0735-13 | High risk | 7.127941 | 17.442819 | 23.275533 | 24.720487 | 34.5797 | 37.212379 |
| TCGA-AB-2821-03A-01T-0735-13 | Low risk | 10.718862 | 17.304313 | 23.007919 | 37.008065 | 40.46868 | 36.70631 |
| TCGA-AB-2822-03A-01T-0734-13 | Low risk | 14.334995 | 27.609981 | 20.190274 | 36.941488 | 44.872074 | 52.013083 |
| TCGA-AB-2823-03A-01T-0736-13 | High risk | 7.7508825 | 24.492111 | 23.556681 | 38.650376 | 33.626556 | 32.651866 |
| TCGA-AB-2825-03A-01T-0736-13 | High risk | 9.4181819 | 18.405512 | 19.805812 | 30.960665 | 35.405031 | 29.842542 |
| TCGA-AB-2826-03A-01T-0734-13 | Low risk | 19.056374 | 29.739093 | 64.418495 | 55.189128 | 110.90303 | 47.552792 |
| TCGA-AB-2828-03A-01T-0734-13 | Low risk | 10.711787 | 21.621144 | 27.955244 | 35.58681 | 47.487397 | 39.08533 |
| TCGA-AB-2830-03A-01T-0736-13 | High risk | 8.7152544 | 15.394011 | 27.256118 | 44.514805 | 19.530727 | 38.080278 |
| TCGA-AB-2834-03A-01T-0734-13 | Low risk | 16.994376 | 25.519534 | 41.812817 | 41.528581 | 78.458939 | 43.971014 |
| TCGA-AB-2835-03A-01T-0736-13 | High risk | 8.776753 | 20.108273 | 26.596627 | 38.7173 | 33.301078 | 33.122637 |
| TCGA-AB-2836-03A-01T-0736-13 | High risk | 7.9855641 | 18.418359 | 19.032749 | 28.33818 | 24.052856 | 25.56243 |
| TCGA-AB-2839-03A-01T-0734-13 | Low risk | 10.824032 | 24.738948 | 29.664543 | 42.034809 | 45.450925 | 55.785245 |
| TCGA-AB-2840-03A-01T-0734-13 | Low risk | 23.218125 | 21.102786 | 50.766507 | 49.260025 | 73.005498 | 49.860386 |
| TCGA-AB-2841-03B-01T-0760-13 | Low risk | 15.593741 | 36.635119 | 102.78557 | 71.671763 | 84.112311 | 60.516844 |
| TCGA-AB-2842-03A-01T-0734-13 | High risk | 18.773227 | 39.703827 | 59.815745 | 61.113577 | 127.05464 | 54.006778 |
| TCGA-AB-2843-03A-01T-0736-13 | High risk | 7.7907397 | 17.04775 | 18.703047 | 28.440355 | 21.382144 | 26.945806 |
| TCGA-AB-2844-03A-01T-0736-13 | Low risk | 6.8589279 | 17.869136 | 16.614403 | 28.50928 | 19.014855 | 29.821551 |
| TCGA-AB-2845-03B-01T-0748-13 | High risk | 7.0490907 | 17.342202 | 18.68191 | 25.83368 | 20.840279 | 30.005325 |
| TCGA-AB-2846-03A-01T-0736-13 | High risk | 8.498398 | 17.969463 | 21.250356 | 34.277145 | 27.301859 | 30.59882 |
| TCGA-AB-2847-03A-01T-0736-13 | High risk | 14.916769 | 18.483139 | 20.206196 | 29.385047 | 25.006877 | 37.731619 |
| TCGA-AB-2849-03A-01T-0734-13 | Low risk | 28.562609 | 26.780093 | 23.465841 | 28.002072 | 64.617614 | 65.623581 |
| TCGA-AB-2851-03A-01T-0736-13 | High risk | 8.4783285 | 21.757118 | 29.186512 | 40.818373 | 34.814312 | 30.601596 |
| TCGA-AB-2853-03A-01T-0734-13 | Low risk | 12.403018 | 30.173453 | 20.335671 | 31.990166 | 40.317787 | 52.500463 |
| TCGA-AB-2856-03A-01T-0736-13 | High risk | 8.0802603 | 21.662265 | 31.157971 | 36.443697 | 19.702682 | 29.536666 |
| TCGA-AB-2857-03A-01T-0736-13 | High risk | 10.782468 | 15.517975 | 25.995605 | 19.847238 | 29.845843 | 45.185382 |
| TCGA-AB-2858-03A-01T-0736-13 | Low risk | 10.677614 | 23.098731 | 30.663938 | 31.941702 | 28.852607 | 45.394517 |
| TCGA-AB-2859-03A-01T-0736-13 | High risk | 8.9541141 | 18.452797 | 19.228523 | 33.088808 | 23.027383 | 37.103809 |
| TCGA-AB-2861-03A-01T-0736-13 | Low risk | 5.6840996 | 16.636726 | 25.191494 | 33.466575 | 21.695853 | 40.249265 |
| TCGA-AB-2862-03A-01T-0736-13 | Low risk | 10.083168 | 22.375647 | 33.894174 | 38.000588 | 30.182323 | 39.768079 |
| TCGA-AB-2863-03A-01T-0734-13 | High risk | 11.656505 | 25.053659 | 13.248206 | 30.849441 | 44.33228 | 40.629806 |
| TCGA-AB-2865-03A-01T-0736-13 | High risk | 13.699442 | 21.927713 | 21.088385 | 34.923563 | 41.327 | 34.444228 |
| TCGA-AB-2866-03A-01T-0736-13 | High risk | 9.2829927 | 17.792664 | 23.725965 | 39.869049 | 32.17716 | 36.483762 |
| TCGA-AB-2867-03A-01T-0734-13 | Low risk | 15.516131 | 24.988583 | 38.556494 | 47.744165 | 83.487575 | 50.225335 |
| TCGA-AB-2869-03A-01T-0735-13 | Low risk | 9.6731697 | 21.176656 | 17.03111 | 25.716032 | 19.393114 | 33.625422 |
| TCGA-AB-2870-03A-01T-0735-13 | High risk | 9.3396622 | 17.207912 | 19.633615 | 26.478579 | 29.830293 | 25.216913 |
| TCGA-AB-2871-03A-01T-0735-13 | Low risk | 7.0387362 | 14.285479 | 16.707406 | 25.073619 | 15.274881 | 36.711948 |
| TCGA-AB-2872-03A-01T-0735-13 | Low risk | 14.304911 | 31.001671 | 73.828694 | 55.428076 | 59.202581 | 58.786302 |
| TCGA-AB-2873-03A-01T-0735-13 | High risk | 13.177818 | 17.486199 | 54.35342 | 44.77137 | 77.861197 | 47.667107 |
| TCGA-AB-2874-03A-01T-0735-13 | Low risk | 8.8655957 | 26.180961 | 16.683186 | 27.377893 | 27.91467 | 40.419516 |
| TCGA-AB-2875-03A-01T-0735-13 | Low risk | 7.3335942 | 21.955653 | 17.025015 | 23.949731 | 16.57442 | 31.629708 |
| TCGA-AB-2876-03A-01T-0734-13 | Low risk | 11.926397 | 26.851175 | 31.733288 | 39.918933 | 48.617373 | 48.301669 |
| TCGA-AB-2877-03A-01T-0735-13 | Low risk | 11.724163 | 19.291354 | 24.289645 | 25.72969 | 25.75437 | 45.371089 |
| TCGA-AB-2878-03A-01T-0734-13 | Low risk | 28.139584 | 29.774462 | 59.043674 | 44.472378 | 127.37373 | 69.962083 |
| TCGA-AB-2880-03A-01T-0735-13 | Low risk | 6.7247255 | 17.867002 | 17.997852 | 23.374468 | 18.973528 | 31.292631 |
| TCGA-AB-2881-03A-01T-0735-13 | High risk | 11.6455 | 19.341955 | 20.844274 | 31.617909 | 21.984616 | 32.68436 |
| TCGA-AB-2882-03A-01T-0740-13 | Low risk | 12.693901 | 19.726341 | 30.968588 | 34.233677 | 38.722978 | 46.510527 |
| TCGA-AB-2883-03A-01T-0734-13 | Low risk | 20.772067 | 30.828738 | 69.20486 | 65.192311 | 84.159131 | 49.925743 |
| TCGA-AB-2884-03A-01T-0735-13 | High risk | 8.9533911 | 16.543022 | 20.957218 | 30.907171 | 24.625788 | 34.14646 |
| TCGA-AB-2885-03A-01T-0735-13 | High risk | 13.592442 | 17.333575 | 18.319638 | 27.006925 | 16.162314 | 39.1455 |
| TCGA-AB-2886-03A-01T-0735-13 | Low risk | 7.7703635 | 20.694299 | 13.466132 | 19.585759 | 17.533938 | 29.786897 |
| TCGA-AB-2888-03B-01T-0748-13 | High risk | 10.517587 | 17.409389 | 25.245203 | 33.739802 | 35.318505 | 30.370499 |
| TCGA-AB-2889-03A-01T-0735-13 | Low risk | 11.24294 | 22.235626 | 24.453819 | 35.580913 | 32.630347 | 33.702123 |
| TCGA-AB-2890-03A-01T-0735-13 | High risk | 11.830996 | 15.844544 | 22.477862 | 34.301236 | 27.944954 | 38.47041 |
| TCGA-AB-2891-03A-01T-0735-13 | High risk | 5.3750494 | 14.104927 | 21.005731 | 24.578235 | 30.595867 | 31.242703 |
| TCGA-AB-2892-03A-01T-0734-13 | Low risk | 18.975931 | 28.551682 | 69.5386 | 58.135679 | 85.238686 | 53.851904 |
| TCGA-AB-2893-03A-01T-0734-13 | High risk | 13.182242 | 21.188956 | 39.608493 | 50.339288 | 54.590501 | 38.450669 |
| TCGA-AB-2894-03A-01T-0734-13 | Low risk | 8.3130925 | 19.618724 | 28.365115 | 34.941268 | 23.36298 | 42.964917 |
| TCGA-AB-2895-03A-01T-0735-13 | High risk | 10.891959 | 20.822151 | 14.337347 | 27.272605 | 18.017174 | 32.779849 |
| TCGA-AB-2896-03B-01T-0751-13 | High risk | 10.858367 | 18.638395 | 16.882426 | 30.14325 | 15.278305 | 30.119505 |
| TCGA-AB-2897-03A-01T-0735-13 | High risk | 9.941239 | 27.547387 | 60.471182 | 59.539388 | 55.306039 | 67.592161 |
| TCGA-AB-2898-03A-01T-0735-13 | High risk | 15.429734 | 20.994958 | 24.223764 | 39.588049 | 51.430395 | 42.000272 |
| TCGA-AB-2899-03A-01T-0736-13 | Low risk | 9.3573517 | 16.305445 | 20.338289 | 26.6447 | 25.014586 | 28.517891 |
| TCGA-AB-2900-03A-01T-0735-13 | High risk | 11.274048 | 21.282702 | 29.228092 | 42.109073 | 41.333876 | 36.336081 |
| TCGA-AB-2901-03A-01T-0735-13 | High risk | 17.054356 | 25.069131 | 19.588091 | 33.137673 | 47.36333 | 37.581328 |
| TCGA-AB-2908-03A-01T-0740-13 | High risk | 19.49168 | 24.098896 | 79.036424 | 51.898172 | 98.733362 | 54.548252 |
| TCGA-AB-2910-03A-01T-0740-13 | High risk | 11.424748 | 14.782609 | 30.830298 | 24.734537 | 27.026683 | 55.163407 |
| TCGA-AB-2911-03A-01T-0734-13 | Low risk | 24.343852 | 34.507806 | 87.677362 | 65.97416 | 104.50789 | 45.055341 |
| TCGA-AB-2912-03A-01T-0734-13 | High risk | 17.726576 | 22.432048 | 41.792548 | 48.080389 | 67.038892 | 53.28629 |
| TCGA-AB-2913-03A-01T-0734-13 | Low risk | 18.271681 | 28.570061 | 49.487046 | 50.134822 | 58.609317 | 66.162223 |
| TCGA-AB-2914-03A-01T-0734-13 | Low risk | 13.911641 | 24.721961 | 34.290599 | 43.299575 | 53.545559 | 48.114059 |
| TCGA-AB-2915-03A-01T-0740-13 | High risk | 7.8813456 | 20.601461 | 14.841721 | 26.840297 | 20.491624 | 27.050082 |
| TCGA-AB-2916-03A-01T-0734-13 | Low risk | 16.507174 | 22.468051 | 59.897821 | 51.072148 | 89.339323 | 46.417778 |
| TCGA-AB-2917-03A-01T-0734-13 | Low risk | 28.169443 | 24.607118 | 34.731131 | 33.759342 | 80.762498 | 91.96533 |
| TCGA-AB-2918-03A-01T-0740-13 | High risk | 8.6424632 | 20.832876 | 17.010623 | 29.456204 | 19.704068 | 28.214266 |
| TCGA-AB-2919-03A-01T-0740-13 | Low risk | 9.353761 | 18.53248 | 12.62573 | 20.380061 | 13.708367 | 32.234993 |
| TCGA-AB-2920-03B-01T-0760-13 | High risk | 12.101889 | 28.608211 | 23.877602 | 34.924004 | 29.948464 | 35.489869 |
| TCGA-AB-2921-03A-01T-0740-13 | High risk | 8.3248963 | 19.169995 | 16.515594 | 28.457401 | 16.161315 | 30.13086 |
| TCGA-AB-2924-03A-01T-0740-13 | High risk | 7.0295407 | 19.081714 | 21.506939 | 30.849467 | 21.491062 | 34.431579 |
| TCGA-AB-2925-03A-01T-0735-13 | High risk | 8.448679 | 21.039993 | 39.847024 | 42.610329 | 36.488354 | 38.026728 |
| TCGA-AB-2927-03A-01T-0740-13 | High risk | 15.424507 | 17.362231 | 39.43096 | 40.010624 | 57.250054 | 46.242091 |
| TCGA-AB-2928-03A-01T-0740-13 | High risk | 11.432268 | 24.000057 | 9.8744477 | 21.741393 | 13.930673 | 43.231327 |
| TCGA-AB-2929-03A-01T-0735-13 | High risk | 9.9511748 | 16.280268 | 13.740703 | 21.026442 | 13.41861 | 35.434961 |
| TCGA-AB-2930-03A-01T-0740-13 | High risk | 6.8521377 | 18.204993 | 16.637702 | 21.987699 | 14.602787 | 26.943061 |
| TCGA-AB-2931-03A-01T-0740-13 | High risk | 8.3556803 | 22.155168 | 18.916251 | 25.835758 | 16.968142 | 30.155191 |
| TCGA-AB-2932-03A-01T-0740-13 | High risk | 9.6044903 | 19.384646 | 24.444861 | 36.987709 | 27.364505 | 30.583702 |
| TCGA-AB-2933-03A-01T-0734-13 | High risk | 23.398265 | 23.777319 | 54.779194 | 52.522034 | 90.767388 | 58.863406 |
| TCGA-AB-2934-03A-01T-0740-13 | High risk | 13.589401 | 22.336714 | 34.333955 | 37.116803 | 39.794359 | 31.861497 |
| TCGA-AB-2935-03A-01T-0740-13 | High risk | 10.960796 | 13.876479 | 17.237378 | 34.484981 | 31.983521 | 39.178002 |
| TCGA-AB-2936-03A-01T-0740-13 | Low risk | 15.74892 | 18.103409 | 22.834148 | 32.566078 | 33.919744 | 44.9586 |
| TCGA-AB-2937-03A-01T-0734-13 | Low risk | 40.375354 | 58.115957 | 143.22146 | 103.54429 | 147.07252 | 69.307272 |
| TCGA-AB-2938-03A-01T-0736-13 | High risk | 22.774967 | 17.05485 | 44.643208 | 32.745241 | 45.901792 | 50.24313 |
| TCGA-AB-2939-03A-01T-0740-13 | High risk | 8.5164811 | 19.670383 | 14.668789 | 25.939925 | 18.952265 | 26.156882 |
| TCGA-AB-2940-03A-01T-0736-13 | Low risk | 6.9858845 | 15.736153 | 22.691786 | 27.133844 | 27.768713 | 34.796896 |
| TCGA-AB-2941-03A-01T-0740-13 | High risk | 18.404166 | 11.098924 | 16.060668 | 23.833051 | 175.1314 | 61.530165 |
| TCGA-AB-2942-03A-01T-0734-13 | Low risk | 14.532906 | 24.425642 | 32.655631 | 43.312809 | 39.053106 | 41.643255 |
| TCGA-AB-2943-03A-01T-0740-13 | High risk | 11.925526 | 13.136716 | 40.591076 | 28.495868 | 39.477578 | 41.724076 |
| TCGA-AB-2944-03A-01T-0740-13 | High risk | 10.657572 | 23.076036 | 17.541368 | 26.462621 | 17.574042 | 39.33367 |
| TCGA-AB-2946-03A-01T-0740-13 | Low risk | 6.5784174 | 15.955219 | 22.953491 | 31.763409 | 24.560571 | 34.736271 |
| TCGA-AB-2948-03A-01T-0740-13 | High risk | 10.784058 | 17.132642 | 26.019927 | 34.510805 | 44.660584 | 38.472249 |
| TCGA-AB-2949-03B-01T-0748-13 | High risk | 12.743915 | 19.334852 | 16.457762 | 27.13106 | 25.646124 | 35.534468 |
| TCGA-AB-2950-03A-01T-0735-13 | Low risk | 12.671315 | 25.734593 | 29.96902 | 30.738966 | 41.840974 | 45.972572 |
| TCGA-AB-2952-03B-01T-0760-13 | High risk | 9.9246897 | 24.068977 | 36.339483 | 31.001125 | 44.075376 | 31.978725 |
| TCGA-AB-2955-03A-01T-0734-13 | Low risk | 12.868939 | 24.6779 | 47.905294 | 33.866708 | 33.414773 | 50.016055 |
| TCGA-AB-2956-03A-01T-0740-13 | High risk | 6.1883966 | 13.632238 | 19.938853 | 27.428676 | 26.564452 | 28.86007 |
| TCGA-AB-2959-03A-01T-0734-13 | Low risk | 18.798561 | 20.566959 | 27.250926 | 35.524521 | 65.887733 | 50.041244 |
| TCGA-AB-2963-03A-01T-0734-13 | Low risk | 11.391394 | 26.624469 | 23.227375 | 31.804023 | 46.768546 | 47.678915 |
| TCGA-AB-2965-03A-01T-0734-13 | Low risk | 16.008572 | 23.167766 | 39.149325 | 41.15276 | 66.144354 | 48.553406 |
| TCGA-AB-2966-03A-01T-0734-13 | Low risk | 13.07966 | 29.723581 | 31.509544 | 39.410817 | 47.349096 | 47.850821 |
| TCGA-AB-2970-03A-01T-0734-13 | Low risk | 15.110356 | 18.134644 | 29.72493 | 40.813841 | 44.90305 | 38.920287 |
| TCGA-AB-2971-03A-01T-0734-13 | Low risk | 15.895291 | 18.541933 | 40.172674 | 40.788998 | 41.884692 | 47.610769 |
| TCGA-AB-2973-03A-01T-0734-13 | Low risk | 29.876065 | 29.727136 | 115.53753 | 70.204334 | 116.00575 | 54.507044 |
| TCGA-AB-2975-03A-01T-0734-13 | Low risk | 15.227194 | 25.293369 | 41.441373 | 42.664043 | 43.72213 | 58.644375 |
| TCGA-AB-2976-03A-01T-0734-13 | Low risk | 20.651801 | 24.45642 | 37.537382 | 45.347384 | 56.880322 | 56.734195 |
| TCGA-AB-2977-03B-01T-0760-13 | Low risk | 14.754041 | 31.967768 | 52.213874 | 66.547248 | 46.147027 | 41.376114 |
| TCGA-AB-2979-03B-01T-0760-13 | Low risk | 5.3603324 | 23.400069 | 26.88125 | 26.032939 | 20.340174 | 30.687721 |
| TCGA-AB-2980-03A-01T-0734-13 | Low risk | 19.832367 | 26.031899 | 43.751537 | 38.9003 | 62.635346 | 63.415961 |
| TCGA-AB-2981-03B-01T-0748-13 | High risk | 12.386015 | 22.818505 | 26.378608 | 33.264035 | 42.60229 | 25.08879 |
| TCGA-AB-2982-03B-01T-0748-13 | Low risk | 14.642778 | 20.759767 | 49.059011 | 47.210605 | 43.362137 | 53.435503 |
| TCGA-AB-2983-03A-01T-0734-13 | High risk | 12.187996 | 18.679856 | 30.11839 | 42.438109 | 48.492679 | 45.609073 |
| TCGA-AB-2984-03A-01T-0734-13 | Low risk | 29.304073 | 58.465168 | 57.09458 | 108.46499 | 235.81499 | 97.011693 |
| TCGA-AB-2986-03A-01T-0734-13 | Low risk | 17.744485 | 40.84164 | 50.546074 | 54.124922 | 44.817375 | 54.215585 |
| TCGA-AB-2987-03A-01T-0734-13 | High risk | 20.832662 | 21.314203 | 66.311661 | 48.033965 | 90.292333 | 41.167297 |
| TCGA-AB-2988-03B-01T-0748-13 | High risk | 8.5000977 | 20.70641 | 14.524242 | 24.427099 | 14.140326 | 29.273967 |
| TCGA-AB-2990-03B-01T-0748-13 | Low risk | 17.573511 | 22.627496 | 40.711692 | 40.229885 | 45.415173 | 53.119533 |
| TCGA-AB-2991-03A-01T-0735-13 | High risk | 7.895954 | 21.305534 | 30.845324 | 41.055612 | 46.902769 | 50.876207 |
| TCGA-AB-2992-03A-01T-0735-13 | Low risk | 18.069973 | 21.747773 | 58.326754 | 47.110016 | 83.456191 | 39.418139 |
| TCGA-AB-2994-03A-01T-0735-13 | Low risk | 9.4951882 | 19.77277 | 32.038259 | 41.248596 | 39.070474 | 34.456301 |
| TCGA-AB-2995-03A-01T-0735-13 | High risk | 20.905328 | 15.619242 | 25.611857 | 29.362189 | 37.64995 | 57.129848 |
| TCGA-AB-2996-03A-01T-0735-13 | Low risk | 13.170454 | 21.227409 | 29.005805 | 36.783528 | 46.436847 | 40.020478 |
| TCGA-AB-2998-03A-01T-0735-13 | High risk | 10.129512 | 19.492588 | 27.528316 | 40.160584 | 52.103551 | 38.002783 |
| TCGA-AB-2999-03B-01T-0748-13 | Low risk | 12.297154 | 24.97576 | 52.193523 | 46.906433 | 63.503934 | 67.149506 |
| TCGA-AB-3000-03A-01T-0736-13 | Low risk | 6.1998075 | 17.27593 | 16.454323 | 23.914384 | 17.599765 | 27.7697 |
| TCGA-AB-3001-03A-01T-0736-13 | Low risk | 8.2901569 | 18.76649 | 29.957831 | 40.792331 | 50.794539 | 49.587604 |
| TCGA-AB-3002-03A-01T-0736-13 | High risk | 14.258221 | 26.613715 | 29.02826 | 36.232012 | 58.908782 | 45.081928 |
| TCGA-AB-3007-03A-01T-0736-13 | High risk | 10.945363 | 21.177531 | 23.094473 | 39.436262 | 34.870301 | 45.862752 |
| TCGA-AB-3008-03A-01T-0736-13 | Low risk | 6.9289672 | 18.435209 | 22.014112 | 21.485678 | 23.361299 | 32.835361 |
| TCGA-AB-3009-03A-01T-0736-13 | High risk | 8.1391103 | 14.162068 | 8.2525846 | 23.716269 | 23.963613 | 26.643262 |
| TCGA-AB-3011-03A-01T-0736-13 | Low risk | 9.2341925 | 15.494172 | 18.929586 | 25.800972 | 25.225948 | 39.154089 |
| TCGA-AB-3012-03A-01T-0736-13 | Low risk | 9.0050502 | 24.495703 | 47.034661 | 47.263719 | 57.359962 | 52.141212 |
| Median IC 50 | NA | 11.432268 | 21.188956 | 26.378608 | 34.531879 | 39.070474 | 40.044875 |

**Supplementary Table 8 (Continue)**

| **TCGA ID** | Group | Ribociclib_1632 | PF-4708671_1129 | LGK974_1598 | VE821_2111 | Wnt-C59_1622 | I-BRD9_1928 |
| --- | --- | --- | --- | --- | --- | --- | --- |
| TCGA-AB-2805-03A-01T-0734-13 | Low risk | 34.069762 | 49.593859 | 50.33026 | 53.058995 | 56.055354 | 63.339819 |
| TCGA-AB-2806-03A-01T-0734-13 | Low risk | 51.835502 | 85.843782 | 45.301622 | 50.895534 | 57.515175 | 43.337683 |
| TCGA-AB-2808-03A-01T-0734-13 | Low risk | 40.801297 | 64.824038 | 45.195206 | 50.606408 | 60.325245 | 63.726102 |
| TCGA-AB-2810-03A-01T-0736-13 | High risk | 72.53783 | 47.92206 | 83.074055 | 125.15076 | 104.57509 | 152.18889 |
| TCGA-AB-2811-03B-01T-0760-13 | High risk | 57.895454 | 41.212284 | 118.48583 | 90.470726 | 103.30054 | 141.71337 |
| TCGA-AB-2812-03A-01T-0734-13 | Low risk | 69.047363 | 79.974129 | 67.521345 | 106.24666 | 103.40216 | 103.72946 |
| TCGA-AB-2813-03A-01T-0736-13 | High risk | 36.060451 | 38.965757 | 59.528801 | 93.217463 | 90.171884 | 141.09257 |
| TCGA-AB-2814-03A-01T-0734-13 | High risk | 56.937947 | 71.81565 | 71.216453 | 70.915495 | 64.253966 | 78.038388 |
| TCGA-AB-2815-03A-01T-0734-13 | Low risk | 40.198384 | 55.282567 | 67.27291 | 100.18545 | 97.34847 | 118.14165 |
| TCGA-AB-2817-03A-01T-0736-13 | High risk | 34.744361 | 38.543828 | 55.996678 | 23.777964 | 53.473815 | 50.913601 |
| TCGA-AB-2818-03A-01T-0734-13 | Low risk | 32.302768 | 40.355958 | 46.248336 | 49.485774 | 57.260651 | 52.427049 |
| TCGA-AB-2819-03A-01T-0734-13 | Low risk | 54.344885 | 78.791952 | 57.559943 | 52.745821 | 73.229446 | 75.780906 |
| TCGA-AB-2820-03A-01T-0735-13 | High risk | 48.602595 | 42.424329 | 41.42554 | 68.655217 | 52.138318 | 69.990852 |
| TCGA-AB-2821-03A-01T-0735-13 | Low risk | 39.489429 | 43.171117 | 51.710594 | 54.131141 | 68.286547 | 66.516599 |
| TCGA-AB-2822-03A-01T-0734-13 | Low risk | 61.283348 | 76.656699 | 78.873121 | 73.857529 | 77.056736 | 75.397856 |
| TCGA-AB-2823-03A-01T-0736-13 | High risk | 48.520097 | 52.064563 | 58.894851 | 78.170387 | 70.586221 | 57.540674 |
| TCGA-AB-2825-03A-01T-0736-13 | High risk | 31.289105 | 33.04119 | 32.509255 | 44.429844 | 58.009191 | 47.600879 |
| TCGA-AB-2826-03A-01T-0734-13 | Low risk | 49.221074 | 53.558015 | 81.121545 | 117.62761 | 89.261803 | 153.77625 |
| TCGA-AB-2828-03A-01T-0734-13 | Low risk | 37.479825 | 52.246185 | 47.996012 | 61.725771 | 73.350477 | 71.476042 |
| TCGA-AB-2830-03A-01T-0736-13 | High risk | 34.28823 | 34.647495 | 54.435755 | 54.089701 | 63.242723 | 72.022101 |
| TCGA-AB-2834-03A-01T-0734-13 | Low risk | 39.124693 | 53.737006 | 51.407626 | 84.3621 | 67.418363 | 83.111487 |
| TCGA-AB-2835-03A-01T-0736-13 | High risk | 35.340435 | 35.568412 | 40.050702 | 65.876863 | 72.664948 | 56.149439 |
| TCGA-AB-2836-03A-01T-0736-13 | High risk | 30.331521 | 28.441295 | 36.742264 | 36.829612 | 52.368961 | 48.639375 |
| TCGA-AB-2839-03A-01T-0734-13 | Low risk | 52.142527 | 66.770202 | 63.712332 | 69.559993 | 80.733508 | 88.614371 |
| TCGA-AB-2840-03A-01T-0734-13 | Low risk | 50.631929 | 53.067728 | 75.317241 | 114.78866 | 83.528646 | 152.27038 |
| TCGA-AB-2841-03B-01T-0760-13 | Low risk | 68.003408 | 52.839443 | 119.08465 | 202.61695 | 113.90064 | 200.51236 |
| TCGA-AB-2842-03A-01T-0734-13 | High risk | 53.979428 | 88.574712 | 79.532653 | 164.24041 | 116.2796 | 134.3309 |
| TCGA-AB-2843-03A-01T-0736-13 | High risk | 30.71304 | 28.917424 | 34.654474 | 23.890959 | 51.554462 | 61.137621 |
| TCGA-AB-2844-03A-01T-0736-13 | Low risk | 36.028344 | 40.649148 | 33.730419 | 38.76229 | 49.757488 | 39.961448 |
| TCGA-AB-2845-03B-01T-0748-13 | High risk | 35.930842 | 42.652015 | 38.294484 | 32.811878 | 45.872923 | 43.823988 |
| TCGA-AB-2846-03A-01T-0736-13 | High risk | 35.779719 | 41.659961 | 50.445392 | 50.796929 | 72.079606 | 60.149291 |
| TCGA-AB-2847-03A-01T-0736-13 | High risk | 39.581378 | 38.652398 | 46.151199 | 39.952726 | 54.753905 | 59.471863 |
| TCGA-AB-2849-03A-01T-0734-13 | Low risk | 57.415269 | 66.624394 | 61.238298 | 62.83558 | 68.047838 | 88.105118 |
| TCGA-AB-2851-03A-01T-0736-13 | High risk | 34.903389 | 30.673031 | 46.689785 | 63.541481 | 65.025911 | 76.843849 |
| TCGA-AB-2853-03A-01T-0734-13 | Low risk | 57.822705 | 83.556958 | 59.444369 | 63.931604 | 79.513936 | 61.292314 |
| TCGA-AB-2856-03A-01T-0736-13 | High risk | 39.8242 | 34.794616 | 37.486866 | 51.904044 | 65.692189 | 67.453925 |
| TCGA-AB-2857-03A-01T-0736-13 | High risk | 31.156634 | 38.802768 | 43.695547 | 20.193267 | 45.225889 | 51.466109 |
| TCGA-AB-2858-03A-01T-0736-13 | Low risk | 47.65117 | 55.840504 | 55.43566 | 60.059836 | 68.37276 | 75.984542 |
| TCGA-AB-2859-03A-01T-0736-13 | High risk | 35.770281 | 39.264854 | 51.680587 | 39.354206 | 56.979073 | 52.780348 |
| TCGA-AB-2861-03A-01T-0736-13 | Low risk | 36.484681 | 43.276723 | 24.109108 | 36.643838 | 46.869944 | 47.65557 |
| TCGA-AB-2862-03A-01T-0736-13 | Low risk | 49.626159 | 40.053604 | 58.95173 | 67.24431 | 63.182227 | 87.799859 |
| TCGA-AB-2863-03A-01T-0734-13 | High risk | 59.716672 | 95.237622 | 59.811714 | 44.439134 | 78.798135 | 47.428827 |
| TCGA-AB-2865-03A-01T-0736-13 | High risk | 37.454561 | 38.675606 | 55.062632 | 53.146951 | 65.762172 | 68.713119 |
| TCGA-AB-2866-03A-01T-0736-13 | High risk | 35.982128 | 33.855487 | 46.16542 | 62.890544 | 64.012148 | 64.954719 |
| TCGA-AB-2867-03A-01T-0734-13 | Low risk | 48.05553 | 63.982858 | 84.178368 | 94.45615 | 81.962791 | 117.90373 |
| TCGA-AB-2869-03A-01T-0735-13 | Low risk | 42.284679 | 39.725334 | 41.982531 | 34.868576 | 53.646922 | 48.649778 |
| TCGA-AB-2870-03A-01T-0735-13 | High risk | 33.235225 | 33.117532 | 40.953437 | 37.508228 | 59.511696 | 54.287009 |
| TCGA-AB-2871-03A-01T-0735-13 | Low risk | 32.812082 | 33.41417 | 46.201577 | 29.762019 | 49.689061 | 45.146845 |
| TCGA-AB-2872-03A-01T-0735-13 | Low risk | 53.053339 | 62.98186 | 94.191254 | 149.94023 | 93.207521 | 160.49523 |
| TCGA-AB-2873-03A-01T-0735-13 | High risk | 31.6056 | 33.143234 | 52.204557 | 70.496837 | 83.164898 | 98.124126 |
| TCGA-AB-2874-03A-01T-0735-13 | Low risk | 42.868015 | 62.4211 | 51.021316 | 61.777672 | 63.052437 | 46.68099 |
| TCGA-AB-2875-03A-01T-0735-13 | Low risk | 41.553874 | 50.315446 | 44.669528 | 39.212719 | 58.134952 | 44.099013 |
| TCGA-AB-2876-03A-01T-0734-13 | Low risk | 51.478768 | 84.584707 | 72.049253 | 80.63503 | 74.138958 | 81.125792 |
| TCGA-AB-2877-03A-01T-0735-13 | Low risk | 53.11301 | 41.902589 | 36.042085 | 44.820441 | 55.570388 | 57.346129 |
| TCGA-AB-2878-03A-01T-0734-13 | Low risk | 69.513263 | 74.889861 | 92.523613 | 67.157917 | 90.671479 | 122.61573 |
| TCGA-AB-2880-03A-01T-0735-13 | Low risk | 39.810186 | 45.221992 | 36.99319 | 35.215198 | 50.788335 | 43.203007 |
| TCGA-AB-2881-03A-01T-0735-13 | High risk | 37.399423 | 43.048961 | 58.636793 | 45.538171 | 72.366972 | 71.568021 |
| TCGA-AB-2882-03A-01T-0740-13 | Low risk | 33.448329 | 36.097334 | 49.449624 | 56.988281 | 61.168192 | 76.310649 |
| TCGA-AB-2883-03A-01T-0734-13 | Low risk | 60.893265 | 50.394255 | 69.678363 | 108.93528 | 91.89261 | 210.13422 |
| TCGA-AB-2884-03A-01T-0735-13 | High risk | 33.82029 | 37.856589 | 40.862678 | 41.716938 | 56.371054 | 49.357243 |
| TCGA-AB-2885-03A-01T-0735-13 | High risk | 39.492688 | 33.402811 | 48.615934 | 34.381139 | 59.979092 | 52.258092 |
| TCGA-AB-2886-03A-01T-0735-13 | Low risk | 44.581381 | 48.818643 | 40.950453 | 33.865756 | 49.074529 | 42.763937 |
| TCGA-AB-2888-03B-01T-0748-13 | High risk | 29.734154 | 33.737763 | 38.144684 | 48.757473 | 55.621924 | 67.394315 |
| TCGA-AB-2889-03A-01T-0735-13 | Low risk | 40.227141 | 44.207031 | 54.14346 | 65.074889 | 74.678773 | 78.485103 |
| TCGA-AB-2890-03A-01T-0735-13 | High risk | 34.660643 | 36.102702 | 52.607361 | 41.952031 | 57.367967 | 81.803689 |
| TCGA-AB-2891-03A-01T-0735-13 | High risk | 31.017313 | 32.8665 | 38.671143 | 51.636803 | 47.106237 | 35.470497 |
| TCGA-AB-2892-03A-01T-0734-13 | Low risk | 47.328951 | 60.51908 | 95.367774 | 135.7512 | 108.42384 | 176.49144 |
| TCGA-AB-2893-03A-01T-0734-13 | High risk | 37.142887 | 38.938451 | 49.829375 | 74.870096 | 79.887917 | 98.761928 |
| TCGA-AB-2894-03A-01T-0734-13 | Low risk | 35.758982 | 45.844746 | 37.759431 | 52.261812 | 50.441623 | 64.618672 |
| TCGA-AB-2895-03A-01T-0735-13 | High risk | 46.959735 | 42.048637 | 48.511932 | 40.596647 | 62.301715 | 42.796502 |
| TCGA-AB-2896-03B-01T-0751-13 | High risk | 44.130489 | 43.538498 | 41.988961 | 32.361774 | 54.642136 | 51.626089 |
| TCGA-AB-2897-03A-01T-0735-13 | High risk | 57.741605 | 58.102627 | 101.12081 | 167.10335 | 106.04281 | 142.37683 |
| TCGA-AB-2898-03A-01T-0735-13 | High risk | 41.440288 | 45.129525 | 67.855154 | 72.780079 | 71.591164 | 74.82639 |
| TCGA-AB-2899-03A-01T-0736-13 | Low risk | 29.315867 | 30.664876 | 43.6598 | 31.03788 | 49.207557 | 59.921024 |
| TCGA-AB-2900-03A-01T-0735-13 | High risk | 40.952799 | 43.237218 | 61.937364 | 67.408051 | 88.345371 | 80.086151 |
| TCGA-AB-2901-03A-01T-0735-13 | High risk | 57.483346 | 76.608715 | 72.987775 | 43.572001 | 82.328864 | 71.986492 |
| TCGA-AB-2908-03A-01T-0740-13 | High risk | 53.897883 | 43.035929 | 73.521487 | 95.318975 | 97.797365 | 185.60143 |
| TCGA-AB-2910-03A-01T-0740-13 | High risk | 38.824935 | 34.486155 | 28.717232 | 50.908361 | 56.196986 | 40.00112 |
| TCGA-AB-2911-03A-01T-0734-13 | Low risk | 50.805941 | 47.085499 | 82.42388 | 133.33636 | 89.362469 | 252.5165 |
| TCGA-AB-2912-03A-01T-0734-13 | High risk | 44.235254 | 71.585202 | 78.125731 | 71.043131 | 99.040806 | 100.91296 |
| TCGA-AB-2913-03A-01T-0734-13 | Low risk | 61.952512 | 75.81325 | 85.656952 | 120.21692 | 93.634728 | 143.6372 |
| TCGA-AB-2914-03A-01T-0734-13 | Low risk | 47.974929 | 74.017059 | 90.586314 | 78.040473 | 91.521721 | 116.53088 |
| TCGA-AB-2915-03A-01T-0740-13 | High risk | 35.779198 | 36.419397 | 43.782615 | 29.51382 | 50.034527 | 48.17403 |
| TCGA-AB-2916-03A-01T-0734-13 | Low risk | 36.482596 | 46.233927 | 79.608596 | 137.49041 | 81.300433 | 154.3615 |
| TCGA-AB-2917-03A-01T-0734-13 | Low risk | 56.845153 | 78.12487 | 83.657097 | 59.812725 | 80.275099 | 88.116271 |
| TCGA-AB-2918-03A-01T-0740-13 | High risk | 47.048139 | 50.620019 | 33.035236 | 42.629526 | 56.399436 | 46.847347 |
| TCGA-AB-2919-03A-01T-0740-13 | Low risk | 49.085384 | 43.722781 | 38.032068 | 27.361322 | 45.978075 | 36.715837 |
| TCGA-AB-2920-03B-01T-0760-13 | High risk | 59.038512 | 36.993391 | 58.416001 | 62.093001 | 58.007708 | 80.715436 |
| TCGA-AB-2921-03A-01T-0740-13 | High risk | 37.604657 | 39.409133 | 34.715985 | 35.155712 | 52.220684 | 40.746501 |
| TCGA-AB-2924-03A-01T-0740-13 | High risk | 34.053746 | 38.25726 | 34.450824 | 41.917205 | 58.084775 | 46.363798 |
| TCGA-AB-2925-03A-01T-0735-13 | High risk | 37.249114 | 35.275657 | 38.755739 | 72.64829 | 69.285609 | 77.778192 |
| TCGA-AB-2927-03A-01T-0740-13 | High risk | 36.296826 | 34.313442 | 64.223392 | 63.426549 | 77.48724 | 139.0313 |
| TCGA-AB-2928-03A-01T-0740-13 | High risk | 58.061953 | 70.582951 | 53.073608 | 31.218092 | 60.776543 | 32.499871 |
| TCGA-AB-2929-03A-01T-0735-13 | High risk | 33.17415 | 37.748151 | 35.142382 | 25.37742 | 38.936806 | 31.230807 |
| TCGA-AB-2930-03A-01T-0740-13 | High risk | 39.185961 | 45.550854 | 28.526923 | 27.104846 | 44.951351 | 33.334232 |
| TCGA-AB-2931-03A-01T-0740-13 | High risk | 49.946297 | 42.646443 | 44.459553 | 30.261598 | 63.81476 | 41.675285 |
| TCGA-AB-2932-03A-01T-0740-13 | High risk | 34.658768 | 33.539255 | 41.166434 | 52.356272 | 70.865026 | 62.697874 |
| TCGA-AB-2933-03A-01T-0734-13 | High risk | 44.051761 | 52.703493 | 126.19127 | 109.09365 | 108.52038 | 157.6787 |
| TCGA-AB-2934-03A-01T-0740-13 | High risk | 40.778763 | 35.011311 | 50.716489 | 54.02735 | 65.899826 | 90.144573 |
| TCGA-AB-2935-03A-01T-0740-13 | High risk | 28.919829 | 33.161008 | 37.413365 | 26.13119 | 41.127844 | 53.502986 |
| TCGA-AB-2936-03A-01T-0740-13 | Low risk | 38.517089 | 42.996848 | 52.136819 | 38.136894 | 54.861695 | 76.498401 |
| TCGA-AB-2937-03A-01T-0734-13 | Low risk | 108.27065 | 79.542689 | 222.48537 | 328.50694 | 188.27457 | 525.59696 |
| TCGA-AB-2938-03A-01T-0736-13 | High risk | 34.464034 | 41.886939 | 56.225207 | 26.121467 | 58.885689 | 103.4436 |
| TCGA-AB-2939-03A-01T-0740-13 | High risk | 40.713725 | 48.295856 | 41.119034 | 31.82397 | 51.451176 | 39.118681 |
| TCGA-AB-2940-03A-01T-0736-13 | Low risk | 32.807595 | 36.941391 | 44.399332 | 39.475791 | 47.931658 | 51.930409 |
| TCGA-AB-2941-03A-01T-0740-13 | High risk | 47.705101 | 34.459099 | 51.832743 | 53.164767 | 51.560839 | 84.335347 |
| TCGA-AB-2942-03A-01T-0734-13 | Low risk | 48.092637 | 58.581184 | 81.787834 | 70.901493 | 86.31854 | 114.70989 |
| TCGA-AB-2943-03A-01T-0740-13 | High risk | 36.127309 | 31.646748 | 46.678439 | 53.98821 | 49.864609 | 87.72963 |
| TCGA-AB-2944-03A-01T-0740-13 | High risk | 44.215478 | 47.50205 | 48.14918 | 32.603113 | 53.669896 | 52.422461 |
| TCGA-AB-2946-03A-01T-0740-13 | Low risk | 34.232178 | 38.846693 | 51.114754 | 38.91898 | 50.093371 | 54.180147 |
| TCGA-AB-2948-03A-01T-0740-13 | High risk | 32.554212 | 32.45154 | 47.150783 | 45.559854 | 62.667291 | 68.874777 |
| TCGA-AB-2949-03B-01T-0748-13 | High risk | 43.976829 | 49.229192 | 46.824391 | 31.741327 | 67.613244 | 63.512402 |
| TCGA-AB-2950-03A-01T-0735-13 | Low risk | 57.043412 | 72.845984 | 69.82654 | 74.239894 | 82.914229 | 90.255763 |
| TCGA-AB-2952-03B-01T-0760-13 | High risk | 44.134049 | 28.431208 | 41.808532 | 46.926965 | 52.640375 | 91.183575 |
| TCGA-AB-2955-03A-01T-0734-13 | Low risk | 59.659348 | 73.90265 | 83.625562 | 73.841419 | 64.988115 | 91.476206 |
| TCGA-AB-2956-03A-01T-0740-13 | High risk | 29.153702 | 29.73885 | 29.01984 | 38.278482 | 57.021845 | 46.550008 |
| TCGA-AB-2959-03A-01T-0734-13 | Low risk | 45.630741 | 63.157336 | 76.319863 | 53.068252 | 83.020589 | 122.94641 |
| TCGA-AB-2963-03A-01T-0734-13 | Low risk | 46.414604 | 67.129751 | 53.01937 | 61.748405 | 72.367832 | 58.044245 |
| TCGA-AB-2965-03A-01T-0734-13 | Low risk | 42.281667 | 61.726712 | 57.707005 | 78.981335 | 82.67168 | 85.26002 |
| TCGA-AB-2966-03A-01T-0734-13 | Low risk | 56.439763 | 72.800878 | 77.075071 | 69.270455 | 67.379926 | 105.68335 |
| TCGA-AB-2970-03A-01T-0734-13 | Low risk | 38.661692 | 48.639023 | 59.613165 | 54.437671 | 70.897841 | 87.22271 |
| TCGA-AB-2971-03A-01T-0734-13 | Low risk | 42.040714 | 46.32037 | 60.976062 | 60.286111 | 72.140482 | 105.01557 |
| TCGA-AB-2973-03A-01T-0734-13 | Low risk | 40.969171 | 47.249621 | 96.949857 | 167.74777 | 99.485092 | 227.3596 |
| TCGA-AB-2975-03A-01T-0734-13 | Low risk | 56.879536 | 55.36558 | 79.527234 | 87.191475 | 86.176334 | 105.30341 |
| TCGA-AB-2976-03A-01T-0734-13 | Low risk | 54.230754 | 58.835268 | 68.627931 | 97.405545 | 83.394858 | 109.01042 |
| TCGA-AB-2977-03B-01T-0760-13 | Low risk | 59.458031 | 41.503704 | 106.14046 | 132.36141 | 99.953759 | 200.5626 |
| TCGA-AB-2979-03B-01T-0760-13 | Low risk | 46.577988 | 38.771841 | 39.404968 | 40.087722 | 54.167187 | 56.462117 |
| TCGA-AB-2980-03A-01T-0734-13 | Low risk | 61.64352 | 78.466304 | 78.280871 | 83.841285 | 90.591052 | 109.46114 |
| TCGA-AB-2981-03B-01T-0748-13 | High risk | 30.845683 | 32.902953 | 40.223988 | 54.134581 | 59.235171 | 75.054441 |
| TCGA-AB-2982-03B-01T-0748-13 | Low risk | 43.145602 | 49.374124 | 76.443839 | 87.617676 | 71.342672 | 112.05149 |
| TCGA-AB-2983-03A-01T-0734-13 | High risk | 37.79083 | 43.645348 | 61.182615 | 73.953452 | 71.698449 | 87.91112 |
| TCGA-AB-2984-03A-01T-0734-13 | Low risk | 171.45268 | 122.56628 | 361.27957 | 340.28479 | 156.64922 | 494.45745 |
| TCGA-AB-2986-03A-01T-0734-13 | Low risk | 74.732274 | 74.36169 | 95.042244 | 105.27054 | 98.349103 | 155.53717 |
| TCGA-AB-2987-03A-01T-0734-13 | High risk | 31.046846 | 34.795898 | 77.717057 | 114.02939 | 81.521744 | 164.52775 |
| TCGA-AB-2988-03B-01T-0748-13 | High risk | 41.382331 | 51.234945 | 34.963827 | 28.285739 | 51.616653 | 39.583553 |
| TCGA-AB-2990-03B-01T-0748-13 | Low risk | 55.208839 | 51.598415 | 65.485328 | 69.201429 | 79.491016 | 120.90853 |
| TCGA-AB-2991-03A-01T-0735-13 | High risk | 46.023721 | 49.986231 | 60.867447 | 95.111837 | 76.355862 | 80.784536 |
| TCGA-AB-2992-03A-01T-0735-13 | Low risk | 37.827146 | 42.184696 | 61.640345 | 102.38329 | 75.51535 | 119.61429 |
| TCGA-AB-2994-03A-01T-0735-13 | Low risk | 38.467287 | 33.794193 | 53.710672 | 68.432043 | 67.573247 | 76.590094 |
| TCGA-AB-2995-03A-01T-0735-13 | High risk | 37.892859 | 37.363989 | 50.320373 | 40.050396 | 64.71402 | 78.401145 |
| TCGA-AB-2996-03A-01T-0735-13 | Low risk | 38.614665 | 46.85073 | 54.789274 | 58.940732 | 73.287875 | 90.966437 |
| TCGA-AB-2998-03A-01T-0735-13 | High risk | 42.917797 | 41.893649 | 57.138886 | 85.193791 | 71.18804 | 70.59694 |
| TCGA-AB-2999-03B-01T-0748-13 | Low risk | 51.601889 | 55.454188 | 80.793111 | 108.643 | 74.214751 | 138.60471 |
| TCGA-AB-3000-03A-01T-0736-13 | Low risk | 34.863681 | 41.81584 | 36.335318 | 34.692079 | 43.69219 | 36.413339 |
| TCGA-AB-3001-03A-01T-0736-13 | Low risk | 40.238225 | 39.822885 | 69.271003 | 81.991221 | 72.812259 | 81.670232 |
| TCGA-AB-3002-03A-01T-0736-13 | High risk | 51.460673 | 64.16993 | 71.305833 | 65.000111 | 86.453432 | 92.811003 |
| TCGA-AB-3007-03A-01T-0736-13 | High risk | 44.451403 | 47.710535 | 59.973562 | 79.871176 | 60.280012 | 62.51467 |
| TCGA-AB-3008-03A-01T-0736-13 | Low risk | 43.356331 | 45.610001 | 43.943283 | 31.180184 | 51.328059 | 46.381119 |
| TCGA-AB-3009-03A-01T-0736-13 | High risk | 35.920347 | 42.618015 | 49.268466 | 40.968221 | 61.038319 | 44.736828 |
| TCGA-AB-3011-03A-01T-0736-13 | Low risk | 43.717262 | 39.411896 | 37.064001 | 46.916159 | 57.607757 | 53.577337 |
| TCGA-AB-3012-03A-01T-0736-13 | Low risk | 51.264346 | 55.824777 | 87.677683 | 117.92832 | 90.262646 | 105.91834 |
| Median IC 50 | NA | 41.553874 | 43.722781 | 53.073608 | 58.940732 | 67.418363 | 75.780906 |

**Supplementary Table 8 (Continue)**

| **TCGA ID** | Group | Dabrafenib_1373 | 5-Fluorouracil_1073 | Leflunomide_1578 | Temozolomide_1375 |
| --- | --- | --- | --- | --- | --- |
| TCGA-AB-2805-03A-01T-0734-13 | Low risk | 119.49564 | 36.54533 | 137.58898 | 284.02258 |
| TCGA-AB-2806-03A-01T-0734-13 | Low risk | 113.3104 | 137.7072 | 135.36235 | 388.9952 |
| TCGA-AB-2808-03A-01T-0734-13 | Low risk | 109.70235 | 155.42347 | 127.89783 | 324.54926 |
| TCGA-AB-2810-03A-01T-0736-13 | High risk | 130.17448 | 233.25563 | 178.06645 | 591.23906 |
| TCGA-AB-2811-03B-01T-0760-13 | High risk | 103.58571 | 62.717842 | 133.58321 | 450.30216 |
| TCGA-AB-2812-03A-01T-0734-13 | Low risk | 134.39657 | 357.09653 | 194.18059 | 538.79961 |
| TCGA-AB-2813-03A-01T-0736-13 | High risk | 117.08595 | 105.40333 | 170.89582 | 377.46512 |
| TCGA-AB-2814-03A-01T-0734-13 | High risk | 117.22712 | 151.25844 | 171.05469 | 527.31348 |
| TCGA-AB-2815-03A-01T-0734-13 | Low risk | 166.92076 | 124.41165 | 200.6426 | 440.11192 |
| TCGA-AB-2817-03A-01T-0736-13 | High risk | 66.586039 | 33.307724 | 117.44419 | 263.06931 |
| TCGA-AB-2818-03A-01T-0734-13 | Low risk | 101.5917 | 50.614691 | 128.84501 | 278.53826 |
| TCGA-AB-2819-03A-01T-0734-13 | Low risk | 91.714531 | 89.171667 | 140.4373 | 435.78235 |
| TCGA-AB-2820-03A-01T-0735-13 | High risk | 76.221436 | 159.82868 | 115.17594 | 432.33669 |
| TCGA-AB-2821-03A-01T-0735-13 | Low risk | 121.05021 | 157.83121 | 147.40085 | 442.00987 |
| TCGA-AB-2822-03A-01T-0734-13 | Low risk | 136.34393 | 331.48837 | 147.8494 | 578.4984 |
| TCGA-AB-2823-03A-01T-0736-13 | High risk | 103.70293 | 102.96724 | 164.11519 | 409.13262 |
| TCGA-AB-2825-03A-01T-0736-13 | High risk | 94.825317 | 15.889141 | 140.15039 | 211.236 |
| TCGA-AB-2826-03A-01T-0734-13 | Low risk | 158.71184 | 129.79162 | 182.928 | 571.22537 |
| TCGA-AB-2828-03A-01T-0734-13 | Low risk | 98.869315 | 58.597897 | 155.98736 | 302.27268 |
| TCGA-AB-2830-03A-01T-0736-13 | High risk | 66.309298 | 100.94801 | 144.89024 | 531.12863 |
| TCGA-AB-2834-03A-01T-0734-13 | Low risk | 186.75901 | 163.17099 | 174.29218 | 415.38708 |
| TCGA-AB-2835-03A-01T-0736-13 | High risk | 82.766084 | 22.404348 | 144.83721 | 234.25437 |
| TCGA-AB-2836-03A-01T-0736-13 | High risk | 67.383902 | 23.782291 | 120.58743 | 186.38231 |
| TCGA-AB-2839-03A-01T-0734-13 | Low risk | 98.245109 | 368.07862 | 178.53344 | 431.78146 |
| TCGA-AB-2840-03A-01T-0734-13 | Low risk | 152.27643 | 282.48895 | 160.35405 | 768.74886 |
| TCGA-AB-2841-03B-01T-0760-13 | Low risk | 122.95667 | 334.91092 | 220.68689 | 870.2548 |
| TCGA-AB-2842-03A-01T-0734-13 | High risk | 254.04315 | 224.35271 | 237.7312 | 612.51619 |
| TCGA-AB-2843-03A-01T-0736-13 | High risk | 56.258041 | 55.923971 | 104.29383 | 245.03326 |
| TCGA-AB-2844-03A-01T-0736-13 | Low risk | 71.172843 | 52.974407 | 116.99315 | 271.38259 |
| TCGA-AB-2845-03B-01T-0748-13 | High risk | 59.508753 | 65.664649 | 94.332751 | 257.00387 |
| TCGA-AB-2846-03A-01T-0736-13 | High risk | 90.297497 | 65.150244 | 142.15099 | 297.84337 |
| TCGA-AB-2847-03A-01T-0736-13 | High risk | 73.531637 | 101.13216 | 152.05464 | 338.57226 |
| TCGA-AB-2849-03A-01T-0734-13 | Low risk | 263.16941 | 526.24605 | 135.94978 | 554.26582 |
| TCGA-AB-2851-03A-01T-0736-13 | High risk | 77.98299 | 39.528874 | 152.61979 | 272.76248 |
| TCGA-AB-2853-03A-01T-0734-13 | Low risk | 130.56396 | 127.35783 | 152.44549 | 386.00943 |
| TCGA-AB-2856-03A-01T-0736-13 | High risk | 46.041892 | 40.71293 | 129.17285 | 311.83842 |
| TCGA-AB-2857-03A-01T-0736-13 | High risk | 113.05677 | 133.61823 | 112.54741 | 258.15313 |
| TCGA-AB-2858-03A-01T-0736-13 | Low risk | 68.972024 | 150.62083 | 135.55326 | 472.77052 |
| TCGA-AB-2859-03A-01T-0736-13 | High risk | 84.468412 | 111.35445 | 132.48384 | 341.79562 |
| TCGA-AB-2861-03A-01T-0736-13 | Low risk | 57.049573 | 15.526073 | 109.23252 | 287.52311 |
| TCGA-AB-2862-03A-01T-0736-13 | Low risk | 76.211696 | 147.80391 | 139.71227 | 586.04657 |
| TCGA-AB-2863-03A-01T-0734-13 | High risk | 144.97972 | 150.10655 | 141.1842 | 407.32406 |
| TCGA-AB-2865-03A-01T-0736-13 | High risk | 88.203592 | 57.453703 | 165.74581 | 226.74447 |
| TCGA-AB-2866-03A-01T-0736-13 | High risk | 99.900384 | 23.700019 | 153.1845 | 260.99713 |
| TCGA-AB-2867-03A-01T-0734-13 | Low risk | 140.36134 | 360.1154 | 163.12922 | 619.61265 |
| TCGA-AB-2869-03A-01T-0735-13 | Low risk | 56.054228 | 109.14729 | 121.2041 | 320.08144 |
| TCGA-AB-2870-03A-01T-0735-13 | High risk | 90.487779 | 50.127016 | 132.13575 | 237.48731 |
| TCGA-AB-2871-03A-01T-0735-13 | Low risk | 44.38695 | 117.38002 | 120.20036 | 327.84545 |
| TCGA-AB-2872-03A-01T-0735-13 | Low risk | 172.34839 | 393.03724 | 201.21696 | 884.96857 |
| TCGA-AB-2873-03A-01T-0735-13 | High risk | 122.32612 | 60.471873 | 182.74195 | 391.28435 |
| TCGA-AB-2874-03A-01T-0735-13 | Low risk | 88.561789 | 83.957988 | 148.59003 | 277.66257 |
| TCGA-AB-2875-03A-01T-0735-13 | Low risk | 61.241774 | 90.944524 | 119.47935 | 286.1798 |
| TCGA-AB-2876-03A-01T-0734-13 | Low risk | 133.14734 | 231.90855 | 135.28114 | 502.80656 |
| TCGA-AB-2877-03A-01T-0735-13 | Low risk | 100.7666 | 84.566341 | 119.80367 | 340.12371 |
| TCGA-AB-2878-03A-01T-0734-13 | Low risk | 158.09546 | 581.86849 | 182.63419 | 554.87772 |
| TCGA-AB-2880-03A-01T-0735-13 | Low risk | 70.926571 | 66.656004 | 105.64285 | 188.08246 |
| TCGA-AB-2881-03A-01T-0735-13 | High risk | 94.691091 | 53.909109 | 131.18847 | 302.15114 |
| TCGA-AB-2882-03A-01T-0740-13 | Low risk | 102.58332 | 126.71528 | 150.49559 | 368.22092 |
| TCGA-AB-2883-03A-01T-0734-13 | Low risk | 149.62773 | 287.88696 | 142.78546 | 1297.3576 |
| TCGA-AB-2884-03A-01T-0735-13 | High risk | 66.326751 | 34.017606 | 124.19495 | 238.6903 |
| TCGA-AB-2885-03A-01T-0735-13 | High risk | 73.959738 | 156.89872 | 126.33106 | 295.69117 |
| TCGA-AB-2886-03A-01T-0735-13 | Low risk | 68.445454 | 77.827643 | 107.77751 | 270.36809 |
| TCGA-AB-2888-03B-01T-0748-13 | High risk | 75.003747 | 24.341201 | 111.7244 | 237.68202 |
| TCGA-AB-2889-03A-01T-0735-13 | Low risk | 95.449794 | 92.838186 | 158.74405 | 369.33404 |
| TCGA-AB-2890-03A-01T-0735-13 | High risk | 81.081864 | 76.672826 | 130.68947 | 299.93727 |
| TCGA-AB-2891-03A-01T-0735-13 | High risk | 78.893978 | 57.241888 | 113.68106 | 252.86591 |
| TCGA-AB-2892-03A-01T-0734-13 | Low risk | 139.67893 | 307.05964 | 215.82649 | 709.07904 |
| TCGA-AB-2893-03A-01T-0734-13 | High risk | 99.154079 | 29.16499 | 173.00536 | 361.2428 |
| TCGA-AB-2894-03A-01T-0734-13 | Low risk | 100.2172 | 37.141553 | 131.90907 | 420.44777 |
| TCGA-AB-2895-03A-01T-0735-13 | High risk | 92.581451 | 96.726824 | 145.9207 | 298.21974 |
| TCGA-AB-2896-03B-01T-0751-13 | High risk | 57.190039 | 59.004723 | 115.38172 | 261.43558 |
| TCGA-AB-2897-03A-01T-0735-13 | High risk | 146.9034 | 372.21394 | 208.47566 | 918.06652 |
| TCGA-AB-2898-03A-01T-0735-13 | High risk | 161.64083 | 197.02156 | 165.3294 | 551.48836 |
| TCGA-AB-2899-03A-01T-0736-13 | Low risk | 90.143669 | 50.529473 | 118.29045 | 369.06938 |
| TCGA-AB-2900-03A-01T-0735-13 | High risk | 99.999746 | 60.087699 | 162.11809 | 295.45192 |
| TCGA-AB-2901-03A-01T-0735-13 | High risk | 127.59019 | 156.65288 | 180.34927 | 332.69819 |
| TCGA-AB-2908-03A-01T-0740-13 | High risk | 100.35689 | 493.2129 | 196.16453 | 706.27619 |
| TCGA-AB-2910-03A-01T-0740-13 | High risk | 69.325507 | 13.884498 | 142.98849 | 350.86609 |
| TCGA-AB-2911-03A-01T-0734-13 | Low risk | 133.39865 | 264.44789 | 221.03811 | 656.57799 |
| TCGA-AB-2912-03A-01T-0734-13 | High risk | 162.59894 | 176.14931 | 179.84971 | 556.46875 |
| TCGA-AB-2913-03A-01T-0734-13 | Low risk | 132.86112 | 711.51181 | 198.62246 | 802.22181 |
| TCGA-AB-2914-03A-01T-0734-13 | Low risk | 136.54392 | 260.44514 | 197.7668 | 526.46484 |
| TCGA-AB-2915-03A-01T-0740-13 | High risk | 67.85924 | 37.41835 | 124.18332 | 205.83303 |
| TCGA-AB-2916-03A-01T-0734-13 | Low risk | 101.29449 | 140.50443 | 189.80281 | 572.89218 |
| TCGA-AB-2917-03A-01T-0734-13 | Low risk | 218.16622 | 476.88082 | 156.21218 | 579.60917 |
| TCGA-AB-2918-03A-01T-0740-13 | High risk | 76.003379 | 52.915165 | 116.52803 | 251.07089 |
| TCGA-AB-2919-03A-01T-0740-13 | Low risk | 73.010915 | 79.162896 | 104.74366 | 269.56972 |
| TCGA-AB-2920-03B-01T-0760-13 | High risk | 68.577456 | 113.69931 | 170.86068 | 380.78255 |
| TCGA-AB-2921-03A-01T-0740-13 | High risk | 53.710311 | 32.690562 | 108.42363 | 274.69757 |
| TCGA-AB-2924-03A-01T-0740-13 | High risk | 60.600258 | 33.293431 | 123.50564 | 228.16397 |
| TCGA-AB-2925-03A-01T-0735-13 | High risk | 83.659037 | 28.364852 | 144.33414 | 375.25022 |
| TCGA-AB-2927-03A-01T-0740-13 | High risk | 91.997278 | 100.33292 | 146.61669 | 567.55908 |
| TCGA-AB-2928-03A-01T-0740-13 | High risk | 82.830137 | 61.913647 | 146.13182 | 216.77139 |
| TCGA-AB-2929-03A-01T-0735-13 | High risk | 50.500044 | 37.668676 | 105.6128 | 199.51506 |
| TCGA-AB-2930-03A-01T-0740-13 | High risk | 46.015063 | 39.789221 | 107.03772 | 191.20572 |
| TCGA-AB-2931-03A-01T-0740-13 | High risk | 63.980601 | 47.378304 | 124.71487 | 202.57073 |
| TCGA-AB-2932-03A-01T-0740-13 | High risk | 91.37556 | 17.769456 | 145.03577 | 240.9706 |
| TCGA-AB-2933-03A-01T-0734-13 | High risk | 140.65267 | 241.88149 | 174.53152 | 734.48162 |
| TCGA-AB-2934-03A-01T-0740-13 | High risk | 71.640706 | 64.220557 | 135.11071 | 340.62915 |
| TCGA-AB-2935-03A-01T-0740-13 | High risk | 52.930397 | 51.664239 | 116.91578 | 330.35422 |
| TCGA-AB-2936-03A-01T-0740-13 | Low risk | 105.08972 | 174.28693 | 120.28844 | 345.30594 |
| TCGA-AB-2937-03A-01T-0734-13 | Low risk | 163.48023 | 1108.2664 | 415.36959 | 1437.0452 |
| TCGA-AB-2938-03A-01T-0736-13 | High risk | 119.0971 | 364.78341 | 148.7817 | 338.19917 |
| TCGA-AB-2939-03A-01T-0740-13 | High risk | 87.489905 | 55.77527 | 145.26381 | 280.04513 |
| TCGA-AB-2940-03A-01T-0736-13 | Low risk | 72.469339 | 129.6256 | 116.52119 | 275.22888 |
| TCGA-AB-2941-03A-01T-0740-13 | High risk | 70.857209 | 731.48419 | 138.88804 | 258.86956 |
| TCGA-AB-2942-03A-01T-0734-13 | Low risk | 104.74901 | 109.74586 | 159.02987 | 405.37184 |
| TCGA-AB-2943-03A-01T-0740-13 | High risk | 76.820732 | 328.70216 | 116.69248 | 482.57788 |
| TCGA-AB-2944-03A-01T-0740-13 | High risk | 77.938088 | 66.046898 | 121.27864 | 265.32624 |
| TCGA-AB-2946-03A-01T-0740-13 | Low risk | 75.292556 | 83.936113 | 107.40796 | 286.72501 |
| TCGA-AB-2948-03A-01T-0740-13 | High risk | 93.165533 | 50.882923 | 136.1866 | 330.74157 |
| TCGA-AB-2949-03B-01T-0748-13 | High risk | 90.933811 | 60.58768 | 123.06419 | 327.77408 |
| TCGA-AB-2950-03A-01T-0735-13 | Low risk | 123.18162 | 159.07489 | 164.45795 | 510.3557 |
| TCGA-AB-2952-03B-01T-0760-13 | High risk | 59.191733 | 101.84871 | 141.69964 | 292.24812 |
| TCGA-AB-2955-03A-01T-0734-13 | Low risk | 100.91321 | 505.5492 | 131.89682 | 450.75731 |
| TCGA-AB-2956-03A-01T-0740-13 | High risk | 91.467545 | 10.789494 | 119.06023 | 256.17015 |
| TCGA-AB-2959-03A-01T-0734-13 | Low risk | 141.87443 | 247.82407 | 157.0221 | 537.86835 |
| TCGA-AB-2963-03A-01T-0734-13 | Low risk | 121.53206 | 114.33834 | 156.26392 | 375.78262 |
| TCGA-AB-2965-03A-01T-0734-13 | Low risk | 132.25632 | 90.104635 | 188.78169 | 417.51597 |
| TCGA-AB-2966-03A-01T-0734-13 | Low risk | 110.0625 | 413.3793 | 126.60423 | 477.90583 |
| TCGA-AB-2970-03A-01T-0734-13 | Low risk | 101.5691 | 124.36905 | 155.61512 | 424.85767 |
| TCGA-AB-2971-03A-01T-0734-13 | Low risk | 119.46187 | 118.85947 | 139.12971 | 531.2953 |
| TCGA-AB-2973-03A-01T-0734-13 | Low risk | 158.35478 | 182.96748 | 208.69577 | 663.94458 |
| TCGA-AB-2975-03A-01T-0734-13 | Low risk | 112.20676 | 165.6149 | 166.46752 | 533.81554 |
| TCGA-AB-2976-03A-01T-0734-13 | Low risk | 130.36431 | 348.39627 | 174.73737 | 568.45812 |
| TCGA-AB-2977-03B-01T-0760-13 | Low risk | 79.398104 | 108.53162 | 167.98814 | 597.16488 |
| TCGA-AB-2979-03B-01T-0760-13 | Low risk | 38.71456 | 96.586459 | 123.79536 | 244.27159 |
| TCGA-AB-2980-03A-01T-0734-13 | Low risk | 140.17258 | 409.3428 | 176.43635 | 620.58168 |
| TCGA-AB-2981-03B-01T-0748-13 | High risk | 92.482162 | 11.689714 | 119.77402 | 172.54039 |
| TCGA-AB-2982-03B-01T-0748-13 | Low risk | 132.66777 | 288.46053 | 152.17058 | 854.69779 |
| TCGA-AB-2983-03A-01T-0734-13 | High risk | 106.11955 | 211.35939 | 152.19216 | 506.79024 |
| TCGA-AB-2984-03A-01T-0734-13 | Low risk | 218.81348 | 659.03208 | 316.86266 | 1835.6266 |
| TCGA-AB-2986-03A-01T-0734-13 | Low risk | 80.800546 | 296.72087 | 186.90075 | 672.15406 |
| TCGA-AB-2987-03A-01T-0734-13 | High risk | 167.27191 | 48.767326 | 175.62465 | 451.4556 |
| TCGA-AB-2988-03B-01T-0748-13 | High risk | 64.015147 | 56.503071 | 104.62591 | 228.23665 |
| TCGA-AB-2990-03B-01T-0748-13 | Low risk | 104.11512 | 153.82516 | 130.31751 | 573.81734 |
| TCGA-AB-2991-03A-01T-0735-13 | High risk | 118.70438 | 208.03166 | 168.02564 | 519.88133 |
| TCGA-AB-2992-03A-01T-0735-13 | Low risk | 143.12405 | 179.13432 | 192.53402 | 558.12236 |
| TCGA-AB-2994-03A-01T-0735-13 | Low risk | 105.07853 | 78.545353 | 154.16047 | 384.78473 |
| TCGA-AB-2995-03A-01T-0735-13 | High risk | 101.38818 | 412.96498 | 144.09123 | 415.70285 |
| TCGA-AB-2996-03A-01T-0735-13 | Low risk | 110.31515 | 173.01641 | 160.67133 | 397.19172 |
| TCGA-AB-2998-03A-01T-0735-13 | High risk | 131.15447 | 89.478066 | 162.50712 | 465.88544 |
| TCGA-AB-2999-03B-01T-0748-13 | Low risk | 123.91822 | 374.82579 | 159.38954 | 882.95013 |
| TCGA-AB-3000-03A-01T-0736-13 | Low risk | 73.385287 | 67.549498 | 101.12161 | 233.35622 |
| TCGA-AB-3001-03A-01T-0736-13 | Low risk | 130.63608 | 295.72424 | 181.65004 | 607.97351 |
| TCGA-AB-3002-03A-01T-0736-13 | High risk | 131.30771 | 147.0547 | 143.23463 | 362.18808 |
| TCGA-AB-3007-03A-01T-0736-13 | High risk | 103.9803 | 198.59529 | 145.68379 | 592.59763 |
| TCGA-AB-3008-03A-01T-0736-13 | Low risk | 82.873478 | 155.8873 | 122.07938 | 253.19727 |
| TCGA-AB-3009-03A-01T-0736-13 | High risk | 80.717861 | 120.89106 | 130.91917 | 279.29832 |
| TCGA-AB-3011-03A-01T-0736-13 | Low risk | 119.06273 | 102.60662 | 114.94785 | 327.18111 |
| TCGA-AB-3012-03A-01T-0736-13 | Low risk | 124.44022 | 307.84018 | 211.03661 | 742.34105 |
| Median IC 50 | NA | 100.2172 | 111.35445 | 144.83721 | 375.78262 |
