# Supplementary material for: Identification and validation of a 7-genes prognostic signature for adult acute myeloid leukemia based on aging-related genes
Source: Aging (Albany NY). 2023 Jun 26;15(12):5826–53. doi: 10.18632/aging.204843 (PMC10333094; doi:10.18632/aging.204843)
Supplement: Supplementary Table 9 [file aging-15-204843-s010.pdf]

Supplementary Table 9. Univariate COX regression analysis for the overall survival.

Characteristics	Total (N)	Univariate analysis	
		Hazard ratio (95% CI)	P value
Risk Score	140	2.718 (1.810-4.081)	<0.001
Age	140	1.042 (1.026-1.058)	<0.001
Cytogenetics risk	138		
Favorable	31	1	
Intermediate	76	2.957 (1.498-5.836)	0.002
Poor	31	4.157 (1.944-8.893)	<0.001
PB-Blast (%)	140	1.003 (0.995-1.012)	0.445
BM-blast (%)	140	1.002 (0.995-1.009)	0.572
Hemoglobin (g/L)	139	1.117 (0.958-1.301)	0.157
WBC ($\times 10^9$/L)	139	1.005 (1.000-1.010)	0.069
PLT ($\times 10^9$/L)	140	1.000 (0.997-1.004)	0.842
Gender	140		
Male	77	1	
Female	63	0.971 (0.636-1.483)	0.892
Race	140		
White	127	1	
Others	10	1.154 (0.377-1.991)	0.736
FLT3 mutation	136		
Negative	97	1	
Positive	39	1.271 (0.801-2.016)	0.309
RAS mutation	139		
Negative	131	1	
Positive	8	0.643 (0.235-1.760)	0.39
NPM1 mutation	139		
Negative	106	1	
Positive	33	1.137 (0.706-1.832)	0.596
IDH1 mutation	138		
Negative	126	1	
Positive	12	0.588 (0.238-1.452)	0.249

Abbreviations: CI, confidence interval; FLT3, FMS-like tyrosine kinase 3; NPM1, Nucleophosmin; IDH1, Isocitrate dehydrogenase 1; PB, Peripheral blood; BM, Bone marrow; WBC, White blood cell; PLT, Platelet.
